# Regio- and enantioselective umpolung *gem-*difluoroallylation of hydrazones via palladium catalysis enabled by *N*-heterocyclic carbene ligand

**DOI:** 10.1038/s41467-021-26667-0

**Published:** 2021-11-12

**Authors:** Shuai Huang, Fei-Fei Tong, Da-Chang Bai, Gao-Peng Zhang, Yang-Jie Jiang, Bo Zhang, Xuebing Leng, Ying-Long Guo, Xiao-Long Wan, Xingang Zhang, Chang-Hua Ding, Xue-Long Hou

**Affiliations:** 1 State Key Laboratory of Organometallic Chemistry, Center for Excellence in Molecular Synthesis, Shanghai Institute of Organic Chemistry (SIOC), Chinese Academy of Sciences (CAS), Shanghai, China; 2 Key Laboratory of Organofluorine Chemistry, Center for Excellence in Molecular Synthesis, SIOC, CAS, Shanghai, China; 3 Department of Analytic Chemistry, Center for Excellence in Molecular Synthesis, SIOC, CAS, Shanghai, China; 4 Department of Chemistry, Innovative Drug Research Center, Shanghai University, Shanghai, China; 5 Shanghai-Hong Kong Joint Laboratory in Chemical Synthesis, SIOC, CAS, Shanghai, China

**Keywords:** Synthetic chemistry methodology, Homogeneous catalysis

## Abstract

The enantioselective construction of C–CF_2_R (R: alkyl or fluoroalkyl) bonds has attracted the attention of synthetic chemists because of the importance of chiral fluorinated compounds in life and materials sciences. Catalytic asymmetric fluoroalkylation has mainly been realized under organocatalysis and Lewis acid catalysis, with substrates limited to carbonyl compounds. Few examples using transition-metal catalysis exist, owing to side reactions including decomposition and isomerization of fluoroalkylating reagents. Herein we report umpolung asymmetric difluoroallylation of hydrazones with 3-bromo-3,3-difluoropropene (BDFP) under palladium catalysis. Difluoroallylation products having quaternary chiral carbon centers are afforded in good yields with high α/γ- and enantioselectivities. The usefulness of the reaction products is demonstrated and an inner-sphere mechanism of the reaction is proposed. The use of chiral *N*-heterocyclic carbene as ligand is the key for the selectivities as well as the productivity of the reaction.

## Abstract (web summary)

Installation of difluoroalkyl groups while also imparting stereochemical information is mostly only possible with organocatalytic methods that activate carbonyls. Here the authors show a method to perform an difluoroallylation of hydrazones, forming a masked amine stereocenter, via palladium- and *N*-heterocyclic-carbene catalysis.

## Introduction

**Fig. 1:**
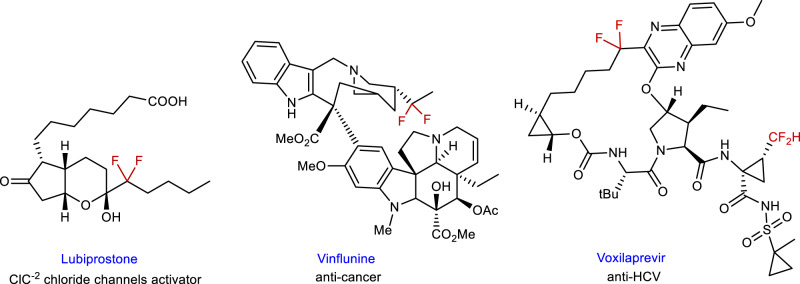
Chiral molecules containing a difluoroalkyl substituent. Examples of pharmaceuticals containing the difluoroalkylated chiral center.

**Fig. 2:**
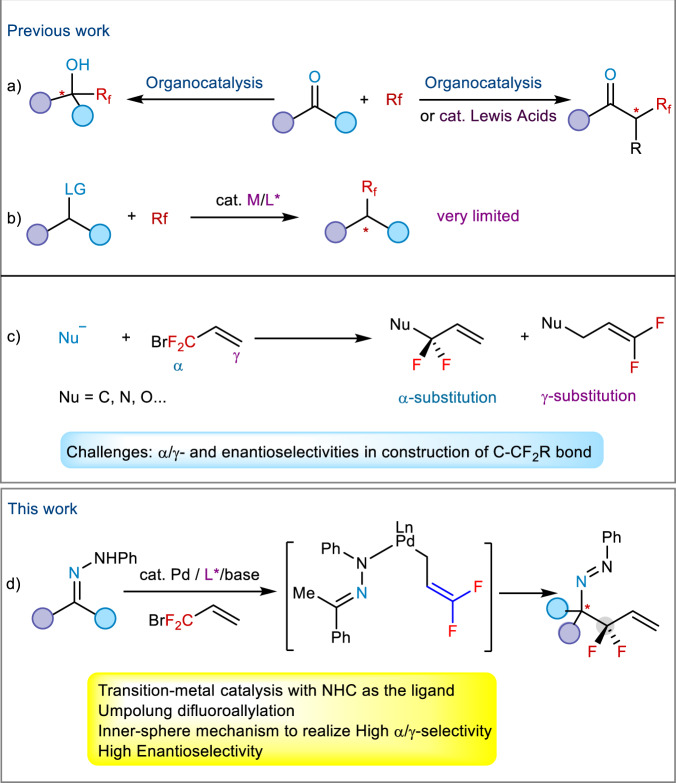
Asymmetric fluoroalkylations. **a** Asymmetric fluoroalkylation under organocatalysis and Lewis acid catalysis. **b** Asymmetric fluoroalkylation under transition-metal catalysis. **c** Regioselectivity in the reaction of nucleophiles with BDFP. **d** Palladium-catalyzed umpolung regio- and enantioselective allylation of hydrazones with BDFP. The circles with different color = different substituents.

The site-selective introduction of fluorinated groups into organic molecules has received extensive attention due to the important applications of fluorinated compounds in pharmaceuticals, agrochemicals, and materials sciences (Fig. 1)^1,2^. Over the past decades, tremendous efforts have been made in synthetic chemistry of fluorine-containing compounds, most of which focus on the direct fluoroalkylation of aromatic frameworks^3–5^, while less attention has been paid to the fluoroalkylation of aliphatic substrates^6^. Asymmetric fluoroalkylation has mainly been developed under organocatalysis^7–17^ and Lewis acid catalysis^18–20^, the substrates being limited to carbonyl compounds (Fig. 2a). Although transition-metal catalysis has been a powerful tool in organic synthesis, few successful examples in synthetic fluorine chemistry have appeared by using this strategy (Fig. 2b)^21–25^ because it is prone to generate side reactions, such as decomposition or isomerization of fluoroalkylating reagents. How to construct the C–CF_2_R bonds at the stereogenic center enantioselectively and efficiently under transition-metal catalysis to meet the increased demanding of life and materials sciences should be a crucial issue to be addressed.

We are interested in the introduction of *gem-*difluoroallyl group into organic molecules in a catalytic asymmetric manner, because the transformations of its carbon-carbon double bond can lead to diversified chiral difluoroalkylated compounds, which have important applications in pharmaceuticals (Fig. 1). We have developed a palladium-catalyzed *gem-*difluoroallylation of arylborons with BDFP^26^ and some methodologies in palladium-catalyzed asymmetric allylic alkylation^27–30^. We questioned whether BDFP^31,32^ could be used in palladium-catalyzed asymmetric *gem-*difluoroallylation. One of the crucial issues in this process is the regioselectivity (α/γ-selectivity), in addition to the challenge of constructing C-CF_2_R bond enantioselectively, because previous reports demonstrated that γ-substitution of BDFP is a favorable pathway for the reaction with carbon or nitrogen nucleophile^33^ (Fig. 2c).

Herein, we disclose our preliminary results of our investigations on a palladium-catalyzed asymmetric umpolung *gem-*difluoroallylation of hydrazones using a newly synthesized chiral NHC as the ligand, affording *gem-*difluoroallylated products bearing a quaternary chiral carbon center with high α- and enantioselectivities (Fig. 2d)^34–38^. A Pd-mediated [3,3]-reductive elimination process (inner-sphere mechanism) is proposed for the high selecitivities^37–46^. The resulting products are easily converted into some other chiral fluoro-containing products, including chiral amines, which play an important role in medicinal chemistry^47–49^.

## Results

### Influence of the reaction parameters on the reaction

**Fig. 3:**
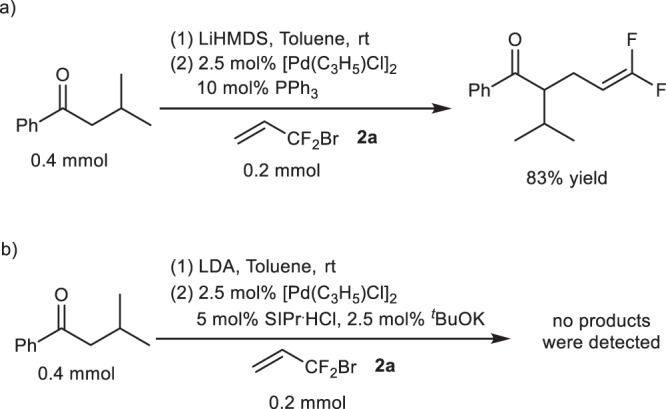
Pd-Catalyzed reaction of ketone with BDFP. **a** Palladium-catalyzed reaction of ketone with BDFP with PPh_3_ as the ligand. **b** Palladium-catalyzed reaction of ketone with BDFP using NHC as the ligand.

**Fig. 4:**
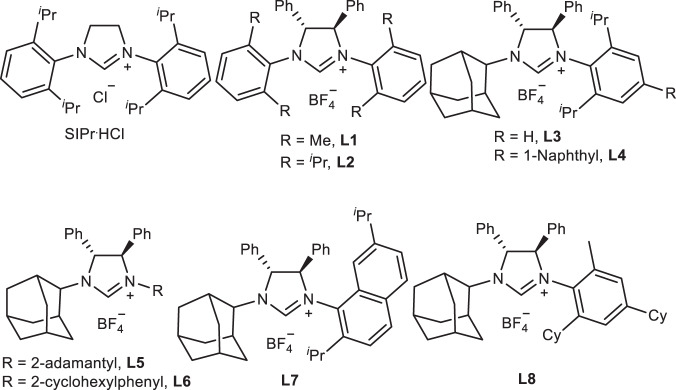
*N*-Heterocyclic carbenes. Structures of NHC ligands **L1**–**L8** tested in Table 1.

At the beginning of our investigation, we carried out the reaction of 2-methyl-1-phenylpropan-1-one with BDFP (**2a**) by using Pd/PPh_3_ as the catalyst. However, only γ-substituted product was produced (Fig. 3a). From the literatures and our own experiences, the regiochemistry in Pd-catalyzed allylic alkylation reaction could be different when the reaction proceeds via inner- or outer-sphere mechanism^40–46^. Thus, 1,3-bis(2,6-di-*i*-propylphenyl)imidazolidine-2-ylidene (SIPr), a NHC ligand previously used by us in Pd-catalyzed allylic alkylation of ketones via inner-sphere mechanism successfully, was tested as the ligand^45,46^. However, the reaction failed to proceed (Fig. 3b). Considering that enolate and aza-allyl anion are formed during the reaction of ketones and imines as nucleophile in Pd-catalyzed allylic alkylation^44–46^, and hydrazone has similar structure, we envisioned that hydrazone may be a suitable nucleophile for this allylation reaction. Pleasantly, allylation products were obtained in 86% yield with a ratio of **3a/4a/5a** = 81/0/19 by the reaction of hydrazone **1a** with BDFP (**2a**) using [Pd(C_3_H_5_)Cl]_2_ and SIPr as the catalyst (Table 1, entry 1). Obviously, the *N*-allylation product **5a** was obtained via an SN_2_′ reaction with nitrogen as the nucleophile because **5a** could be obtained without **L** and Pd (Table 1, entry 2), while α- and γ-substituted products **3a** and **4a** are umpolung allylic alkylation products. However, the chemoselectivity (C-/N-allylation) and the reaction efficiency were lower. To improve the chemoselectivity and to synthesize **3a** enantioselectively, a series of ligands were tested (Table 1, Fig. 4). Phosphine ligands led to γ-substituted **4a** as major product or sole formation of **5a** (Table 1, entries 3 and 4). However, chiral NHC ligand **L1** could provide allylation products in 48% yield with 50/0/50 ratio of **3a/4a/5a** and 56:44 er for **3a** (Table 1, entry 5). The yield increased to 93% with 57/7/36 ratio of **3a/4a/5a** and 68.5:31.5 er for **3a** by using a bulkier **L2** as the ligand (Table 1, entry 6). The er value of **3a** could increase further to 90.5:9.5 when **L3** possessing an adamantyl (Ad) as one of the substituents on nitrogen was used (Table 1, entry 7). No γ-substitution product **4** was produced under these reaction conditions, but chemoselectivity was poor. No further improvement in chemo- and enantioselectivities was observed when NHC ligands **L4**–**L7** with different substituents on nitrogen were used (Table 1, entries 8–11). Based on the structures of **L3** and **L7**, we also designed and synthesized new NHC ligand **L8**. When it was used, the er increased a little, but the chemoselectivity was still low (Table 1, entry 12). To improve the efficiency and the selectivities of the reaction, the impact of other parameters on the reaction were investigated by using **L8** as the ligand (Table 1). The results showed that the solvents we screened slightly affected the efficiency and the selectivities of the reaction (Table 1, entries 13–15), while the base has great influences on them. The reaction delivered the products in 86% yield with 57/0/43 ratio of **3a/4a/5a** and 93.5:6.5 er for **3a** when LiHMDS [HMDS: bis(trimethylsilyl)amide] was used as the base (Table 1, entry 12). Worse results were obtained if NaHMDS and KHMDS were the base (Table 1, entries 16 and 17). The efficiency and the selectivities were greatly improved, however, yield being 96%, the ratio of **3a/4a/5a** being >99:0:1 with 95:5 er for **3a**, if lithium diisopropylamide (LDA) was the base (Table 1, entry 18). Similar results were obtained by using *n*-BuLi as the base (Table 1, entry 19). If the reaction ran at lower temperature, similar enantioselectivity was observed, but the yield and the chemoselectivity were poorer (Table 1, entry 20). As comparison, the reaction with allyl bromide was also tested under the reaction condition of entry 18, Table 1. Only 24% yields of umpolung allylation and *N*-allylation products were obtained in a ratio of 27:73 with 86.5:13.5 er for umpolung product (not showed in Table 1). These results might reflect the importance of the difluoromethylene moiety of **2a** regarding the efficiency and the selectivities of the reaction.

**Table 1:** Influence of the reaction parameters on the reaction^a^.


Entry	Base	Solvent	Ligand	3a/4a/5a^b^	Yield%^b^	er%^c^
1	LiHMDS	Toluene	SIPr·HCl	81/0/19	86	–
2	LiHMDS	Toluene	None **L** & Pd	0/0/100	94	–
3	LiHMDS	Toluene	PPh_3_	5/75/20	80	–
4	LiHMDS	Toluene	(*R*)-BINAP^d^	0/0/100	78	–
5	LiHMDS	Toluene	**L1**	50/0/50	48	56:44
6	LiHMDS	Toluene	**L2**	57/7/36	93	68.5:31.5
7	LiHMDS	Toluene	**L3**	50/0/50	99	90.5:9.5
8	LiHMDS	Toluene	**L4**	57/0/43	79	91.5:8.5
9	LiHMDS	Toluene	**L5**	8/0/92	98	nd^e^
10	LiHMDS	Toluene	**L6**	1/0/99	98	nd^e^
11	LiHMDS	Toluene	**L7**	45/0/55	75	90:10
12	LiHMDS	Toluene	**L8**	57/0/43	86	93.5:6.5
13	LiHMDS	DCM	**L8**	–	trace	–
14	LiHMDS	THF	**L8**	7/0/93	71	nd^e^
15	LiHMDS	Hexane	**L8**	30/0/70	99	86:14
16	NaHMDS	Toluene	**L8**	19/0/81	84	91:9
17	KHMDS	Toluene	**L8**	3/0/97	67	nd^e^
18	LDA	Toluene	**L8**	>99/0/<1	96	95:5
19	*n*-BuLi	Toluene	**L8**	>99/0/<1	98	94.5:5.5
20^f^	LDA	Toluene	**L8**	72/0/28	67	95.5:4.5

^a^Reaction conditions: **1a**/base/**2a**/[Pd(C_3_H_5_)Cl]_2_/**L** = 200/200/100/2.5/5; 0.05 M of **2a**.

^b^Ratio and yield of **3a/4a/5a** were determined by ^19^F NMR of crude products with trifluoromethylbenzene as the internal standard.

^c^er value was determined by Chiral HPLC.

^d^BINAP = 2,2’-bis(diphenylphosphino)-1,1’-binaphthalene.

^e^Not determined.

^f^Ran at 0 °C.

### Substrate scope

**Fig. 5:**
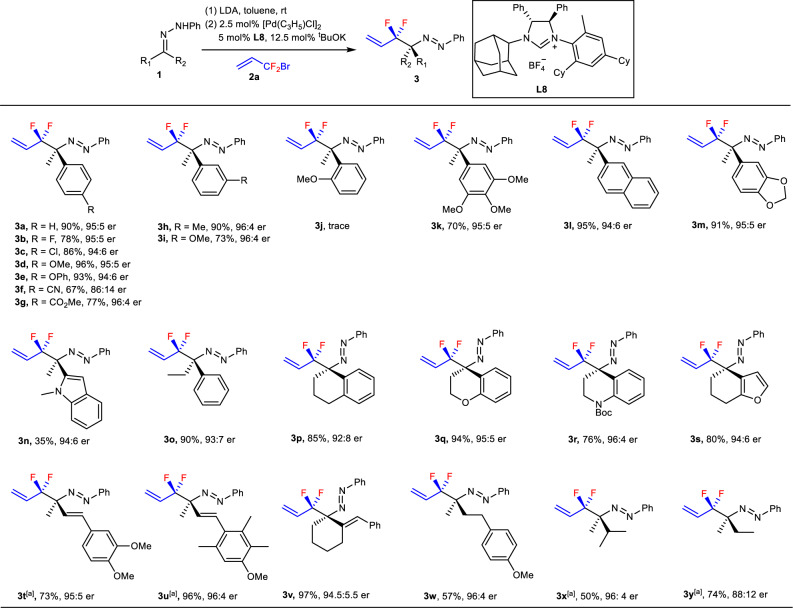
Substrate scope of Pd-catalyzed asymmetric umpolung difluoroallylation of different hydrazones 1 with allyl reagent **2a**. Reaction conditions: **1**/LDA/**2a**/[Pd(C_3_H_5_)Cl]_2_/**L8** = 200/200/100/2.5/5; 0.05 M of **2a**; **3/4/5** ratio was determined by ^19^F NMR of crude products, trifluoromethylbenzene as the internal standard; yield was the isolated yields for **3**; er value was determined by chiral HPLC. ^[a]^Ran at 10 °C.

**Fig. 6:**
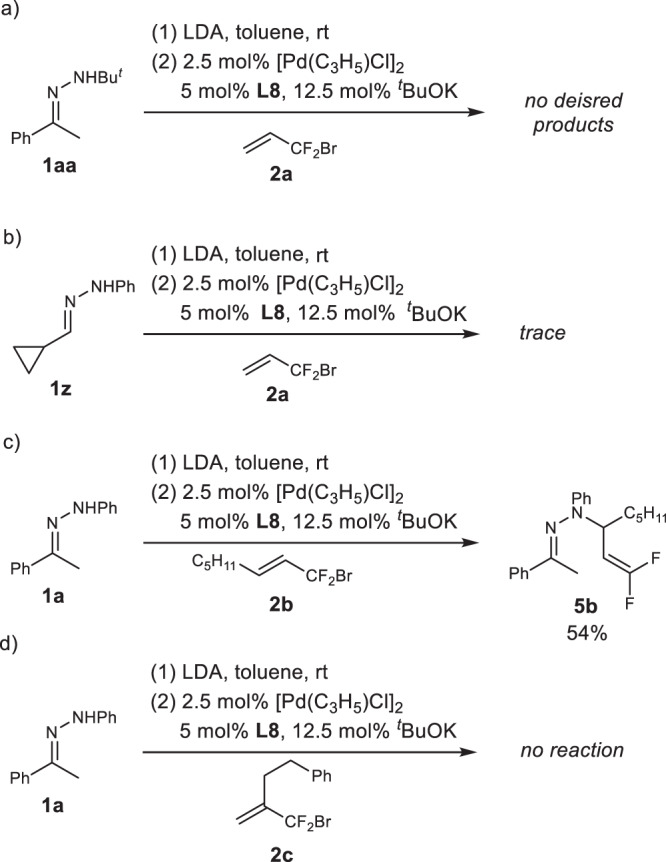
Examples of unsuccessful palladiumd-catalyzed asymmetric umpolung difluoroallylation. **a** Palladium-c-atalyzed reaction of *N-tert*-Bu hydrazone **1aa** with BDFP. **b** Palladium-catalyzed reaction of hydrazone **1z** with BDFP using **L8** as the ligand. **c** Palladium-catalyzed reaction of hydrazone **1a** with (*E*)-1-bromo-1,1-difluorooct-2-ene (**2b**). **d** Palladium-catalyzed reaction of hydrazone **1a** with (3-(bromodifluoromethyl)but-3-en-1-yl)benzene (**2c**).

With the viable reaction conditions in hand, the substrate scope of the reaction was investigated (Fig. 5). It can be seen that a wide range of α-substituted products **3** with R_1_ as phenyl bearing electron-withdrawing groups (**3b, 3c, 3** **f, 3** **g**) or electron-donating groups (**3d, 3e, 3h, 3i, 3k, 3m**) at *m*- or *p*-position were afforded in 67–96% yields with 86:14-96:4 er. The substituent R_1_ of compounds **3** can also be naphthyl and 2-indolyl (**3l** and **3n**), the enantioselectivities being 94:6 and 95:5 er, respectively, but the yield for **3n** was a little bit lower. However, the reaction was sensitive to the steric effect, substrate with R_1_ as *o*-substituted phenyl failed to deliver the product (**3j**). Hydrazones derived from tetralone and its derivatives as well as from α, β-unsaturated ketones were also suitable substrates, delivering **3p**–**s** and **3t**–**v** as the products in high yields and high enantioselectivities. The hydrazone derived from propiophenone also underwent the current palladium-catalyzed process smoothly to provide product **3o** in 90% with 93:7 er. Noteworthy is that the aliphatic hydrazones were also suitable substrate, affording α-substituted products **3x** and **3y** with Me, *i*-Pr and Me, Et as the substituents in 50% and 74% yields with 96:4 and 88:12 er, respectively. It was found that the presence of phenyl group on nitrogen should be important, the reaction failed to afford allylation product if *N*-*tert*-Bu hydrazone **1aa** was the substrate (Fig. 6a). Only trace products were afforded when hydrazone **1z** derived from cyclopropanecarbaldehyde was the substrate (Fig. 6b) although α-substituted product in 75% yield was obtained if SIPr·HCl was used as the ligand (not showed in Fig. 6). The reactions of hydrazone **1a** with substituted BDFP, (*E*)-1-bromo-1,1-difluorooct-2-ene (**2b**) and (3-(bromodifluoromethyl)but-3-en-1-yl)benzene (**2c**), were also unsuccessful, only *N*-allylation with γ-selectivity and trace α-substituted products were observed respectively (Fig. 6c and d).

### Synthetic applications

**Fig. 7:**
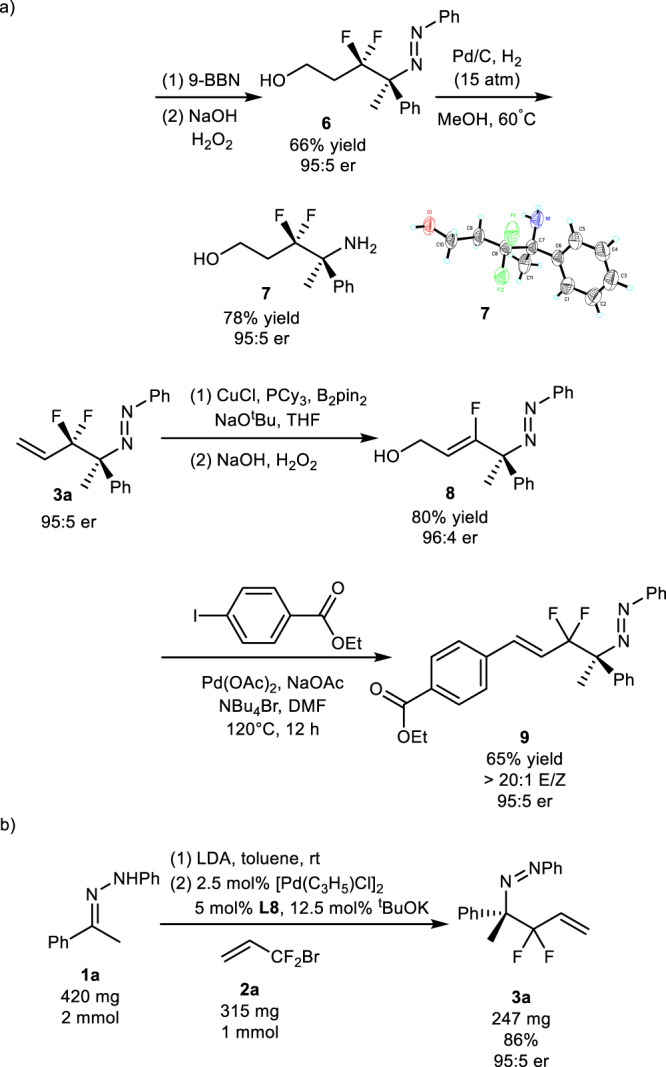
Transformation of reaction product and one mmol scale reaction. **a** Reactions of **3a** under different reaction conditions. **b** Pd-Catalyzed asymmetric umpolung allylation of hydrazine **1a** with BDFP **2a** on mmol-scale.

To demonstrate the utility of the methodology, various conversions of the reaction products were carried out (Fig. 7a). The N=N and C=C bonds of difluoroallylation product **3a** were transformed into NH_2_ and C–C–OH groups, respectively, keeping er ratio of product **7** unchanged. The Heck reaction of **3a** produced the desired product **9** in 65% yield with >20:1 *E/Z* ratio and 95:5 er. Treatment of **3a** with B_2_pin_2_ under copper catalysis followed by oxidation provided mono-fluoro allylalcohol **8** in 80% yield with 96:4 er (Fig. 7a). Since these products such as fluorinated amino alcohols have important applications in medicinal chemistry^47–49^, this protocol provides an efficient route for applications in drug discovery and development. One-mmol scale reaction also proceeded to deliver similar results (Fig. 7b). The absolute configuration of product **7** was determined as (*S*) by X-ray diffraction analysis of its single crystal (Fig. 7).

### Mechanistic study

**Fig. 8:**
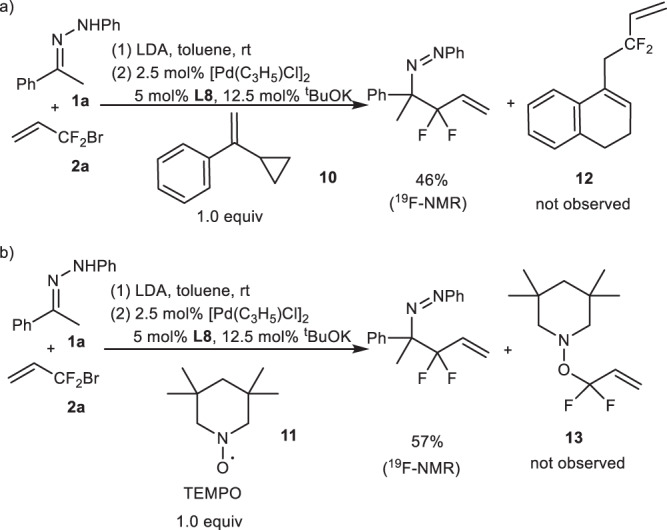
Control experiments. **a** Palladium-catalyzed reaction of hydrazone **1a** with BDFP **2** in the presence of radical clock **10**. **b** Pd-Catalyzed reaction of hydrazone **1a** with BDFP **2** in the presence of TEMPO **11**.

**Fig. 9:**
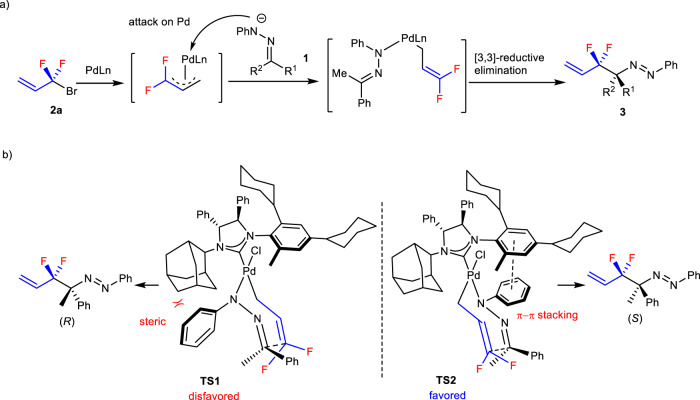
Proposed reaction mechanism. **a** Inner-sphere process for the formation of **3**. **b** Proposed transition states of the reaction.

It should be possible that the reaction of BDFP under transition-metal catalysis proceeds via radical mechanism^50^. Thus, control experiments by using radical clock probe **10** and TEMPO **11** were carried out (Fig. 8), from which the radical mechanism could be ruled out. In Pd-catalyzed allylic alkylation, there are two ways, inner- or outer-sphere processes, to form allylation products. In our previous works, the reactions using PPh_3_ as the ligand proceeded via outer-sphere mechanism to afford linear products predominantly, while that using NHC as the ligand underwent inner-sphere process to provide branched products^45,46^. Based upon these results and the literature reports^37–39,43–45^ as well as the present observations, the inner-sphere mechanism could be proposed for our present study, that is, the reaction proceeded through the attack of the nitrogen anion of hydrazone on the Pd of Pd-π difluoroallyl complex, followed by Pd-mediated [3,3]-reductive elimination (Fig. 9a). With this proposal and the results from the reaction using *N*-*tert* Bu hydrazone **1aa** as the reagent (Fig. 6a), the possible transition states of the reaction could be proposed, in which π-π stacking between ligand and phenyl group on nitrogen of hydrazone should play the role, and products in (*S*)-configuration would be obtained via favored transition state TS2 (Fig. 9b). It should be noted that the detailed reaction mechanism should be studied further.

In conclusion, a regio- and enantioselective *gem-*difluoroallylation of hydrazones was realized under Pd/NHC catalysis in an umpolung manner. The usefulness of the protocol was demonstrated. A rational inner-sphere mechanism was proposed. The use of bulky chiral NHC ligand overcomes the challenges in chemo-, regio-, and enantioselective *gem-*difluoroallylation of hydrazones, paving a way for the catalytic asymmetric synthesis of fluorinated compounds. Detailed investigation of the reaction mechanism as well as the exploration of further applications of BDFP in asymmetric fluoroalkylations are underway.

## Methods

### General procedure for *gem-*difluoroallylation of hydrazones

A dry Schlenk tube was flame dried and flushed with Argon. Hydrazone **1** (0.4 mmol) and toluene (2.0 mL) were added into the dry Schlenk tube. LDA (1.0 M in THF, 0.4 mL, 0.4 mmol) was added at 0 °C and stirred at room temperature for 30 min. In a separated flask, [Pd(C_3_H_5_)Cl]_2_ (1.83 mg, 0.005 mmol), **L8** (7.1 mg, 0.01 mmol) and toluene (1.0 mL) were mixed, followed by addition of *t*-BuOK (1.0 M in THF, 25 μL, 0.025 mmol) at rt. The resulting mixture was stirred at room temperature for 30 min, then added to the hydrazone solution. The BDFP **2** (31.5 mg, 0.2 mmol) and toluene (1.0 mL) was then added and the mixture was stirred at room temperature. After the reaction was completed, the reaction mixture was quenched by H_2_O (0.3 mL). The regio- and diastereoselectivities were then determined by ^19^F NMR spectroscopy by using trifluoromethylbenzene (24 μL) as an internal standard. After this analysis, the crude reaction mixture was dried (anhydrous Na_2_SO_4_) and then filtered through a 0.5 inch plug of silica gel (eluting with AcOEt) to remove the solid. The crude reaction mixture was concentrated under reduced pressure and then purified by preparative TLC (petroleum ether/ethyl acetate = 50/1) to afford products.

## Supplementary information

Supplementary Information

## Data Availability

Detailed experimental procedures and characterization of compounds as well as NMR and HPLC spectra can be found in the Supplementary Information. The X-ray crystallographic coordinates for structure reported in this study have been deposited at the Cambridge Crystallographic Data Centre (CCDC) under deposition number CCDC 2032248. The data can be obtained free of charge from The Cambridge Crystallographic Data Centre via www.ccdc.cam.ac.uk/data_request/cif.
