# Supplementary material for: Regio- and enantioselective umpolung gem-difluoroallylation of hydrazones via palladium catalysis enabled by N-heterocyclic carbene ligand
Source: Nat Commun. 2021 Nov 12;12:6551. doi: 10.1038/s41467-021-26667-0 (PMC8589859; doi:10.1038/s41467-021-26667-0)
Supplement: Supplementary file 1 — Supplementary Information [file 41467_2021_26667_MOESM1_ESM.pdf]

Supplementary Information

Regio- and Enantioselective Umpolung *gem*-Difluoroallylation of Hydrazones via Palladium Catalysis Enabled by *N*-Heterocyclic Carbene Ligand

Shuai Huang,[1] Fei-Fei Tong,[2] Da-Chang Bai,[1] Gao-Peng Zhang,[1] Yang-Jie Jiang,[1] Bo Zhang,[1] Xuebing Leng,[1] Ying-Long Guo,[1] Xiao-Long Wan,[3] Xingang Zhang,[2,*] Chang-Hua Ding,[4,*] and Xue-Long Hou[1,5,*]

[1]State Key Laboratory of Organometallic Chemistry, Center for Excellence in Molecular Synthesis, Shanghai Institute of Organic Chemistry (SIOC), Chinese Academy of Sciences (CAS), Shanghai, China.

[2]Key Laboratory of Organofluorine Chemistry, Center for Excellence in Molecular Synthesis, SIOC, CAS, Shanghai, China.

[3]Department of Analytic Chemistry, Center for Excellence in Molecular Synthesis, SIOC, CAS, Shanghai, China.

[4]Department of Chemistry, Innovative Drug Research Center, Shanghai University, Shanghai, China.

[5]Shanghai-Hong Kong Joint Laboratory in Chemical Synthesis, SIOC, CAS, Shanghai, China.

Table of Contents

1.Supplementary Notes……………………………………………………..S2

2. Supplementary Methods ……………….……………………………..S2

3. Supplementary Tables…..…………..……………………………...S18

4. Supplementary Data - Analysis Data of Products…..…….………S19

5. Supplementary Figures - NMR and HPLC spectra…………………S38

6. Supplementary References………………………………….……S197

1.Supplementary Notes

Commercially available reagents were used without further purification. Solvents were purified prior to use according to the standard methods. Unless otherwise noted, all reactions were carried out under an atmosphere of argon and flame-dried glassware with standard vacuum-line techniques. NMR spectra are recorded at room temperature on 400 MHz Varian-400 or 400M Agilent-400 spectrometers. The chemical shifts for ^{1}H NMR are reported in ppm from tetramethylsilane (TMS) with the solvent resonance as the internal standard (7.26 ppm for $CHCl_3$). Data are reported as follows: chemical shift, multiplicity (s = singlet, d = doublet, t = triplet, q = quartet, sep = septet, bs = broad singlet, m = multiplet), coupling constants (Hz), and integration. Chemical shifts are reported in ppm from tetramethylsilane with the solvent resonance as the internal standard ($CDCl_3$: 77.15 ppm). MS and HRMS were measured in EI, ESI, or DART (direct analysis in real-time) mode and the mass analysis mode of the HRMS was TOF. Infrared spectra were recorded from thin films of pure samples and measured in cm^{-1}. Melting points were measured on an XT-4 micromelting point apparatus. Flash column chromatography was performed on silica gel. The hydrazones **1**,[1,2] ligands **L1**[3,4], **L2**,[5,6] were synthesized following the related references.

2. Supplementary Methods

2.1 Synthesis of chiral NHC ligands L3, L4, L5, L6 and L7.

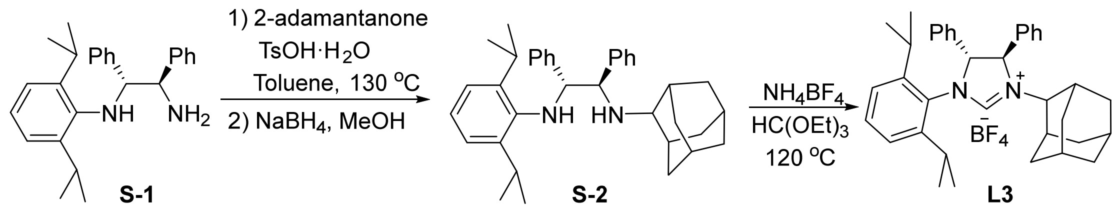

Supplementary Figure 1. Synthetic route **L3**

The **S-1**[5,6] (1.08 g, 2.91 mmol), 2-adamantanone (437 mg, 2.91 mmol), *p*-toluenesulfonic acid hydrate (5.8 mg, 0.029 mmol), and toluene (40 mL) were added into a 100 mL round-bottom flask. A Dean Stark apparatus was fitted and the mixture was refluxed for 9 h. After cooling down to room temperature, the solution was concentrated and methanol (50 mL) was added to the crude imine solution. $NaBH_4$ (450 mg, 11.8 mmol) was then slowly added in batches at 0 °C and stirred overnight. The solvent was removed under reduced pressure. The aqueous phase is then further extracted with Et_2O (3*40 mL). The combined organic phases are washed with brine (30 mL), then dried over anhydrous Na_2SO_4 and filtered. The filtrate is concentrated. The residue was purified by flash column chromatography (PE/EA = 50/1) to provide the product **S-2** as yellow oil (1.22 g, 83%).

The diamine **S-2** (1.22 g, 2.4 mmol) and NH_4BF_4 (250 mg, 2.4 mmol) were added into a 5 mL round-bottomed tube. $HC(OEt)_3$ (1.5 mL) was then added. The solution was stirred for 9 h at 120 °C. After cooling down to room temperature, the solution was concentrated. The residue was purified by flash column chromatography (DCM/CH_3OH = 10/1) to provide the product **L3** as white solid (300 mg, 55%). mp: 123.6-124.1 °C; $[\alpha]_D^{20}$ = 295.8 (1.8, $CHCl_3$); ^1H NMR (400 MHz, $CDCl_3$) δ 8.50 (s, 1H), 7.55 (d, *J* = 4.2 Hz, 4H), 7.51 – 7.45 (m, 1H), 7.39 (d, *J* = 6.9 Hz, 3H), 7.32 (t, *J* = 7.8 Hz, 1H), 7.26 (s, 1H), 7.21 (d, *J* = 8.1 Hz, 3H), 6.95 (d, *J* = 7.6 Hz, 1H), 5.66 (d, *J* = 6.1 Hz, 1H), 5.09 (d, *J* = 6.0 Hz, 1H), 4.11 (s, 1H), 3.16 – 2.97 (m, 1H), 2.83 (s, 1H), 2.58 – 2.34 (m, 1H), 2.12 (s, 2H), 1.97 (d, *J* = 15.0 Hz, 4H), 1.86 (s, 3H), 1.78 (d, *J* = 13.2 Hz, 3H), 1.57 (d, *J* = 13.8 Hz, 2H), 1.41 (d, *J* = 6.5 Hz, 3H), 1.17 (d, *J* = 6.9 Hz, 3H), 1.11 (d, *J* = 6.8 Hz, 3H), 0.40 (d, *J* = 6.6 Hz, 3H); ^{13}C NMR (126 MHz, $CDCl_3$) δ 157.32, 146.78, 146.23, 135.88, 133.75, 130.94, 130.40, 130.30, 130.13, 129.70, 128.45, 127.86, 126.04, 125.39, 124.36, 77.24, 69.90, 60.97, 36.85, 36.09, 36.08, 32.67, 30.82, 29.39, 28.93, 28.75, 28.56, 26.89, 26.44, 25.65, 24.46, 24.34, 22.82. IR (film): v 3064, 2919, 2857, 1724, 1625, 1453, 1259, 1224, 1051, 811, 762, 700, 636; ESI-MS *m/z* (rel): 517.3 $(M-BF_4)^+$; HRMS

Calcd. for $C_{37}H_{45}N_2$ $(M-BF_4)^+$: 517.3577; Found: 517.357.

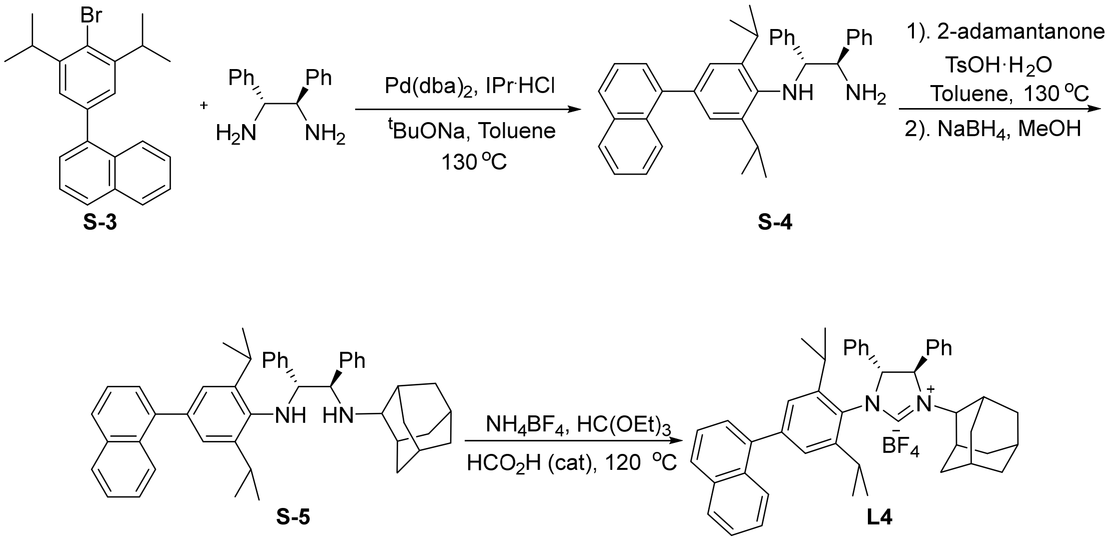

Supplementary Figure 2. Synthetic route **L4**

Pd(dba)2 (190 mg, 0.33 mmol), IPr·HCl (318 mg, 0.75 mmol), ${}^tBuONa$ (1.23 g, 12.7 mmol), (*R,R*)-1,2-diphenylethylenediamine (700 mg, 3.3 mmol), **S-3**[S5,S6] **(**1.22 g, 3.3 mmol) and toluene (10 mL) were added into 30 mL round-bottom flask. the resulting mixture was heated to 130 ℃ and stirred for 18 h. After cooling to rt, Et_2O (30 mL) was added. and then filtered through a 0.5 inch plug of silica gel (eluting with EtOAc) to remove the solid. The filtrate is concentrated. The residue was purified by flash column chromatography (DCM/CH_3OH = 10/1) to provide the product **S-4** as yellow oil (1.13 g, 69%). ^1H NMR (400 MHz, $CDCl_3$) δ 7.92 (dd, *J* = 7.8, 4.4 Hz, 2H), 7.84 (d, *J* = 7.9 Hz, 1H), 7.55 – 7.43 (m, 5H), 7.36 (d, *J* = 7.5 Hz, 2H), 7.30 – 7.23 (m, 3H), 7.20 – 7.13 (m, 6H), 4.59 (d, *J* = 7.5 Hz, 1H), 4.37 (d, *J* = 7.6 Hz, 1H), 3.28 (dt, *J* = 13.5, 6.8 Hz, 2H), 1.27 (d, *J* = 6.8 Hz, 6H), 0.99 (d, *J* = 6.7 Hz, 6H). ^{13}C NMR (101 MHz, $CDCl_3$) δ 143.26, 141.39, 141.17, 140.80, 140.60, 134.45, 133.91, 131.81, 128.26, 128.17, 128.09, 128.01, 127.88, 127.79, 127.41, 127.20, 127.06, 127.00, 126.64, 126.33, 125.73, 125.56, 125.44, 70.40, 60.72, 27.74, 24.30, 23.98.

The **S-4** (1.13 g, 2.26 mmol), 2-adamantanone (375 mg, 2.5 mmol), *p*-toluenesulfonic acid hydrate (3.8 mg, 0.02 mmol), and toluene (30 mL) were

added into a 50 mL round-bottom flask. A Dean Stark apparatus was fitted and the mixture was refluxed for 9 h. After cooling down to room temperature, the solution was concentrated and methanol (15 mL) was added to the crude imine solution. $NaBH_4$ (284 mg, 7.5 mmol) was then slowly added in batches at 0 °C and stirred overnight. The solvent was removed under reduced pressure. The aqueous phase is then further extracted with Et_2O (3*20 mL). The combined organic phases are washed with brine (20 mL), then dried over anhydrous Na_2SO_4 and filtered. The filtrate is concentrated. The residue was purified by flash column chromatography (PE/EA = 30/1) to provide the product **S-5** as yellow oil (790 mg, 56%).

The diamine **S-5** (730 mg, 1.15 mmol) and NH_4BF_4 (120 mg, 1.15 mmol) were added into a 5 mL round-bottomed tube. $HC(OEt)_3$ (1.0 mL) and one drop of HCO_2H were then added. The solution was stirred for 9 h at 120 °C. After cooling down to room temperature, the solution was concentrated. The residue was purified by flash column chromatography (DCM/CH_3OH = 50/1) to provide the product **L4** as white solid (725 mg, 86%). mp: 168.6-169.3 °C; $[\alpha]_D^{20}$ = 185.4 (*c* 1.0, $CHCl_3$); ^1H NMR (400 MHz, $CDCl_3$) δ 8.56 (s, 1H), 7.88 (dd, *J* = 13.5, 8.1 Hz, 2H), 7.72 (s, 1H), 7.66 – 7.55 (m, 4H), 7.47 (s, 7H), 7.37 (d, *J* = 9.6 Hz, 2H), 7.30 (s, 2H), 7.10 (s, 1H), 5.76 (d, *J* = 5.8 Hz, 1H), 5.20 (d, *J* = 6.1 Hz, 1H), 4.14 (s, 1H), 3.30 – 3.14 (m, 1H), 2.88 (s, 1H), 2.69 – 2.51 (m, 1H), 2.18 (d, *J* = 18.9 Hz, 2H), 2.01 (s, 4H), 1.91 (s, 3H), 1.82 (s, 3H), 1.59 (d, *J* = 12.2 Hz, 1H), 1.48 (d, *J* = 6.1 Hz, 3H), 1.24 (d, *J* = 6.6 Hz, 3H), 1.18 (d, *J* = 6.5 Hz, 3H), 0.46 (d, *J* = 6.3 Hz, 3H). ^{13}C NMR (101 MHz, $CDCl_3$) δ 157.09, 146.65, 146.29, 143.17, 138.99, 135.82, 133.73, 133.54, 131.15, 130.58, 130.28, 130.11, 129.79, 128.47, 128.36, 128.19, 127.09, 127.04, 126.85, 126.43, 126.14, 126.09, 125.91, 125.47, 125.23, 77.22, 69.84, 61.03, 36.81, 36.17, 36.05, 32.53, 30.86, 29.42, 29.05, 28.88, 28.53, 26.82, 26.46, 25.63, 24.52, 24.29, 22.82. IR (film): v 3061, 2963, 2925, 2856, 1624, 1585, 1496, 1455, 1388, 1333, 1314, 1220, 1182, 1058, 889, 802, 756, 701. HRMS Calcd. for $C_{47}H_{51}N_2$ $(M-BF_4)^+$: 643.4046; Found: 643.4034.

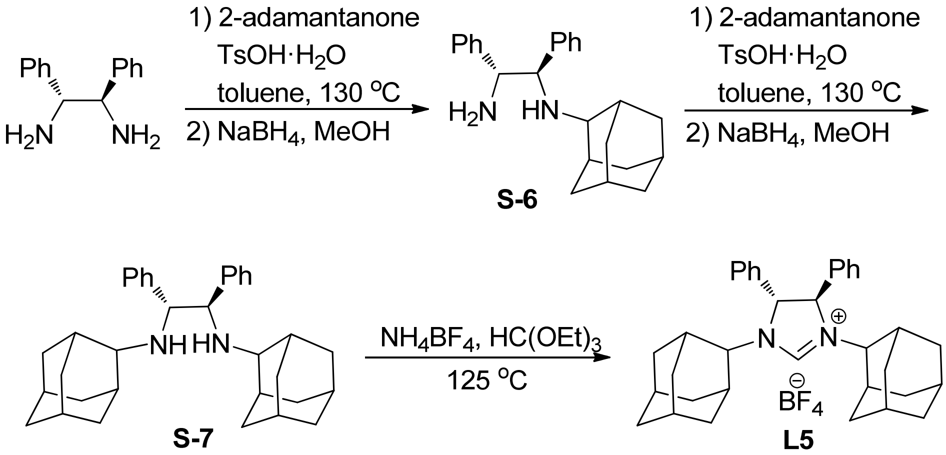

Supplementary Figure 3. Synthetic route **L5**

(*R,R*)-1,2-diphenylethylenediamine (1.06 g, 5.0 mmol), 2-adamantanone (796 mg, 5.3 mmol), *p*-toluenesulfonic acid hydrate (9.5 mg, 0.05 mmol), and toluene (60 mL) were added into a 100 mL round-bottom flask. A Dean Stark apparatus was fitted and the mixture was refluxed for 9 h. After cooling down to room temperature, the solution was concentrated and methanol (20 mL) was added to the crude imine solution. $NaBH_4$ (567 mg, 15.0 mmol) was then slowly added in batches at 0 °C and stirred overnight. The solvent was removed under reduced pressure. The aqueous phase is then further extracted with Et_2O (3*30 mL). The combined organic phases are washed with brine (30 mL), then dried over anhydrous Na_2SO_4 and filtered. The filtrate is concentrated. The residue was purified by flash column chromatography (DCM/CH_3OH = 50/1) to provide the product **S-6** as yellow oil (1.4 g, 82%).

The **S-6** (2.0 mmol, 0.7 g) was added into a 50 mL one-neck round bottom flask and the above procedure was repeated to provide the diamine **S-7**. The diamine **S-7** (0.84 mmol, 405 mg) and NH_4BF_4 (0.84 mmol, 88 mg) were added into a 5 mL round-bottomed tube. $HC(OEt)_3$ (2.0 mL) was then added. The solution was stirred for 9 h at 125 °C. After cooling down to room temperature, the solution was concentrated. The residue was purified by flash column chromatography (DCM/CH_3OH = 50/1) to provide the product **L5** as white solid (170 mg, 35%). mp: 122.6-123.1 °C; $[\alpha]_D^{20}$ = 178.7 (*c* 1.0, $CHCl_3$);

^{1}H NMR (400 MHz, $CDCl_3$) δ 8.60 (s, 1H), 7.50 (dq, J = 13.9, 6.8 Hz, 6H), 7.30 (d, J = 7.9 Hz, 4H), 4.88 (s, 2H), 3.72 (s, 2H), 2.61 (s, 2H), 1.96 (s, 8H), 1.80 (d, J = 15.1 Hz, 6H), 1.72 (d, J = 16.6 Hz, 6H), 1.59 (s, 4H), 1.49 (d, J = 12.7 Hz, 2H). ^{13}C NMR (101 MHz, $CDCl_3$) δ 156.05, 135.94, 130.23, 130.03, 125.83, 72.26, 61.23, 36.71, 36.30, 36.15, 31.28, 30.72, 29.61, 28.72, 26.68, 26.47. IR (film): ν 3061, 2905, 2854, 1620, 1494, 1470, 1452, 1316, 1281, 1219, 1049, 1026, 821, 758, 700, 630. ESI-MS *m/z*: 491.3 $(M\text{-}BF_4)^+$; HRMS Calcd. for $C_{35}H_{43}N_2$ $(M\text{-}BF_4)^+$: 491.3421; Found: 491.3412.

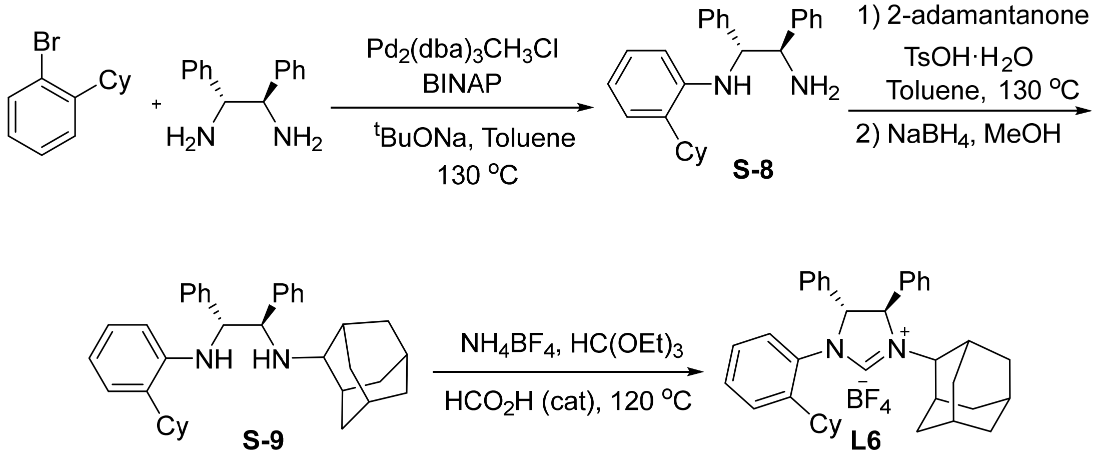

Supplementary Figure 4. Synthetic route **L6**

$Pd_2(dba)_3CH_3Cl$ (414 mg, 0.4 mmol), BINAP (620 mg, 0.96 mmol), ^{t}BuONa (2.32 g, 24 mmol), (*R,R*)-1,2-diphenylethylenediamine (1.7 g, 8.0 mmol), 1-bromo-2-cyclohexylbenzene (2.15 g, 9.0 mmol) and toluene (13 mL) were added into 30 mL round-bottom flask. the resulting mixture was heated to 130 ℃ and stirred for 10 h. After cooling to rt, then filtered through a 0.5 inch plug of silica gel (eluting with EtOAc) to remove the solid. The filtrate is concentrated. The residue was purified by flash column chromatography (DCM/CH_3OH = 50/1) to provide the product **S-8** (2.5 g, 84%) as yellow oil. ^{1}H NMR (400 MHz, $CDCl_3$) δ 7.45 (d, J = 7.4 Hz, 2H), 7.42 – 7.29 (m, 6H), 7.28 – 7.23 (m, 3H), 7.04 (d, J = 7.4 Hz, 1H), 6.81 (t, J = 7.6 Hz, 1H), 6.57 (t, J = 7.4 Hz, 1H), 6.12 (d, J = 8.1 Hz, 1H), 4.60 – 4.31 (m, 2H), 2.56 (t, J = 11.3 Hz, 1H),

2.04 – 1.84 (m, 3H), 1.74 (d, *J* = 13.1 Hz, 1H), 1.52 (t, *J* = 12.8 Hz, 2H), 1.42 – 1.20 (m, 4H).

The **S-8** (2.5 g, 6.7 mmol), 2-adamantanone (470 mg, 7.0 mmol), *p*-toluenesulfonic acid hydrate (9.5 mg, 0.05 mmol), and toluene (30 mL) were added into a 50 mL round-bottom flask. A Dean Stark apparatus was fitted and the mixture was refluxed for 9 h. After cooling down to room temperature, the solution was concentrated and methanol (15 mL) was added to the crude imine solution. $NaBH_4$ (227 mg, 6.0 mmol) was then slowly added in batches at 0 °C and stirred overnight. The solvent was removed under reduced pressure. The aqueous phase is then further extracted with Et_2O (3*20 mL). The combined organic phases are washed with brine (20 mL), then dried over anhydrous Na_2SO_4 and filtered. The filtrate is concentrated. The residue was purified by flash column chromatography (PE/EA = 30/1) to provide the product **S-9** as yellow oil (1.64 g, 54%). ^1H NMR (400 MHz, $CDCl_3$) δ 7.24 (dt, *J* = 6.7, 4.9 Hz, 3H), 7.21 – 7.09 (m, 8H), 6.84 (dd, *J* = 11.2, 4.2 Hz, 1H), 6.64 (t, *J* = 7.1 Hz, 1H), 6.21 (d, *J* = 7.6 Hz, 1H), 5.79 (s, 1H), 4.32 (dd, *J* = 7.0, 2.3 Hz, 1H), 3.97 (d, *J* = 6.9 Hz, 1H), 2.75 (t, *J* = 11.2 Hz, 1H), 2.53 (s, 1H), 2.20 (d, *J* = 12.8 Hz, 1H), 2.03 (d, *J* = 12.1 Hz, 1H), 1.95 (d, *J* = 13.1 Hz, 2H), 1.87 – 1.63 (m, 10H), 1.60 – 1.33 (m, 10H). ^{13}C NMR (101 MHz, $CDCl_3$) δ 144.44, 141.86, 141.23, 131.99, 128.20, 128.14, 127.67, 127.10, 126.92, 126.31, 125.18, 116.90, 111.81, 65.31, 64.44, 57.79, 38.35, 37.88, 37.61, 36.87, 34.33, 33.05, 32.75, 31.77, 31.04, 29.52, 27.70, 27.62, 27.38, 27.26, 26.57.

The diamine **S-9** (707 mg, 1.4 mmol) and NH_4BF_4 (146 mg, 1.4 mmol) were added into a 5 mL round-bottomed tube. $HC(OEt)_3$ (1.0 mL) and one drop of HCO_2H were then added. The solution was stirred for 9 h at 120 °C. After cooling down to room temperature, the solution was concentrated. The residue was purified by flash column chromatography (DCM/CH_3OH = 50/1) to provide the product **L6** as white solid (517 mg, 62%). mp: 141.3-142.1 °C; $[\alpha]_D^{20}$ = 263.9 (*c* 1.0, $CHCl_3$); ^1H NMR (400 MHz, $CDCl_3$) δ 8.44 (s, 1H), 7.56 (dt, *J* = 15.1, 7.4 Hz, 4H), 7.49 (d, *J* = 7.1 Hz, 1H), 7.41 – 7.33 (m, 3H), 7.21 (dt, *J* =

16.5, 7.4 Hz, 6H), 5.36 (d, J = 7.3 Hz, 1H), 5.25 (d, J = 7.3 Hz, 1H), 3.92 (s, 1H), 2.75 (s, 1H), 2.48 (s, 1H), 2.09 (s, 2H), 1.98 – 1.64 (m, 15H), 1.52 (s, 1H), 1.26 (m, 4H), 1.13 – 1.03 (m, 1H); ^{13}C NMR (101 MHz, $CDCl_3$) δ 156.88, 144.11, 135.39, 134.54, 131.25, 130.27, 130.14, 129.71, 127.91, 127.58, 127.34, 127.25, 126.79, 71.43, 60.77, 38.86, 36.78, 36.36, 35.96, 35.13, 33.51, 31.94, 31.21, 29.05, 28.38, 26.88, 26.84, 26.73, 26.44, 25.79. IR (film): v 3064, 2925, 2853, 1627, 1601, 1578, 1496, 1454, 1314, 1282, 1219, 1182, 1057, 963, 894, 760, 701. HRMS Calcd. for $C_{37}H_{43}N_2$ $(M\text{-}BF_4)^+$: 515.3420; Found: 515.3414.

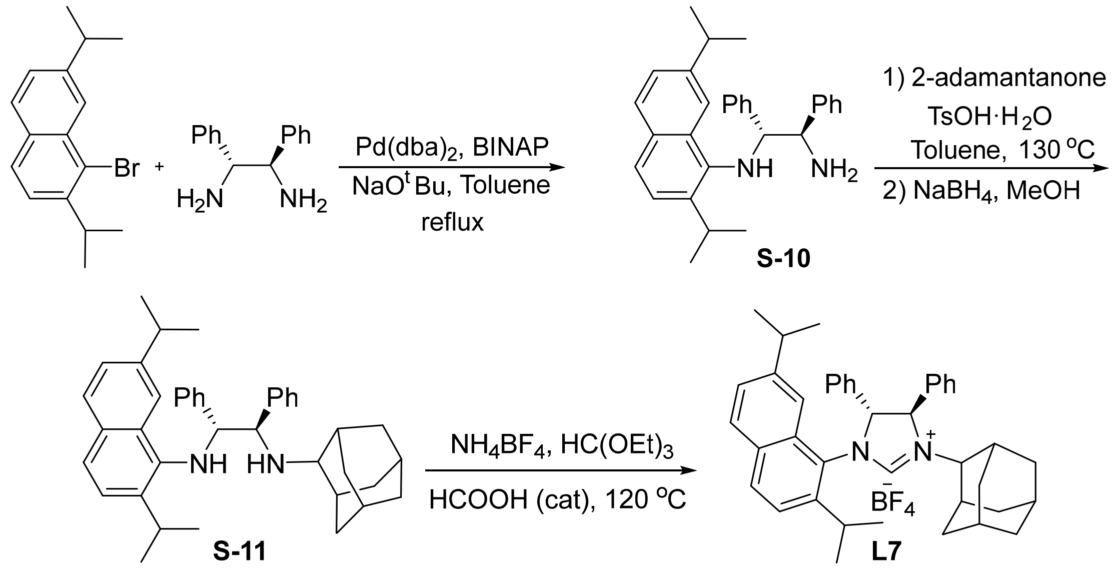

Supplementary Figure 5. Synthetic route **L7**

Pd(dba)$_2$ (345 mg, 0.6 mmol), BINAP (448 mg, 0.72 mmol), ^{t}BuONa (1.73 g, 18 mmol), (R,R)-1,2-diphenylethylenediamine (1.27 g, 6.0 mmol), were added into 30 mL round-bottom flask and reflush with Ar for 3 times, then toluene (5.0 mL) was added. After stirred for 5 min, 1-bromo-2,7-diisopropylnaphthalene (1.68 g, 5.0 mmol) and toluene (3.0 mL) were added. Then, the mixture was heated to 130 °C and stirred for 12 h. After cooling to rt, then filtered through a 0.5 inch plug of silica gel (eluting with EtOAc) to remove the solid. The filtrate is concentrated. The residue was purified by flash column chromatography (DCM/CH_3OH = 50/1) to provide the product **S-10** (1.45 g, 58%) as yellow solid. ^{1}H NMR (400 MHz, $CDCl_3$) δ 8.34

(s, 1H), 7.69 (d, *J* = 8.3 Hz, 1H), 7.41 (d, *J* = 8.5 Hz, 1H), 7.33 (dd, *J* = 13.3, 8.1 Hz, 3H), 7.20 (t, *J* = 7.3 Hz, 2H), 7.14 (d, *J* = 8.3 Hz, 2H), 7.05 (dd, *J* = 24.0, 5.6 Hz, 6H), 4.65 (d, *J* = 9.5 Hz, 2H), 3.06 (dt, *J* = 13.5, 6.7 Hz, 2H), 2.71 (s, 2H), 1.38 (dd, *J* = 9.5, 7.1 Hz, 6H), 1.16 (d, *J* = 6.7 Hz, 3H), 0.76 (d, *J* = 6.6 Hz, 3H). ^{13}C NMR (101 MHz, $CDCl_3$) δ 145.58, 143.30, 141.65, 138.72, 135.90, 131.80, 128.69, 128.11, 128.08, 127.95, 127.72, 127.47, 127.05, 126.94, 124.74, 123.08, 122.62, 120.85, 69.78, 60.95, 34.70, 27.58, 24.33, 24.23, 23.98, 23.08.

The **S-10** (845 mg, 2.0 mmol), 2-adamantanone (316 mg, 2.1 mmol), *p*-toluenesulfonic acid hydrate (3.8 mg, 0.02 mmol), and toluene (40 mL) were added into a 100 mL round-bottom flask. A Dean Stark apparatus was fitted and the mixture was refluxed for 7 h. After cooling down to room temperature, the solution was concentrated and methanol (50 mL) was added to the crude imine solution. $NaBH_4$ (227 mg, 6.0 mmol) was then slowly added in batches at 0 ^{o}C and stirred overnight. The solvent was removed under reduced pressure. The aqueous phase is then further extracted with Et_2O (3*20 mL). The combined organic phases are washed with brine (20 mL), then dried over anhydrous Na_2SO_4 and filtered. The filtrate is concentrated. The residue was purified by flash column chromatography (PE/EA = 50/1) to provide the product **S-11** as yellow solid (690 mg, 62%). ^{1}H NMR (400 MHz, $CDCl_3$) δ 8.65 (s, 1H), 7.68 (d, *J* = 8.5 Hz, 1H), 7.42 (d, *J* = 8.6 Hz, 1H), 7.33 (d, *J* = 8.1 Hz, 1H), 7.25 (d, *J* = 7.2 Hz, 2H), 7.16 – 7.05 (m, 4H), 6.99 (s, 3H), 6.90 (d, *J* = 3.7 Hz, 2H), 4.50 (d, *J* = 9.1 Hz, 1H), 4.39 (d, *J* = 9.1 Hz, 1H), 4.29 (s, 1H), 3.42 (s, 1H), 3.15 (dt, *J* = 13.9, 6.9 Hz, 1H), 3.07 (dd, *J* = 13.4, 6.7 Hz, 1H), 2.68 (s, 1H), 2.18 (d, *J* = 14.0 Hz, 3H), 1.91 – 1.69 (m, 7H), 1.58 (dd, *J* = 36.0, 10.6 Hz, 4H), 1.47 (d, *J* = 6.9 Hz, 3H), 1.42 (d, *J* = 6.9 Hz, 3H), 1.13 (d, *J* = 6.8 Hz, 3H), 0.60 (d, *J* = 6.5 Hz, 3H). ^{13}C NMR (101 MHz, $CDCl_3$) δ 145.89, 142.40, 141.25, 138.47, 136.82, 131.82, 129.09, 128.28, 128.10, 128.06, 127.71, 126.82, 126.62, 124.73, 123.10, 121.13, 70.14, 64.81, 58.72, 38.03, 37.94, 37.41,

34.88, 34.16, 31.90, 31.64, 30.12, 27.85, 27.71, 27.54, 24.42, 24.31, 24.11, 22.86.

The diamine **S-11** (600 mg, 1.07 mmol) and NH_4BF_4 (113 mg, 1.07 mmol) were added into a 5 mL round-bottomed tube. $HC(OEt)_3$ (1.0 mL) and one drop of HCO_2H were then added. The solution was stirred for 9 h at 120 °C. After cooling down to room temperature, the solid was filtered and washed with ether to obtain the product **L7** as white solid (250 mg, 35%). A minor atropisomer was observed. mp: 162.3-162.9 °C; $[\alpha]_D^{20}$ = 201.2 (*c* 1.0, $CHCl_3$); ^1H NMR (400 MHz, $CDCl_3$) (taken as mixture of diastereoisomers) (major diastereoisomer) δ 8.51 (s, 1H), 7.79 – 7.71 (m, 4H), 7.61 (t, *J* = 7.5 Hz, 2H), 7.56 – 7.50 (m, 1H), 7.43 (d, *J* = 8.7 Hz, 1H), 7.37 – 7.22 (m, 4H), 7.13 (d, *J* = 8.7 Hz, 1H), 7.03 (d, *J* = 7.3 Hz, 2H), 5.80 (d, *J* = 6.8 Hz, 1H), 5.58 (d, *J* = 6.9 Hz, 1H), 4.17 (s, 1H), 3.04 (dt, *J* = 13.7, 6.8 Hz, 1H), 2.79 (s, 1H), 2.73 (dd, *J* = 13.6, 6.8 Hz, 1H), 2.22 (s, 1H), 2.13 (s, 1H), 1.98 (s, 4H), 1.89 (s, 3H), 1.81 (s, 3H), 1.60 (d, *J* = 13.4 Hz, 1H), 1.31 (d, *J* = 6.9 Hz, 3H), 1.27 (d, *J* = 6.9 Hz, 3H), 1.19 (d, *J* = 6.9 Hz, 3H), 0.50 (d, *J* = 6.6 Hz, 3H). ^{13}C NMR (101 MHz, $CDCl_3$) δ 157.20, 149.38, 144.44, 135.97, 133.47, 131.35, 130.87, 130.45, 130.30, 130.09, 129.56, 129.22, 129.09, 128.59, 128.35, 126.49, 124.74, 122.33, 118.58, 75.71, 69.78, 61.10, 36.82, 36.16, 36.07, 34.55, 32.54, 30.83, 29.35, 29.04, 28.55, 26.89, 26.45, 25.08, 23.88, 23.83, 22.55. IR (film): ν 2959, 2914, 2853, 1622, 1459, 1314, 1282, 1203, 1072, 1051, 1030, 964, 844, 700. HRMS Calcd. for $C_{41}H_{47}N_2$ $(M-BF_4)^+$: 567.3733; Found: 567.3738.

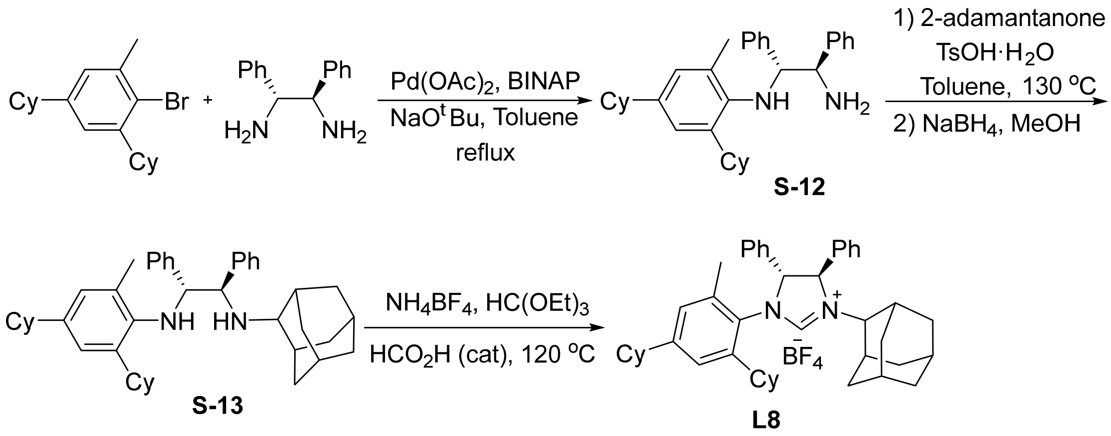

Supplementary Figure 6. Synthetic route **L8**

$Pd(OAc)_2$ (113 mg, 0.5 mmol)，BINAP (623 mg, 1.0 mmol)，^tBuONa (1.45 g, 15 mmol)，(*R,R*)-1,2-diphenylethylenediamine (1.06 g, 5.0 mmol)，2-bromo-1,5-dicyclohexyl-1-methylbenzene[3,4] (1.68 g, 5.0 mmol) and toluene (10 mL) were added into 30 mL round-bottom flask. the resulting mixture was heated to 130 ℃ and stirred for 12 h. After cooling to rt, then filtered through a 0.5 inch plug of silica gel (eluting with EtOAc) to remove the solid. The filtrate is concentrated. The residue was purified by flash column chromatography (DCM/CH_3OH = 50/1) to provide the product **S-12** (1.25 g, 54%) as yellow oil. ^1H NMR (400 MHz, $CDCl_3$) δ 7.29 (d, *J* = 7.3 Hz, 3H), 7.19 (t, *J* = 7.3 Hz, 2H), 7.14 – 7.08 (m, 3H), 7.03 (d, *J* = 6.6 Hz, 2H), 6.71 (d, *J* = 19.9 Hz, 2H), 4.47 (d, *J* = 7.2 Hz, 1H), 4.31 (d, *J* = 7.4 Hz, 1H), 2.63(m, 1H), 2.31(m, 1H), 2.19 (s, 3H), 1.87 – 1.64 (m, 10H), 1.19 – 1.35 (m, 10H). ^{13}C NMR (101 MHz, $CDCl_3$) δ 143.18, 141.94, 140.91, 140.50, 138.48, 128.85, 128.05, 127.91, 127.63, 127.44, 126.99, 126.86, 126.79, 122.99, 68.42, 60.95, 43.91, 38.18, 34.67, 34.57, 33.90, 27.27, 27.07, 27.00, 26.32, 26.24, 20.07.

The **S-12** (1.2 g, 2.67 mmol), 2-adamantanone (450 mg, 3.0 mmol), *p*-toluenesulfonic acid hydrate (5.7 mg, 0.03 mmol), and toluene (30 mL) were added into a 50 mL round-bottom flask. A Dean Stark apparatus was fitted and the mixture was refluxed for 9 h. After cooling down to room temperature, the solution was concentrated and methanol (15 mL) was added to the crude imine solution. $NaBH_4$ (227 mg, 6.0 mmol) was then slowly added in batches at 0 °C and stirred overnight. The solvent was removed under reduced pressure.

The aqueous phase is then further extracted with Et_2O (3*20 mL). The combined organic phases are washed with brine (20 mL), then dried over anhydrous Na_2SO_4 and filtered. The filtrate is concentrated. The residue was purified by flash column chromatography (PE/EA = 30/1) to provide the product **S-13** as yellow oil (500 mg, 32%). ^1H NMR (400 MHz, $CDCl_3$) δ 7.27 – 7.21 (m, 3H), 7.13 (t, *J* = 7.2 Hz, 3H), 7.09 (d, *J* = 9.1 Hz, 2H), 6.98 (d, *J* = 5.9 Hz, 2H), 6.80 (s, 1H), 6.66 (s, 1H), 4.24 (d, *J* = 8.2 Hz, 1H), 4.19 (d, *J* = 8.3 Hz, 1H), 2.10 (s, 3H), 1.91 – 1.66 (m, 20H), 1.63 – 1.45 (m, 7H), 1.40 – 1.26 (m, 10H). ^{13}C NMR (101 MHz, $CDCl_3$) δ 142.39, 141.85, 141.18, 140.44, 139.22, 129.25, 128.25, 127.89, 127.75, 127.68, 126.76, 126.72, 126.56, 123.36, 69.06, 64.78, 58.41, 43.94, 37.99, 37.88, 37.31, 34.92, 34.59, 34.56, 34.13, 31.82, 31.37, 29.97, 27.81, 27.64, 27.24, 27.05, 27.00, 26.44, 26.24, 19.97. IR (film): v 3060, 3026, 2920, 2848, 1491, 1474, 1447, 1351, 1259, 1235, 1211, 1131, 1071, 1026, 962, 860, 758, 698. ESI-MS *m/z* (rel): 601.4 $(M+H)^+$; HRMS (ESI) Calcd. for $C_{43}H_{57}N_2$ $(M+H)^+$: 601.4516; Found: 601.4496.

The diamine **S-13** (400 mg, 0.66 mmol) and NH_4BF_4 (69 mg, 0.66 mmol) were added into a 5 mL round-bottomed tube. $HC(OEt)_3$ (1.0 mL) and one drop of HCO_2H were then added. The solution was stirred for 9 h at 120 °C. After cooling down to room temperature, the solution was concentrated. The residue was purified by flash column chromatography (DCM/CH_3OH = 50/1) to provide the product **L8** as white solid (295 mg, 64%). A minor atropisomer was observed. mp: 159.9-160.5 ℃; $[\alpha]_D^{20}$ = 241.2 (*c* 1.0, $CHCl_3$); ^1H NMR (400 MHz, $CDCl_3$) (taken as mixture of diastereoisomers) (major diastereoisomer) δ 8.40 (d, *J* = 8.9 Hz, 1H), 8.18 (s, 1H), 7.58 (d, *J* = 6.4 Hz, 3H), 7.37 (d, *J* = 5.1 Hz, 3H), 7.19 (d, *J* = 5.5 Hz, 3H), 6.91 (s, 1H), 6.74 (s, 1H), 5.71 (d, *J* = 7.4 Hz, 1H), 5.27 (d, *J* = 7.5 Hz, 1H), 3.98 (s, 1H), 2.79 (s, 1H), 2.47 (s, 3H), 2.37 (s, 1H), 2.18 (s, 1H), 1.96 (d, *J* = 17.1 Hz, 6H), 1.79 (s, 12H), 1.69 (s, 2H), 1.38 – 1.14 (m, 13H). ^{13}C NMR (101 MHz, $CDCl_3$) (major diastereoisomer) δ 156.33, 150.05, 144.91, 135.65, 130.27, 130.21, 129.93, 129.52, 128.44, 128.28, 127.91, 127.13, 126.63, 126.27, 123.66, 74.78, 69.51, 60.53, 44.21, 39.29,

36.77, 36.31, 35.99, 35.91, 34.10, 34.00, 32.82, 32.25, 31.13, 28.97, 28.29, 26.94, 26.84, 26.76, 26.69, 26.47, 25.99, 25.70, 18.78. IR (film): v 3063, 2925, 2851, 1628, 1585, 1496, 1451, 1314, 1280, 1261, 1220, 1182, 1057, 958, 861, 759, 700. HRMS Calcd. for $C_{44}H_{55}N_2$ $(M-BF_4)^+$: 611.4359; Found: 611.4356.

2.2 General Procedure for *gem*-difluoroallylation products (3)

A dry Schlenk tube was flame dried and flushed with Argon. Hydrazone **1** (0.4 mmol) and toluene (2.0 mL) were added into the dry Schlenk tube. LDA (1.0 M in THF, 0.4 mL, 0.4 mmol) were added at 0 °C and stirred at room temperature for 30 min. In a separated flask, $[Pd(C_3H_5)Cl]_2$ (1.83 mg, 0.005 mmol), **L8** (7.1 mg, 0.01 mmol) and toluene (1.0 mL) were mixed, followed by addition of *t*-BuOK (1.0 M in THF, 25 uL, 0.025 mmol) at rt. The resulting mixture was stirred at room temperature for 30 min, then added to the hydrazone solution. The BDFP **2** (31.5 mg, 0.2 mmol) and toluene (1.0 mL) was then added and the mixture was stirred at room temperature. After the reaction was completed, the reaction mixture was quenched by H_2O (0.3 mL). Trifluorotoluene (24 μL) was added as an internal standard. The regio- and diastereoselectivity was then determined by ^{19}F NMR spectroscopy. After this analysis, the crude reaction mixture was dried (anhydrous Na_2SO_4) and then filtered through a 0.5 inch plug of silica gel (eluting with EtOAc) to remove the solid. The crude reaction mixture was concentrated under reduced pressure and then purified by preparative TLC (petroleum ether/ethyl acetate 50/1) to afford the product.

2.3 Conversion of the Obtained Products

Synthesis of 6:

To a mixture of **3a** (0.35 mmol, 101 mg) and THF (1.0 mL) in a 20 mL tube, 9-BBN (4.0 mL, 2.0 mmol, 0.5 M in THF) was slowly injected via a syringe into the reaction tube at 0 ℃. Then the reaction mixture was allowed to warm

slowly to 50 ℃ and stirred for 18 h. After the reaction was completed, the reaction mixture was room temperature. Then 33% NaOH solution (0.4 mL) and 35% H_2O_2 (0.5 mL) were added. The resulting mixture was stirred for 4 h at 40 ℃ The organic layer was extracted with ethyl acetate, washed with water, and dried over anhydrous sodium sulfate. The volatiles were removed under reduced pressure to yield crude compound. The crude compound was purified by preparative TLC (petroleum ether/ ethyl acetate = 10:1) to give the product **6**. Yield: 66%; er: 95:5.

Synthesis of 7:

6 (0.23 mmol, 70 mg) and Pd/C (20 mg) were dissolved in MeOH (5.0 mL) under H_2 (15 atm) atmosphere. Then the mixture was stirred at 65 ℃ for 25 h. After the reaction was completed, the reaction mixture was filtered and the filtrate was concentrated under vacuum condition. The residue was purified by preparative TLC (petroleum ether/ethyl acetate 10:1) and directly to give the product **7**. The single crystal was obtained by recrystallization from $CHCl_3$. Yield: 78%; er: 95:5.

Synthesis of 8:

Following Hartwig's method,[7] a modified procedure was applied. In a nitrogen-filled tube, CuCl (0.04 mmol, 4.0 mg), PCy_3 (0.04 mmol, 12.0 mg) and THF (0.4 mL) were added. The solution was allowed to stir at room temperature for 20 minutes, at which time neat NaO^tBu (0.08 mmol, 8.0 mg) was added, and the subsequent solution was allowed to stir at room temperature for 1 hour. Separately, a 4 mL schlenck tube equipped with a stir bar was charged with **3a** (0.2 mmol, 58.0 mg) and the newly prepared catalyst solution. The resulting mixture was allowed to stir for 10 seconds, after which time neat bis(pinacolato)diboron (0.4 mmol, 101.6 mg) was added. The reaction tube was then heated at 45 °C with stirring for 8 hours. After cooling down to room temperature, the solution was diluted with 1.0 mL THF. The resulting solution was cooled to 0 °C with an ice bath, and aqueous 3 M NaOH (1.2 mL) was added dropwise, followed by immediate dropwise addition of

aqueous hydrogen peroxide with a plastic syringe (1.0 mL, 34% by mass in H_2O). The reaction mixture was stirred in an ice bath, where it was allowed to slowly warm to room temperature overnight. After this time, the reaction mixture was quenched by dropwise addition of 2 M sodium thiosulfate. The organic layer was extracted with dichloromethane (3 x 20 mL), and dried over anhydrous sodium sulfate. The volatiles were removed under reduced pressure to yield crude compound. The crude compound was purified by preparative TLC (petroleum ether/ ethyl acetate = 8:1) to give the product **8**. Yiled: 81%; er: 94:6.

Synthesis of 9:

To a 25 mL of Schlenk tube equipped with a magnetic stir bar were added $Pd(OAc)_2$ (3.4 mg, 10 mol%), NaOAc (18 mg, 1.5 equiv) and NBu_4Br (72 mg, 1.5 equiv) in a glove box. The tube was then taken out from the glove box. Substrate **3a** (43 mg, 0.15 mmol, 1.0 equiv), ethyl 4-iodobenzoate (83 mg, 0.3 mmol, 2 equiv) and anhydrous DMF(2ml) were added subsequently under Ar. After stirring for 12 h at 110 ℃, the reaction mixture was cooled to room temperature, and then sat. NH_4Cl aq. was poured into the reaction mixture and extracted with ethyl acetate. The organic layer was washed with water, dried over Na_2SO_4, and filtered. The filtrate was concentrated in vacuo and the residue was purified by silica gel column chromatography (eluent: PE:DCM= 5:1) to afford product **9**. Yiled: 65%; er: 95:5.

2.4 Control experiments

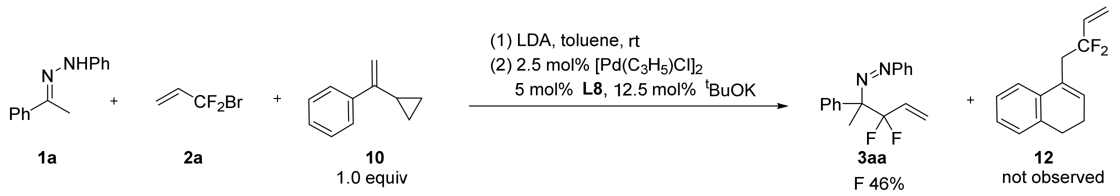

A dry Schlenk tube was flame dried and flushed with Argon. Hydrazone **1a** (84.0 mg, 0.4 mmol) and toluene (2.0 mL) were added into the dry Schlenk tube. LDA (1.0 M in THF, 0.4 mL, 0.4 mmol) were added at 0 °C and stirred at

room temperature for 30 min. In a separated flask, $[Pd(C_3H_5)Cl]_2$ (1.83 mg, 0.005 mmol), **L8** (7.1 mg, 0.01 mmol) and toluene (1.0 mL) were mixed, followed by addition of *t*-BuOK (1.0 M in THF, 25 uL, 0.025 mmol) at rt. The resulting mixture was stirred at room temperature for 30 min, then added to the hydrazone solution. The BDFP **2a** (31.5 mg, 0.2 mmol), **10** and toluene (1.0 mL) was then added subsequently. The mixture was stirred at room temperature. After the reaction was completed, the reaction mixture was quenched by H_2O (0.3 mL). Fluorobenzene (40 μL) was added as an internal standard.

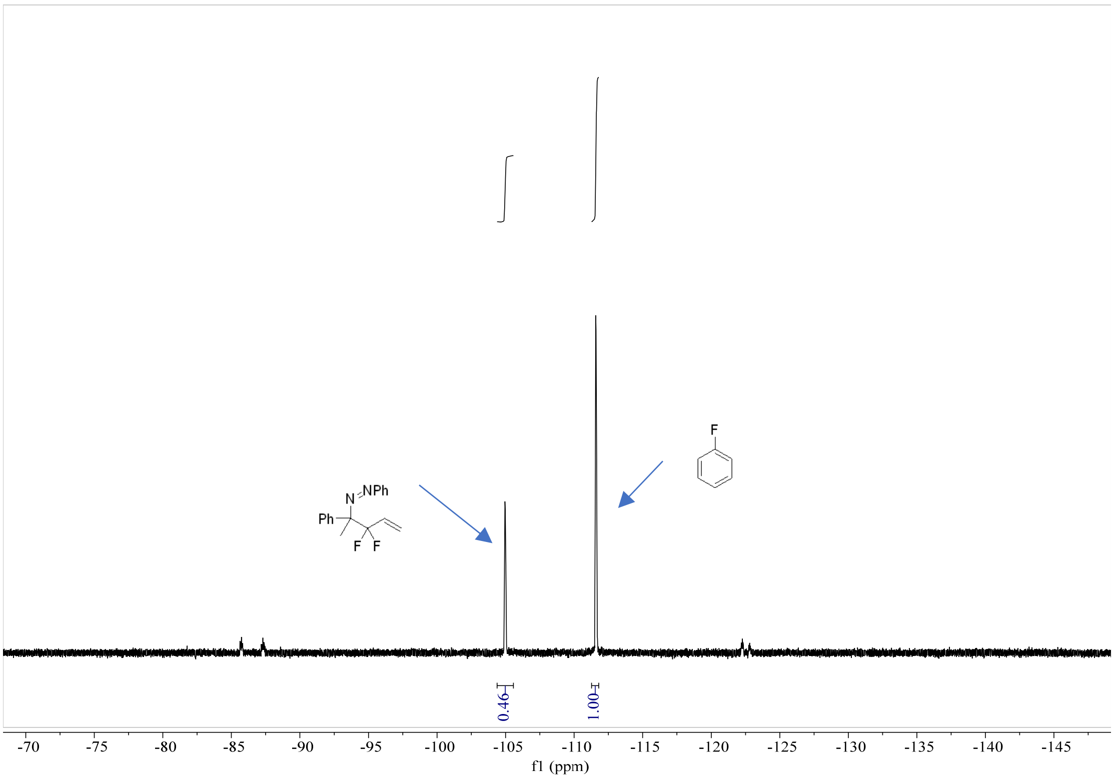

Supplementary Figure 7. ^{19}F- NMR Spectra of crude products in control experiments with **10**

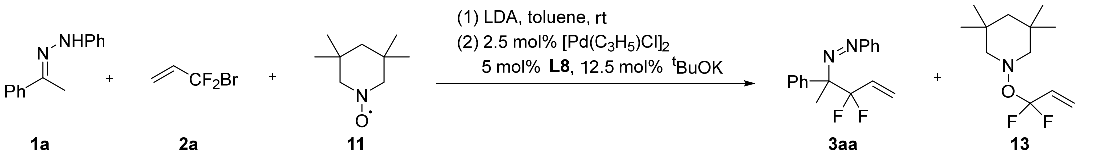

A dry Schlenk tube was flame dried and flushed with Argon. Hydrazone **1a** (84.0 mg, 0.4 mmol) and toluene (2.0 mL) were added into the dry Schlenk tube. LDA (1.0 M in THF,

0.4 mL, 0.4 mmol) were added at 0 °C and stirred at room temperature for 30 min. In a separated flask, $[Pd(C_3H_5)Cl]_2$ (1.83 mg, 0.005 mmol), **L8** (7.1 mg, 0.01 mmol) and toluene (1.0 mL) were mixed, followed by addition of *t*-BuOK (1.0 M in THF, 25 uL, 0.025 mmol) at rt. The resulting mixture was stirred at room temperature for 30 min, then added to the hydrazone solution. The BDFP **2a** (31.5 mg, 0.2 mmol), TEMPO and toluene (1.0 mL) was then added subsequently. The mixture was stirred at room temperature. After the reaction was completed, the reaction mixture was quenched by H_2O (0.3 mL). Fluorobenzene (40 μL) was added as an internal standard.

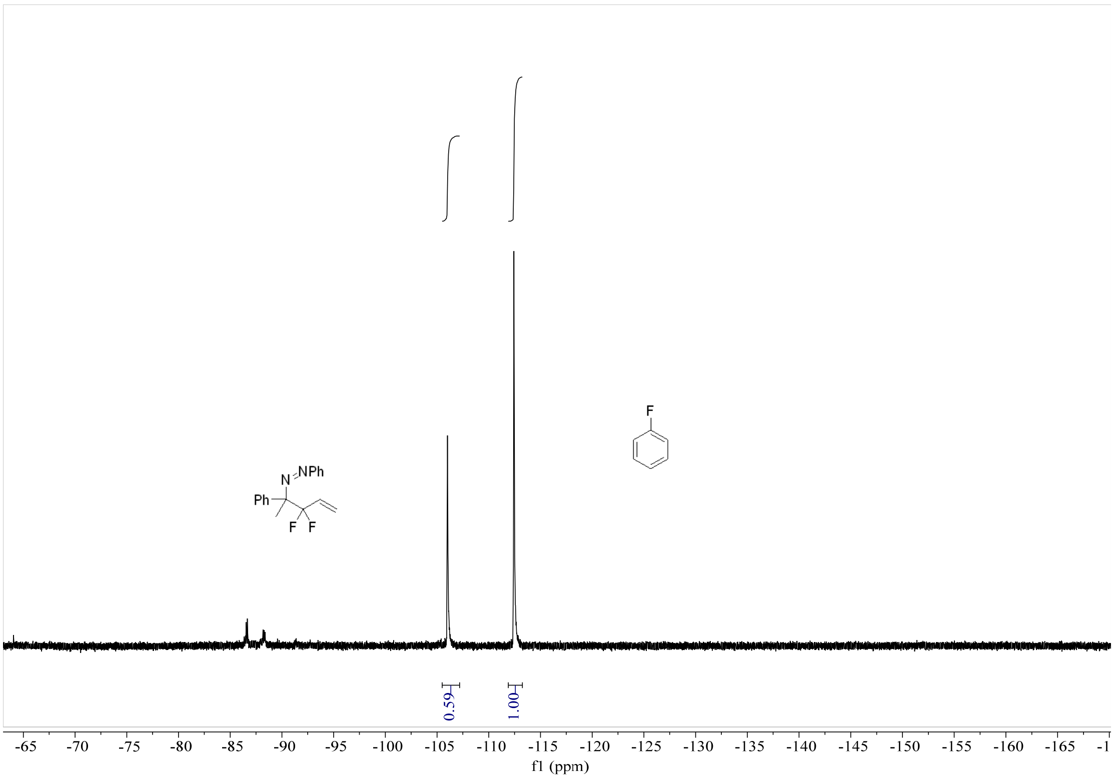

Supplementary Figure 8. ^{19}F- NMR Spectra of crude products in control experiments with **11**

3.Supplementary Tables

Supplementary Table 1. Effects of ligands on the Pd-Catalyzed reaction of **1a** with **2**[a]

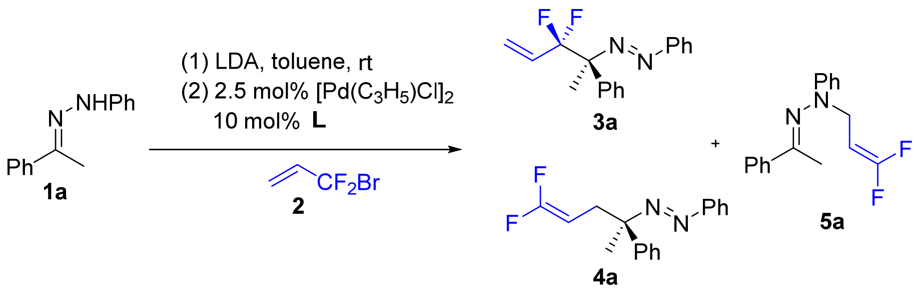

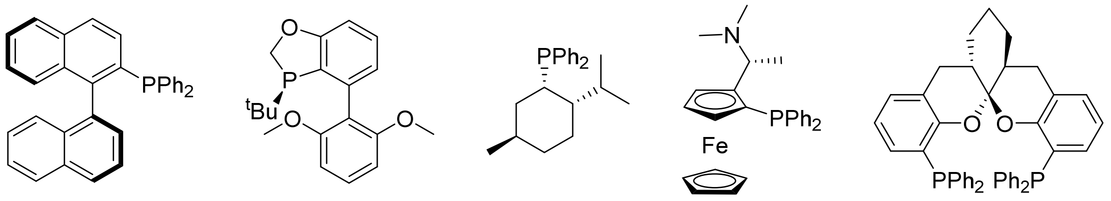

Entry	Ligand	**3a/4a/5a**[b]	Yield[b]/%	Ee[c]/% (**3a**)	Ee[c]/% (**4a**)
1	**PPh_3**	8/92/0	99	--	--
2	**$P(4\text{-}MeOC_6H_5)_3$**	10/90/0	99	--	--
3	**$P(2\text{-}MeC_6H_5)_3$**	23/0/77	60	--	--
4	**$P(2\text{-furyl})_3$**	6/94/0	99	--	--
5	**(*R*)-MOP**	54/46/0	99	10	8
6	**L9**	19/67/14	95	nd	40
7	**L10**	30/70/0	99	37	14
8	**L11**	0/0/100	99	--	--
9	**L12**	0/55/45	91	--	40

[a]Reaction conditions: **1a**/LDA/**2a**/$[Pd(C_3H_5)Cl]_2$/**L** = 200/200/100/2.5/10; 0.05 M of **2**. [b]Determined by ^{19}F NMR of crude, benzotrifluoride as interal standard. [c]Determined by Chiral HPLC.

4. Supplementary Data - Analysis Data of Products

4.1 Analysis data for products

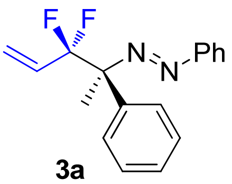

3a

Yellow oil; Yield: 90%; er: 95:5; $[\alpha]_D^{29}$ = -55.8 (*c* 1.0, $CHCl_3$); ^{1}H NMR (400 MHz, $CDCl_3$) δ 7.90 – 7.74 (m, 2H), 7.49 (dd, *J* = 15.1, 7.2 Hz, 5H), 7.37 – 7.28 (m, 3H), 6.12 (dq, *J* = 17.3, 11.8 Hz, 1H), 5.62 (d, *J* = 17.4 Hz, 1H), 5.46 (d, *J* = 11.1 Hz, 1H), 1.77 (s, 3H). ^{13}C NMR (101 MHz, $CDCl_3$) δ 157.33, 145.60, 136.54, 136.23 (t, *J* = 25.7 Hz), 134.43, 133.59, 133.29, 133.05, 127.95, 126.32 (t, *J* = 9.5 Hz), 126.18(t, *J* = 248.9 Hz), 84.16 (t, *J* = 23.9 Hz), 23.19. ^{19}F NMR (376 MHz, $CDCl_3$) δ -106.14 (dd, *J* = 245.5, 12.2 Hz), -106.87 (dd, *J* = 245.1, 12.0 Hz). IR (film): v 3061, 3000, 1599, 1525, 1495, 1450, 1373, 1208, 1093, 1038, 993, 924, 762, 687. ESI-MS *m/z* (rel): 287.1 $(M+H)^+$; HRMS (ESI) Calcd. for $C_{17}H_{17}F_2N_2$ $(M+H)^+$: 287.1354; Found: 287.1354. HPLC (PC-3 (Phenomenex Cellulose-3), CH_3CN : H_2O = 62 : 38, 0.7 mL/min, 214 nm): t_{minor} = 16.72 min, t_{major} = 18.52 min.

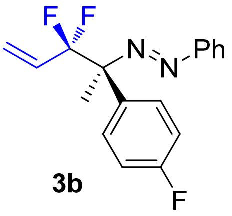

3b

Yellow oil; Yield: 78%; er: 95:5; $[\alpha]_D^{20}$ = -115.38 (*c* 1.0, $CHCl_3$); ^{1}H NMR (400 MHz, $CDCl_3$) δ 7.92 (dd, *J* = 5.5, 1.9 Hz, 2H), 7.64 (dd, *J* = 8.4, 2.8 Hz, 3H), 7.61 – 7.52 (m, 2H), 7.15 (dd, *J* = 12.2, 5.0 Hz, 2H), 6.22 (dq, *J* = 17.4, 12.0 Hz, 1H), 5.74 (d, *J* = 17.4 Hz, 1H), 5.60 (d, *J* = 11.1 Hz, 1H), 1.87 (s, 3H). ^{13}C NMR (101 MHz, $CDCl_3$) δ 163.53, 161.08, 151.98, 135.97, 131.46, 130.76 (t, *J* = 25.5 Hz), 130.25, 130.18, 129.23, 122.72, 121.31 (t, *J* = 9.6 Hz), 120.81 (t, *J* = 248.7 Hz), 114.92 (d, *J* = 21.2 Hz), 78.48 (t, *J* = 24.0 Hz), 18.05. ^{19}F NMR (376 MHz, $CDCl_3$) δ -106.28 (dd, *J* = 245.1, 12.2 Hz), -107.06 (dd, *J* = 244.7, 11.4 Hz), -114.57 (s). IR (film): v 3068, 3001, 2949, 1898, 1604, 1509, 1478, 1454,

1417, 1236, 1166, 1151, 1039, 997, 848, 798, 763, 688. ESI-MS *m/z* (rel): 305.2 $(M+H)^+$; HRMS (ESI) Calcd. for $C_{17}H_{16}F_3N_2$ $(M+H)^+$: 305.1260; Found: 305.1261. HPLC (PC-3 (Phenomenex Cellulose-3), CH_3CN : H_2O = 70 : 30, 0.7 mL/min, 214 nm): t_{minor} = 9.40 min, t_{major} = 10.34 min.

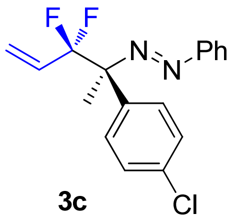

3c

Yellow oil; Yield: 86%; er: 94:6; $[\alpha]_D^{20}$ = -133.10 (*c* 1.0, $CHCl_3$); ^1H NMR (400 MHz, $CDCl_3$) δ 7.99 – 7.86 (m, 2H), 7.64 (d, *J* = 5.5 Hz, 3H), 7.54 (d, *J* = 8.2 Hz, 2H), 7.49 – 7.40 (m, 2H), 6.22 (dq, *J* = 17.4, 11.7 Hz, 1H), 5.75 (d, *J* = 17.4 Hz, 1H), 5.61 (d, *J* = 11.0 Hz, 1H), 1.86 (s, 3H). ^{13}C NMR (101 MHz, $CDCl_3$) δ 151.95, 138.74, 133.93, 131.53, 130.67 (t, *J* = 25.7 Hz), 129.88, 129.25, 128.22, 122.73, 121.43 (t, *J* = 9.5 Hz), 120.73 (t, *J* = 248.9 Hz), 78.54 (t, *J* = 24.2 Hz), 17.95. ^{19}F NMR (376 MHz, $CDCl_3$) δ -106.14 (dd, *J* = 245.1, 12.1 Hz), -106.97 (dd, *J* = 245.1, 11.5 Hz). IR (film): ν 3066, 3000, 2948, 1905, 1595, 1525, 1493, 1454, 1417, 1401, 1217, 1096, 1039, 997, 909, 808, 765, 688. ESI-MS *m/z* (rel): 321.1 $(M+H)^+$; HRMS (ESI) Calcd. for $C_{17}H_{16}N_2ClF_2$ $(M+H)^+$: 321.0965; Found: 321.0965. HPLC (PC-3 (Phenomenex Cellulose-3), CH_3CN : H_2O = 80 : 20, 0.7 mL/min, 214 nm): t_{minor} = 7.28 min, t_{major} = 8.47 min.

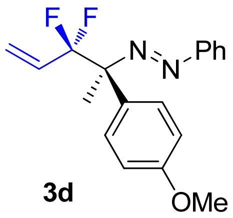

3d

Yellow oil; Yield: 96%; er: 95:5; $[\alpha]_D^{20}$ = -146.10 (*c* 1.0, $CHCl_3$); ^1H NMR (400 MHz, $CDCl_3$) δ 7.80 (dd, *J* = 7.0, 1.0 Hz, 2H), 7.51 (d, *J* = 6.9 Hz, 3H), 7.40 (d, *J* = 8.4 Hz, 2H), 6.88 (d, *J* = 8.9 Hz, 2H), 6.12 (dd, *J* = 17.3, 12.0 Hz, 1H), 5.63 (d, *J* = 17.4 Hz, 1H), 5.47 (d, *J* = 11.1 Hz, 1H), 3.81 (s, 3H), 1.74 (s, 3H). ^{13}C

NMR (101 MHz, $CDCl_3$) δ 158.93, 152.01, 132.21, 131.09 (t, *J* = 12.7 Hz), 129.44, 129.05, 122.56, 120.92 (t, *J* = 248.3 Hz), 120.87 (t, *J* = 9.3 Hz), 113.29, 78.42 (t, *J* = 24.1 Hz), 55.17, 17.87. ^{19}F NMR (376 MHz, $CDCl_3$) δ -106.63 (d, *J* = 11.9 Hz). IR (film): v 3065, 3000, 2951, 2837, 1895, 1610, 1581, 1513, 1454, 1416, 1299, 1254, 1184, 1034, 951, 820, 763, 688. ESI-MS *m/z* (rel): 317.1 $(M+H)^+$; HRMS (ESI) Calcd. for $C_{18}H_{19}ON_2F_2$ $(M+H)^+$: 317.1460; Found: 317.1460. HPLC (PC-3 (Phenomenex Cellulose-3), CH_3CN : H_2O = 70 : 30, 0.7 mL/min, 214 nm): t_{minor} = 9.34 min, t_{major} = 12.35 min.

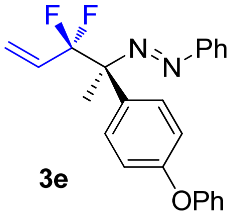

3e

Yellow oil; Yield: 93%; er: 94:6; $[\alpha]_D^{20}$ = -113.49 (*c* 1.4, $CHCl_3$); ^{1}H NMR (400 MHz, $CDCl_3$) δ 7.91 – 7.80 (m, 2H), 7.56 – 7.46 (m, 5H), 7.42 – 7.34 (m, 2H), 7.22 – 7.12 (m, 1H), 7.12 – 7.05 (m, 2H), 7.01 (dd, *J* = 8.7, 1.7 Hz, 2H), 6.28 – 6.04 (m, 1H), 5.68 (dd, *J* = 17.4, 1.1 Hz, 1H), 5.52 (d, *J* = 11.1 Hz, 1H), 1.80 (s, 3H). ^{13}C NMR (101 MHz, $CDCl_3$) δ 156.93, 156.73, 151.98, 134.70, 131.24, 130.87 (t, *J* = 25.7 Hz), 129.80, 129.76, 129.11, 123.58, 122.61, 121.06 (t, *J* = 9.5 Hz), 120.85 (t, *J* = 248.8 Hz), 119.32, 117.81, 78.51 (t, *J* = 24.2 Hz), 17.92. ^{19}F NMR (376 MHz, $CDCl_3$) δ -105.53 – -106.48 (dd, *J* = 244.3, 11.6 Hz), -106.86 (dd, *J* = 244.3, 11.6 Hz). IR (film): v 3065, 2999, 1588, 1506, 1489, 1454, 1416, 1373, 1286, 1243, 1174, 1039, 996, 873, 764, 690. ESI-MS *m/z* (rel): 379.2 $(M+H)^+$; HRMS (ESI) Calcd. for $C_{23}H_{21}ON_2F_2$ $(M+H)^+$: 379.1616; Found: 379.1615. HPLC (Chiralpak OD-H, Hexane : *i*-Propanol = 98 : 2, 0.7 mL/min, 214 nm): t_{major} = 10.56 min, t_{minor} = 15.52 min.

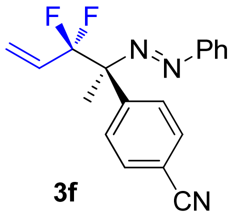

3f

Yellow oil; Yield: 67%; er: 86:14; $[\alpha]_D^{20}$ = -118.50 (c 1.0, $CHCl_3$); ^{1}H NMR (400 MHz, $CDCl_3$) δ 7.85 – 7.71 (m, 2H), 7.59 (q, J = 8.3 Hz, 4H), 7.54 – 7.46 (m, 3H), 6.04 (dq, J = 17.3, 11.8 Hz, 1H), 5.60 (d, J = 17.4 Hz, 1H), 5.48 (d, J = 11.1 Hz, 1H), 1.73 (s, 3H). ^{13}C NMR (101 MHz, $CDCl_3$) δ 151.76, 145.39, 131.84, 131.74, 130.23 (t, J = 25.3 Hz), 129.36, 129.32, 122.77, 121.86 (t, J = 9.2 Hz), 120.48 (t, J = 249.3 Hz), 118.72, 111.80, 78.75 (t, J = 24.3 Hz), 17.96. ^{19}F NMR (376 MHz, $CDCl_3$) δ -105.58 (dd, J = 246.2, 12.3 Hz), -106.84 (dd, J = 246.2, 11.6 Hz). IR (film): ν 3067, 3000, 2949, 2230, 1922, 1608, 1523, 1504, 1454, 1375, 1301, 1215, 1040, 954, 818, 763, 688. ESI-MS *m/z* (rel): 312.1 $(M+H)^+$; HRMS (ESI) Calcd. for $C_{18}H_{16}N_3F_2$ $(M+H)^+$: 312.1307; Found: 312.1307. HPLC (Chiralpak IG, Hexane : *i*-Propanol = 95 : 5, 0.7 mL/min, 214 nm): t_{minor} = 8.68 min, t_{major} = 8.98 min.

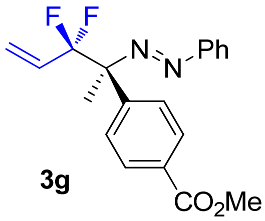

3g

Yellow oil; Yield: 77%; er: 96:4; $[\alpha]_D^{20}$ = -133.00 (c 1.0, $CHCl_3$); ^{1}H NMR (400 MHz, $CDCl_3$) δ 7.98 (d, J = 8.3 Hz, 2H), 7.85 – 7.71 (m, 2H), 7.51 (dd, J = 11.8, 6.7 Hz, 5H), 6.07 (dq, J = 17.3, 11.8 Hz, 1H), 5.58 (d, J = 17.4 Hz, 1H), 5.45 (d, J = 11.2 Hz, 1H), 3.89 (s, 3H), 1.74 (s, 3H). ^{13}C NMR (101 MHz, $CDCl_3$) δ 166.90, 151.91, 145.27, 131.57, 130.59 (t, J = 26.2 Hz), 129.51, 129.25, 129.19, 128.50, 122.74, 121.48 (t, J = 9.4 Hz), 120.66 (t, J = 249.2 Hz), 78.95 (t, J = 24.1 Hz), 52.30, 52.25, 17.93. ^{19}F NMR (376 MHz, $CDCl_3$) δ -105.98 (dd, J = 245.4, 12.4 Hz), -106.81 (dd, J = 245.3, 11.7 Hz). IR (film): ν 3065, 3000, 2951, 1935, 1724, 1611, 1524, 1436, 1281, 1192, 1115, 1019, 953, 814, 771,

690. ESI-MS *m/z* (rel): 345.1 $(M+H)^+$; HRMS (ESI) Calcd. for $C_{19}H_{19}O_2N_2F_2$ $(M+H)^+$: 345.1409; Found: 345.1410. HPLC (Chiralpak OJ-H, Hexane : *i*-Propanol = 98 : 2, 0.7 mL/min, 214 nm): t_{major} = 23.02 min, t_{minor} = 31.77 min.

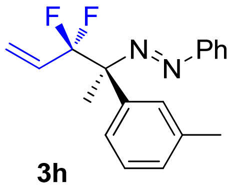

3h

Yellow oil; Yield: 90%; er: 96:4; $[\alpha]_D^{20}$ = -144.76 (*c* 1.0, $CHCl_3$); ^{1}H NMR (400 MHz, $CDCl_3$) δ 7.96 (dd, *J* = 4.7, 2.2 Hz, 2H), 7.64 (t, *J* = 9.2 Hz, 3H), 7.53 – 7.32 (m, 3H), 7.27 (d, *J* = 7.0 Hz, 1H), 6.29 (dq, *J* = 17.3, 11.7 Hz, 1H), 5.79 (d, *J* = 17.4 Hz, 1H), 5.62 (d, *J* = 11.1 Hz, 1H), 2.51 (s, 3H), 1.91 (s, 3H). ^{13}C NMR (101 MHz, $CDCl_3$) δ 152.14, 140.32, 137.60, 131.36, 131.10 (t, *J* = 25.4 Hz), 129.21, 128.98, 128.61, 127.95, 125.49, 122.74, 121.03 (t, *J* = 9.4 Hz), 120.99 (t, *J* = 248.7 Hz), 120.93, 78.90 (t, *J* = 24.0 Hz), 21.85, 18.08. ^{19}F NMR (376 MHz, $CDCl_3$) δ -105.95 (dd, *J* = 245.2, 12.0 Hz), -106.77 (dd, *J* = 244.7, 11.5 Hz). IR (film): ν 3064, 3000, 2948, 1953, 1606, 1525, 1454, 1416, 1373, 1220, 1040, 996, 950, 778, 688. ESI-MS *m/z* (rel): 301.2 $(M+H)^+$; HRMS (ESI) Calcd. for $C_{18}H_{19}N_2F_2$ $(M+H)^+$: 301.1511; Found: 301.1511. HPLC (Chiralpak OJ-H, Hexane : *i*-Propanol = 98 : 2, 0.7 mL/min, 214 nm): t_{major} = 7.88 min, t_{minor} = 9.33 min.

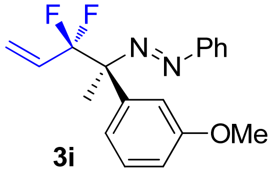

3i

Yellow oil; Yield: 73%; er: 96:4; $[\alpha]_D^{20}$ = -173.08 (*c* 1.0, $CHCl_3$); ^{1}H NMR (400 MHz, $CDCl_3$) δ 7.80 (d, *J* = 7.1 Hz, 2H), 7.49 (d, *J* = 6.1 Hz, 3H), 7.26 (t, *J* = 8.3 Hz, 1H), 7.05 (s, 2H), 6.85 (d, *J* = 7.9 Hz, 1H), 6.12 (dq, *J* = 17.3, 12.0 Hz, 1H), 5.63 (d, *J* = 17.3 Hz, 1H), 5.46 (d, *J* = 11.1 Hz, 1H), 3.78 (s, 3H), 1.75 (s, 3H). ^{13}C NMR (101 MHz, $CDCl_3$) δ 159.03, 151.94, 141.68, 131.17, 130.85 (t, *J* =

25.6 Hz), 129.06, 128.85, 122.59, 120.92 (t, J = 9.5 Hz), 120.73 (t, J = 249.5 Hz), 120.60, 114.92, 112.46, 78.69 (t, J = 24.1 Hz), 55.18, 17.82 (t, J = 3.2 Hz). ^{19}F NMR (376 MHz, $CDCl_3$) δ -106.02 (dd, J = 245.3, 11.9 Hz), -106.73 (dd, J = 245.3, 11.9 Hz). IR (film): v 3086, 3008, 2838, 1607, 1580, 1486, 1453, 1422, 1292, 1082, 1038, 988, 878, 770, 692. ESI-MS *m/z* (rel): 317.1 $(M+H)^+$; HRMS (ESI) Calcd. for $C_{18}H_{19}ON_2F_2$ $(M+H)^+$: 317.1460; Found: 317.1456. HPLC (Chiralpak AD-H, Hexane : *i*-Propanol = 98 : 2, 0.7 mL/min, 214 nm): t_{minor} = 6.31 min, t_{major} = 6.99 min.

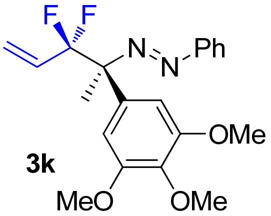

3k

Yellow oil; Yield: 70%; er: 95:5; $[\alpha]_D^{20}$ = -152.80 (*c* 1.0, $CHCl_3$); ^{1}H NMR (400 MHz, $CDCl_3$) δ 7.84 – 7.73 (m, 2H), 7.58 – 7.44 (m, 3H), 6.70 (s, 2H), 6.09 (dq, J = 17.4, 11.8 Hz, 1H), 5.64 (d, J = 17.4 Hz, 1H), 5.47 (d, J = 11.1 Hz, 1H), 3.85 (s, 3H), 3.82 (s, 6H), 1.73 (s, 3H). ^{13}C NMR (101 MHz, $CDCl_3$) δ 152.53, 151.95, 137.72, 135.50, 131.23, 130.82 (t, J = 25.7 Hz), 129.13, 122.45, 120.96 (t, J = 9.5 Hz), 120.73 (t, J = 249.0 Hz), 106.02, 78.60 (t, J = 24.0 Hz), 60.79, 56.15, 17.90. ^{19}F NMR (376 MHz, $CDCl_3$) δ -105.76 (dd, J = 244.8, 12.1 Hz), -106.57 (dd, J = 244.5, 11.6 Hz). IR (film): v 3070, 2998, 2938, 2836, 1588, 1510, 1454, 1414, 1328, 1248, 1131, 1043, 1003, 951, 832, 770, 688. ESI-MS *m/z* (rel): 377.2 $(M+H)^+$; HRMS (ESI) Calcd. for $C_{20}H_{22}O_3N_2F_2Na$ $(M+Na)^+$: 399.1491; Found: 399.1490. HPLC (PC-3 (Phenomenex Cellulose-3), CH_3CN : H_2O = 70 : 30, 0.7 mL/min, 214 nm): t_{minor} = 7.12 min, t_{major} = 7.67 min.

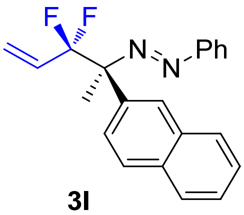

3l

Yellow oil; Yield: 95%; er: 94:6; $[\alpha]_D^{20}$ = -163.60 (*c* 1.0, $CHCl_3$); ^1H NMR (400 MHz, $CDCl_3$) δ 7.94 (s, 1H), 7.92 – 7.80 (m, 5H), 7.66 (d, *J* = 8.4 Hz, 1H), 7.61 – 7.46 (m, 5H), 6.20 (dq, *J* = 16.9, 11.8 Hz, 1H), 5.68 (d, *J* = 17.4 Hz, 1H), 5.49 (d, *J* = 11.1 Hz, 1H), 1.91 (s, 3H). ^{13}C NMR (101 MHz, $CDCl_3$) δ 152.05, 137.75, 132.83, 132.64, 131.27, 130.94 (t, *J* = 25.7 Hz), 129.14, 128.45, 127.62, 127.45, 126.39, 126.14, 126.08, 122.67, 121.09 (t, *J* = 9.3 Hz), 79.01 (t, *J* = 23.7 Hz), 18.10. ^{19}F NMR (376 MHz, $CDCl_3$) δ -105.69 (d, *J* = 243.3 Hz), -106.38 (d, *J* = 252.4 Hz). IR (film): ν 3060, 3000, 2984, 1910, 1598, 1524, 1453, 1416, 1375, 1300, 1216, 1183, 1039, 950, 818, 747, 688. ESI-MS *m/z* (rel): 337.1 $(M+H)^+$; HRMS (ESI) Calcd. for $C_{21}H_{19}N_2F_2$ $(M+H)^+$: 337.1511; Found: 337.1510. HPLC (PC-3 (Phenomenex Cellulose-3), CH_3CN : H_2O = 80 : 20, 0.7 mL/min, 214 nm): t_{minor} = 7.79 min, t_{major} = 12.28 min.

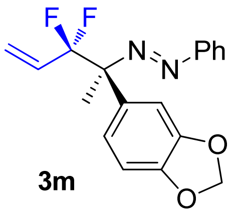

3m

Yellow oil; Yield: 91%; er: 95:5; $[\alpha]_D^{20}$ = -170.90 (*c* 1.0, $CHCl_3$); ^1H NMR (400 MHz, $CDCl_3$) δ 7.86 – 7.70 (m, 2H), 7.59 – 7.42 (m, 3H), 7.00 (s, 1H), 6.92 (d, *J* = 8.3 Hz, 1H), 6.77 (d, *J* = 8.3 Hz, 1H), 6.10 (dq, *J* = 17.1, 11.7 Hz, 1H), 5.95 (dd, *J* = 2.4, 1.8 Hz, 2H), 5.63 (d, *J* = 17.4 Hz, 1H), 5.47 (d, *J* = 11.1 Hz, 1H), 1.70 (s, 3H). ^{13}C NMR (101 MHz, $CDCl_3$) δ 151.93, 147.25, 146.95, 133.76, 131.19, 130.82 (t, *J* = 26.1 Hz), 129.05, 122.58, 121.77, 120.94 (t, *J* = 9.5 Hz), 109.16, 107.66, 101.07, 78.44 (t, *J* = 24.1 Hz), 17.99. ^{19}F NMR (376 MHz, $CDCl_3$) δ -106.55 (s). IR (film): ν 3066, 2999, 2893, 1610, 1528, 1505, 1488, 1435, 1345, 1251, 1234, 1081, 1039, 937, 797, 689. ESI-MS *m/z* (rel): 331.1 $(M+H)^+$; HRMS (ESI) Calcd. for $C_{18}H_{17}O_2N_2F_2$ $(M+H)^+$: 331.1253; Found: 331.1254. HPLC (Chiralpak OJ-H, Hexane : *i*-Propanol = 98 : 2, 0.7 mL/min, 214 nm): t_{major} = 18.89 min, t_{minor} = 24.38 min.

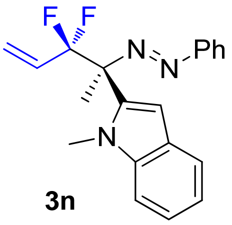

3n

Yellow oil; Yield: 35%; er: 94:6; $[\alpha]_D^{20}$ = -50.16 (*c* 1.0, $CHCl_3$); ^{1}H NMR (400 MHz, $CDCl_3$) δ 7.81 (d, *J* = 7.4 Hz, 2H), 7.67 (d, *J* = 8.1 Hz, 1H), 7.56 – 7.46 (m, 3H), 7.29 (d, *J* = 8.2 Hz, 1H), 7.21 (t, *J* = 7.6 Hz, 1H), 7.12 – 7.04 (m, 2H), 6.23 (dq, *J* = 17.5, 10.9 Hz, 1H), 5.74 (d, *J* = 17.3 Hz, 1H), 5.49 (d, *J* = 11.0 Hz, 1H), 3.75 (s, 3H), 1.86 (s, 3H). ^{13}C NMR (101 MHz, $CDCl_3$) δ 152.21, 137.26, 131.61 (t, *J* = 24.6 Hz), 131.08, 129.17, 128.62, 128.57, 126.68, 123.16, 122.73, 121.77 (t, *J* = 248.0 Hz), 121.67, 120.73 (t, *J* = 9.4 Hz), 113.83, 109.41, 77.99 (t, *J* = 24.7 Hz), 33.05, 32.98, 18.49. ^{19}F NMR (376 MHz, $CDCl_3$) δ -104.97 (dd, *J* = 242.4, 9.7 Hz), -106.83 (dd, *J* = 242.5, 10.8 Hz). IR (film): ν 3052, 2998, 2946, 1744, 1614, 1541, 1476, 1416, 1332, 1209, 1143, 1071, 986, 909, 802, 741, 689. ESI-MS *m/z* (rel): 340.2 $(M+H)^+$; HRMS (ESI) Calcd. for $C_{20}H_{20}N_3F_2$ $(M+H)^+$: 340.1620; Found: 340.1620. HPLC (PC-3 (Phenomenex Cellulose-3), CH_3CN : H_2O = 80 : 20, 0.7 mL/min, 214 nm): t_{minor} = 14.04 min, t_{major} = 16.83 min.

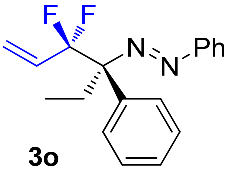

3o

Yellow oil; Yield: 90%; er: 93:7; $[\alpha]_D^{20}$ = -25.30 (*c* 1.0, $CHCl_3$); ^{1}H NMR (400 MHz, $CDCl_3$) δ 7.85 (dd, *J* = 7.2, 1.5 Hz, 2H), 7.57 (dd, *J* = 21.2, 7.3 Hz, 5H), 7.42 (t, *J* = 7.6 Hz, 2H), 7.36 (d, *J* = 7.3 Hz, 1H), 6.00 (dq, *J* = 17.1, 11.8 Hz, 1H), 5.50 (d, *J* = 17.4 Hz, 1H), 5.38 (d, *J* = 11.1 Hz, 1H), 2.64 (dq, *J* = 14.4, 7.2 Hz, 1H), 2.36 (dq, *J* = 14.7, 7.3 Hz, 1H), 0.71 (t, *J* = 7.2 Hz, 3H). ^{13}C NMR (101 MHz, $CDCl_3$) δ 152.03, 137.75, 131.24 (t, *J* = 25.6 Hz), 130.99, 129.10, 129.01, 128.03, 127.55, 122.57, 120.43 (t, *J* = 9.6 Hz), 117.74, 81.67 (t, *J* = 22.6 Hz), 22.66, 8.14. ^{19}F NMR (376 MHz, $CDCl_3$) δ -104.00 (dd, *J* = 243.4, 12.2 Hz),

-106.83 (dd, *J* = 243.4, 11.8 Hz). IR (film): ν 3061, 3027, 2940, 1601, 1525, 1447, 1416, 1302, 1260, 1150, 1069, 981, 949, 764, 688. ESI-MS *m/z* (rel): 301.2 $(M+H)^+$; HRMS (ESI) Calcd. for $C_{18}H_{19}N_2F_2$ $(M+H)^+$: 301.1511; Found: 301.1513. HPLC (Chiralpak OJ-H, Hexane : *i*-Propanol = 98 : 2, 0.7 mL/min, 214 nm): t_{major} = 9.26 min, t_{minor} = 10.13 min.

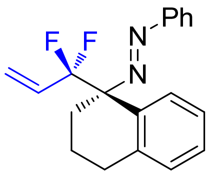

3p

Yellow oil; Yield: 85%; er: 92:8; $[\alpha]_D^{20}$ = -112.04 (*c* 1.0, $CHCl_3$); ^{1}H NMR (400 MHz, $CDCl_3$) δ 7.71 (dd, *J* = 7.6, 1.6 Hz, 2H), 7.60 (d, *J* = 7.8 Hz, 1H), 7.47 (ddd, *J* = 7.2, 3.9, 1.5 Hz, 3H), 7.23 (ddd, *J* = 23.1, 10.3, 5.0 Hz, 3H), 6.14 (dq, *J* = 17.3, 11.7 Hz, 1H), 5.76 (d, *J* = 17.3 Hz, 1H), 5.54 (d, *J* = 11.1 Hz, 1H), 2.81 (t, *J* = 6.2 Hz, 2H), 2.38 (d, *J* = 12.7 Hz, 1H), 2.13 (ddd, *J* = 14.2, 10.8, 3.4 Hz, 1H), 2.05 – 1.92 (m, 1H), 1.72 – 1.56 (m, 1H). ^{13}C NMR (101 MHz, $CDCl_3$) δ 152.11, 140.01, 133.27, 131.18 (t, *J* = 25.7 Hz), 131.09 (t, *J* = 3.7 Hz), 130.86, 129.00, 128.95, 127.60, 125.58, 122.49, 122.21 (t, *J* = 249.5 Hz), 121.00 (t, *J* = 9.5 Hz), 77.50 (t, *J* = 22.2 Hz), 30.21, 28.95, 18.87. ^{19}F NMR (376 MHz, $CDCl_3$) δ -103.45 (dd, *J* = 247.7, 11.5 Hz), -105.26 (dd, *J* = 247.8, 11.2 Hz). IR (film): ν 3061, 2940, 2877, 1597, 1490, 1453, 1416, 1287, 1212, 1149, 1062, 988, 951, 764, 689. ESI-MS *m/z* (rel): 313.2 $(M+H)^+$; HRMS (ESI) Calcd. for $C_{19}H_{19}N_2F_2$ $(M+H)^+$: 313.1511; Found: 313.1507. HPLC (PC-3 (Phenomenex Cellulose-3), CH_3CN : H_2O = 70 : 30, 0.7 mL/min, 214 nm): t_{major} = 10.56 min, t_{minor} = 11.51 min.

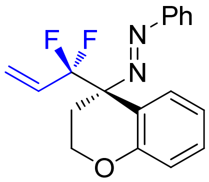

3q

Yellow oil; Yield: 74%; er: 95:5; $[\alpha]_D^{20}$ = -109.07 (*c* 1.5, $CHCl_3$); ^1H NMR (400 MHz, $CDCl_3$) δ 7.73 (dd, *J* = 4.9, 1.5 Hz, 2H), 7.65 (d, *J* = 7.7 Hz, 1H), 7.46 (dd, *J* = 5.2, 1.5 Hz, 3H), 7.30 – 7.19 (m, 1H), 6.94 (ddd, *J* = 8.5, 6.7, 2.0 Hz, 2H), 6.19 – 6.00 (m, 1H), 5.77 (d, *J* = 17.3 Hz, 1H), 5.56 (d, *J* = 11.1 Hz, 1H), 4.41 – 4.29 (m, 1H), 3.94 (t, *J* = 9.5 Hz, 1H), 2.58 (d, *J* = 14.8 Hz, 1H), 2.47 – 2.31 (m, 1H). ^{13}C NMR (101 MHz, $CDCl_3$) δ 156.31, 151.65, 131.26, 130.93 (t, *J* = 3.4 Hz), 130.42 (t, *J* = 25.4 Hz), 129.53, 129.01, 122.62, 121.72 (t, *J* = 9.6 Hz), 121.67 (t, *J* = 249.6 Hz), 120.45, 119.07, 117.35, 73.87 (t, *J* = 23.0 Hz), 62.34, 27.49. ^{19}F NMR (376 MHz, $CDCl_3$) δ -103.44 (dd, *J* = 248.7, 10.3 Hz), -105.88 (dd, *J* = 248.8, 11.9 Hz). IR (film): v 3066, 2976, 2886, 1916, 1607, 1581, 1522, 1451, 1417, 1309, 1225, 1050, 996, 688. ESI-MS *m/z* (rel): 315.1 $(M+H)^+$; HRMS (ESI) Calcd. for $C_{18}H_{17}ON_2F_2$ $(M+H)^+$: 315.1303; Found: 315.1300. HPLC (Chiralpak OJ-H, Hexane : *i*-Propanol = 95 : 5, 0.7 mL/min, 214 nm): t_{major} = 10.84 min, t_{mino} = 13.06 min.

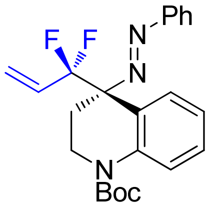

3r

Yellow oil; Yield: 76%; er: 96:4; $[\alpha]_D^{20}$ = -213.97 (*c* 1.3, $CHCl_3$); ^1H NMR (400 MHz, $CDCl_3$) δ 7.77 – 7.70 (m, 2H), 7.65 (t, *J* = 7.3 Hz, 2H), 7.51 – 7.43 (m, 3H), 7.28 (dd, *J* = 11.5, 4.3 Hz, 1H), 7.09 (t, *J* = 7.6 Hz, 1H), 6.02 (ddd, *J* = 23.6, 17.3, 11.1 Hz, 1H), 5.72 (d, *J* = 17.3 Hz, 1H), 5.52 (d, *J* = 11.1 Hz, 1H), 4.34 (dd, *J* = 9.2, 3.9 Hz, 1H), 3.12 – 2.99 (m, 1H), 2.57 (d, *J* = 14.7 Hz, 1H), 2.36 – 2.23 (m, 1H), 1.54 (s, 9H). ^{13}C NMR (101 MHz, $CDCl_3$) δ 153.37, 151.72, 140.67, 131.27, 130.70 (t, *J* = 3.2 Hz), 130.38 (t, *J* = 25.6 Hz), 129.03, 127.75, 126.64, 124.90, 123.59, 122.60, 121.74 (t, *J* = 9.3 Hz), 121.54 (t, *J* = 249.7 Hz), 81.04, 75.43 (t, *J* = 22.8 Hz), 41.14, 28.91, 28.35. ^{19}F NMR (376 MHz, $CDCl_3$) δ -104.79 (d, *J* = 246.5 Hz), -108.42 (dd, *J* = 245.5, 11.1 Hz). IR (film): v 3067,

2976, 2933, 1701, 1602, 1578, 1521, 1490, 1455, 1377, 1254, 1159, 1016, 954, 765, 689. ESI-MS *m/z* (rel): 414.2 $(M+H)^+$; HRMS (ESI) Calcd. for $C_{23}H_{26}O_2N_3F_2$ $(M+H)^+$: 414.1988; Found: 414.1986. HPLC (Chiralpak IE3, Hexane : *i*-Propanol = 98 : 2, 0.7 mL/min, 214 nm): t_{minor} = 7.57 min, t_{major} = 7.94 min.

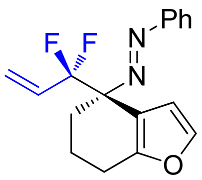

3s

Yellow oil; Yield: 80%; er: 94:6; $[\alpha]_D^{20}$ = -36.62 (*c* 1.2, $CHCl_3$); ^1H NMR (400 MHz, $CDCl_3$) δ 7.74 – 7.65 (m, 2H), 7.49 – 7.41 (m, 3H), 7.32 (s, 1H), 6.45 (s, 1H), 6.22 (dq, *J* = 17.5, 11.7 Hz, 1H), 5.81 (d, *J* = 17.4 Hz, 1H), 5.59 (d, *J* = 11.1 Hz, 1H), 2.70 – 2.54 (m, 2H), 2.40 – 2.31 (m, 1H), 2.12 – 2.01 (m, 1H), 1.94 (dd, *J* = 9.5, 4.2 Hz, 1H), 1.62 (tdd, *J* = 12.9, 9.6, 3.0 Hz, 1H). ^{13}C NMR (101 MHz, $CDCl_3$) δ 154.64, 151.96, 140.47, 130.92 (t, *J* = 25.4 Hz), 130.67, 128.90, 122.48, 121.51 (t, *J* = 247.8 Hz), 121.06 (t, *J* = 9.7 Hz), 113.52, 110.81, 76.67 (t, *J* = 23.2 Hz), 27.79 (t, *J* = 2.3 Hz), 22.80, 18.42. ^{19}F NMR (376 MHz, $CDCl_3$) δ -106.03 (dd, *J* = 247.5, 11.2 Hz), -107.11 (d, *J* = 246.2 Hz). IR (film): v 3065, 2948, 1848, 1807, 1648, 1525, 1478, 1454, 1416, 1338, 1310, 1217, 1096, 1042, 993, 896, 766, 689. ESI-MS *m/z* (rel): 303.1 $(M+H)^+$; HRMS (ESI) Calcd. for $C_{17}H_{17}ON_2F_2$ $(M+H)^+$: 303.1303; Found: 303.1305. HPLC (Chiralpak OJ-H, Hexane : *i*-Propanol = 98 : 2, 0.7 mL/min, 214 nm): t_{minor} = 10.73 min, t_{major} = 13.31 min.

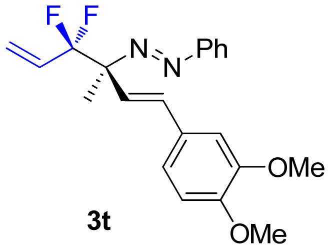

3t

Yellow oil; Yield: 73%; er: 95:5; $[\alpha]_D^{27}$ = 49.11 (*c* 1.0, $CHCl_3$); ^1H NMR (400 MHz, $CDCl_3$) δ 7.78 (dd, *J* = 7.6, 1.6 Hz, 2H), 7.57 – 7.40 (m, 3H), 6.97 (d, *J* = 5.1 Hz, 2H), 6.88 – 6.79 (m, 1H), 6.48 (q, *J* = 16.4 Hz, 2H), 6.27 (dq, *J* = 17.4, 12.0 Hz, 1H), 5.81 (d, *J* = 17.3 Hz, 1H), 5.59 (d, *J* = 11.1 Hz, 1H), 3.91 (s, 3H), 3.89 (s, 3H), 1.60 (s, 3H). ^{13}C NMR (101 MHz, $CDCl_3$) δ 152.11, 149.24, 149.14, 132.65, 131.09 (t, *J* = 25.6 Hz), 131.21, 129.74, 129.15, 124.91 (t, *J* = 2.6 Hz), 121.14 (t, *J* = 9.4 Hz), 122.65, 121.07 (t, *J* = 248.1 Hz), 120.01, 111.17, 109.12, 78.37 (t, *J* = 24.1 Hz), 56.04, 55.99, 16.88. ^{19}F NMR (376 MHz, $CDCl_3$) δ -107.45 (dd, *J* = 244.4, 11.4 Hz), -108.36 (dd, *J* = 244.4, 12.3 Hz). IR (film): v 3059, 2998, 2937, 2835, 1061, 1583, 1512, 1453, 1416, 1263, 1234, 1138, 1073, 1025, 762, 689. ESI-MS *m/z* (rel): 373.2 $(M+H)^+$; HRMS (ESI) Calcd. for $C_{21}H_{23}O_2N_2F_2$ $(M+H)^+$: 373.1718; Found: 373.1720. HPLC (Chiralpak IA, Hexane : *i*-Propanol = 95 : 5, 0.7 mL/min, 214 nm): t_{minor} = 12.62 min, t_{major} = 17.57 min.

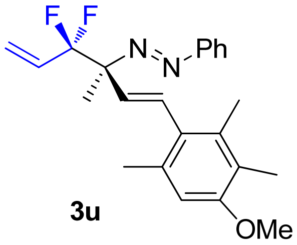

3u

Yellow oil; Yield: 96%; er: 96:4; $[\alpha]_D^{28}$ = -7.96 (*c* 1.0, $CHCl_3$); ^1H NMR (400 MHz, $CDCl_3$) δ 7.80 (d, *J* = 6.2 Hz, 2H), 7.52 (d, *J* = 7.0 Hz, 3H), 6.69 – 6.52 (m, 2H), 6.42 – 6.23 (m, 1H), 5.96 – 5.80 (m, 2H), 5.62 (d, *J* = 11.0 Hz, 1H), 3.85 (s, 3H), 2.33 (s, 3H), 2.26 (s, 3H), 2.20 (s, 3H), 1.67 (s, 3H). ^{13}C NMR (101 MHz, $CDCl_3$) δ 156.36, 152.21, 135.94, 133.84, 132.10, 131.98, 131.22 (t, *J* = 25.6 Hz), 131.18, 129.76, 129.19, 122.65, 122.58, 121.10 (t, *J* = 247.4 Hz), 121.05 (t, *J* = 9.5 Hz), 109.88, 78.73 (t, *J* = 24.2 Hz), 55.65, 21.35, 17.40, 16.91, 11.94. ^{19}F NMR (376 MHz, $CDCl_3$) δ -107.34 (dd, *J* = 244.9, 10.1 Hz), -108.72 (dd, *J* = 245.1, 11.3 Hz). IR (film): v 2995, 2941, 1593, 1523, 1453, 1415, 1371, 1309, 1219, 1119, 1073, 1042, 992, 949, 836, 762, 688. ESI-MS *m/z* (rel): 385.2 $(M+H)^+$; HRMS (ESI) Calcd. for $C_{23}H_{27}ON_2F_2$ $(M+H)^+$: 385.2086; Found:

385.2085. HPLC (Chiralpak OJ-H, Hexane : *i*-Propanol = 98 : 2, 0.7 mL/min, 214 nm): t_{major} = 7.48 min, t_{minor} = 9.09 min.

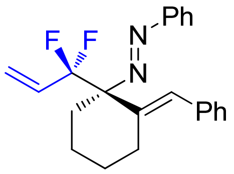

3v

Yellow oil; Yield: 97%; er: 94.5:5.5; $[\alpha]_D^{20}$ = -80.40 (*c* 1.0, $CHCl_3$); ^1H NMR (400 MHz, $CDCl_3$) δ 7.81 (d, *J* = 7.1 Hz, 2H), 7.57 – 7.47 (m, 3H), 7.35 (t, *J* = 7.3 Hz, 2H), 7.23 (dd, *J* = 14.4, 7.6 Hz, 3H), 6.67 (s, 1H), 6.48 – 6.24 (m, 1H), 5.78 (d, *J* = 17.4 Hz, 1H), 5.55 (d, *J* = 11.0 Hz, 1H), 2.84 (d, *J* = 14.1 Hz, 1H), 2.49 (t, *J* = 12.6 Hz, 1H), 2.35 – 2.20 (m, 2H), 1.97 (s, 1H), 1.80 (s, 2H), 1.45 (d, *J* = 10.0 Hz, 1H). ^{13}C NMR (101 MHz, $CDCl_3$) δ 152.13, 140.10, 137.91, 131.80 (t, *J* = 25.3 Hz), 131.20, 129.92, 129.23, 129.16, 128.19, 126.68, 122.64, 122.20 (t, *J* = 251.2 Hz), 120.11 (t, *J* = 9.7 Hz), 78.60 (t, *J* = 23.6 Hz), 28.87, 27.67, 26.10, 22.00, 21.98. ^{19}F NMR (376 MHz, $CDCl_3$) δ -100.35 (dd, *J* = 247.3, 12.7 Hz), -103.15 (dd, *J* = 247.2, 11.2 Hz). IR (film): ν 3057, 2937, 2873, 1599, 1526, 1494, 1453, 1416, 1218, 1142, 1071, 994, 855, 788, 688. ESI-MS *m/z* (rel): 353.2 $(M+H)^+$; HRMS (ESI) Calcd. for $C_{22}H_{23}N_2F_2$ $(M+H)^+$: 353.1824; Found: 353.1824. HPLC (PC-3 (Phenomenex Cellulose-3), CH_3CN : H_2O = 70 : 30, 0.7 mL/min, 214 nm): t_{major} = 17.91 min, t_{minor} = 23.47 min.

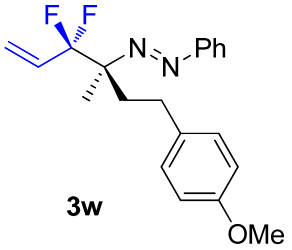

3w

Yellow oil; Yield: 57%; er: 96:4; $[\alpha]_D^{20}$ = 85.00 (*c* 1.0, $CHCl_3$); ^1H NMR (400 MHz, $CDCl_3$) δ 7.88 (d, *J* = 7.7 Hz, 2H), 7.72 – 7.53 (m, 3H), 7.21 (d, *J* = 8.5 Hz, 2H), 6.95 (d, *J* = 8.5 Hz, 2H), 6.34 (tt, *J* = 12.5, 11.1 Hz, 1H), 5.92 (d, *J* = 17.4 Hz, 1H), 5.69 (d, *J* = 11.1 Hz, 1H), 3.91 (s, 3H), 2.77 – 2.59 (m, 1H), 2.47 (td, *J*

= 16.2, 4.1 Hz, 2H), 2.27 (td, *J* = 13.4, 4.9 Hz, 1H), 1.59 (s, 3H). ^{13}C NMR (101 MHz, $CDCl_3$) δ 157.96, 151.98, 134.07, 131.19, 130.94 (t, *J* = 25.4 Hz), 129.37, 129.16, 122.55, 121.82 (t, *J* = 248.5 Hz), 120.80 (t, *J* = 9.4 Hz), 113.96, 77.20 (t, *J* = 23.2 Hz), 55.38, 35.66, 28.73, 15.15. ^{19}F NMR (376 MHz, $CDCl_3$) δ -106.58 (dd, *J* = 246.0, 10.6 Hz), -109.88 (dd, *J* = 245.9, 13.6 Hz). IR (film): v 3063, 2997, 2953, 1611, 1513, 1454, 1417, 1375, 1301, 1247, 1178, 1054, 994, 821, 689. ESI-MS *m/z* (rel): 345.2 $(M+H)^+$; HRMS (ESI) Calcd. for $C_{20}H_{23}ON_2F_2$ $(M+H)^+$: 345.1773; Found: 345.1773. HPLC (PC-3 (Phenomenex Cellulose-3), CH_3CN : H_2O = 60 : 40, 0.7 mL/min, 214 nm): t_{minor} = 35.86 min, t_{major} = 38.81 min.

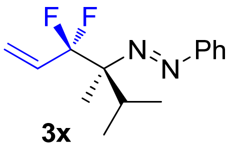

3x

Yellow oil; Yield: 50%; er: 96:4; $[\alpha]_D^{30}$ = 86.55 (*c* 1.0, $CHCl_3$); ^1H NMR (400 MHz, $CDCl_3$) δ 7.71 (dt, *J* = 17.3, 8.8 Hz, 2H), 7.56 – 7.44 (m, 3H), 6.34 – 6.12 (m, 1H), 5.79 (dd, *J* = 17.4, 1.6 Hz, 1H), 5.54 (d, *J* = 11.1 Hz, 1H), 2.71 (dt, *J* = 13.7, 6.8 Hz, 1H), 1.29 (s, 3H), 1.17 – 1.11 (m, 3H), 0.66 (d, *J* = 6.9 Hz, 3H). ^{13}C NMR (101 MHz, $CDCl_3$) δ 150.78, 130.31 (t, *J* = 25.3 Hz), 129.64, 127.95, 120.77 (dd, *J* = 251.1, 245.7 Hz), 121.34, 118.94 (t, *J* = 9.8 Hz), 79.28 (t, *J* = 22.6 Hz), 29.68, 16.96 (dd, *J* = 4.5, 1.6 Hz), 16.13 (d, *J* = 1.2 Hz), 8.22 (t, *J* = 3.6 Hz). ^{19}F NMR (376 MHz, $CDCl_3$) δ -99.88 (dd, *J* = 249.1, 8.4 Hz), -107.76 (dd, *J* = 249.1, 15.3 Hz). IR (film): v 3066, 2968, 2881, 1528, 1469, 1453, 1416, 1392,, 1374, 1228, 1150, 1076, 1051, 1018, 991, 927, 762, 687. ESI-MS *m/z* (rel): 253.2 $(M+H)^+$; HRMS (ESI) Calcd. for $C_{14}H_{19}N_2F_2$ $(M+H)^+$: 253.1511; Found: 253.1511. HPLC (Chiralpak OJ-H, Hexane : *i*-Propanol = 95 : 5, 0.7 mL/min, 214 nm): t_{major} = 5.74 min, t_{minor} = 7.20 min.

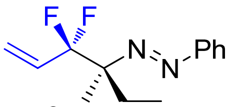

3y

Yellow oil; Yield: 74%; er: 88:12; $[\alpha]_D^{30}$ = 88.31 (*c* 1.0, $CHCl_3$); ^{1}H NMR (400 MHz, $CDCl_3$) δ 7.72 (dd, *J* = 7.9, 1.7 Hz, 2H), 7.53 – 7.42 (m, 3H), 6.20 (ddt, *J* = 17.4, 13.8, 11.0 Hz, 1H), 5.84 – 5.71 (m, 1H), 5.55 (d, *J* = 11.1 Hz, 1H), 2.11 (td, *J* = 15.1, 7.7 Hz, 1H), 1.89 (dq, *J* = 14.8, 7.5 Hz, 1H), 1.34 (s, 3H), 0.79 (t, *J* = 7.5 Hz, 3H). ^{13}C NMR (101 MHz, $CDCl_3$) δ 151.90, δ130.99 (t, *J* = 25.7 Hz), 130.72, 128.96, 122.32, 121.83 (dd, *J* = 250.7, 243.9 Hz), 120.36 (t, *J* = 9.9 Hz), 77.09 (d, *J* = 46.9 Hz), 25.95, 14.01 (t, *J* = 3.3 Hz), 7.41. ^{19}F NMR (376 MHz, $CDCl_3$) δ -106.48 (dd, *J* = 245.8, 10.7 Hz), -110.37 (dd, *J* = 245.8, 13.8 Hz). IR (film): v 3066, 2975, 2943, 2884, 1527, 1455, 1417, 1375, 1310, 1225, 1143, 1086, 988, 948, 762, 687. ESI-MS *m/z* (rel): 239.1 $(M+H)^+$; HRMS (ESI) Calcd. for $C_{13}H_{17}N_2F_2$ $(M+H)^+$: 239.1354; Found: 239.1355. HPLC (Chiralpak OJ-H, Hexane : *i*-Propanol = 98 : 2, 0.7 mL/min, 214 nm): t_{major} = 6.14 min, t_{minor} = 6.61 min.

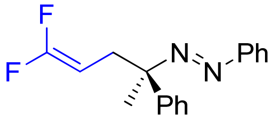

4a

Yellow oil; Yield: 90%; ^{1}H NMR (400 MHz, $CDCl_3$) δ 7.91 – 7.83 (m, 2H), 7.60 – 7.51 (m, 5H), 7.48 (t, *J* = 7.6 Hz, 2H), 7.41 – 7.35 (m, 1H), 4.14 (dtd, *J* = 25.2, 8.0, 2.5 Hz, 1H), 3.00 – 2.85 (m, 2H), 1.77 (s, 3H). ^{13}C NMR (101 MHz, $CDCl_3$) δ155.90 (dd, *J* = 288.1, 285.4 Hz), 151.05, 142.78, 129.69, 128.09, 127.50, 126.15, 125.67, 121.46, 73.84 (t, *J* = 2.1 Hz),, 73.35 (dd, *J* = 24.3, 19.6 Hz), 32.90 (d, *J* = 4.4 Hz), 22.24. ^{19}F NMR (376 MHz, $CDCl_3$) δ -87.08 (d, *J* = 43.8 Hz), -90.13 (dd, *J* = 43.8, 16.0 Hz). HRMS (ESI) Calcd. for $C_{17}H_{17}F_2N_2$ $(M+H)^+$: 287.1354; Found: 287.1354.

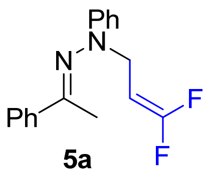

5a

Yellow oil; ^{1}H NMR (400 MHz, $CDCl_3$) δ 7.94 (d, J = 3.7 Hz, 2H), 7.48 (d, J = 2.4 Hz, 3H), 7.31 (dd, J = 11.3, 4.0 Hz, 2H), 7.00 (dd, J = 9.4, 4.1 Hz, 3H), 4.62 (dt, J = 26.9, 7.4 Hz, 1H), 4.21 (d, J = 7.4 Hz, 2H), 2.22 (s, 3H). ^{13}C NMR (101 MHz, $CDCl_3$) δ 171.17, 162.56 (t, J = 288.4 Hz), 155.23, 143.56, 135.25, 134.47, 133.78, 132.01, 127.02, 123.75, 80.79 (dd, J = 21.2, 19.3 Hz), 56.38 (d, J = 6.2 Hz), 22.13. ^{19}F NMR (376 MHz, $CDCl_3$) δ -86.30 (d, J = 27.9 Hz), -87.87 (dd, J = 41.1, 25.4 Hz). HRMS (ESI) Calcd. for $C_{17}H_{17}F_2N_2$ $(M+H)^+$: 287.1354; Found: 287.1353.

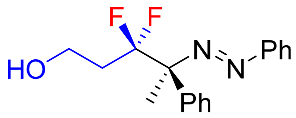

6

Yellow oil; Yield: 66%; er: 95:5; $[\alpha]_D^{20}$ = -175.64 (c 1.4, $CHCl_3$); ^{1}H NMR (400 MHz, $CDCl_3$) δ 7.89 – 7.76 (m, 2H), 7.57 – 7.48 (m, 3H), 7.41 (d, J = 7.5 Hz, 2H), 7.34 (td, J = 8.5, 4.5 Hz, 3H), 3.98 – 3.85 (m, 2H), 2.63 (ddt, J = 21.2, 13.8, 6.8 Hz, 1H), 2.27 (ddt, J = 21.5, 13.3, 6.5 Hz, 1H), 1.89 (s, 1H), 1.76 (s, 3H). ^{13}C NMR (101 MHz, $CDCl_3$) δ 151.84, 140.82, 140.78, 131.38, 129.15, 128.25, 127.76, 127.70, 122.59, 78.83 (t, J = 22.8 Hz), 56.85 (t, J = 5.3 Hz), 36.39 (t, J = 22.9 Hz), 29.71, 17.66 (d, J = 3.6 Hz). ^{19}F NMR (376 MHz, $CDCl_3$) δ -104.34 (ddd, J = 248.5, 31.2, 6.6 Hz, 1H), -108.56 (ddd, J = 248.6, 30.2, 7.3 Hz, 1H). IR (film): ν 3353, 2949, 1494, 1478, 1449, 1393, 1373, 1196, 1151, 1066, 1046, 967, 924, 763, 688. ESI-MS m/z (rel): 305.1 $(M+H)^+$; HRMS (ESI) Calcd. for $C_{17}H_{19}ON_2F_2$ $(M+H)^+$: 305.1460; Found: 305.1456. HPLC (PC-3 (Phenomenex Cellulose-3), CH_3CN : H_2O = 50 : 50, 0.7 mL/min, 214 nm): t_{minor} = 10.78 min, t_{major} = 11.51 min.

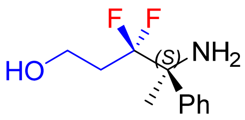

7

White solid; Yield: 78%; er: 95:5; $[\alpha]_D^{22}$ = -16.13 (*c* 1.0, $CHCl_3$); ^1H NMR (400 MHz, $CDCl_3$) δ 7.53 (d, *J* = 7.9 Hz, 2H), 7.33 (ddd, *J* = 11.1, 9.5, 5.9 Hz, 3H), 3.77 (ddd, *J* = 11.8, 8.4, 3.3 Hz, 1H), 3.70 – 3.60 (m, 1H), 2.85 (s, 3H), 2.12 – 1.86 (m, 2H), 1.71 (s, 3H). ^{13}C NMR (101 MHz, $CDCl_3$) δ 141.20, 128.30, 127.73, 126.72, 109.99, 59.72 – 59.20 (m), 56.25 – 56.02 (m), 36.76 (t, *J* = 25.2 Hz), 25.79. ^{19}F NMR (376 MHz, $CDCl_3$) δ -100.55 (d, *J* = 250.8 Hz), -110.12 (d, *J* = 252.4 Hz). IR (film): v 3330, 3286, 3064, 2920, 2850, 1601, 1498, 1469, 1371, 1188, 1123, 1077, 1042, 988, 963, 908, 772, 695. ESI-MS *m/z* (rel): 216.1 $(M+H)^+$; HRMS (ESI) Calcd. for $C_{11}H_{16}ONF_2$ $(M+H)^+$: 216.1194; Found: 216.1194. HPLC (Chiralpak OD-H, Hexane : *i*-Propanol = 90 : 10, 0.8 mL/min, 210 nm): t_{major} = 10.78 min, t_{minor} = 13.99 min.

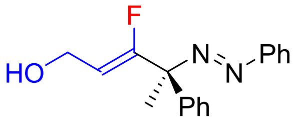

8

Yellow oil; Yiled: 81%; er: 94:6; $[\alpha]_D^{20}$ = -95.64 (*c* 0.7, $CHCl_3$); ^1H NMR (400 MHz, $CDCl_3$) δ 7.85 – 7.72 (m, 2H), 7.50 (dd, *J* = 12.6, 6.8 Hz, 5H), 7.40 (t, *J* = 7.4 Hz, 2H), 7.33 (t, *J* = 7.2 Hz, 1H), 5.31 (dt, *J* = 36.8, 6.9 Hz, 1H), 4.41 – 4.35 (m, 2H), 1.82 (s, 3H), 1.62 (s, 1H). ^{13}C NMR (101 MHz, $CDCl_3$) δ 161.62 (d, *J* = 262.4 Hz), 151.89, 140.94, 131.21, 129.18, 128.61, 127.86, 127.35, 122.72, 108.89 (d, *J* = 11.5 Hz), 77.29 (d, *J* = 23.8 Hz), 55.90 (d, *J* = 8.2 Hz), 22.60. ^{19}F NMR (376 MHz, $CDCl_3$) δ -111.10 (d, *J* = 36.9 Hz). ESI-MS *m/z* (rel): 285.1 $(M+H)^+$; HRMS (ESI) Calcd. for $C_{17}H_{18}ON_2F$ $(M+H)^+$: 285.1398; Found: 285.1396. HPLC (IG-3, CO_2 : MeOH = 90 : 10, 1.0 mL/min, 214 nm): t_{major} = 12.02 min, t_{minor} = 12.69 min.

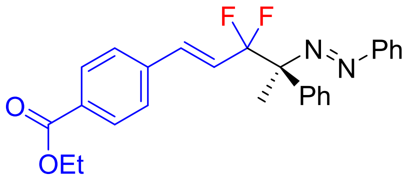

9

Yellow oil; Yiled: 65%; er: 95:5; $[\alpha]_D^{20}$ = -30.11, (*c* 0.9, $CHCl_3$); ^1H NMR (400 MHz, $CDCl_3$) δ 8.00 (d, *J* = 8.2 Hz, 2 H), 7.85 – 7.76 (m, 2 H), 7.52 – 7.43 (m, 5 H), 7.39 (d, *J* = 8.2 Hz, 2 H), 7.36 – 7.29 (m, 3H), 6.85 (d, *J* = 16.1 Hz, 1 H), 6.48 (dt, *J* = 16.1, 12.1 Hz, 1 H), 4.37 (q, *J* = 7.1 Hz, 2 H), 1.80 (s, 3 H), 1.39 (t, *J* = 7.0 Hz, 3H).^{19}F NMR (376 MHz, $CDCl_3$) δ -108.76 (dd, *J* = 244.5, 12.4 Hz), -109.70 (dd, *J* = 243.9, 11.5 Hz). ^{13}C NMR (101 MHz, $CDCl_3$) δ 166.1, 151.9, 140.2, 139.3, 134.3 (t, *J* = 9.2 Hz), 131.3, 130.5, 129.9, 129.1, 128.2, 128.0, 127.8, 127.0, 124.0(t, *J* = 25.0 Hz), 122.6, 121.3 (t, *J* = 249.3 Hz), 79.3 (t, *J* = 24.4 Hz), 61.1, 17.7, 14.3. ESI-MS *m/z* (rel): 435.2 $(M+H)^+$; HRMS (ESI) Calcd. for $C_{26}H_{25}O_2N_2F_2$ $(M+H)^+$: 435.1879; Found: 435.1875. HPLC (PC-3, CH_3CN : H_2O = 70 :30, 0.7 mL/min, 214nm) t_{minor} = 16.75 min, t_{major}= 22.08 min.

5. Supplementary Figures - NMR and HPLC spectra

Supplementary Figure 9 ^{1}H NMR (400 MHz, $CDCl_3$) of **3a**

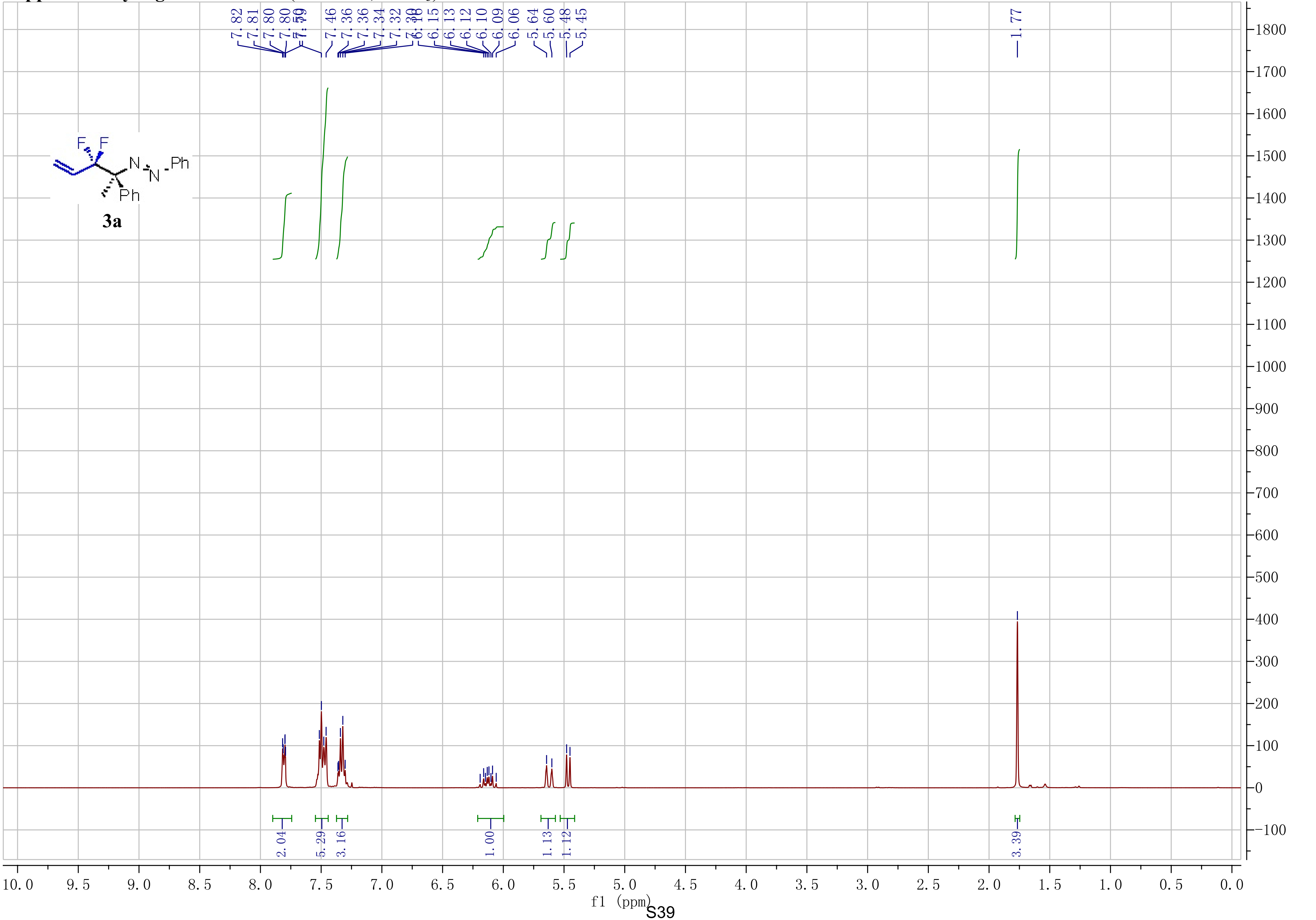

Supplementary Figure 10 ^{13}C NMR (101 MHz, $CDCl_3$) of **3a**

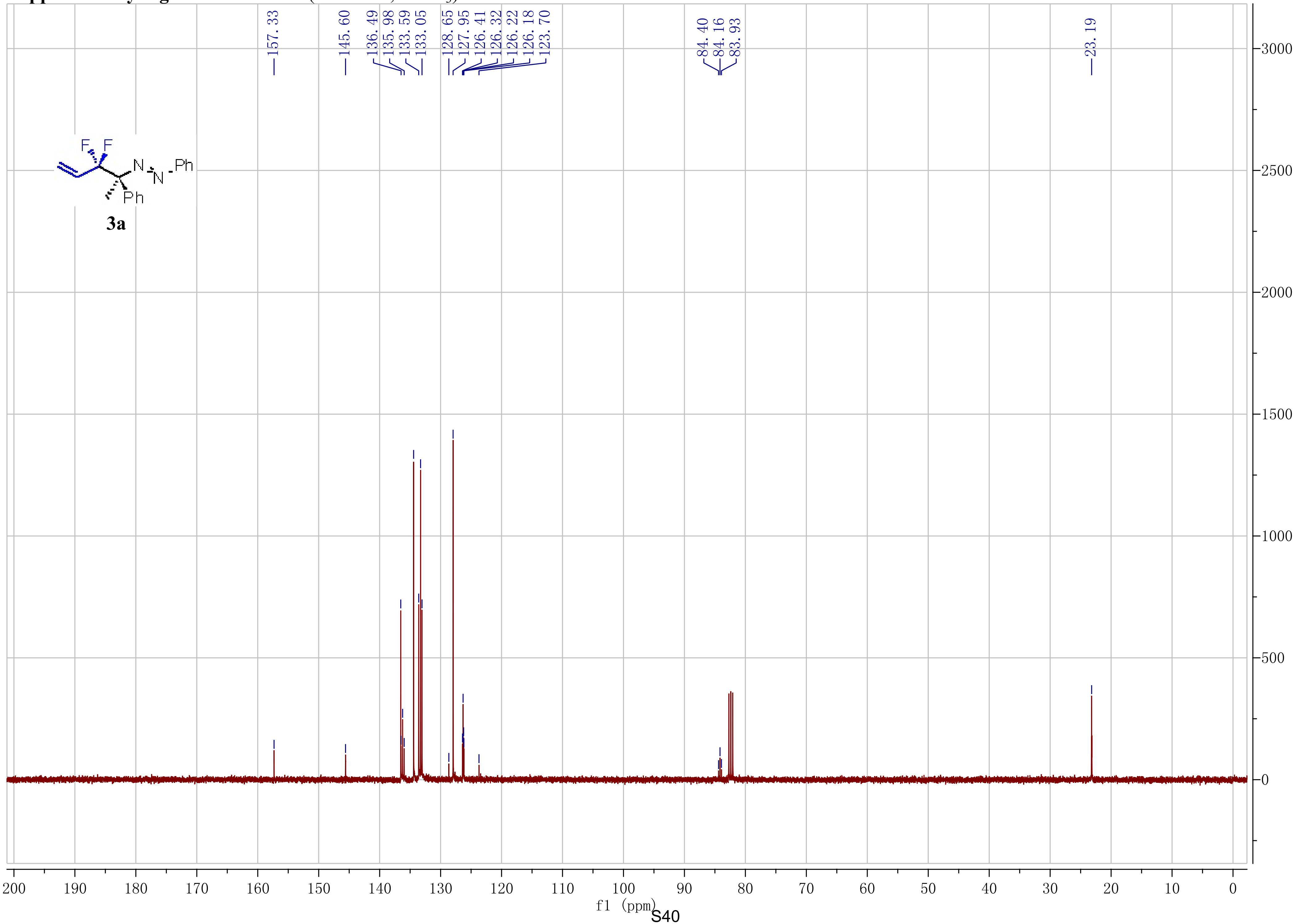

Supplementary Figure 11 ^{19}F NMR (376 MHz, $CDCl_3$) of **3a**

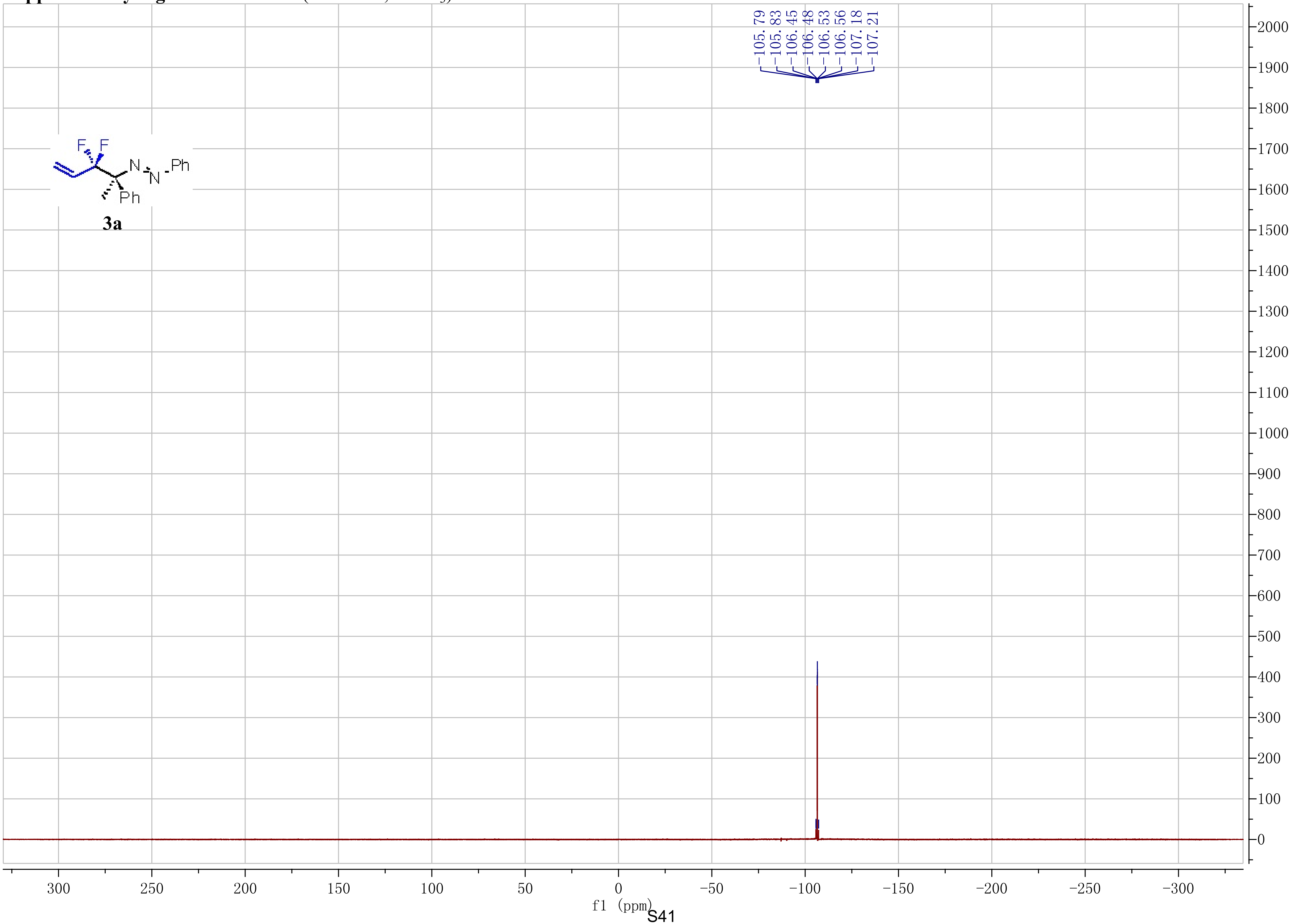

Supplementary Figure 12 HPLC spectra of racemic **3a**

Chromatogram and Results

Injection Details

Injection Name:	**HS-11-74-1+- PC3 A62W38 214 0.7**	*Run Time (min):*	**30.00**
Vial Number:	**RD1**	*Injection Volume:*	**5.00**
Injection Type:	**Unknown**	*Channel:*	**UV_VIS_1**
Calibration Level:		*Wavelength:*	**214.0**
Instrument Method:	**20160223-DAD3**	*Bandwidth:*	**4**
Processing Method:	**20160223**	*Dilution Factor:*	**1.0000**
Injection Date/Time:	**22/04/20 15:20**	*Sample Weight:*	**1.0000**

Chromatogram

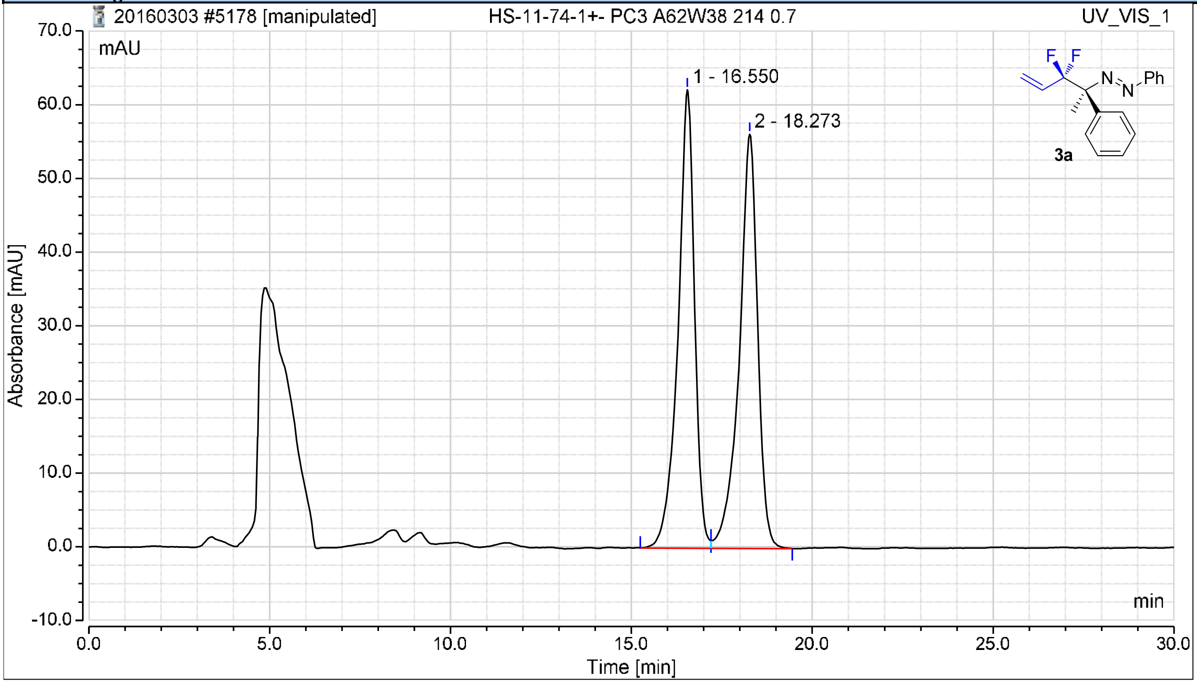

Integration Results

No.	Retention Time min	Area mAU*min	Height mAU	Relative Area %
1	16.550	32.8890	62.2097	49.840
2	18.273	33.1007	56.2166	50.160
**Total:**		**65.990**	**1401.998**	**100.000**

Supplementary Figure 13 HPLC spectra of (*S*)-**3a**

Chromatogram and Results

Injection Details

Injection Name:	**HS-13-51-1 PC3 A62W38 214 0.7**	*Run Time (min):*	**30.00**
Vial Number:	**RD5**	*Injection Volume:*	**5.00**
Injection Type:	**Unknown**	*Channel:*	**UV_VIS_1**
Calibration Level:		*Wavelength:*	**214.0**
Instrument Method:	**20160223-DAD3**	*Bandwidth:*	**4**
Processing Method:	**20160223**	*Dilution Factor:*	**1.0000**
Injection Date/Time:	**22/04/20 17:26**	*Sample Weight:*	**1.0000**

Chromatogram

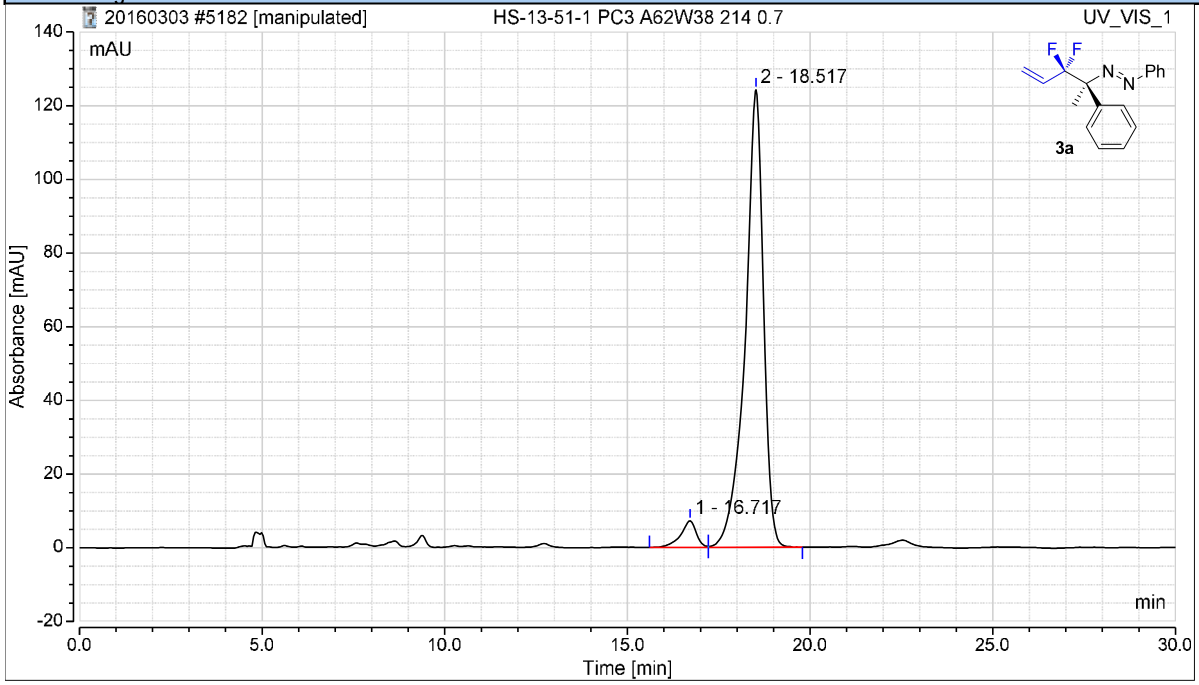

Integration Results

No.	Retention Time min	Area mAU*min	Height mAU	Relative Area %
1	16.717	3.6300	7.2261	4.834
2	18.517	71.4610	124.1597	95.166
**Total:**		**75.091**	**1401.998**	**100.000**

Supplementary Figure 14 ^{1}H NMR (400 MHz, $CDCl_3$) of **3b**

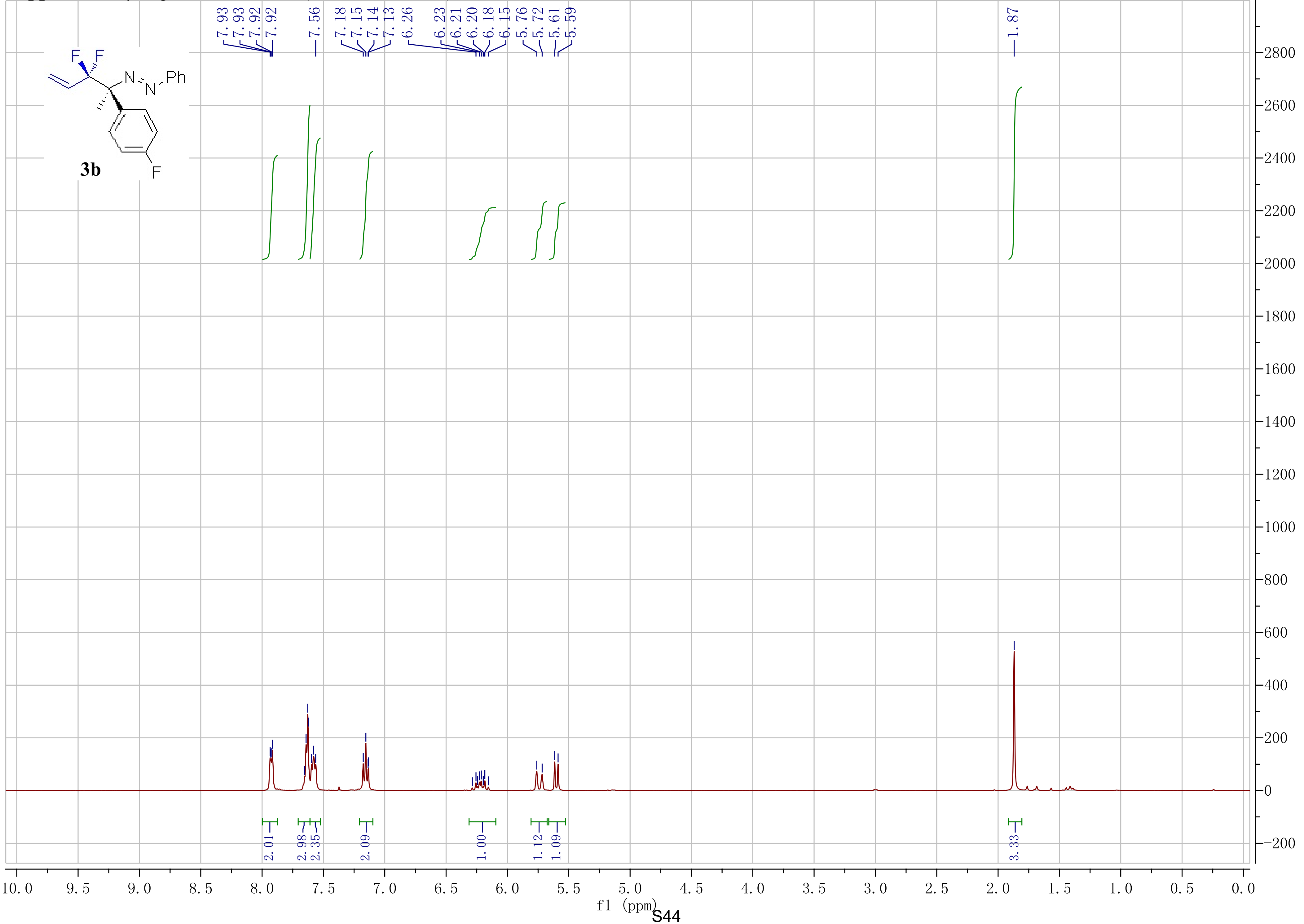

Supplementary Figure 15 ^{13}C NMR (101 MHz, $CDCl_3$) of **3b**

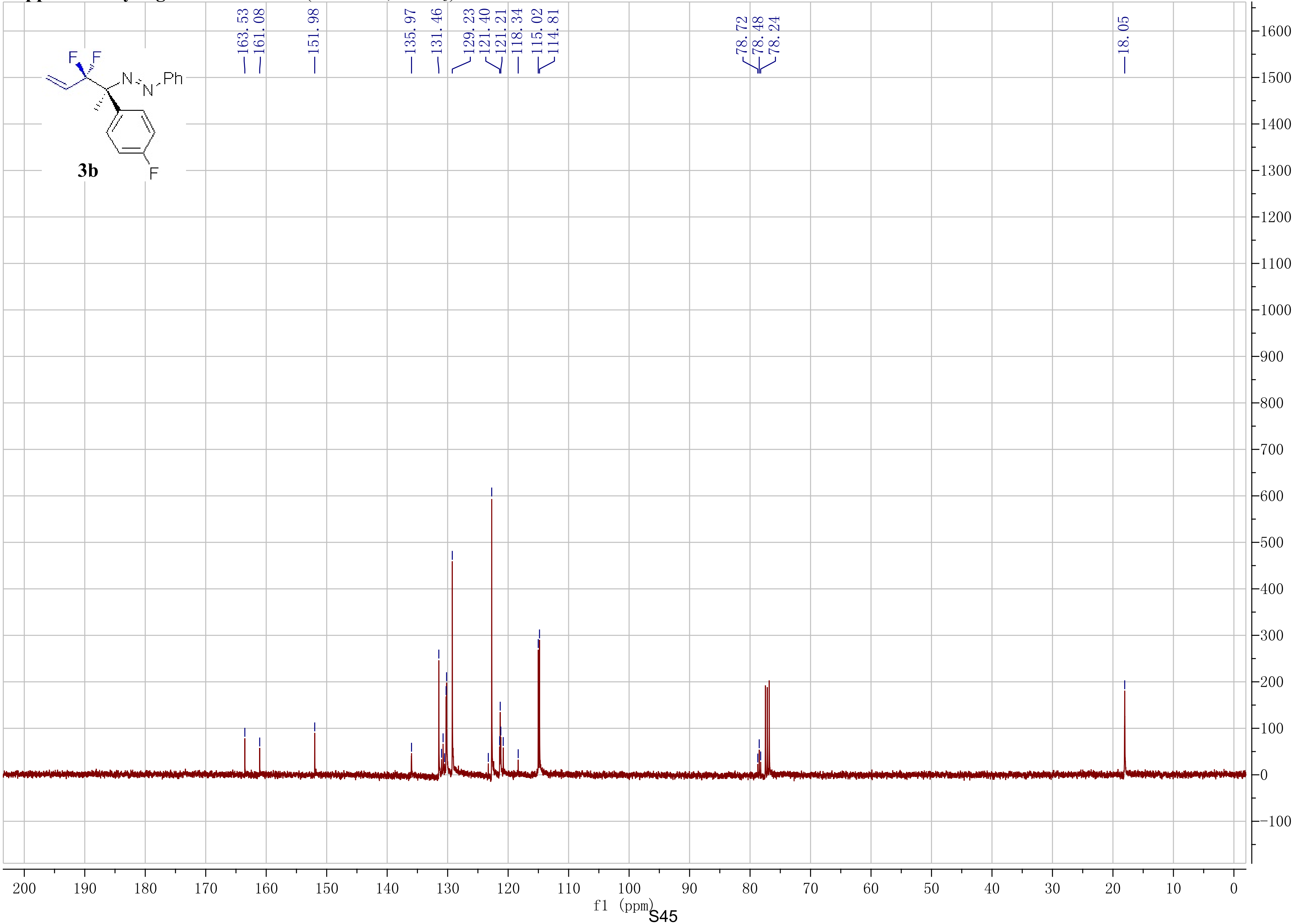

Supplementary Figure 16 ^{19}F NMR (376 MHz, $CDCl_3$) of **3b**

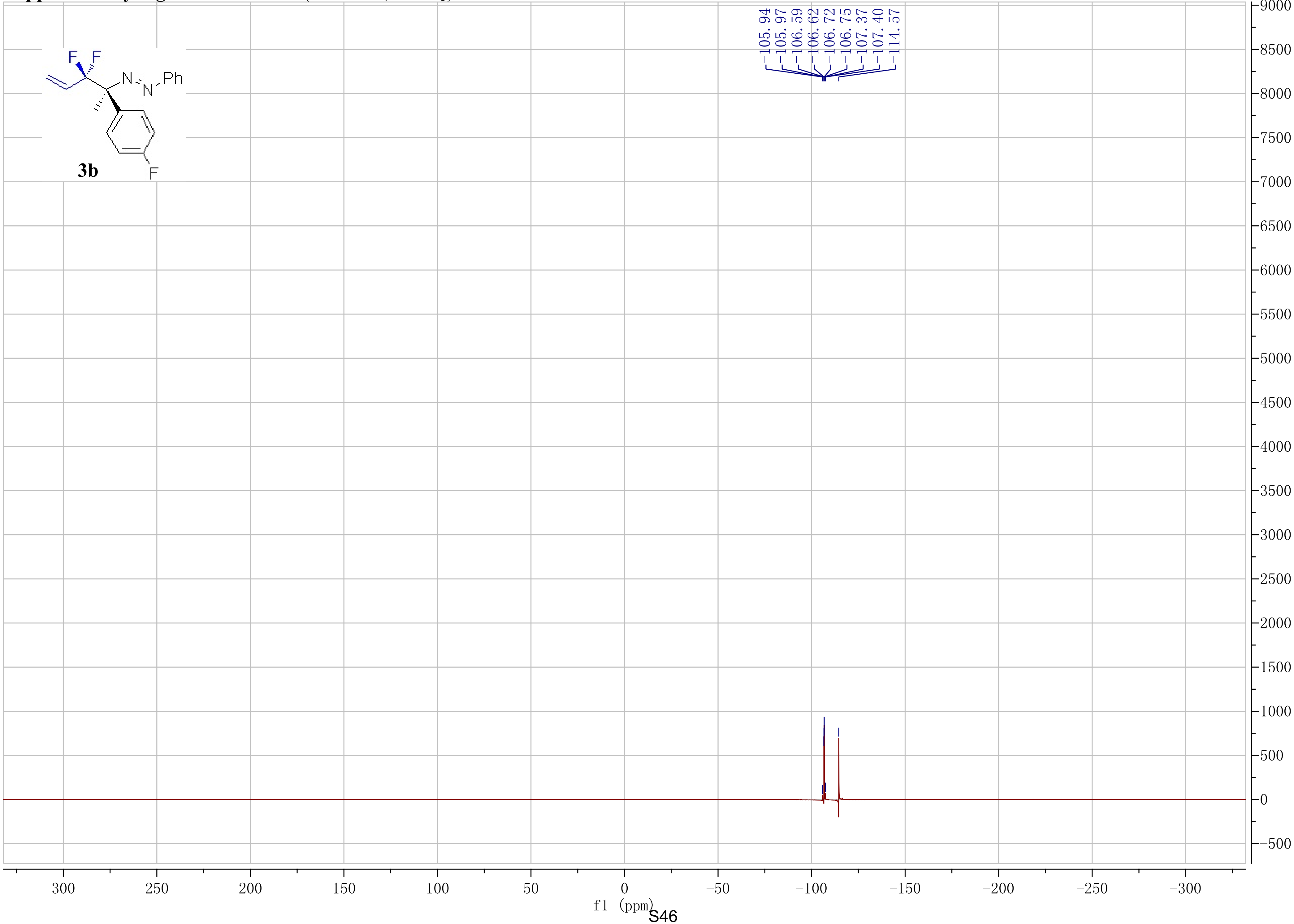

Supplementary Figure 17 HPLC spectra of racemic **3b**

Chromatogram and Results

Injection Details

Injection Name:	**HS-13-46-4 PC3 A7W3 214 0.7**	*Run Time (min):*	**26.03**
Vial Number:	**RD3**	*Injection Volume:*	**5.00**
Injection Type:	**Unknown**	*Channel:*	**UV_VIS_1**
Calibration Level:		*Wavelength:*	**214.0**
Instrument Method:	**20160223-DAD3**	*Bandwidth:*	**4**
Processing Method:	**20160223**	*Dilution Factor:*	**1.0000**
Injection Date/Time:	**27/04/20 14:12**	*Sample Weight:*	**1.0000**

Chromatogram

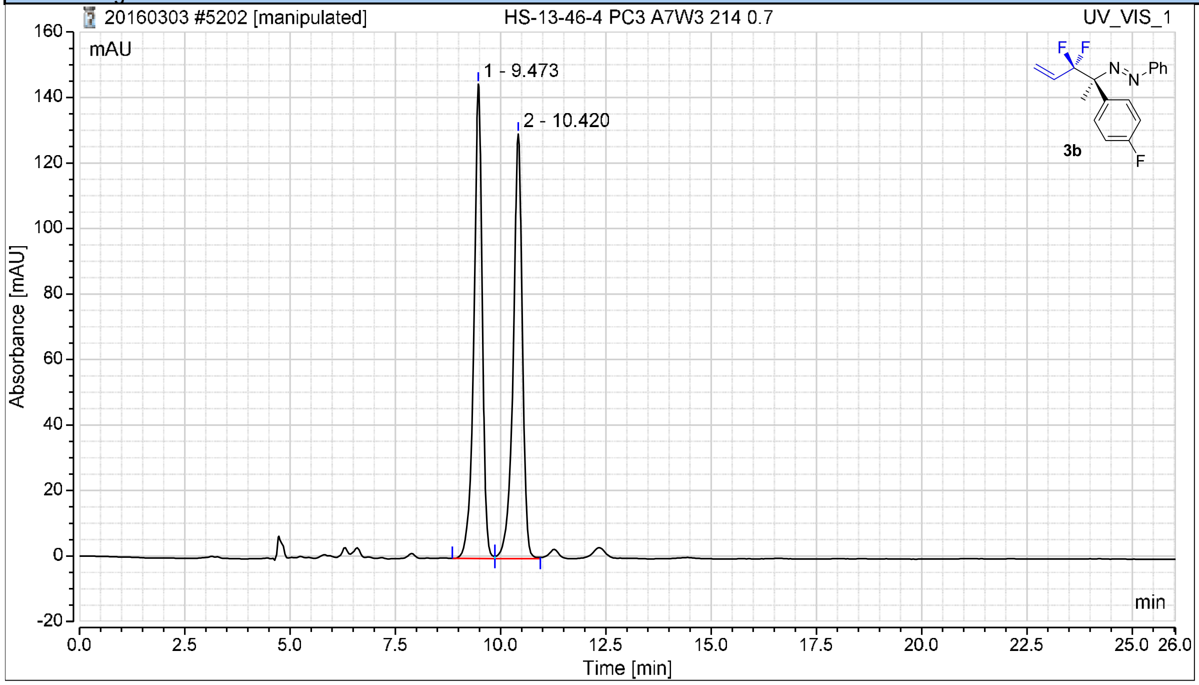

Integration Results

No.	Retention Time min	Area mAU*min	Height mAU	Relative Area %
1	9.473	32.9815	144.8647	50.114
2	10.420	32.8309	129.6526	49.886
**Total:**		**65.812**	**1401.998**	**100.000**

Supplementary Figure 18 HPLC spectra of (*S*)-**3b**

Chromatogram and Results

Injection Details

Injection Name:	**HS-13-56-1 PC3 A7W3 214 0.7**	*Run Time (min):*	**15.19**
Vial Number:	**RD4**	*Injection Volume:*	**2.00**
Injection Type:	**Unknown**	*Channel:*	**UV_VIS_1**
Calibration Level:		*Wavelength:*	**214.0**
Instrument Method:	**20160223-DAD3**	*Bandwidth:*	**4**
Processing Method:	**20160223**	*Dilution Factor:*	**1.0000**
Injection Date/Time:	**27/04/20 14:54**	*Sample Weight:*	**1.0000**

Chromatogram

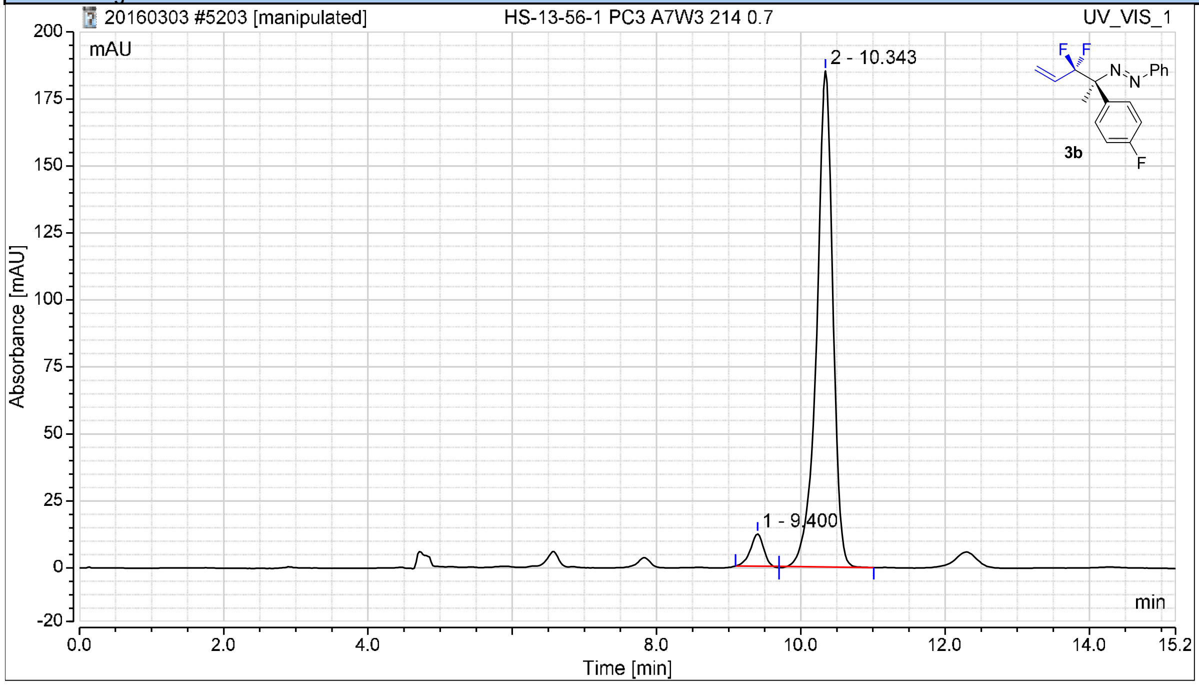

Integration Results

No.	Retention Time min	Area mAU*min	Height mAU	Relative Area %
1	9.400	2.5235	12.0563	5.011
2	10.343	47.8327	185.1531	94.989
**Total:**		**50.356**	**1401.998**	**100.000**

Supplementary Figure 19 ^{1}H NMR (400 MHz, $CDCl_3$) of **3c**

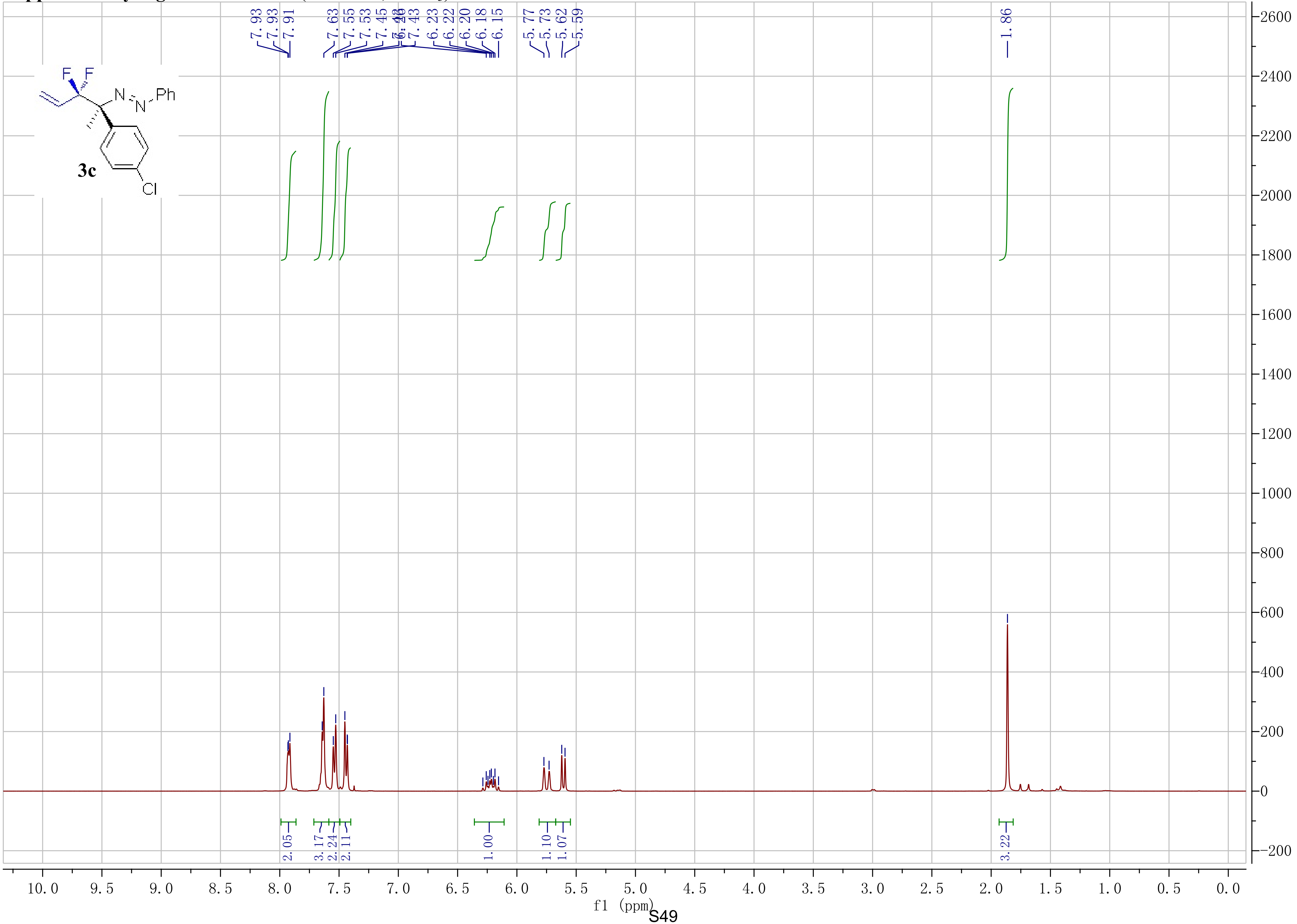

Supplementary Figure 20 ^{13}C NMR (101 MHz, $CDCl_3$) of **3c**

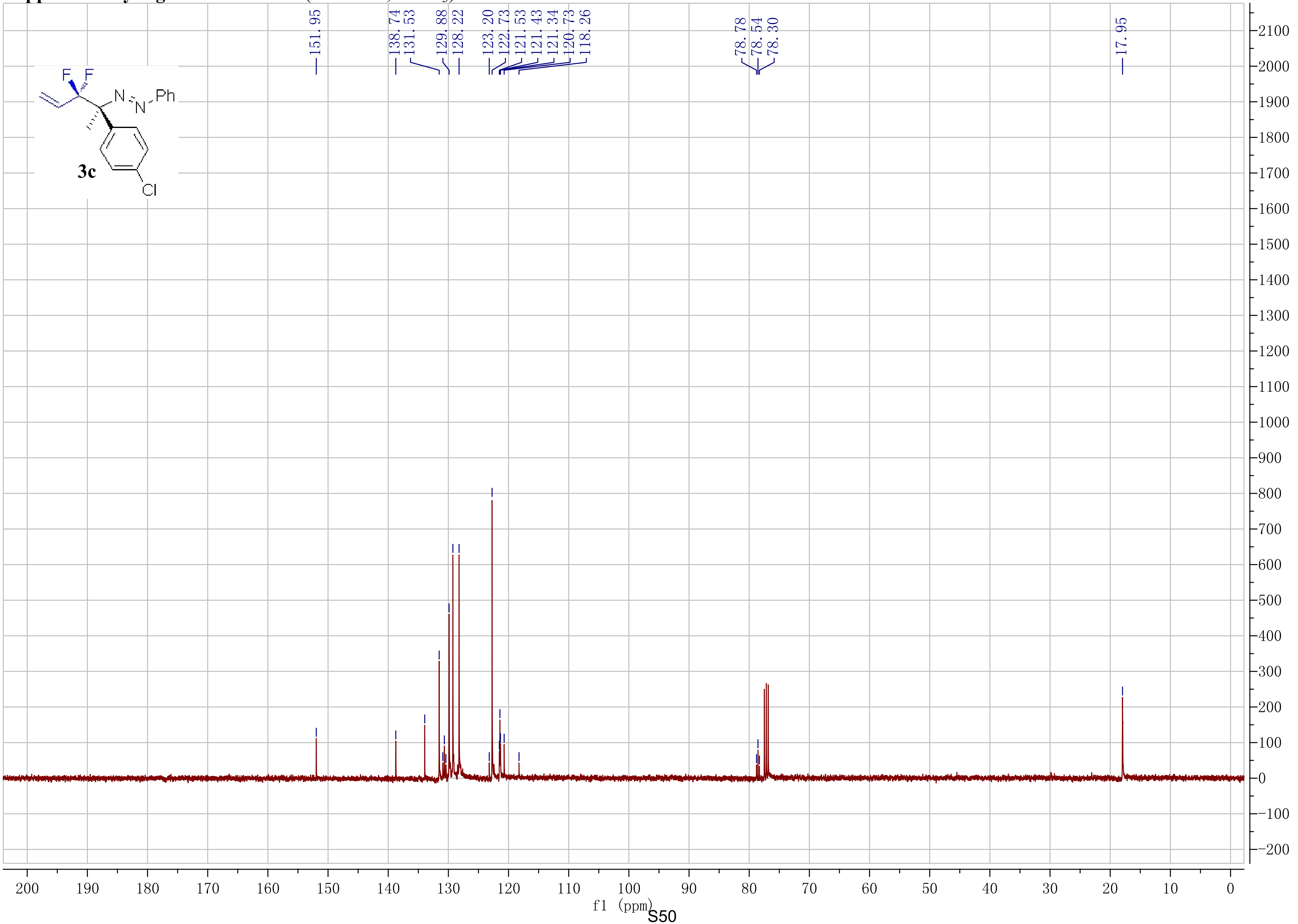

Supplementary Figure 21 ^{19}F NMR (376 MHz, $CDCl_3$) of **3c**

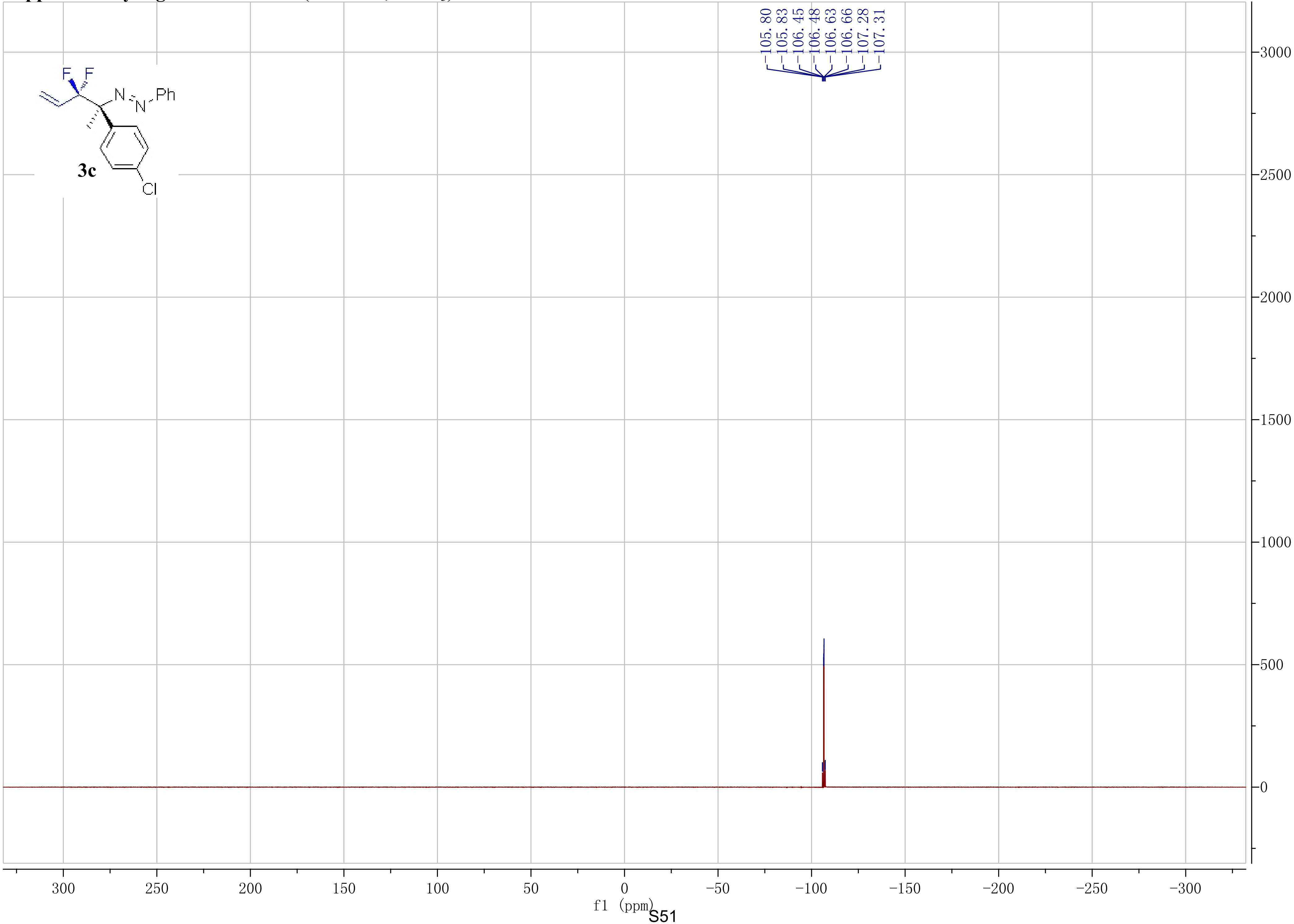

Supplementary Figure 22 HPLC spectra of racemic **3c**

Data File D:\CHEM32\1\DATA\2020\DEF_LC 2020-05-08 17-01-22\021-1101.D
Sample Name: 13-46-5+-

===

Acq. Operator : YZR Seq. Line : 11
Acq. Instrument : instr1 Location : Vial 21
Injection Date : 08/05/2020 22:09:57 Inj : 1
Inj Volume : 3.000 µl
Acq. Method : D:\CHEM32\1\DATA\2020\DEF_LC 2020-05-08 17-01-22\YZR3.M
Last changed : 08/05/2020 17:01:21 by YZR
Analysis Method : D:\CHEM32\1\METHODS\YZR3.M
Last changed : 08/05/2020 08:37:34 by YZR
(modified after loading)
Additional Info : Peak(s) manually integrated

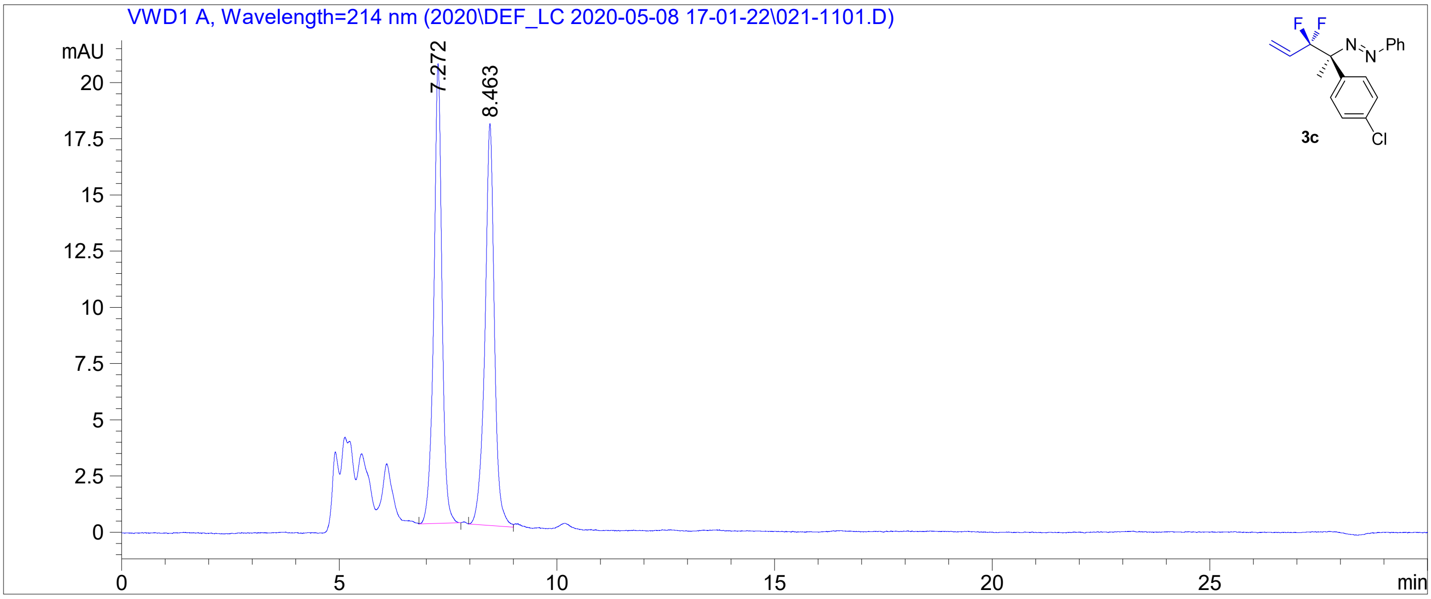

===

Area Percent Report

===

Sorted By : Signal
Multiplier: : 1.0000
Dilution: : 1.0000
Use Multiplier & Dilution Factor with ISTDs

Signal 1: VWD1 A, Wavelength=214 nm

Peak #	RetTime [min]	Type	Width [min]	Area [mAU*s]	Height [mAU]	Area %
1	7.272	BB	0.1912	266.61777	20.43679	49.6252
2	8.463	BV	0.2217	270.64520	17.87221	50.3748

Totals : 537.26297 38.30900

===

*** End of Report ***

Supplementary Figure 23 HPLC spectra of (*S*)-**3c**

Data File D:\CHEM32\1\DATA\2020\DEF_LC 2020-05-08 17-01-22\022-1201.D
Sample Name: 13-62-6

===

Acq. Operator : YZR | Seq. Line : 12
Acq. Instrument : instr1 | Location : Vial 22
Injection Date : 08/05/2020 22:40:44 | Inj : 1
Inj Volume : 3.000 µl
Acq. Method : D:\CHEM32\1\DATA\2020\DEF_LC 2020-05-08 17-01-22\YZR3.M
Last changed : 08/05/2020 17:01:21 by YZR
Analysis Method : D:\CHEM32\1\METHODS\YZR3.M
Last changed : 08/05/2020 08:37:34 by YZR
(modified after loading)
Additional Info : Peak(s) manually integrated

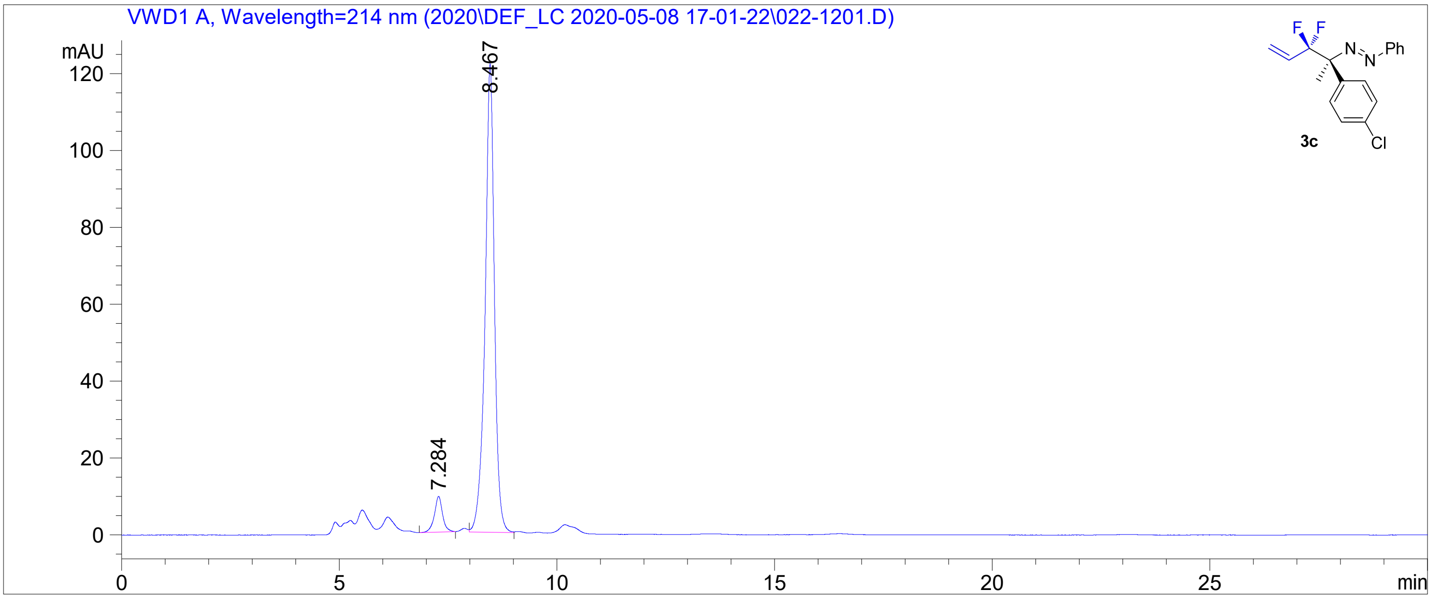

===

Area Percent Report

===

Sorted By : Signal
Multiplier: : 1.0000
Dilution: : 1.0000
Use Multiplier & Dilution Factor with ISTDs

Signal 1: VWD1 A, Wavelength=214 nm

Peak #	RetTime [min]	Type	Width [min]	Area [mAU*s]	Height [mAU]	Area %
1	7.284	BB	0.1937	121.18623	9.31700	6.2586
2	8.467	VV	0.2188	1815.11487	121.86586	93.7414

Totals : 1936.30109 131.18286

===

*** End of Report ***

Supplementary Figure 24 ^{1}H NMR (400 MHz, $CDCl_3$) of **3d**

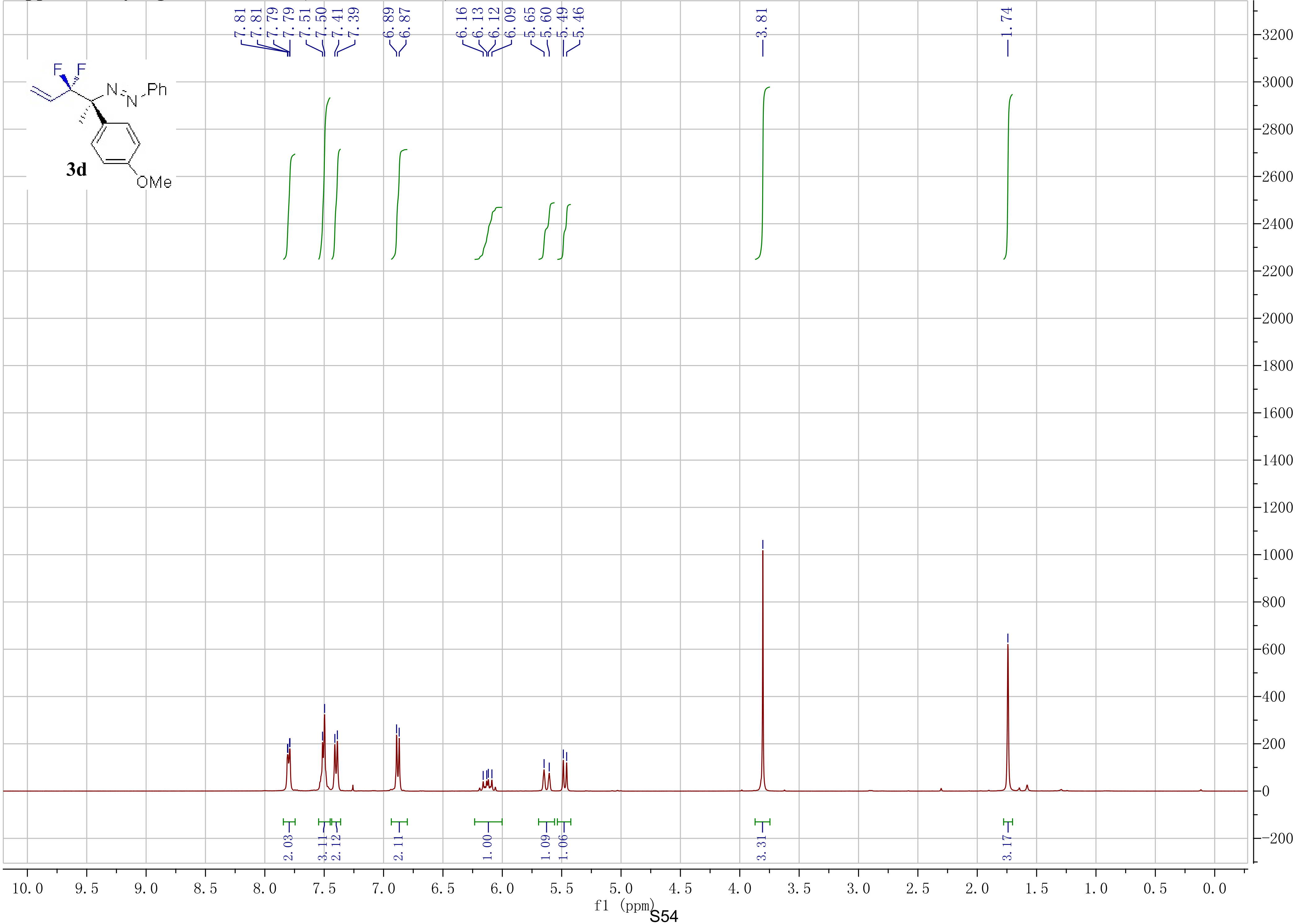

Supplementary Figure 25 ^{13}C NMR (101 MHz, $CDCl_3$) of **3d**

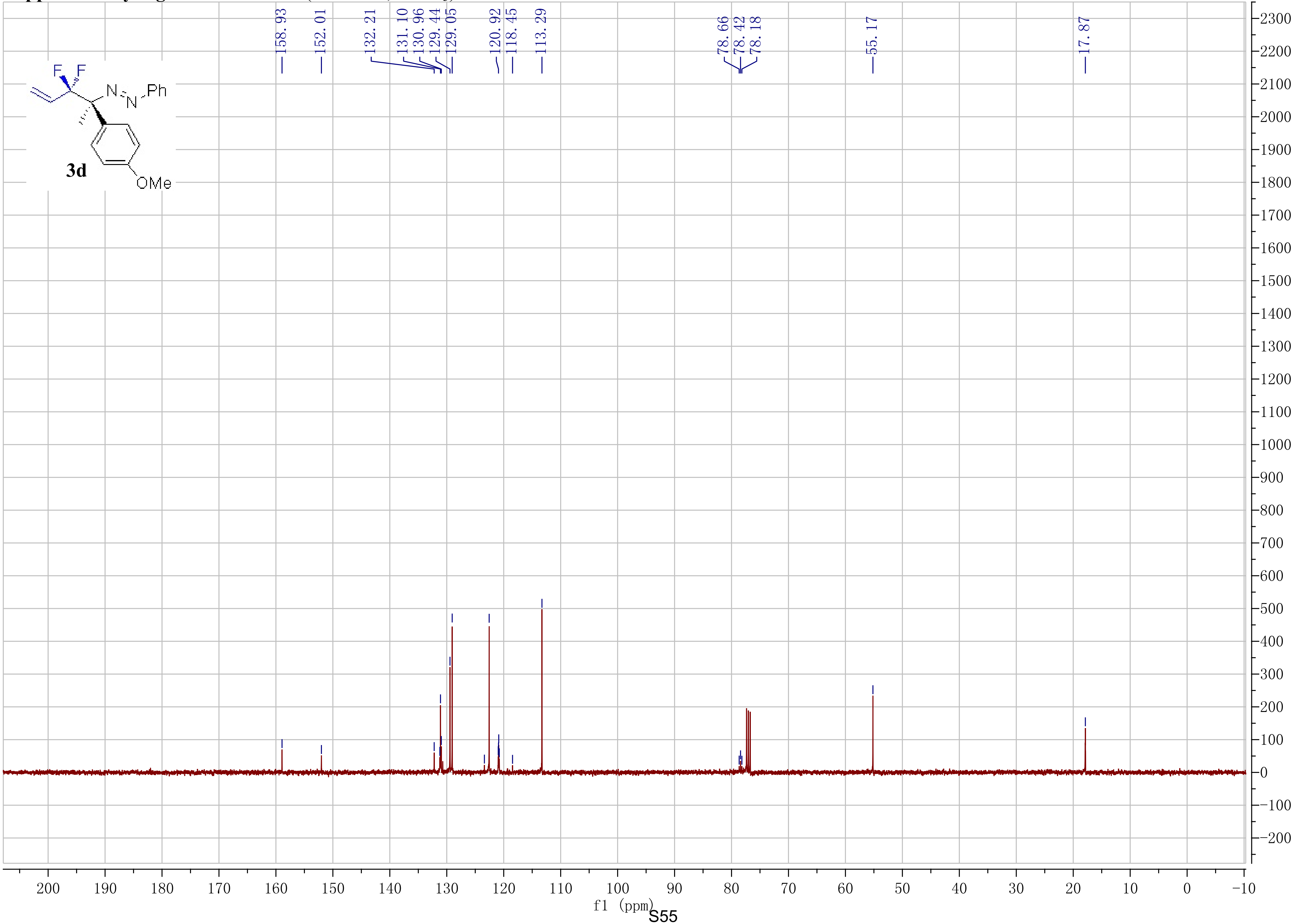

Supplementary Figure 26 ^{19}F NMR (376 MHz, $CDCl_3$) of **3d**

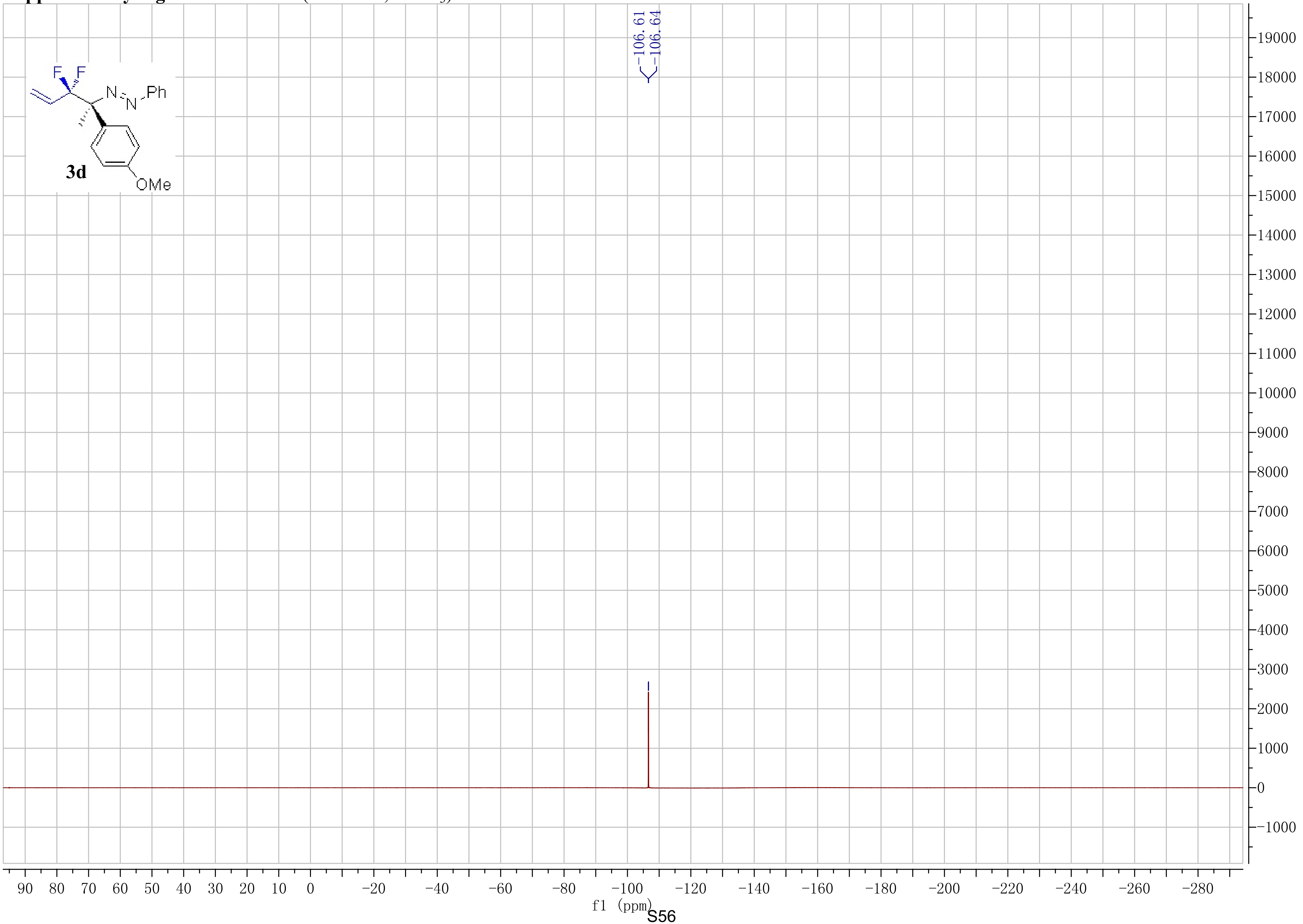

Supplementary Figure 27 HPLC spectra of racemic **3d**

Chromatogram and Results

Injection Details

Injection Name:	**HS-13-38-4+- PC3 A7W3 214 0.7**	*Run Time (min):*	**35.31**
Vial Number:	**RD5**	*Injection Volume:*	**2.00**
Injection Type:	**Unknown**	*Channel:*	**UV_VIS_1**
Calibration Level:		*Wavelength:*	**214.0**
Instrument Method:	**20160223-DAD3**	*Bandwidth:*	**4**
Processing Method:	**20160223**	*Dilution Factor:*	**1.0000**
Injection Date/Time:	**27/04/20 15:13**	*Sample Weight:*	**1.0000**

Chromatogram

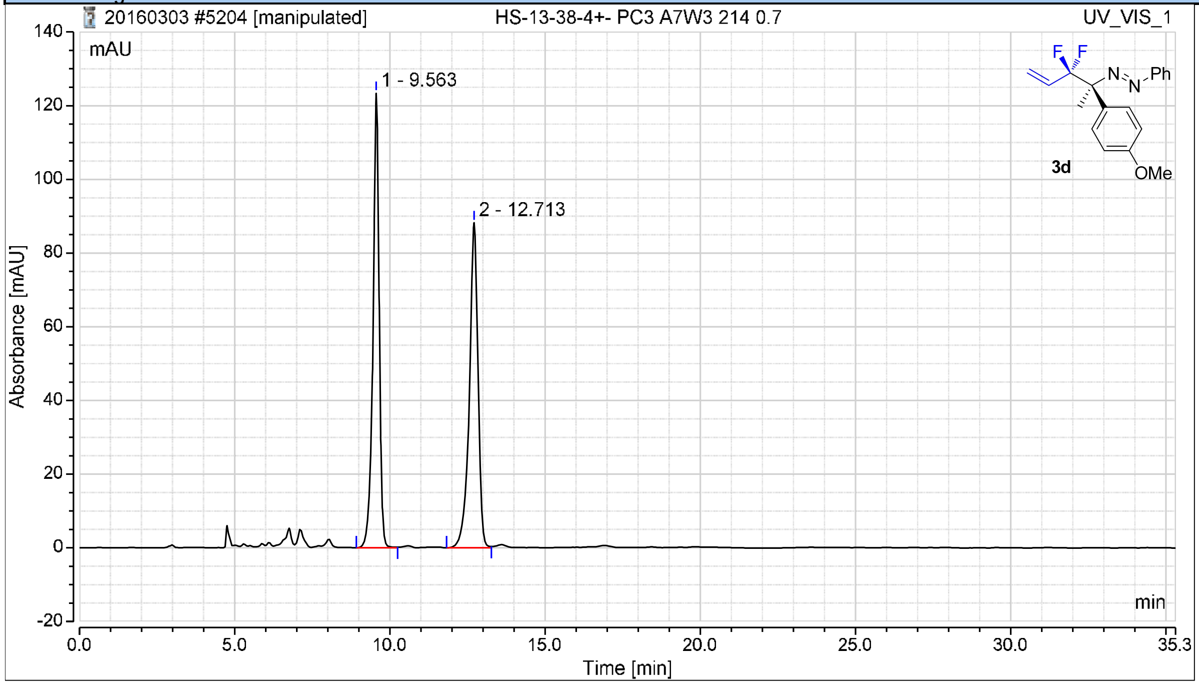

Integration Results

No.	Retention Time min	Area mAU*min	Height mAU	Relative Area %
1	9.563	28.5154	123.3313	49.931
2	12.713	28.5946	88.1553	50.069
**Total:**		**57.110**	**1401.998**	**100.000**

Supplementary Figure 28 HPLC spectra of (*S*)-**3d**

Chromatogram and Results

Injection Details

Injection Name:	**HS-13-56-2 PC3 A7W3 214 0.7**	*Run Time (min):*	**30.61**
Vial Number:	**RD6**	*Injection Volume:*	**2.00**
Injection Type:	**Unknown**	*Channel:*	**UV_VIS_1**
Calibration Level:		*Wavelength:*	**214.0**
Instrument Method:	**20160223-DAD3**	*Bandwidth:*	**4**
Processing Method:	**20160223**	*Dilution Factor:*	**1.0000**
Injection Date/Time:	**27/04/20 15:52**	*Sample Weight:*	**1.0000**

Chromatogram

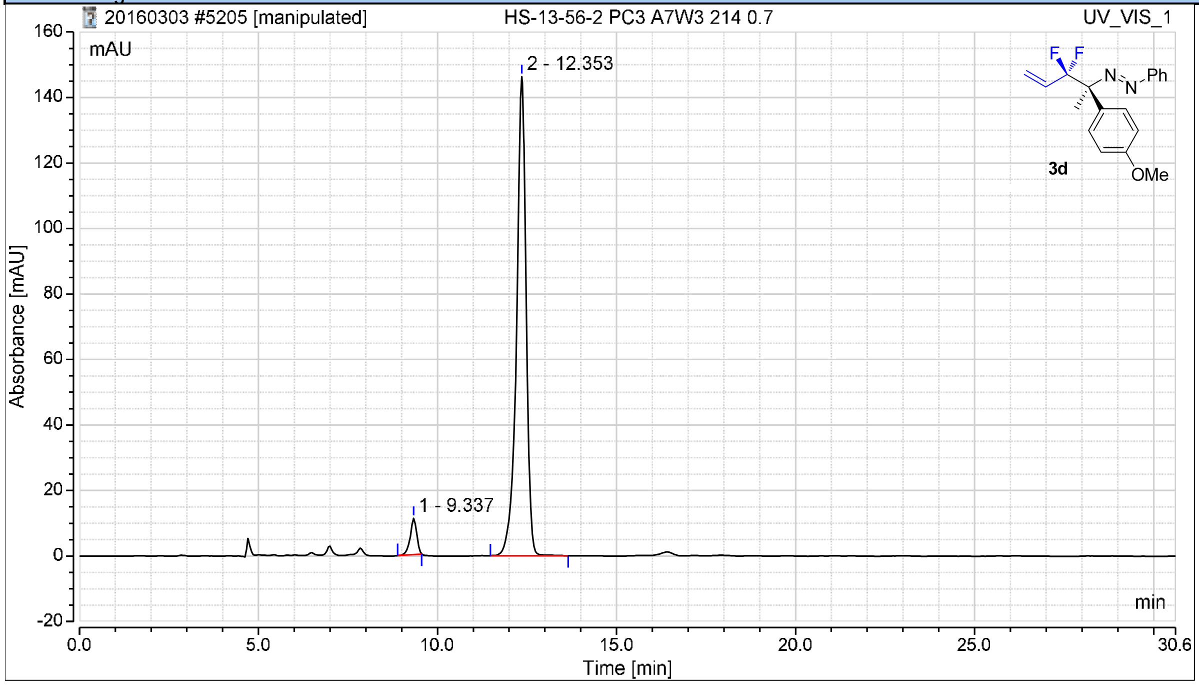

Integration Results

No.	Retention Time min	Area mAU*min	Height mAU	Relative Area %
1	9.337	2.3211	11.0488	4.876
2	12.353	45.2822	146.2910	95.124
**Total:**		**47.603**	**1401.998**	**100.000**

Supplementary Figure 29 ^{1}H NMR (400 MHz, $CDCl_3$) of **3e**

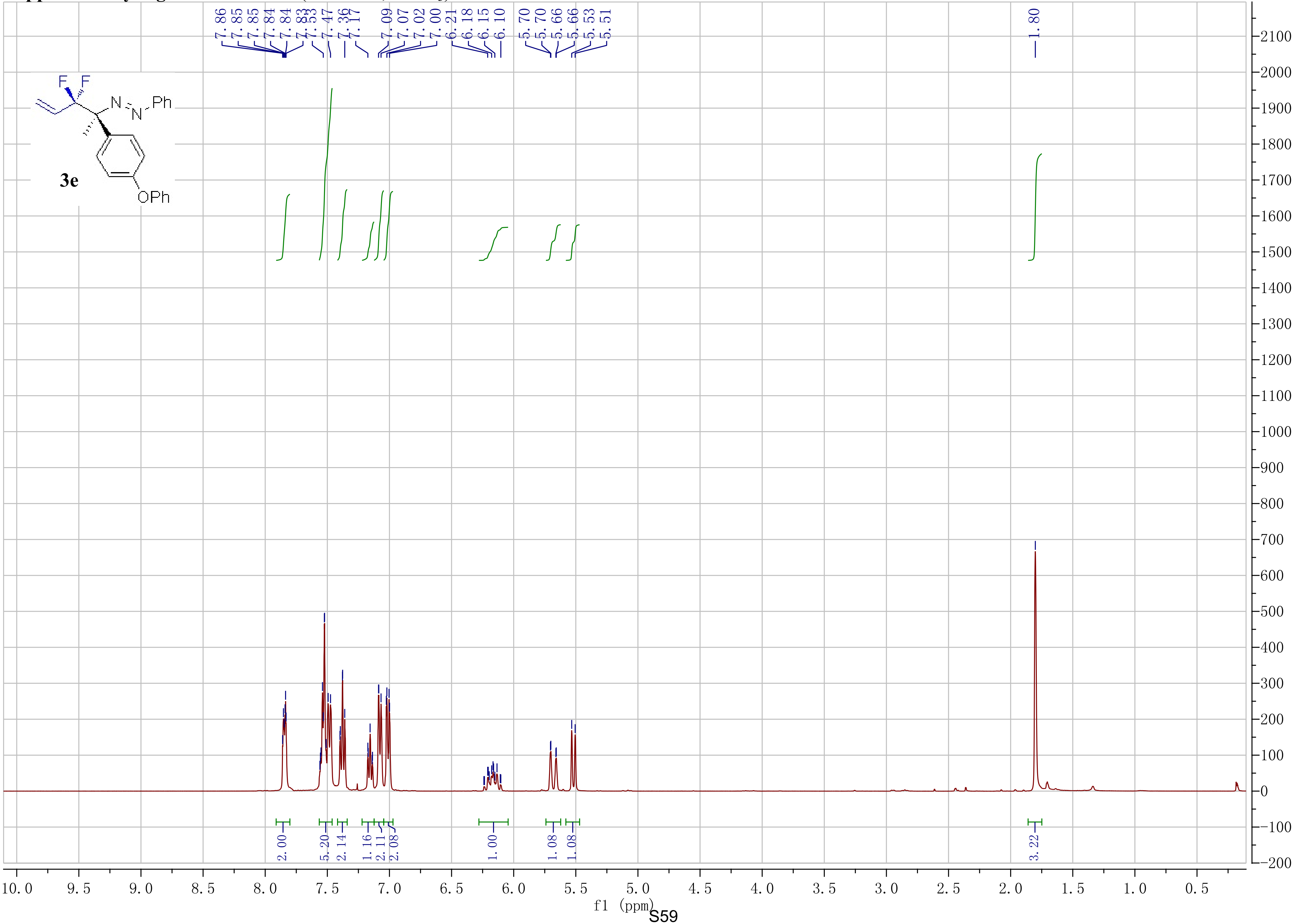

Supplementary Figure 30 ^{13}C NMR (101 MHz, $CDCl_3$) of **3e**

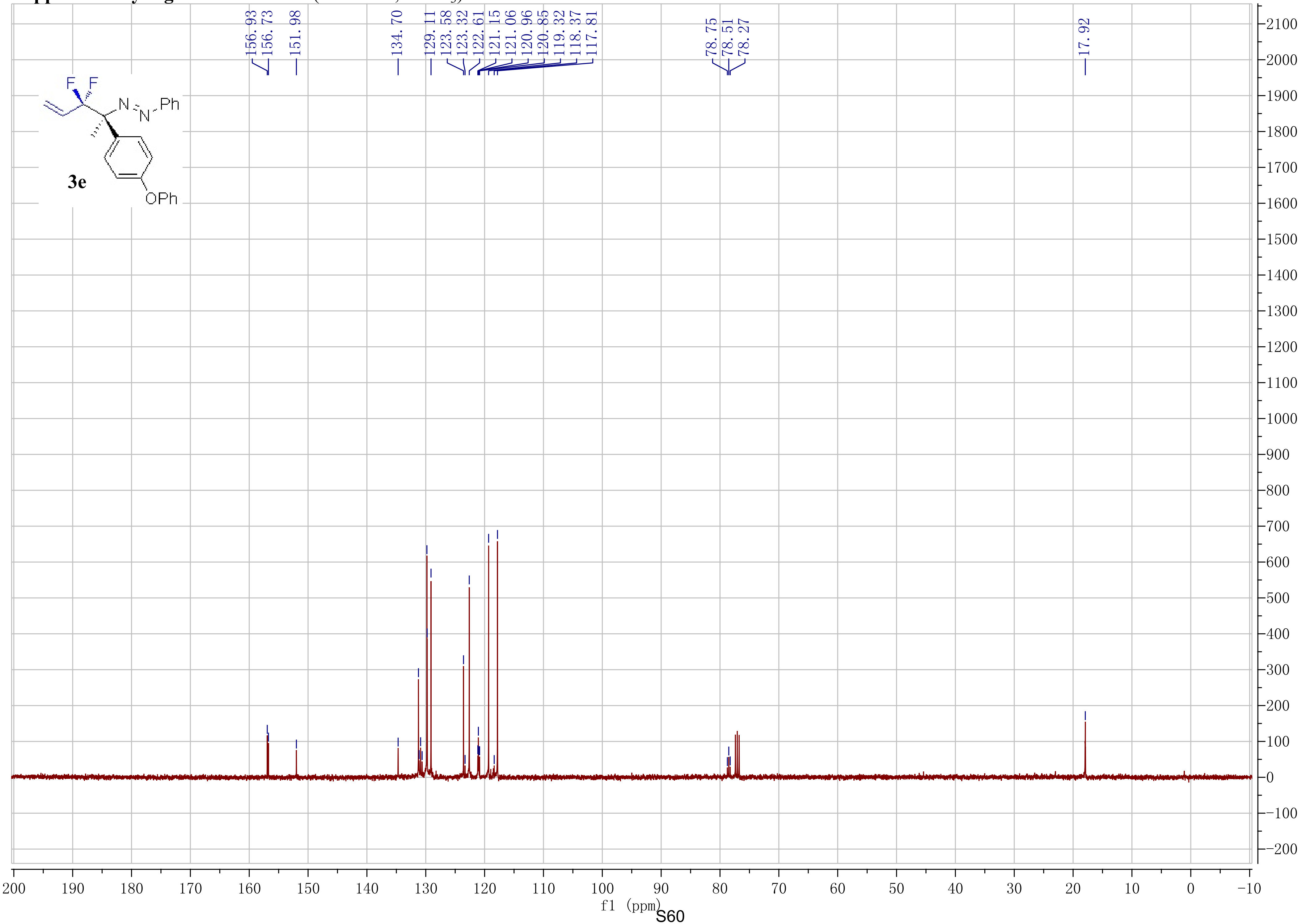

Supplementary Figure 31 ^{19}F NMR (376 MHz, $CDCl_3$) of **3e**

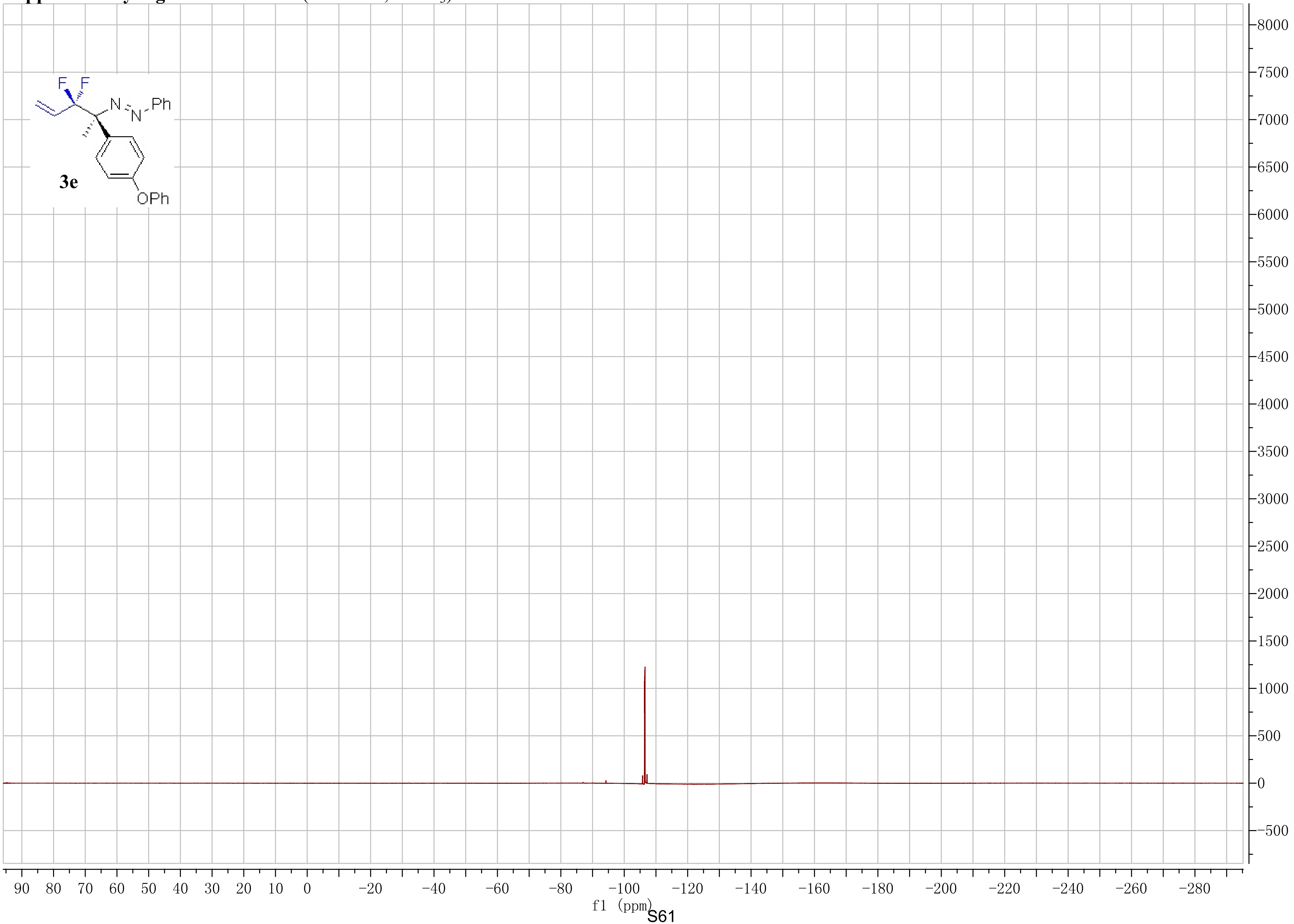

Supplementary Figure 32 HPLC spectra of racemic **3e**

8648 HS-13-73-1+- ODH 982 214 0.7

Sample Name:	**HS-13-73-1+- ODH 982 214 0.7**	*Injection Volume:*	**2.0**
Vial Number:	**RD7**	*Channel:*	**UV_VIS_2**
Sample Type:	**unknown**	*Wavelength:*	**214.0**
Control Program:	**test-dad3**	*Bandwidth:*	**4**
Quantif. Method:	**20170608**	*Dilution Factor:*	**1.0000**
Recording Time:	**2020-6-2 14:49**	*Sample Weight:*	**1.0000**
Run Time (min):	**30.00**	*Sample Amount:*	**1.0000**

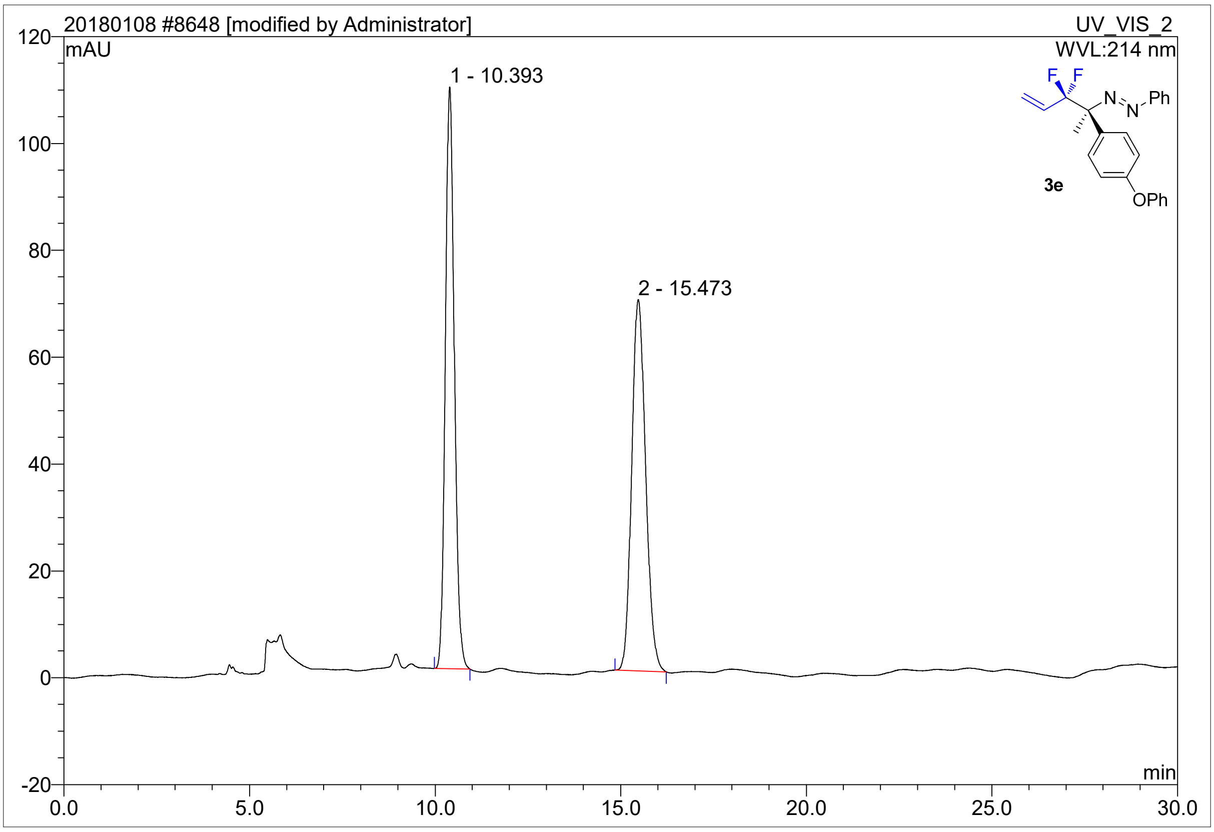

No.	Ret.Time min	Peak Name	Height mAU	Area mAU*min	Rel.Area %	Amount	Type
1	10.39	n.a.	108.928	30.626	50.22	n.a.	BMB
2	15.47	n.a.	69.534	30.361	49.78	n.a.	BMB
**Total:**			178.463	60.986	100.00	0.000	

Supplementary Figure 33 HPLC spectra of (*S*)-**3e**

8649 HS-13-62-8 ODH 982 214 0.7

Sample Name:	**HS-13-62-8 ODH 982 214 0.7**	*Injection Volume:*	**3.0**
Vial Number:	**RC7**	*Channel:*	**UV_VIS_2**
Sample Type:	**unknown**	*Wavelength:*	**214.0**
Control Program:	**test-dad3**	*Bandwidth:*	**4**
Quantif. Method:	**20170608**	*Dilution Factor:*	**1.0000**
Recording Time:	**2020-6-2 14:16**	*Sample Weight:*	**1.0000**
Run Time (min):	**30.00**	*Sample Amount:*	**1.0000**

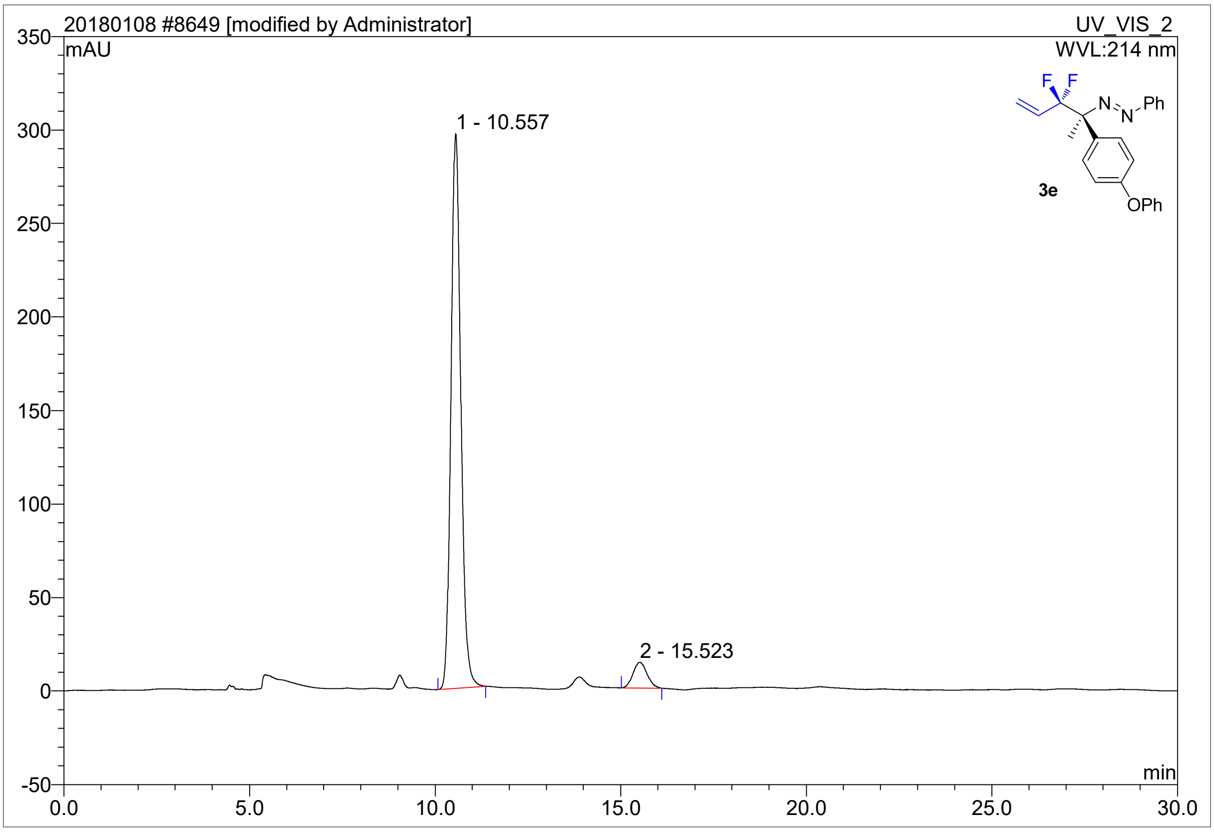

No.	Ret.Time min	Peak Name	Height mAU	Area mAU*min	Rel.Area %	Amount	Type
1	10.56	n.a.	296.765	91.340	93.83	n.a.	BMB
2	15.52	n.a.	13.762	6.011	6.17	n.a.	BMB
**Total:**			310.527	97.351	100.00	0.000	

Supplementary Figure 34 ^{1}H NMR (400 MHz, $CDCl_3$) of **3f**

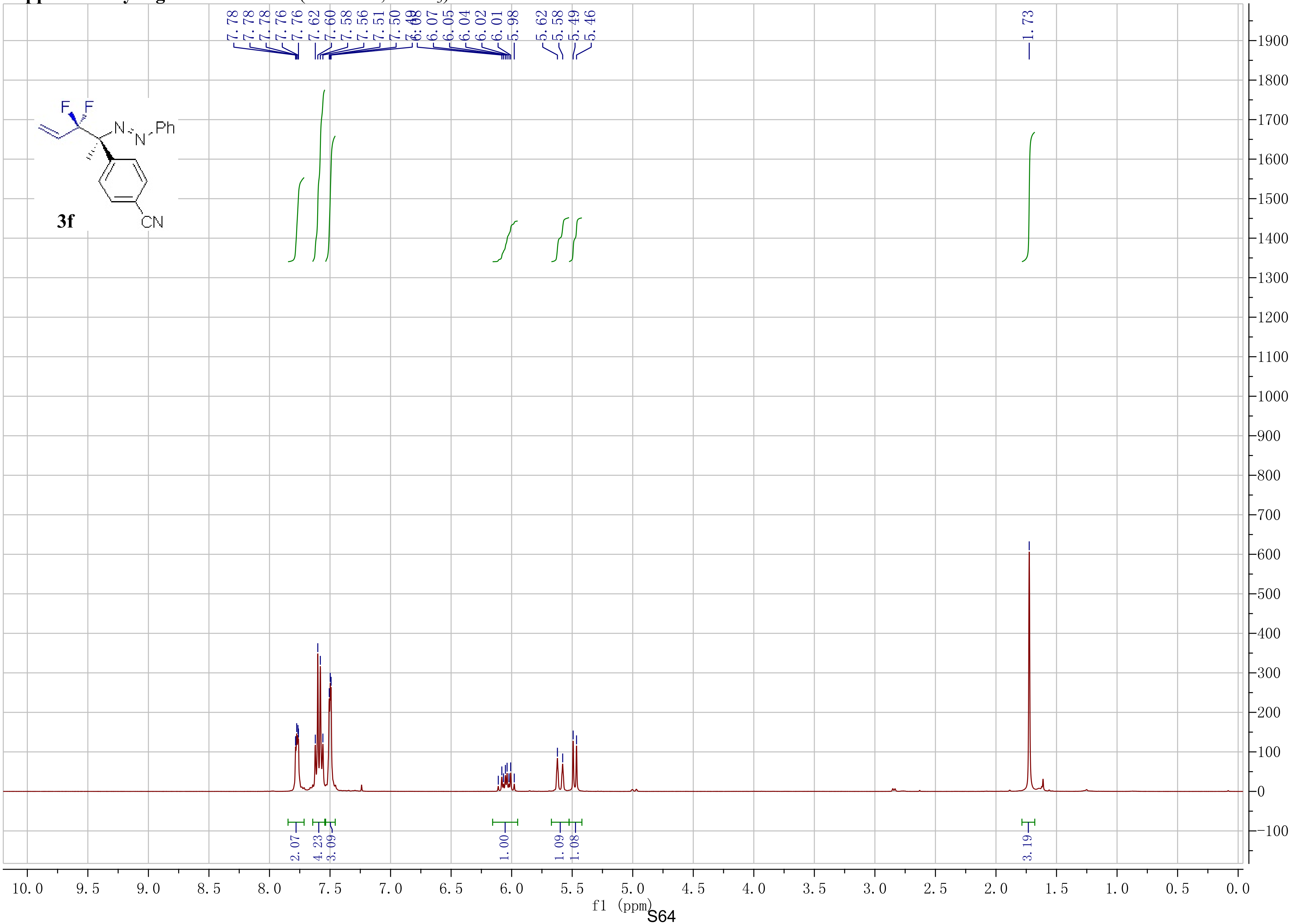

Supplementary Figure 35 ^{13}C NMR (101 MHz, $CDCl_3$) of **3f**

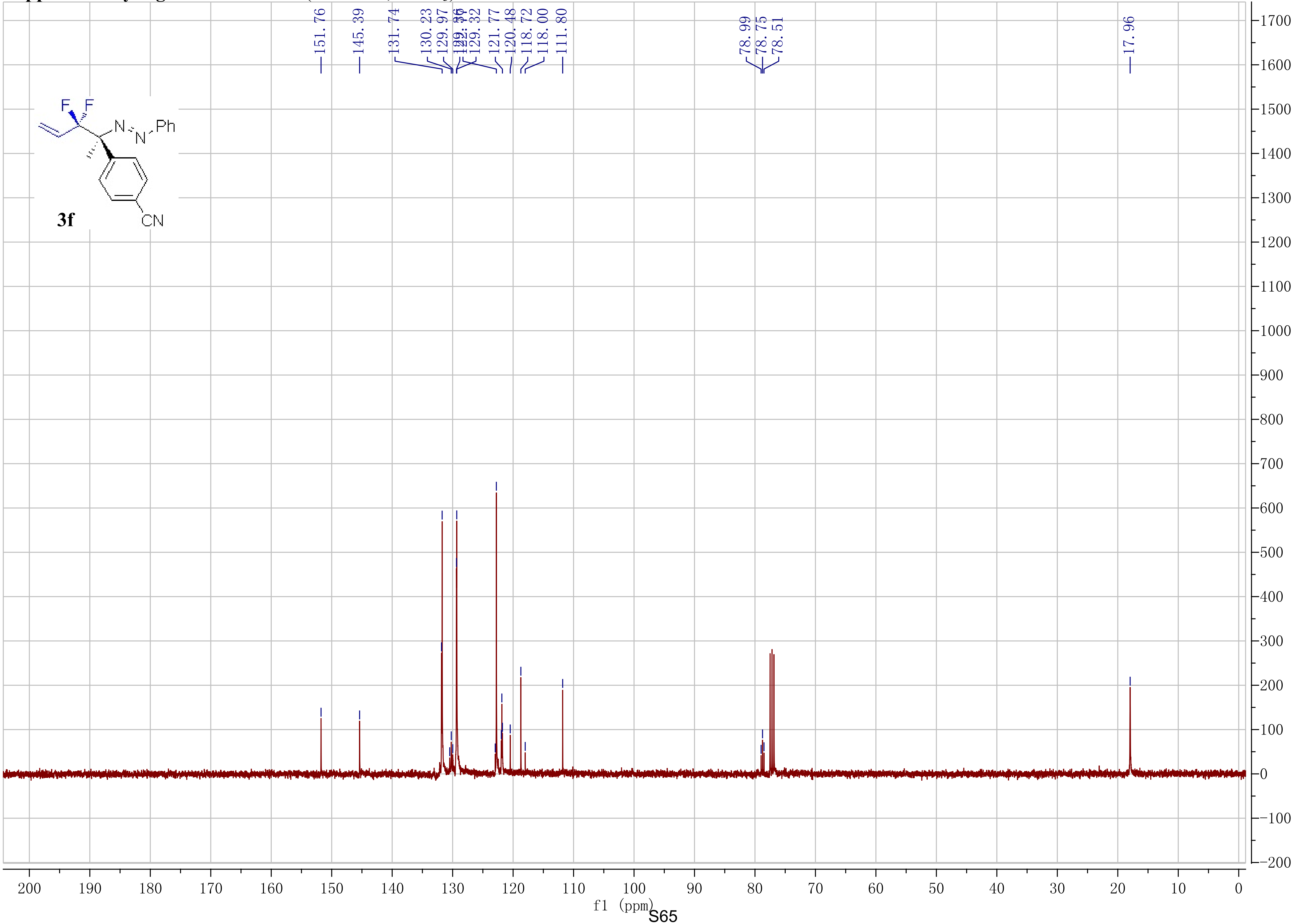

Supplementary Figure 36 ^{19}F NMR (376 MHz, $CDCl_3$) of **3f**

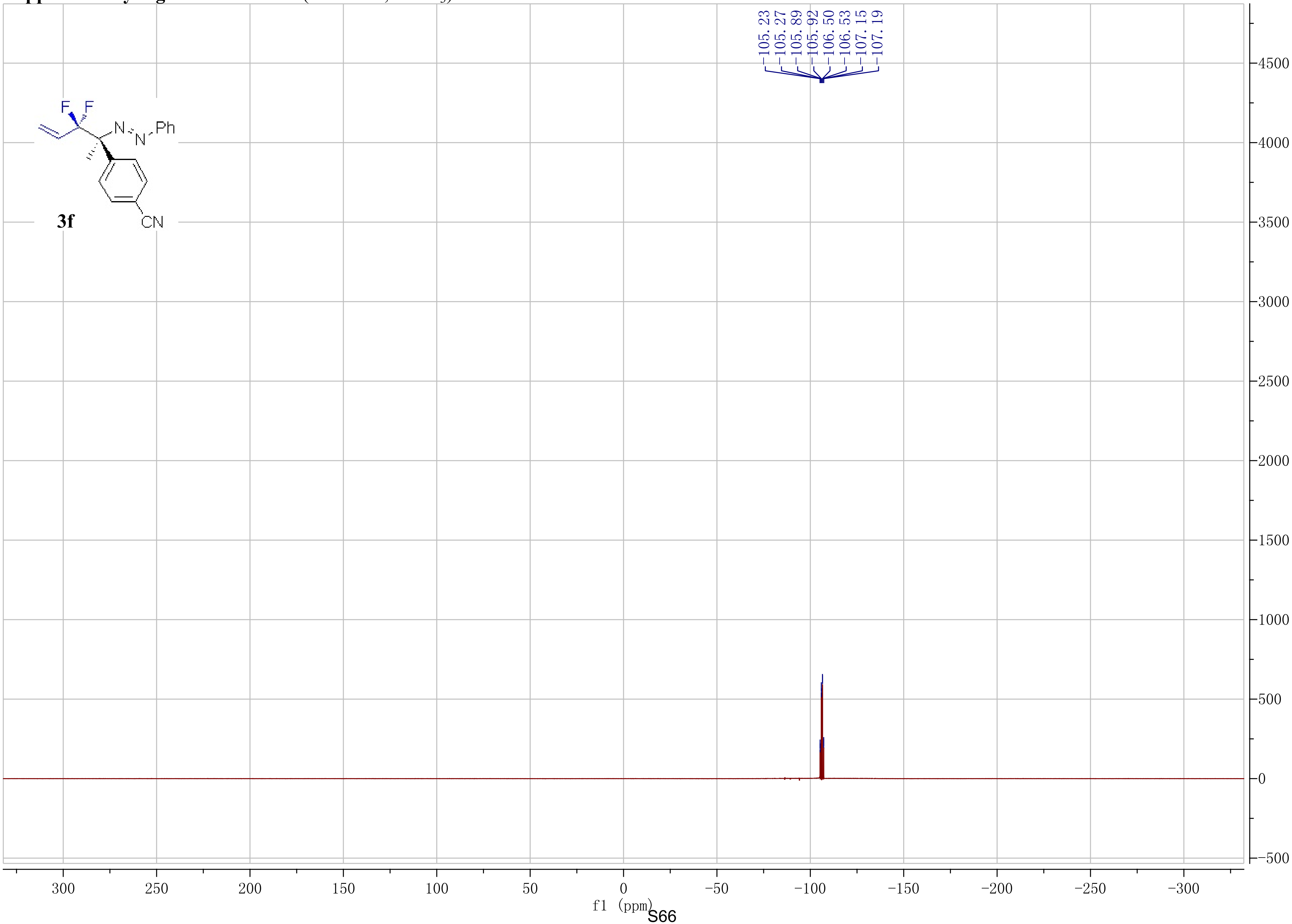

3f

Supplementary Figure 37 HPLC spectra of racemic **3f**

8667 HS-13-49-1+- IG 955 214 0.7

Sample Name:	**HS-13-49-1+- IG 955 214 0.7**		**3.0**
Vial Number:	**RB7**	*Channel:*	**UV_VIS_2**
Sample Type:	**unknown**	*Wavelength:*	**254.0**
Control Program:	**test-dad2**	*Bandwidth:*	**4**
Quantif. Method:	**20170608**	*Dilution Factor:*	**1.0000**
Recording Time:	**2020-6-4 9:56**	*Sample Weight:*	**1.0000**
Run Time (min):	**15.13**	*Sample Amount:*	**1.0000**

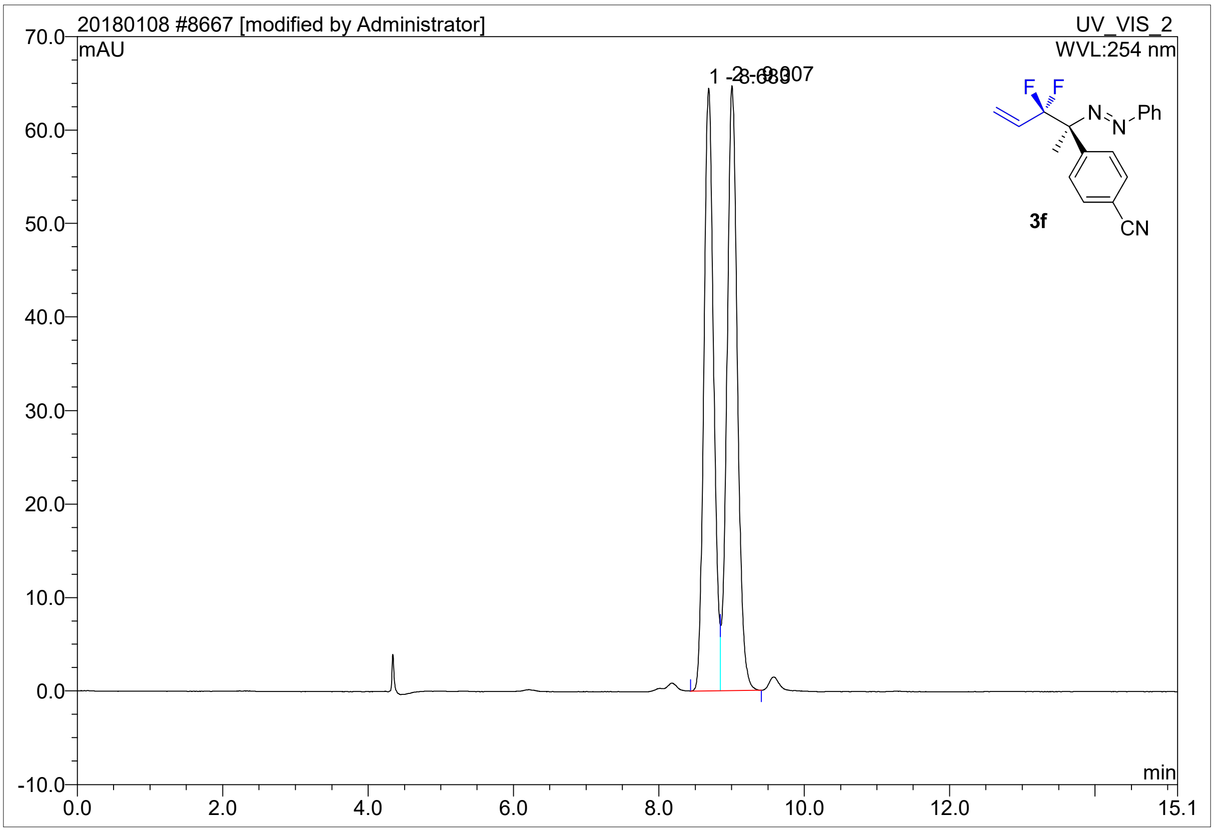

No.	Ret.Time min	Peak Name	Height mAU	Area mAU*min	Rel.Area %	Amount	Type
1	8.68	n.a.	64.477	10.042	48.18	n.a.	BM *
2	9.01	n.a.	64.727	10.801	51.82	n.a.	MB*
**Total:**			129.203	20.842	100.00	0.000	

Supplementary Figure 38 HPLC spectra of (*S*)-**3f**

8699 HS-13-62-1 IG 955 214 0.7

Sample Name:	**HS-13-62-1 IG 955 214 0.7**	*Injection Volume:*	**2.0**
Vial Number:	**RC6**	*Channel:*	**UV_VIS_2**
Sample Type:	**unknown**	*Wavelength:*	**214.0**
Control Program:	**test-dad2**	*Bandwidth:*	**4**
Quantif. Method:	**20170608**	*Dilution Factor:*	**1.0000**
Recording Time:	**2020-6-8 12:33**	*Sample Weight:*	**1.0000**
Run Time (min):	**32.51**	*Sample Amount:*	**1.0000**

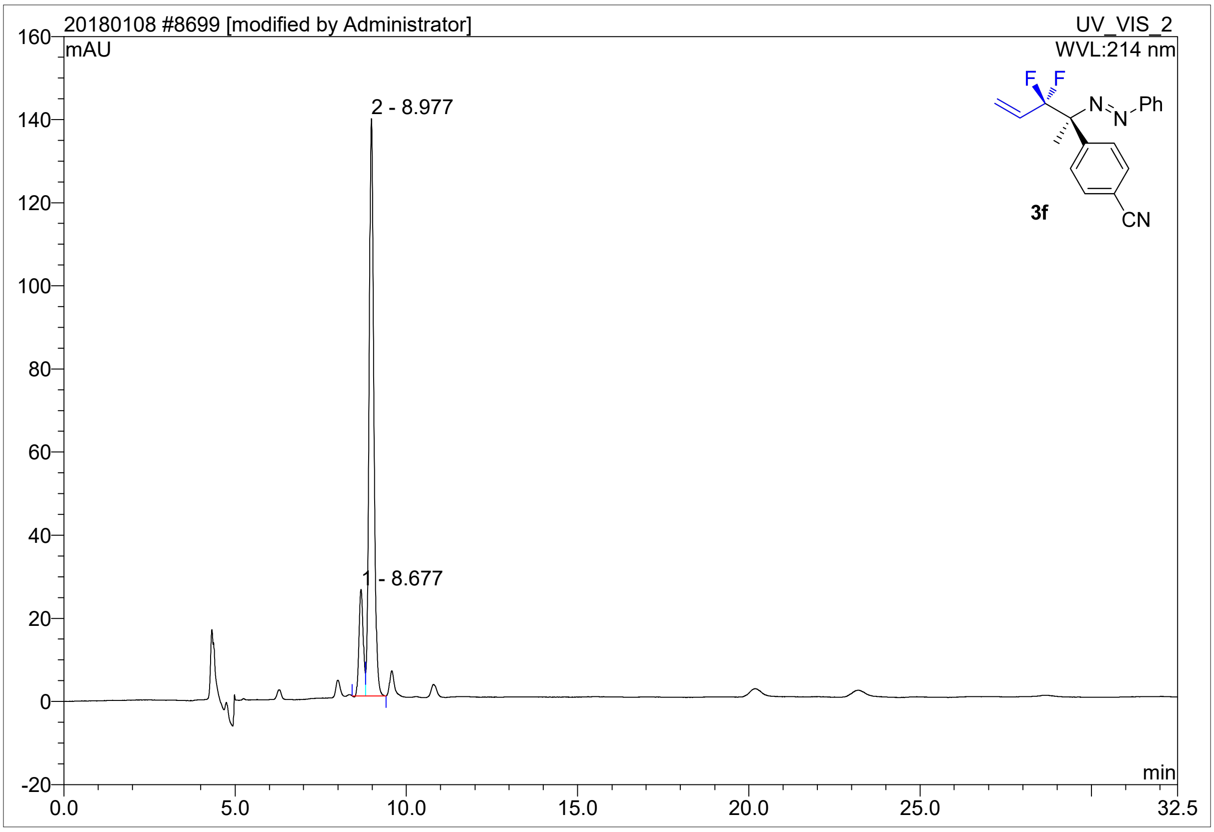

No.	Ret.Time min	Peak Name	Height mAU	Area mAU*min	Rel.Area %	Amount	Type
1	8.68	n.a.	25.685	3.702	14.27	n.a.	BM *
2	8.98	n.a.	138.966	22.241	85.73	n.a.	MB*
**Total:**			164.652	25.944	100.00	0.000	

default/Integration

Chromeleon (c) Dionex 1996-2006
Version 6.80 SR14 Build 4527 (238909)

Supplementary Figure 39 ^{1}H NMR (400 MHz, $CDCl_3$) of **3g**

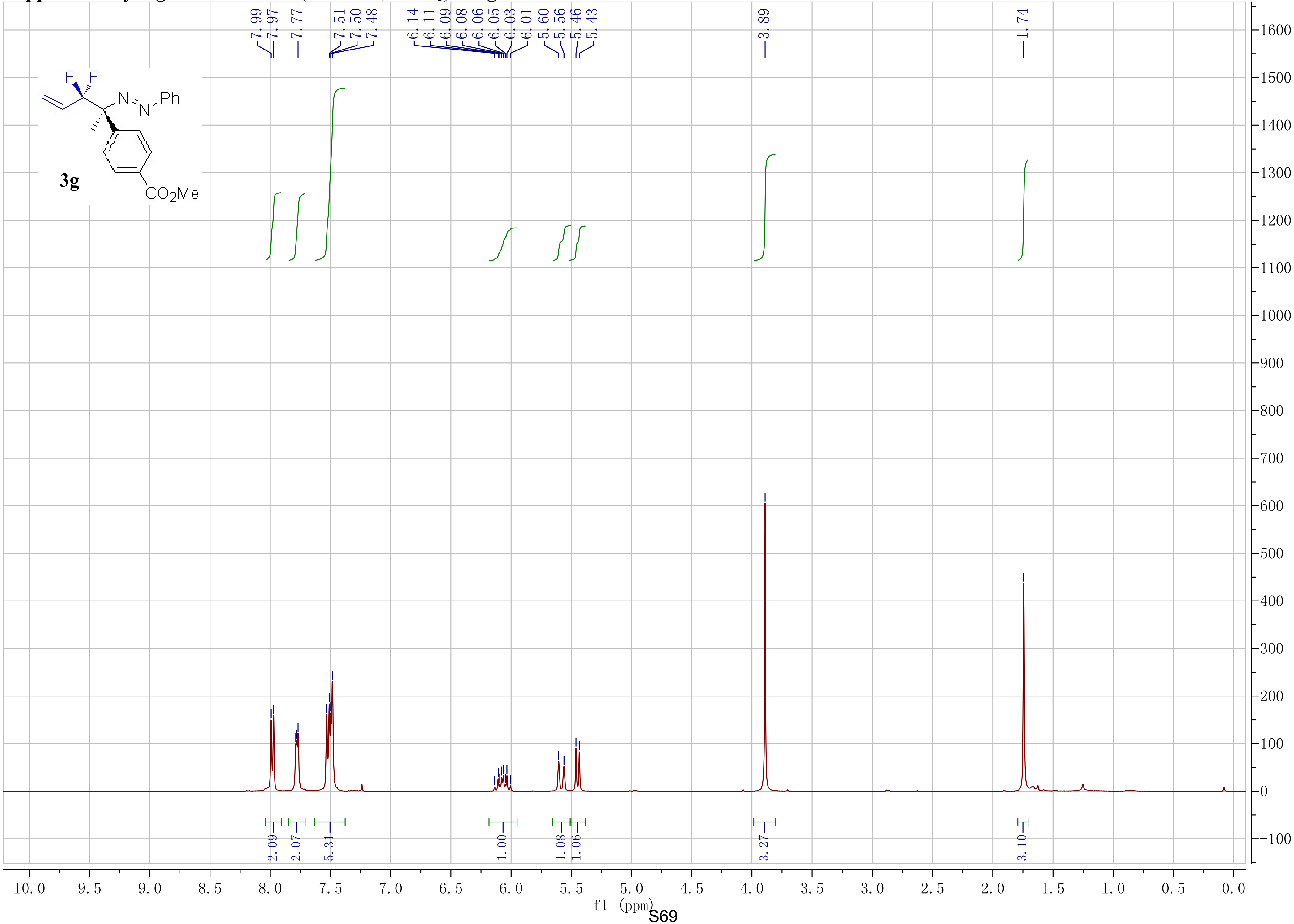

Supplementary Figure 40 ^{13}C NMR (101 MHz, $CDCl_3$) of **3g**

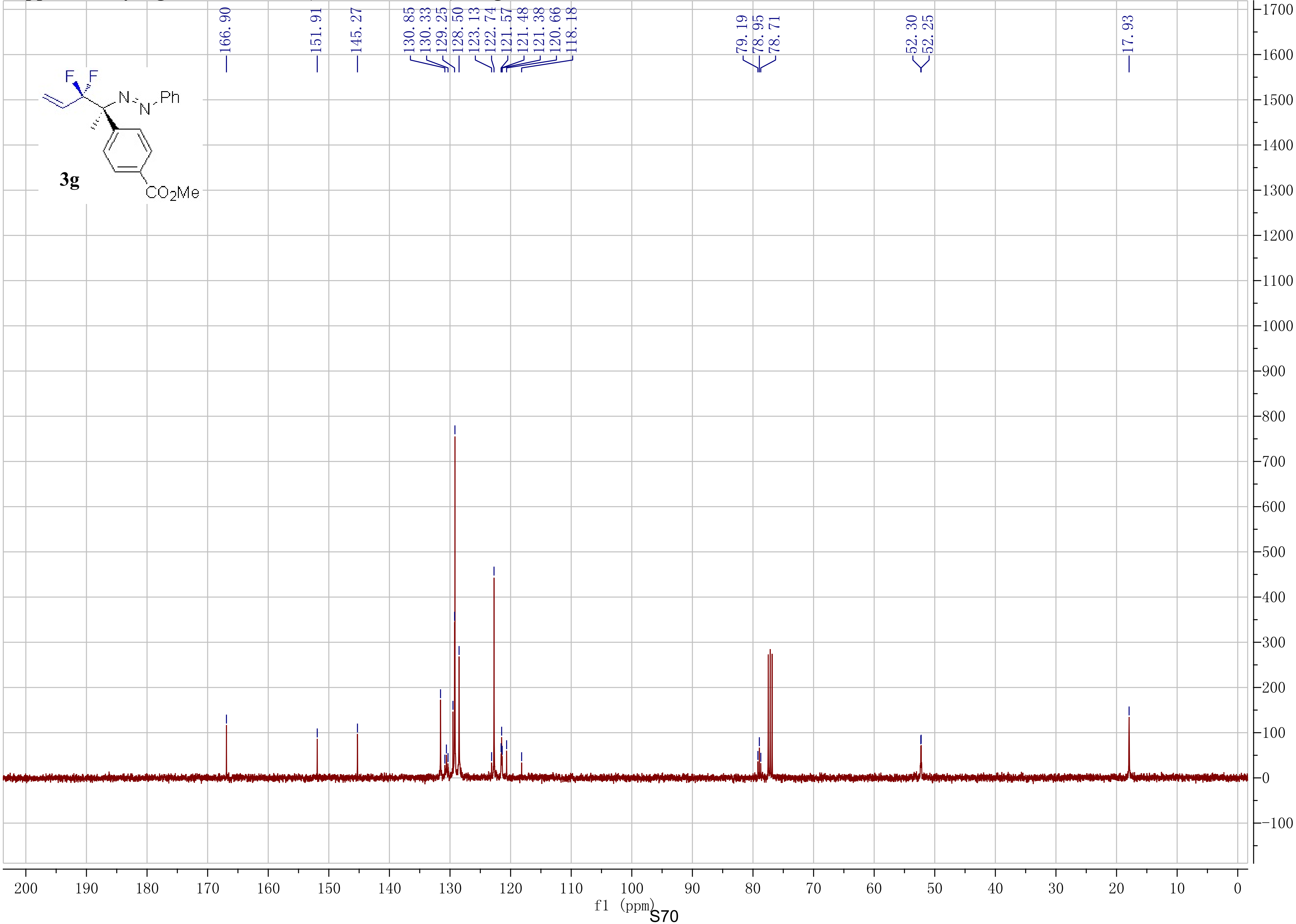

Supplementary Figure 41 ^{19}F NMR (376 MHz, $CDCl_3$) of **3g**

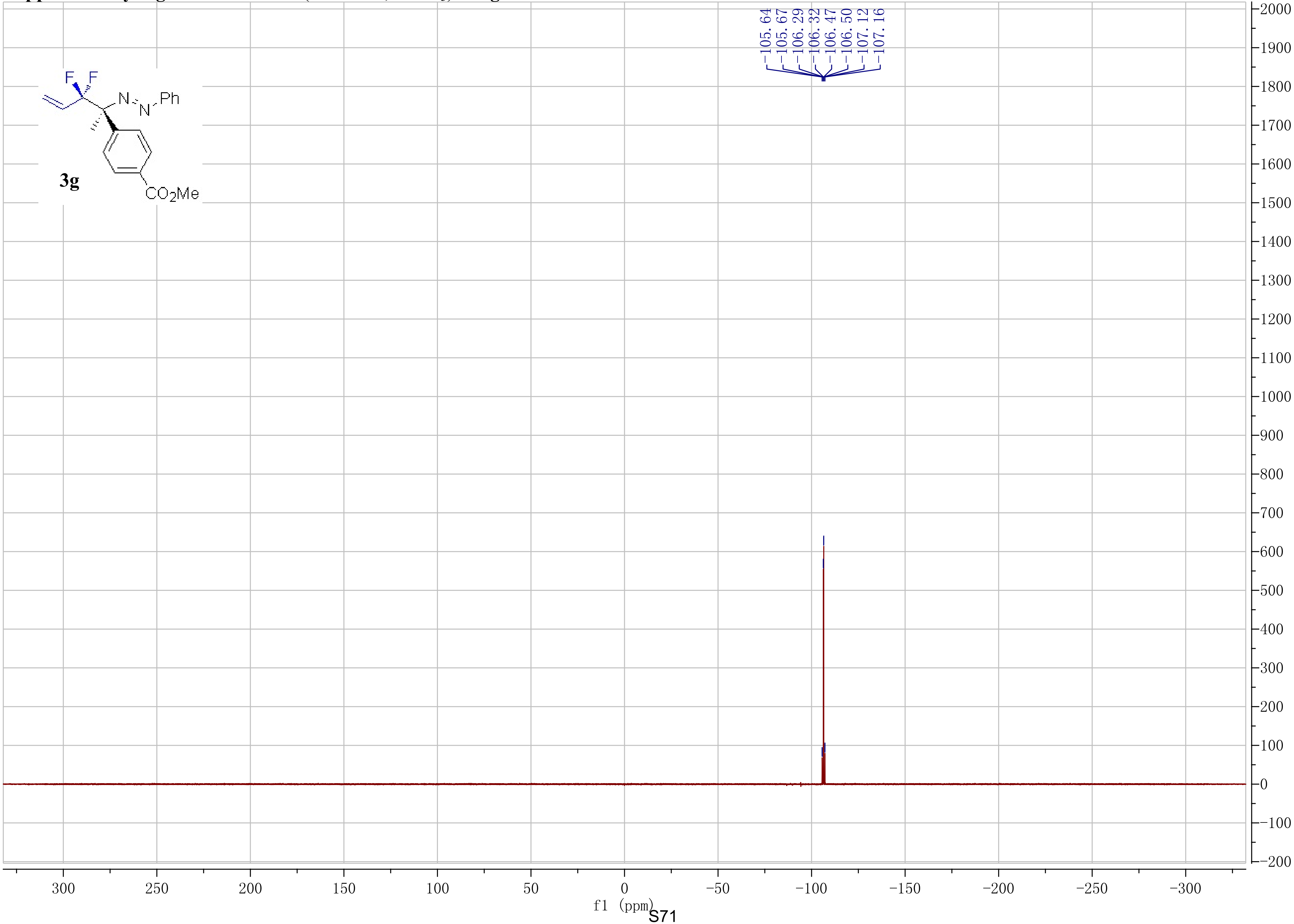

Supplementary Figure 42 HPLC spectra of racemic **3g**

Operator:Administrator Timebase:HPLC Sequence:20180108 | Page 1-1
2020-5-18 2:10 PM

8442 HS-13-46-6+- OJH 982 214 0.7

Sample Name:	**HS-13-46-6+- OJH 982 214 0.7**		**3.0**
Vial Number:	**RC7**	*Channel:*	**UV_VIS_2**
Sample Type:	**unknown**	*Wavelength:*	**214.0**
Control Program:	**test-dad3**	*Bandwidth:*	**4**
Quantif. Method:	**20170608**	*Dilution Factor:*	**1.0000**
Recording Time:	**2020-5-11 9:42**	*Sample Weight:*	**1.0000**
Run Time (min):	**40.00**	*Sample Amount:*	**1.0000**

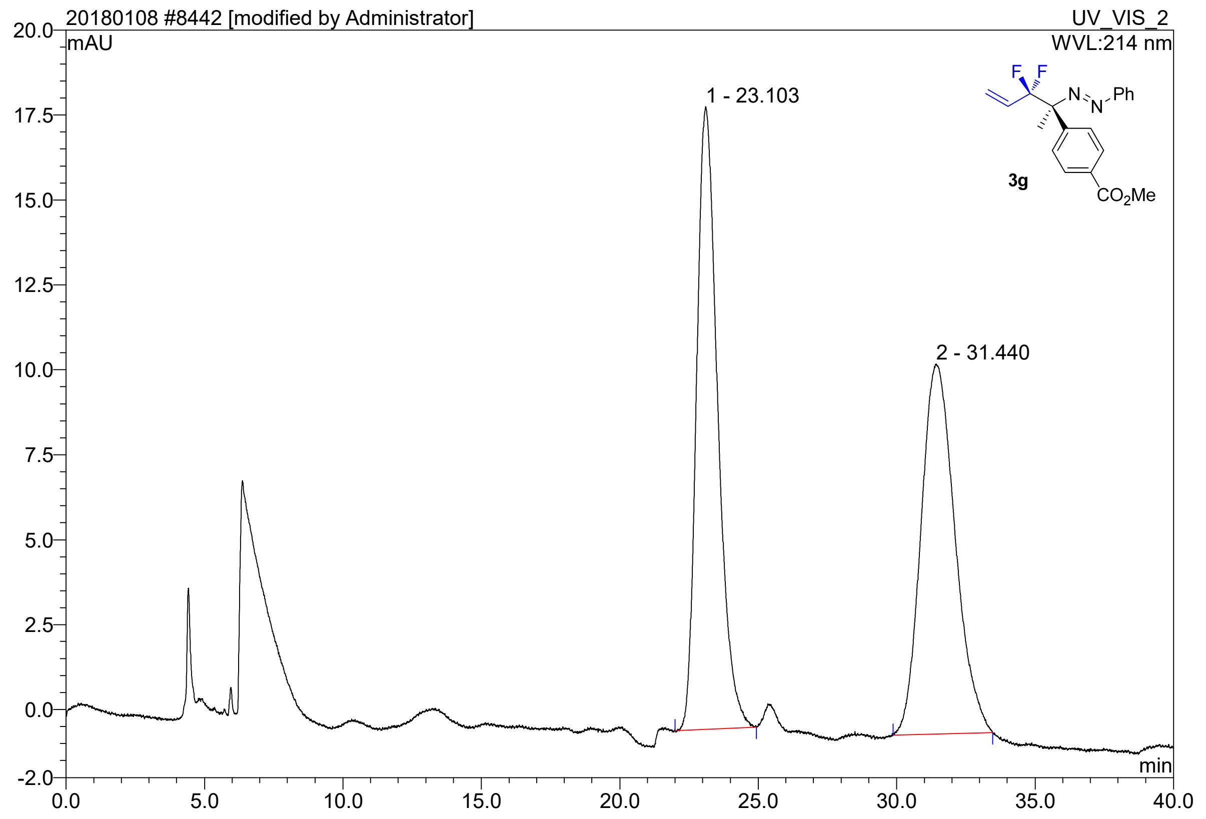

No.	Ret.Time min	Peak Name	Height mAU	Area mAU*min	Rel.Area %	Amount	Type
1	23.10	n.a.	18.330	16.195	51.06	n.a.	BMB*
2	31.44	n.a.	10.895	15.522	48.94	n.a.	BMB*
**Total:**			29.225	31.717	100.00	0.000	

default/Integration

Chromeleon (c) Dionex 1996-2006
Version 6.80 SR14 Build 4527 (238909)

Supplementary Figure 43 HPLC spectra of (*S*)-**3g**

8443 HS-13-62-2 OJH 982 214 0.7

Sample Name:	**HS-13-62-2 OJH 982 214 0.7**	*Injection Volume:*	**3.0**
Vial Number:	**RC6**	*Channel:*	**UV_VIS_2**
Sample Type:	**unknown**	*Wavelength:*	**214.0**
Control Program:	**test-dad3**	*Bandwidth:*	**4**
Quantif. Method:	**20170608**	*Dilution Factor:*	**1.0000**
Recording Time:	**2020-5-11 10:30**	*Sample Weight:*	**1.0000**
Run Time (min):	**40.00**	*Sample Amount:*	**1.0000**

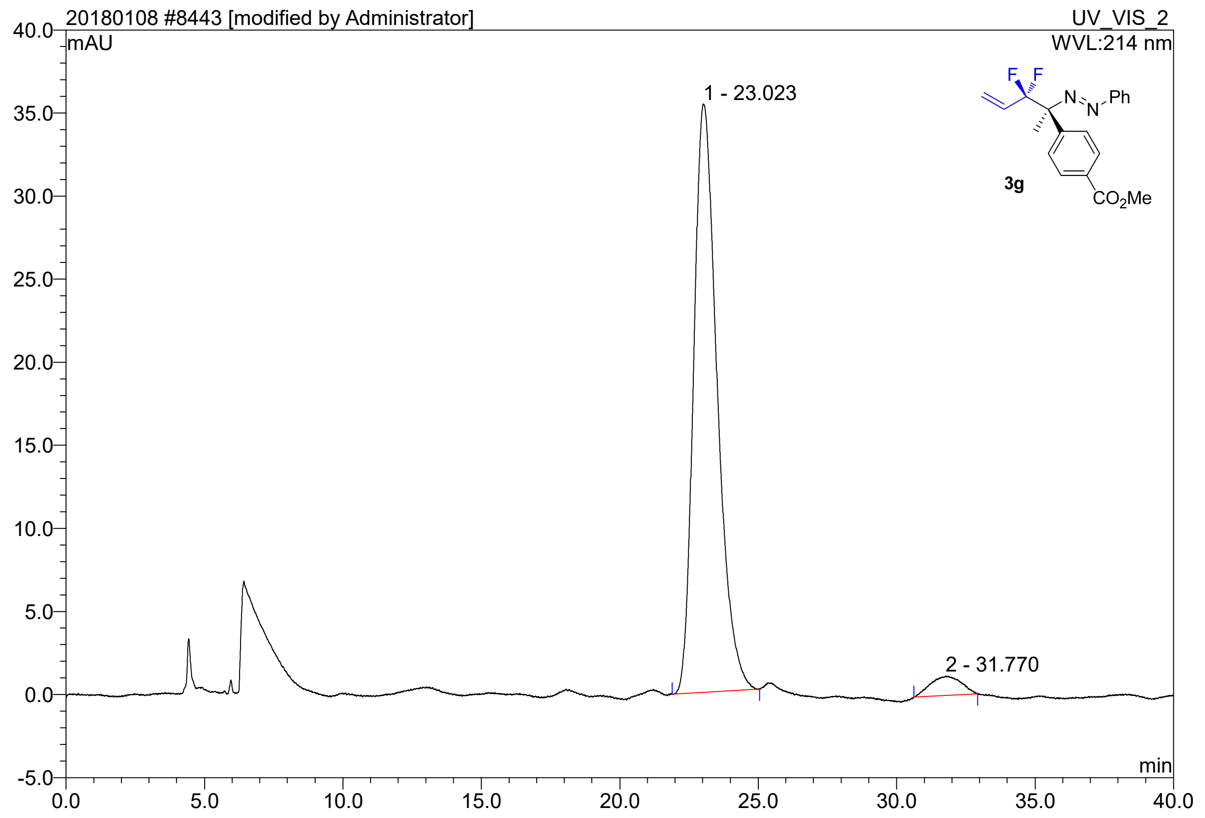

No.	Ret.Time min	Peak Name	Height mAU	Area mAU*min	Rel.Area %	Amount	Type
1	23.02	n.a.	35.412	34.862	95.84	n.a.	BMB*
2	31.77	n.a.	1.184	1.514	4.16	n.a.	BMB*
**Total:**			36.596	36.376	100.00	0.000	

Supplementary Figure 44 ^{1}H NMR (400 MHz, $CDCl_3$) of **3h**

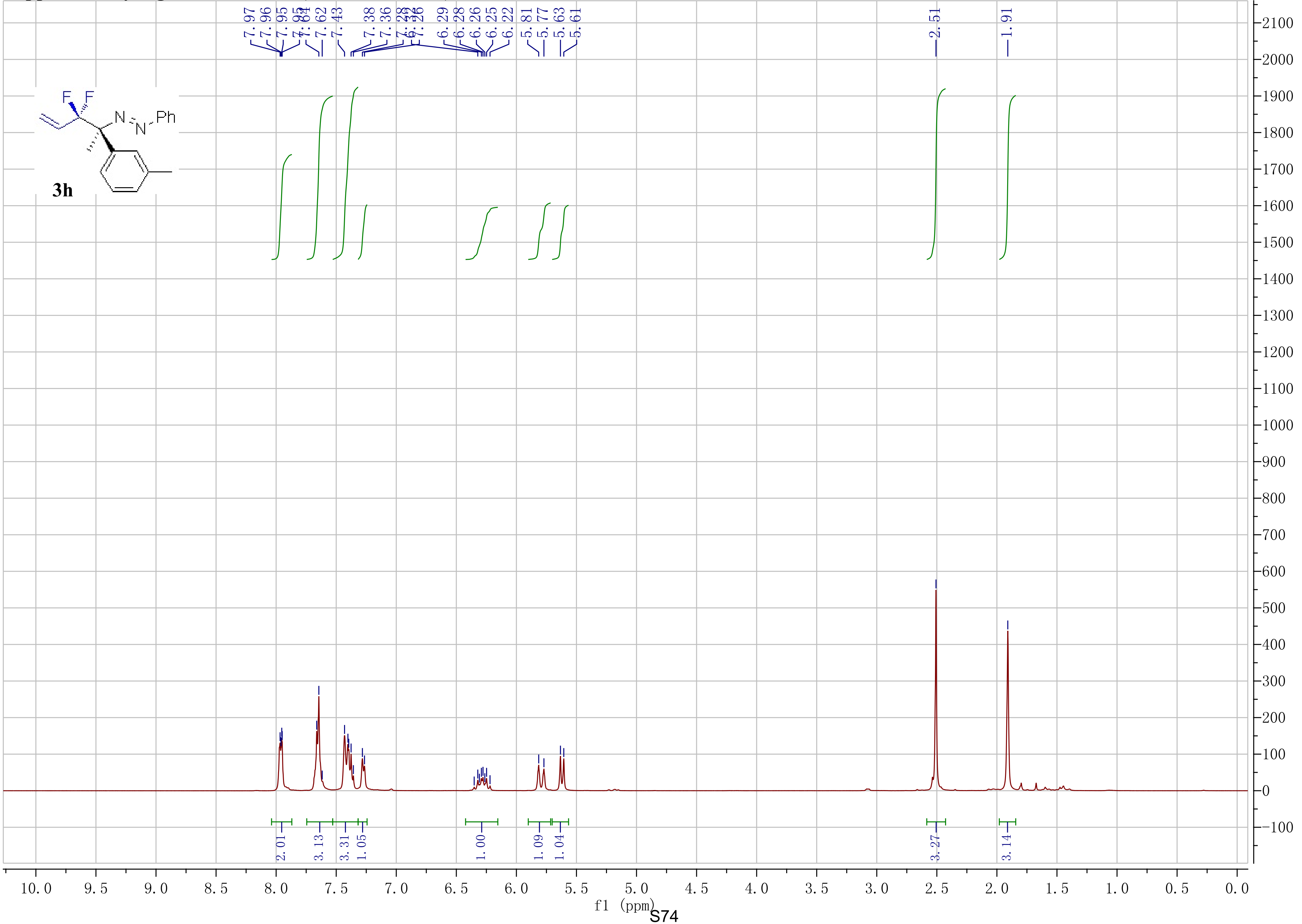

Supplementary Figure 45 ^{13}C NMR (101 MHz, $CDCl_3$) of **3**

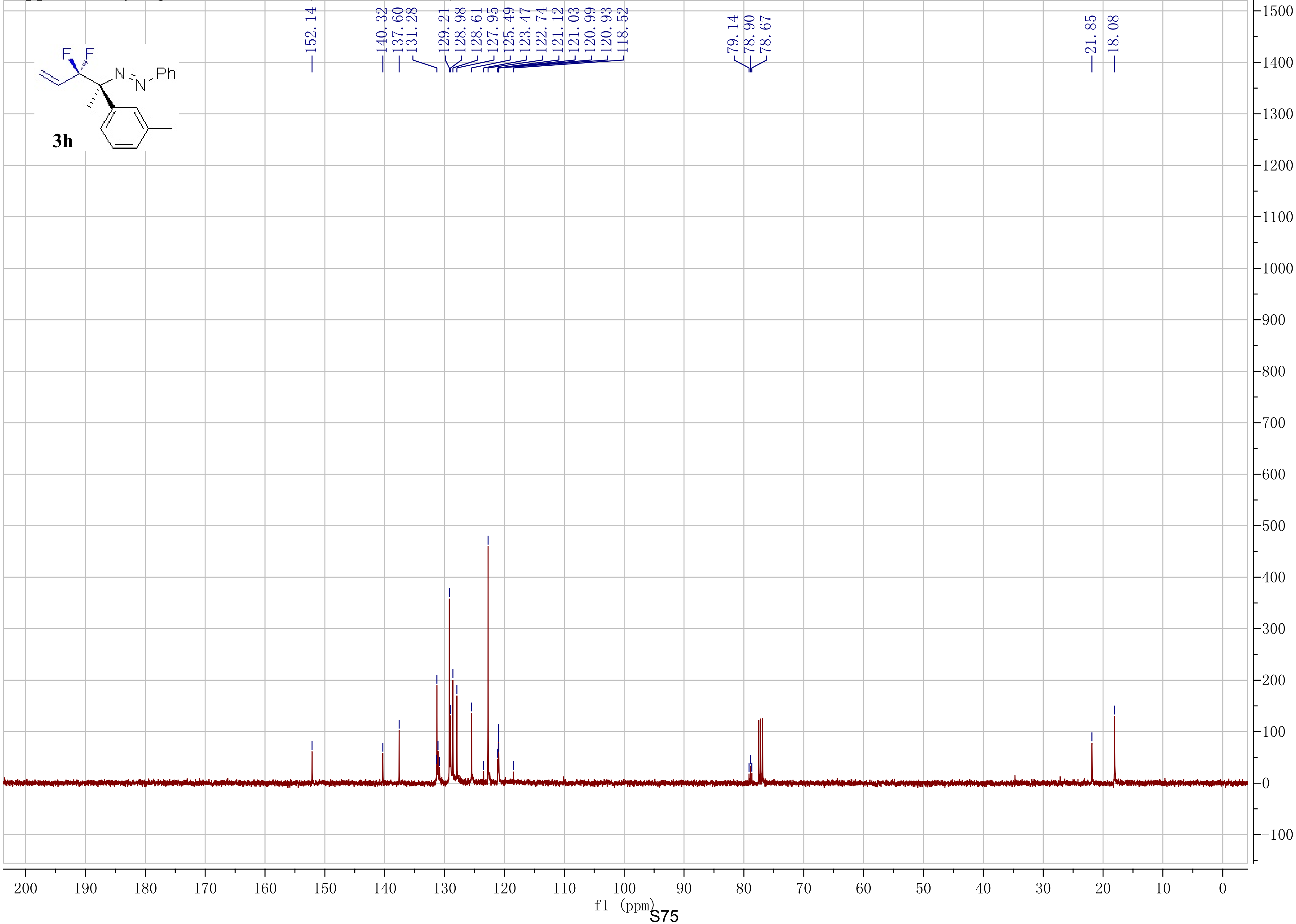

Supplementary Figure 46 ^{19}F NMR (376 MHz, $CDCl_3$) of **3h**

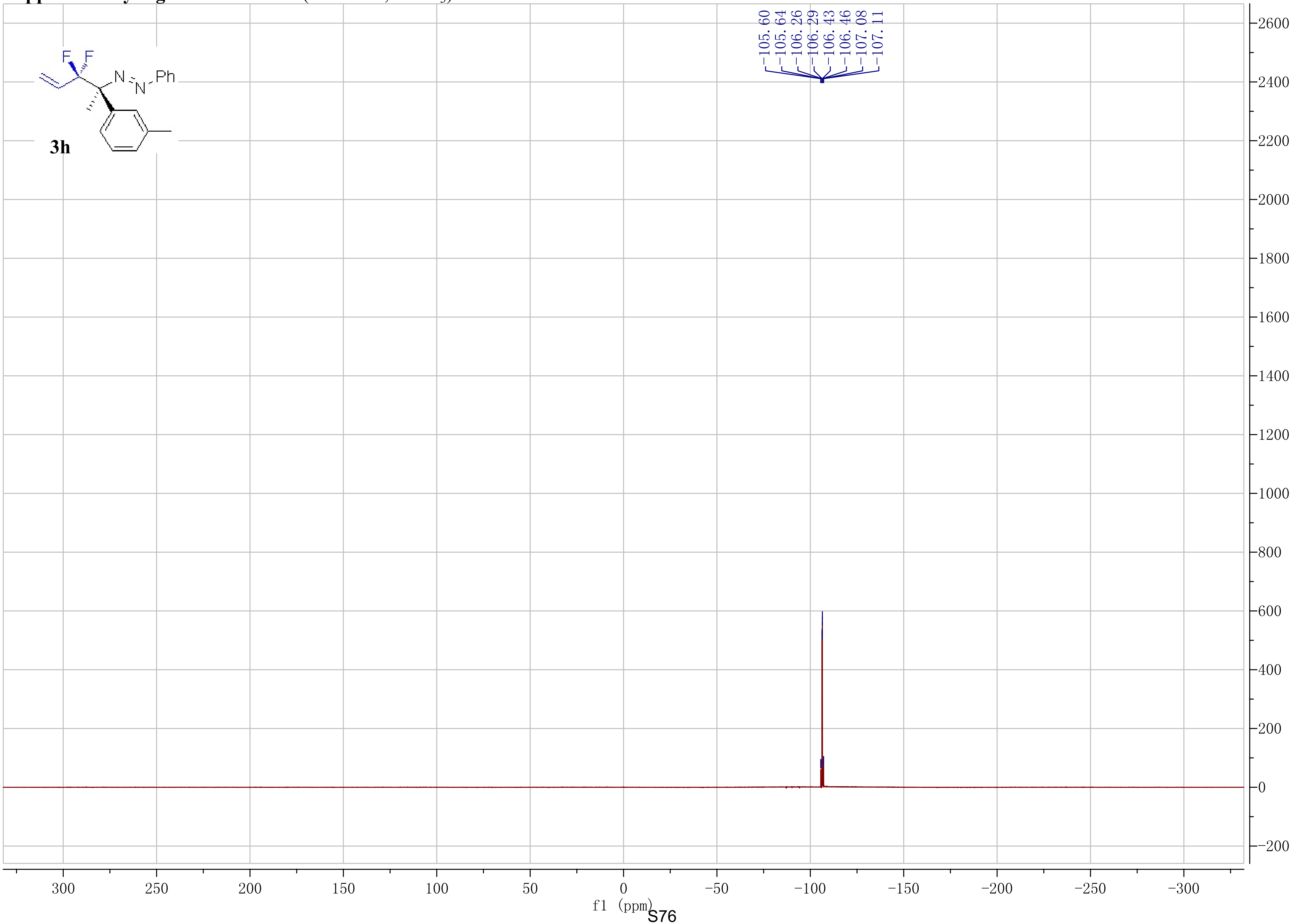

Supplementary Figure 47 HPLC spectra of racemic **3h**

8429 HS-13-46-2+- OJH 982 214 0.7

Sample Name:	**HS-13-46-2+- OJH 982 214 0.7**		**3.0**
Vial Number:	**RE7**	*Channel:*	**UV_VIS_2**
Sample Type:	**unknown**	*Wavelength:*	**214.0**
Control Program:	**test-dad3**	*Bandwidth:*	**4**
Quantif. Method:	**20170608**	*Dilution Factor:*	**1.0000**
Recording Time:	**2020-5-9 12:19**	*Sample Weight:*	**1.0000**
Run Time (min):	**20.00**	*Sample Amount:*	**1.0000**

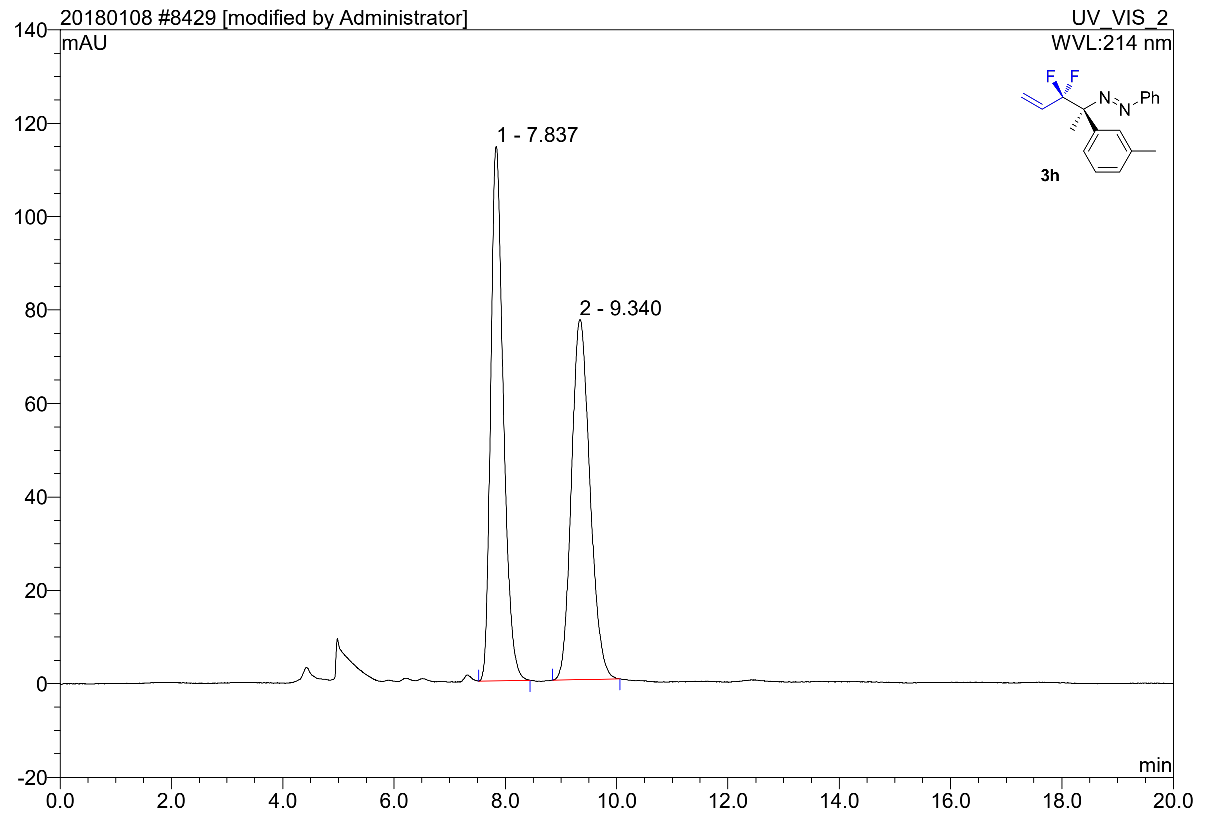

No.	Ret.Time min	Peak Name	Height mAU	Area mAU*min	Rel.Area %	Amount	Type
1	7.84	n.a.	114.464	30.099	50.33	n.a.	BMB*
2	9.34	n.a.	77.035	29.702	49.67	n.a.	BMB
**Total:**			191.499	59.802	100.00	0.000	

Supplementary Figure 48 HPLC spectra of (*S*)-**3h**

8430 HS-13-56-6 OJH 982 214 0.7

Sample Name:	**HS-13-56-6 OJH 982 214 0.7**	*Injection Volume:*	**3.0**
Vial Number:	**RD7**	*Channel:*	**UV_VIS_2**
Sample Type:	**unknown**	*Wavelength:*	**214.0**
Control Program:	**test-dad3**	*Bandwidth:*	**4**
Quantif. Method:	**20170608**	*Dilution Factor:*	**1.0000**
Recording Time:	**2020-5-9 11:49**	*Sample Weight:*	**1.0000**
Run Time (min):	**27.15**	*Sample Amount:*	**1.0000**

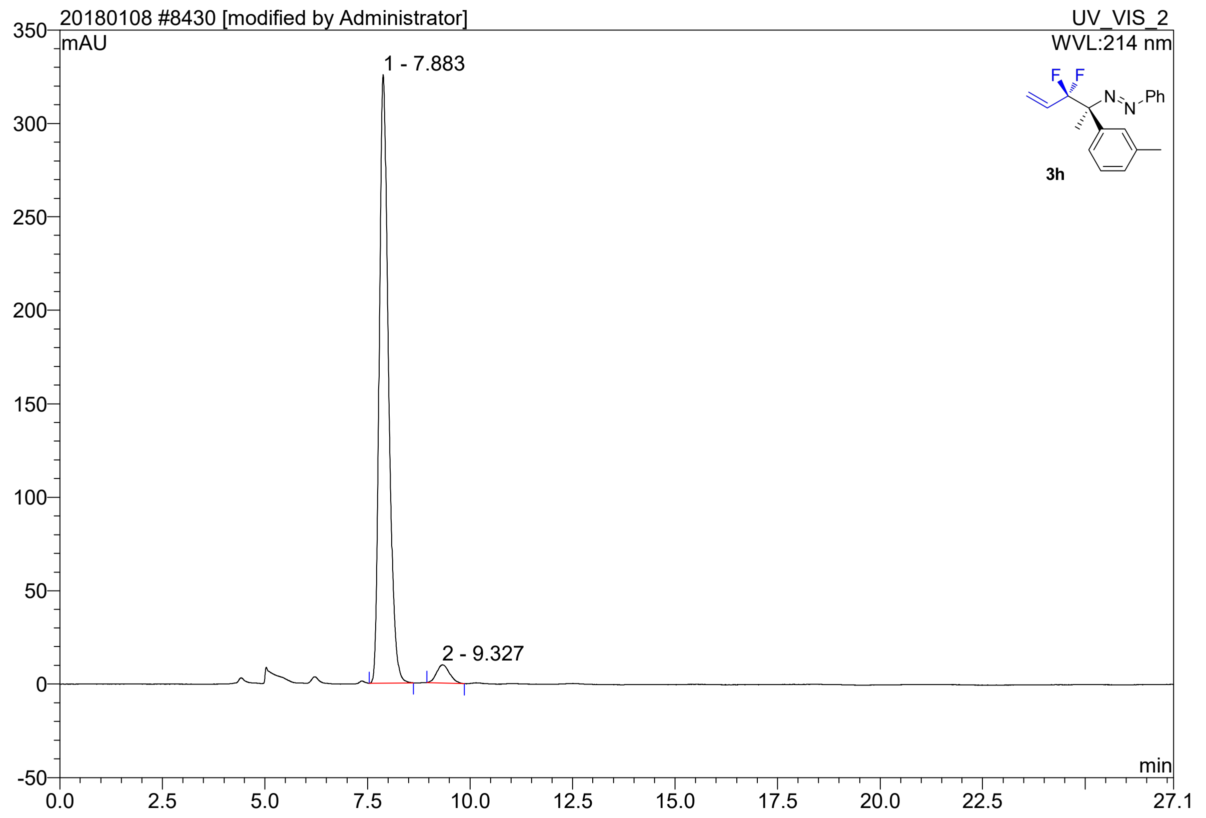

No.	Ret.Time min	Peak Name	Height mAU	Area mAU*min	Rel.Area %	Amount	Type
1	7.88	n.a.	325.692	85.139	95.98	n.a.	BMB*
2	9.33	n.a.	9.731	3.565	4.02	n.a.	BMB
**Total:**			335.423	88.703	100.00	0.000	

Supplementary Figure 49 ^{1}H NMR (400 MHz, $CDCl_3$) of **3i**

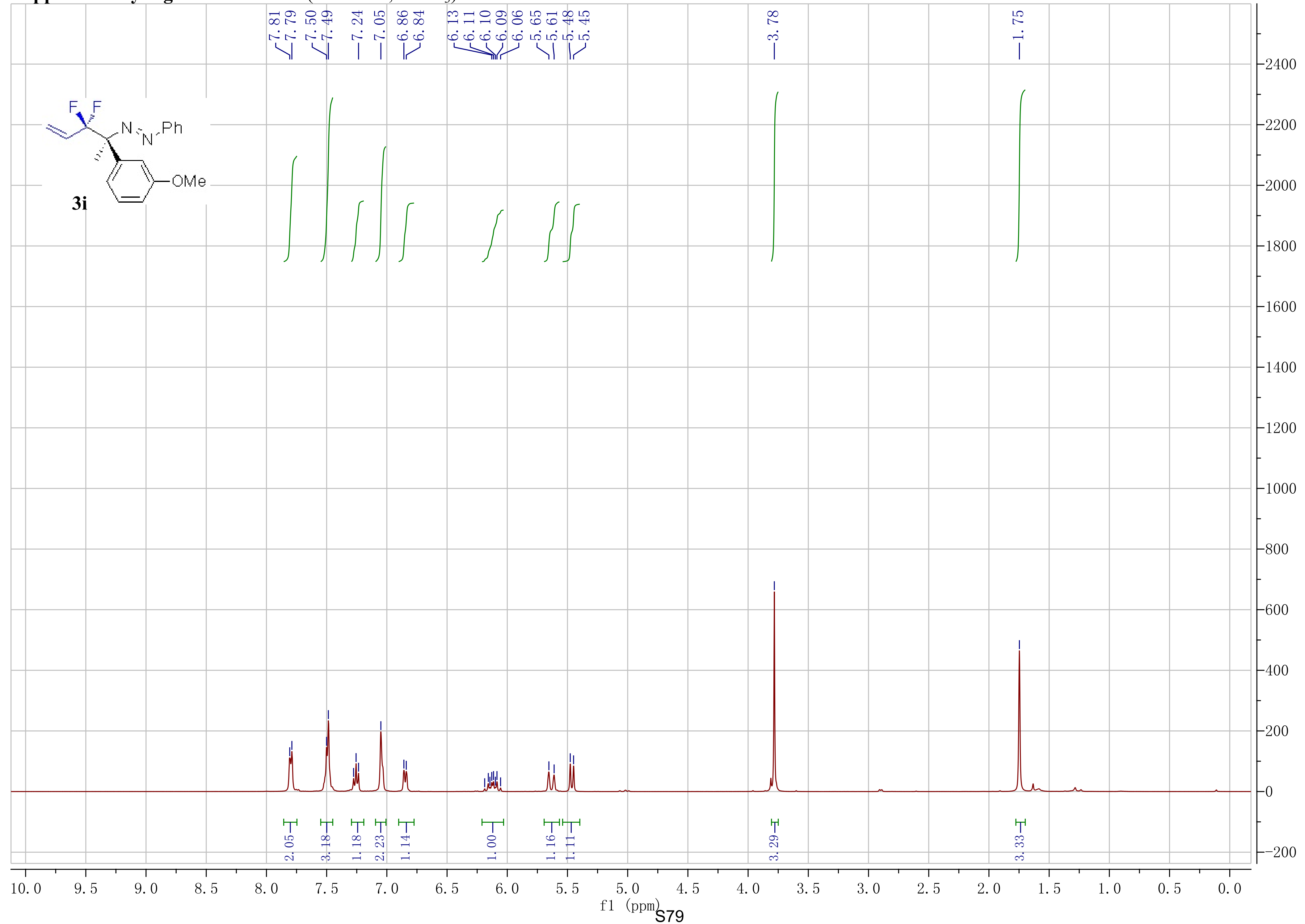

Supplementary Figure 50 ^{13}C NMR (101 MHz, $CDCl_3$) of **3i**

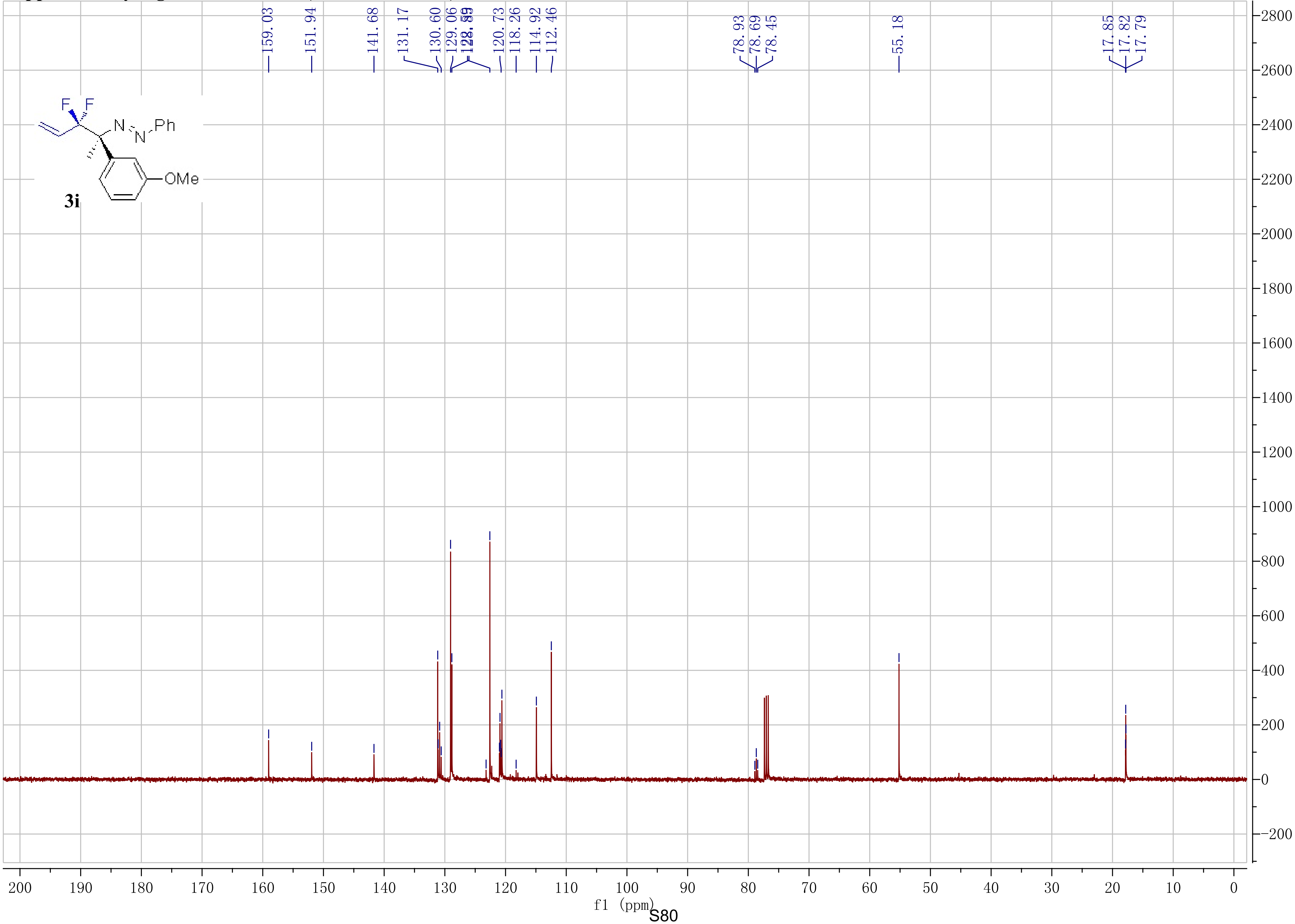

Supplementary Figure 51 ^{19}F NMR (376 MHz, $CDCl_3$) of **3i**

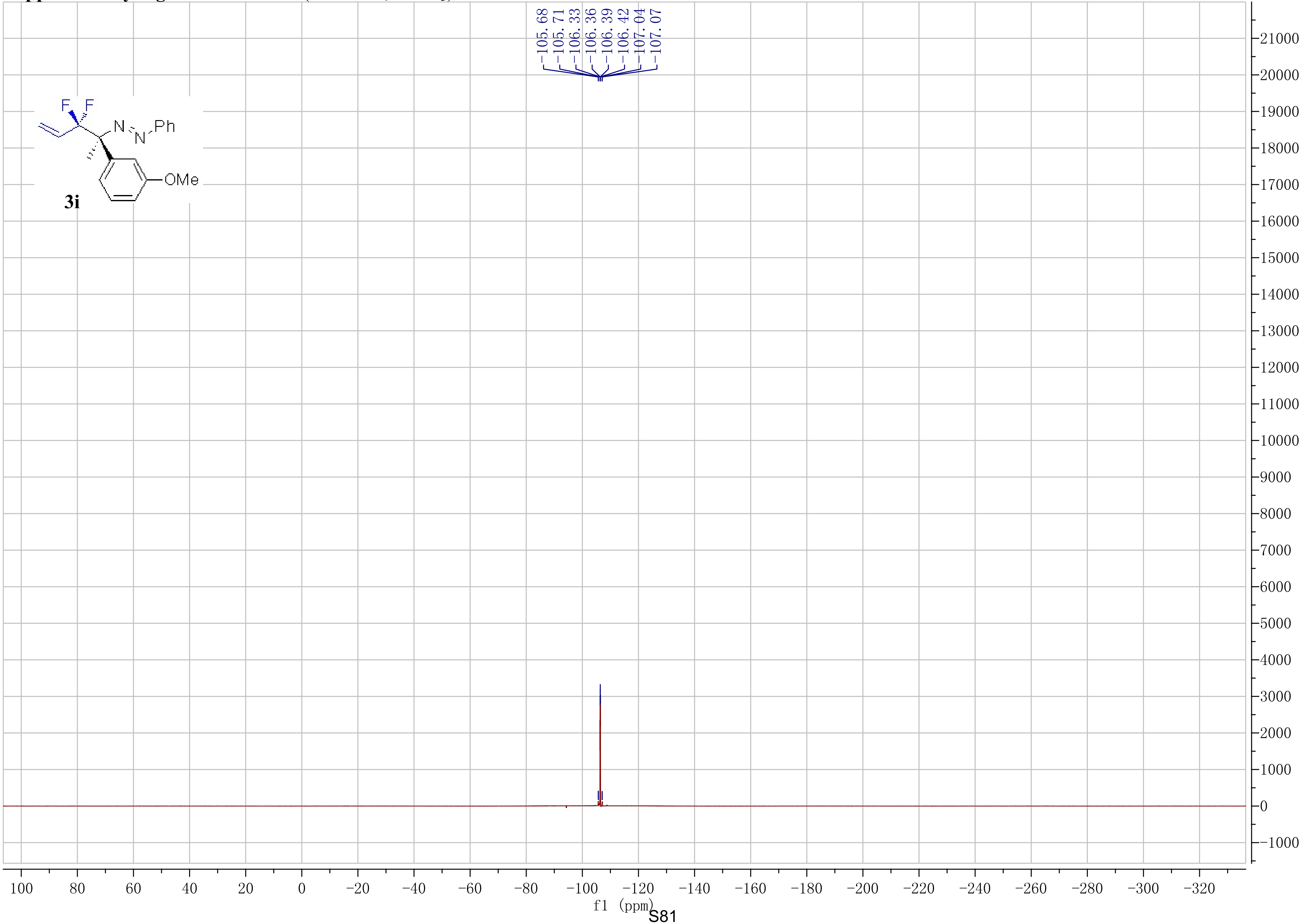

Supplementary Figure 52 HPLC spectra of racemic **3i**

8468 HS-13-48-1+- ADH 982 214 0.7

Sample Name:	**HS-13-48-1+- ADH 982 214 0.7**		**3.0**
Vial Number:	**RD1**	*Channel:*	**UV_VIS_2**
Sample Type:	**unknown**	*Wavelength:*	**214.0**
Control Program:	**test-dad3**	*Bandwidth:*	**4**
Quantif. Method:	**20170608**	*Dilution Factor:*	**1.0000**
Recording Time:	**2020-5-14 13:07**	*Sample Weight:*	**1.0000**
Run Time (min):	**10.88**	*Sample Amount:*	**1.0000**

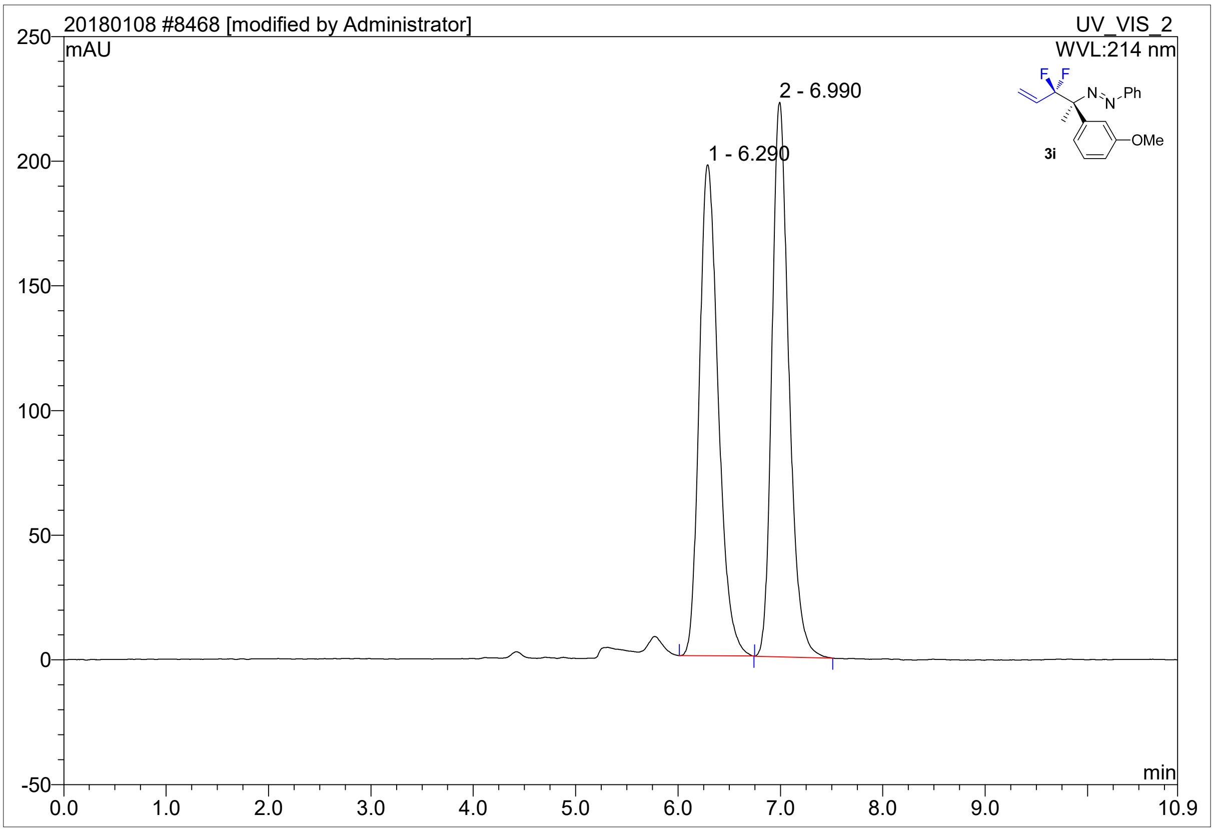

No.	Ret.Time min	Peak Name	Height mAU	Area mAU*min	Rel.Area %	Amount	Type
1	6.29	n.a.	197.045	42.383	49.66	n.a.	BMB*
2	6.99	n.a.	222.442	42.958	50.34	n.a.	BMB
**Total:**			419.487	85.341	100.00	0.000	

Supplementary Figure 53 HPLC spectra of (*S*)-**3i**

8469 HS-13-69-1 ADH 982 214 0.7

Sample Name:	**HS-13-69-1 ADH 982 214 0.7**	*Injection Volume:*	**3.0**
Vial Number:	**RC1**	*Channel:*	**UV_VIS_2**
Sample Type:	**unknown**	*Wavelength:*	**214.0**
Control Program:	**test-dad3**	*Bandwidth:*	**4**
Quantif. Method:	**20170608**	*Dilution Factor:*	**1.0000**
Recording Time:	**2020-5-14 13:20**	*Sample Weight:*	**1.0000**
Run Time (min):	**16.74**	*Sample Amount:*	**1.0000**

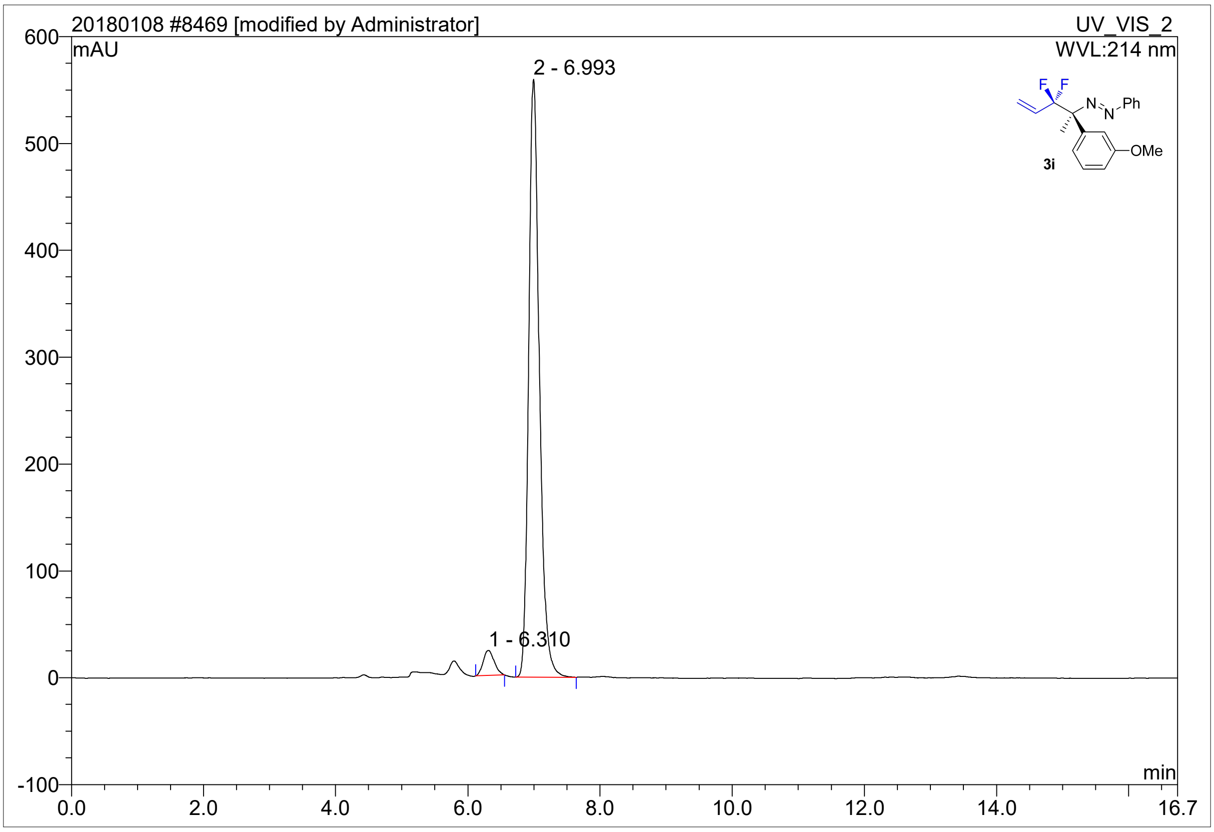

No.	Ret.Time min	Peak Name	Height mAU	Area mAU*min	Rel.Area %	Amount	Type
1	6.31	n.a.	23.493	4.519	4.12	n.a.	BMB*
2	6.99	n.a.	559.764	105.179	95.88	n.a.	BMB
**Total:**			583.257	109.698	100.00	0.000	

Supplementary Figure 54 ^{1}H NMR (400 MHz, $CDCl_3$) of **3k**

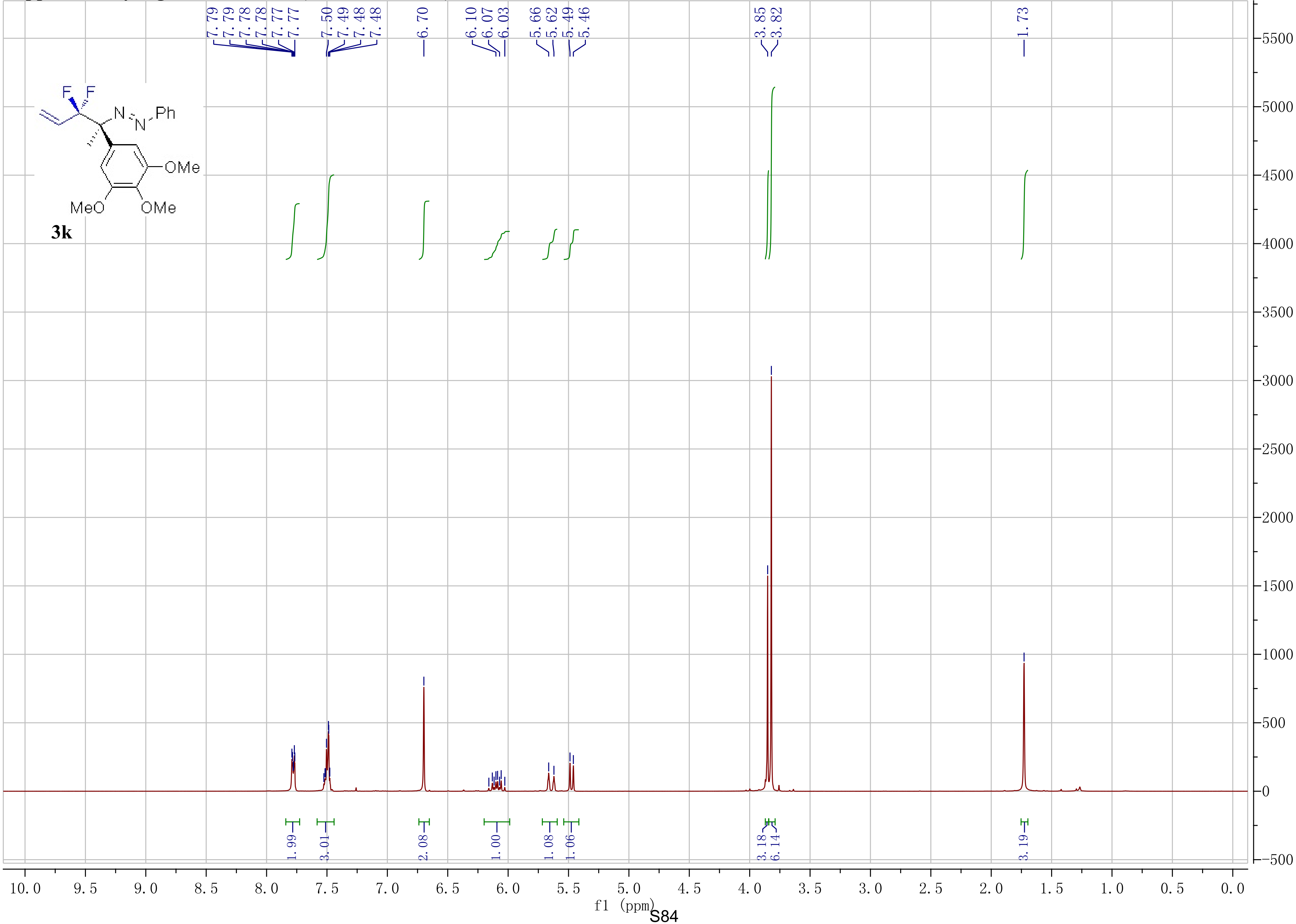

Supplementary Figure 55 ^{13}C NMR (101 MHz, $CDCl_3$) of **3k**

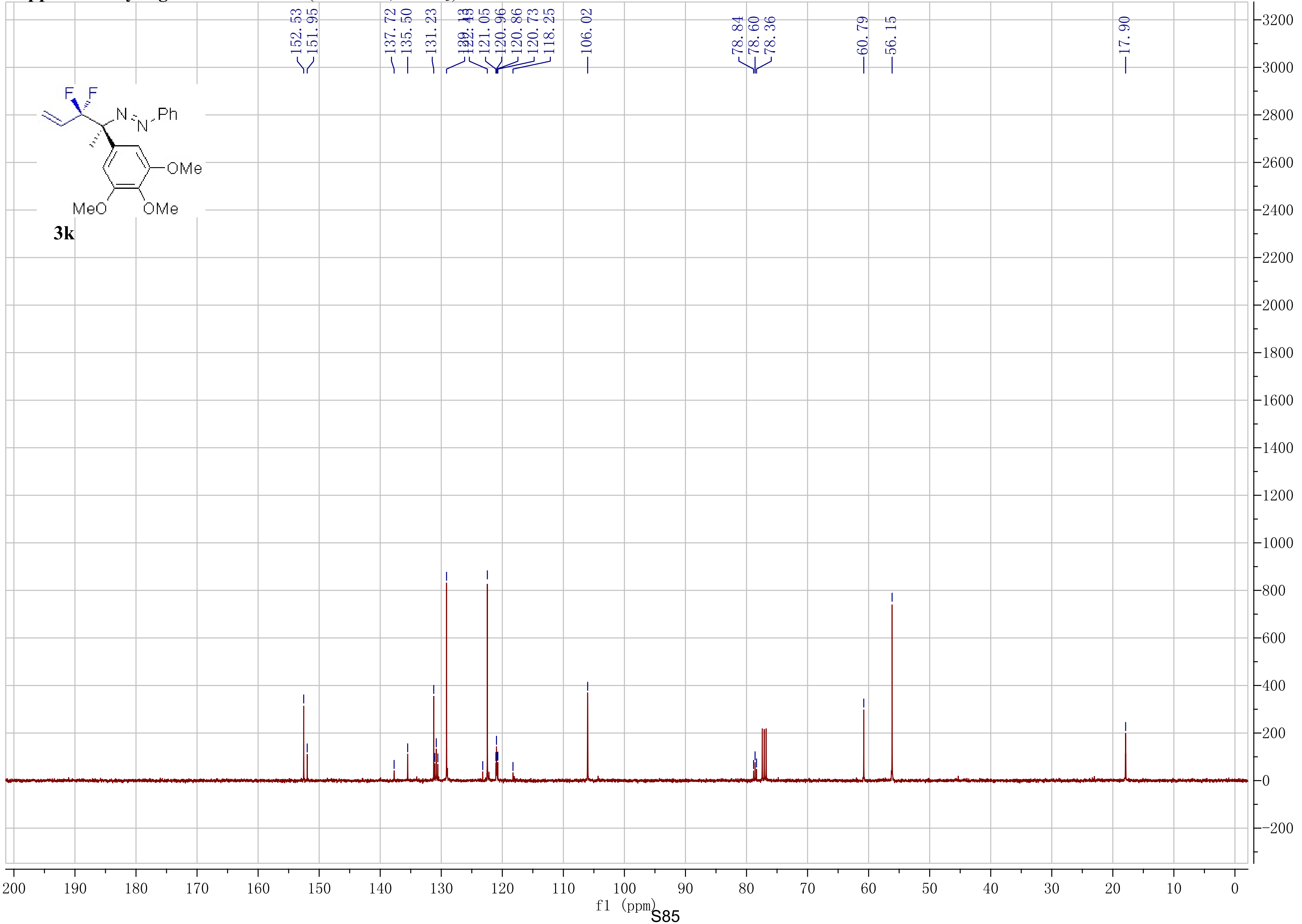

Supplementary Figure 56 ^{19}F NMR (376 MHz, $CDCl_3$) of **3k**

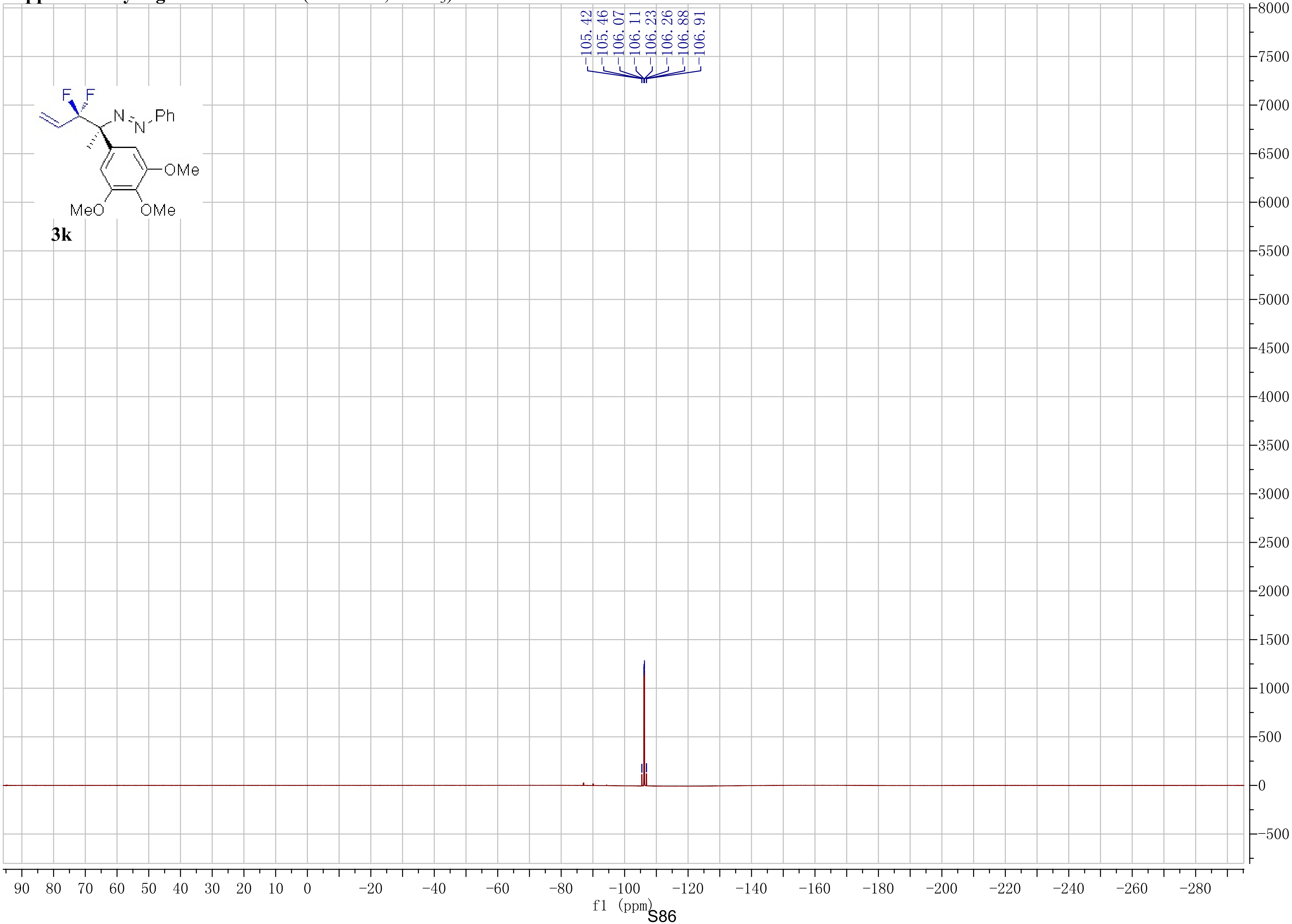

Supplementary Figure 57 HPLC spectra of racemic **3k**

Chromatogram and Results

Injection Details

Injection Name:	**HS-13-49-5+- PC3 A7W3 214 0.7**	*Run Time (min):*	**8.96**
Vial Number:	**RE2**	*Injection Volume:*	**5.00**
Injection Type:	**Unknown**	*Channel:*	**UV_VIS_1**
Calibration Level:		*Wavelength:*	**214.0**
Instrument Method:	**20160223-DAD3**	*Bandwidth:*	**4**
Processing Method:	**20160223**	*Dilution Factor:*	**1.0000**
Injection Date/Time:	**04/06/20 09:17**	*Sample Weight:*	**1.0000**

Chromatogram

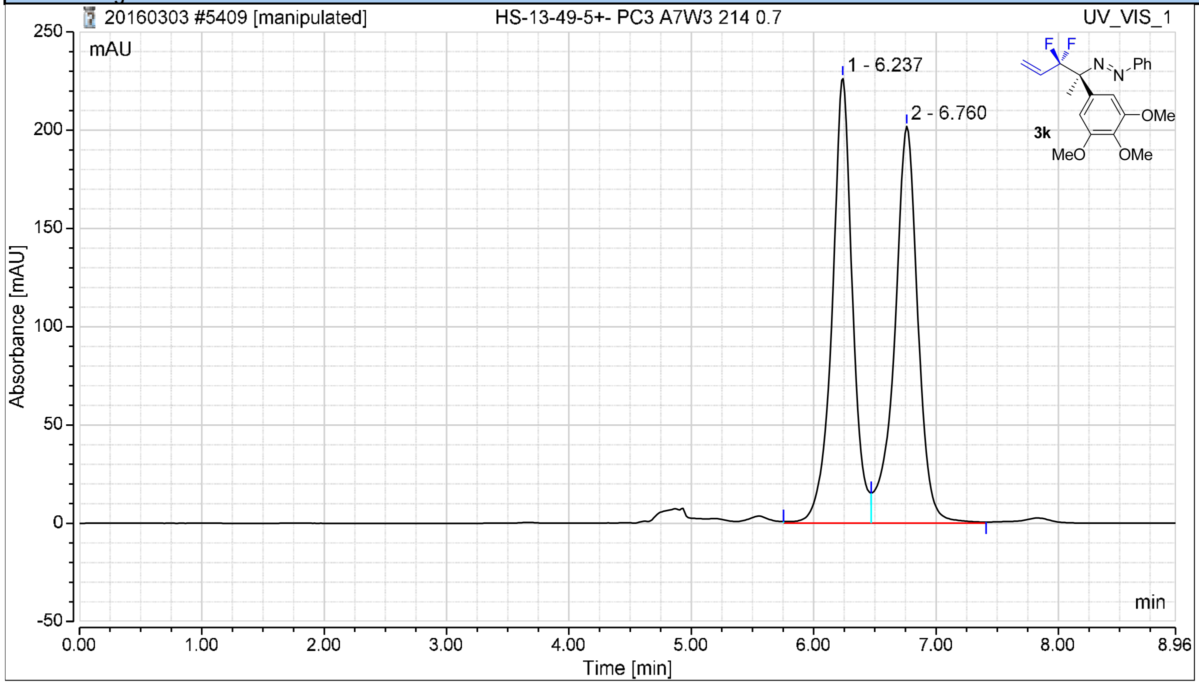

Integration Results

No.	Retention Time min	Area mAU*min	Height mAU	Relative Area %
1	6.237	43.4501	226.4544	49.391
2	6.760	44.5224	201.9036	50.609
**Total:**		**87.973**	**1401.998**	**100.000**

Supplementary Figure 58 HPLC spectra of (*S*)-**3k**

Chromatogram and Results

Injection Details

Injection Name:	**HS-13-69-4 PC3 A7W3 214 0.7**	*Run Time (min):*	**19.48**
Vial Number:	**RD1**	*Injection Volume:*	**5.00**
Injection Type:	**Unknown**	*Channel:*	**UV_VIS_1**
Calibration Level:		*Wavelength:*	**214.0**
Instrument Method:	**20160223-DAD3**	*Bandwidth:*	**4**
Processing Method:	**20160223**	*Dilution Factor:*	**1.0000**
Injection Date/Time:	**04/06/20 08:54**	*Sample Weight:*	**1.0000**

Chromatogram

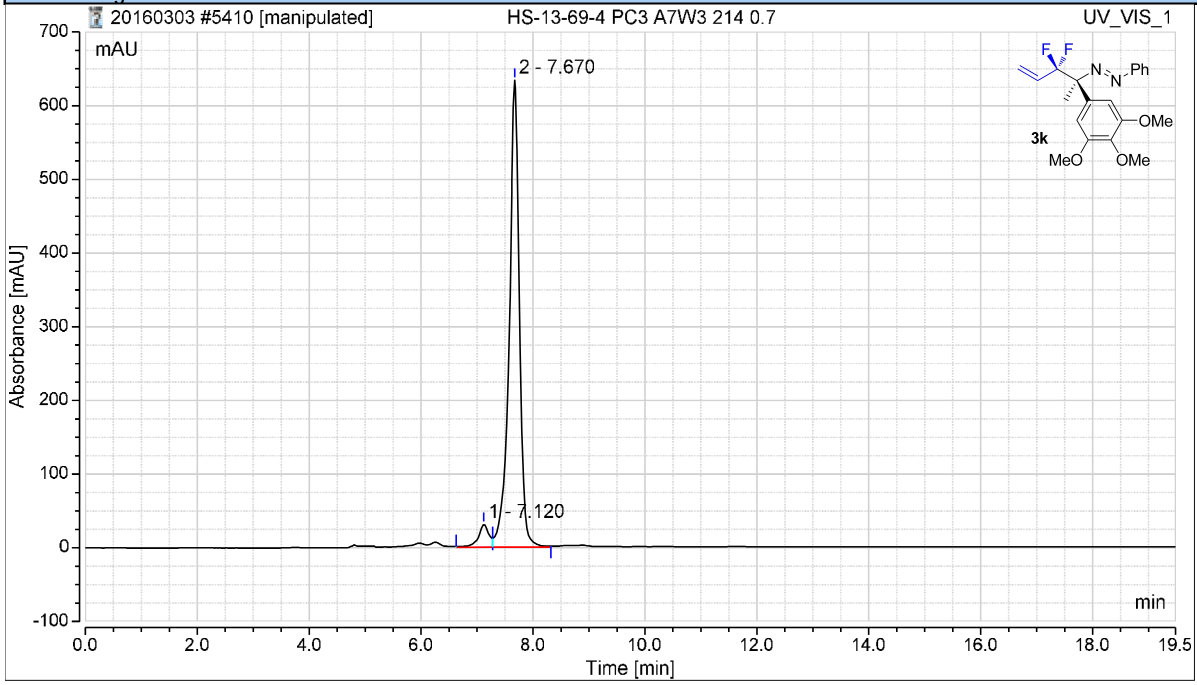

Integration Results

No.	Retention Time min	Area mAU*min	Height mAU	Relative Area %
1	7.120	6.9097	30.9927	4.952
2	7.670	132.6244	633.4767	95.048
**Total:**		**139.534**	**1401.998**	**100.000**

Supplementary Figure 59 ^{1}H NMR (400 MHz, $CDCl_3$) of **3l**

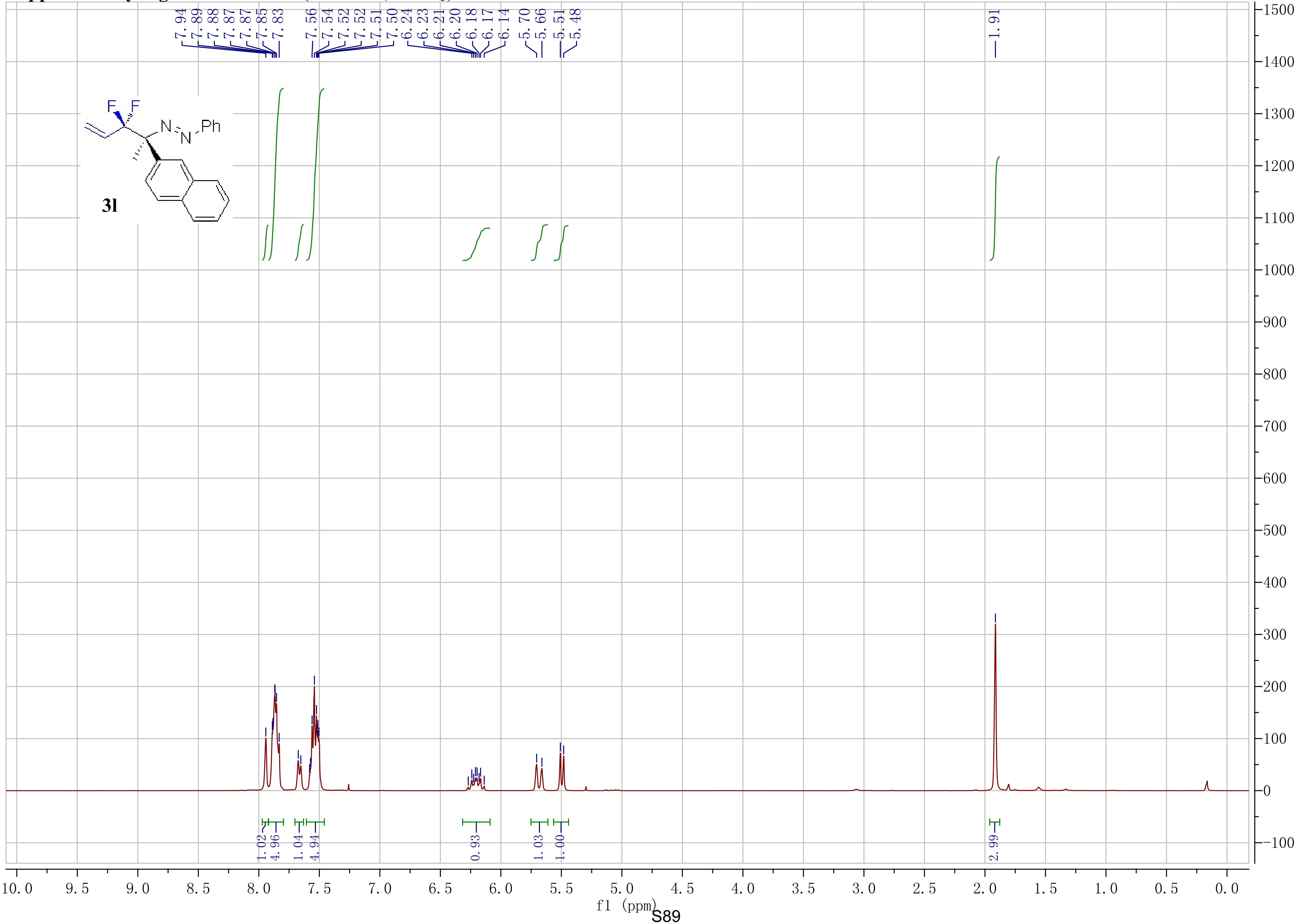

Supplementary Figure 60 ^{13}C NMR (101 MHz, $CDCl_3$) of **3l**

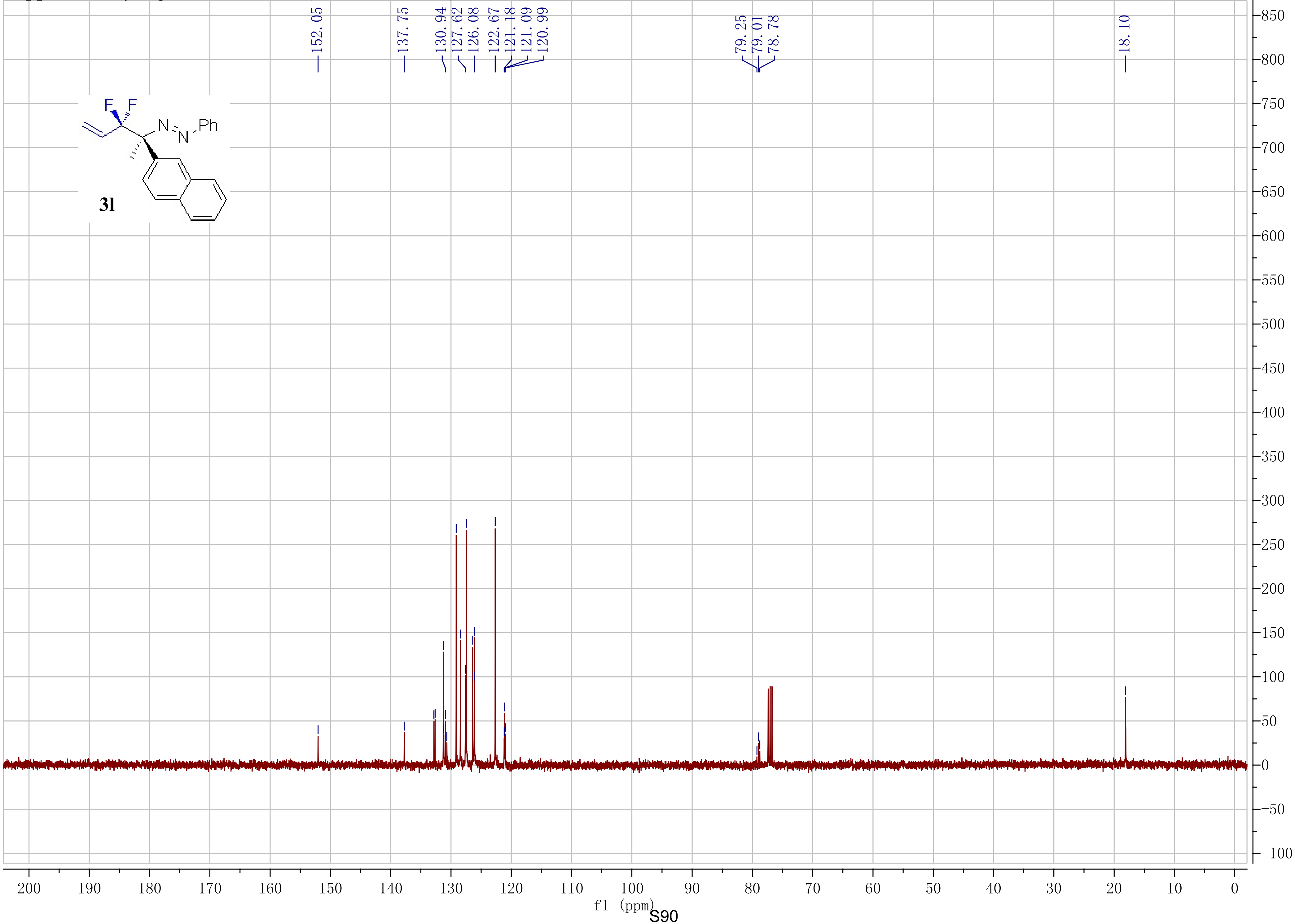

Supplementary Figure 61 ^{19}F NMR (376 MHz, $CDCl_3$) of **3l**

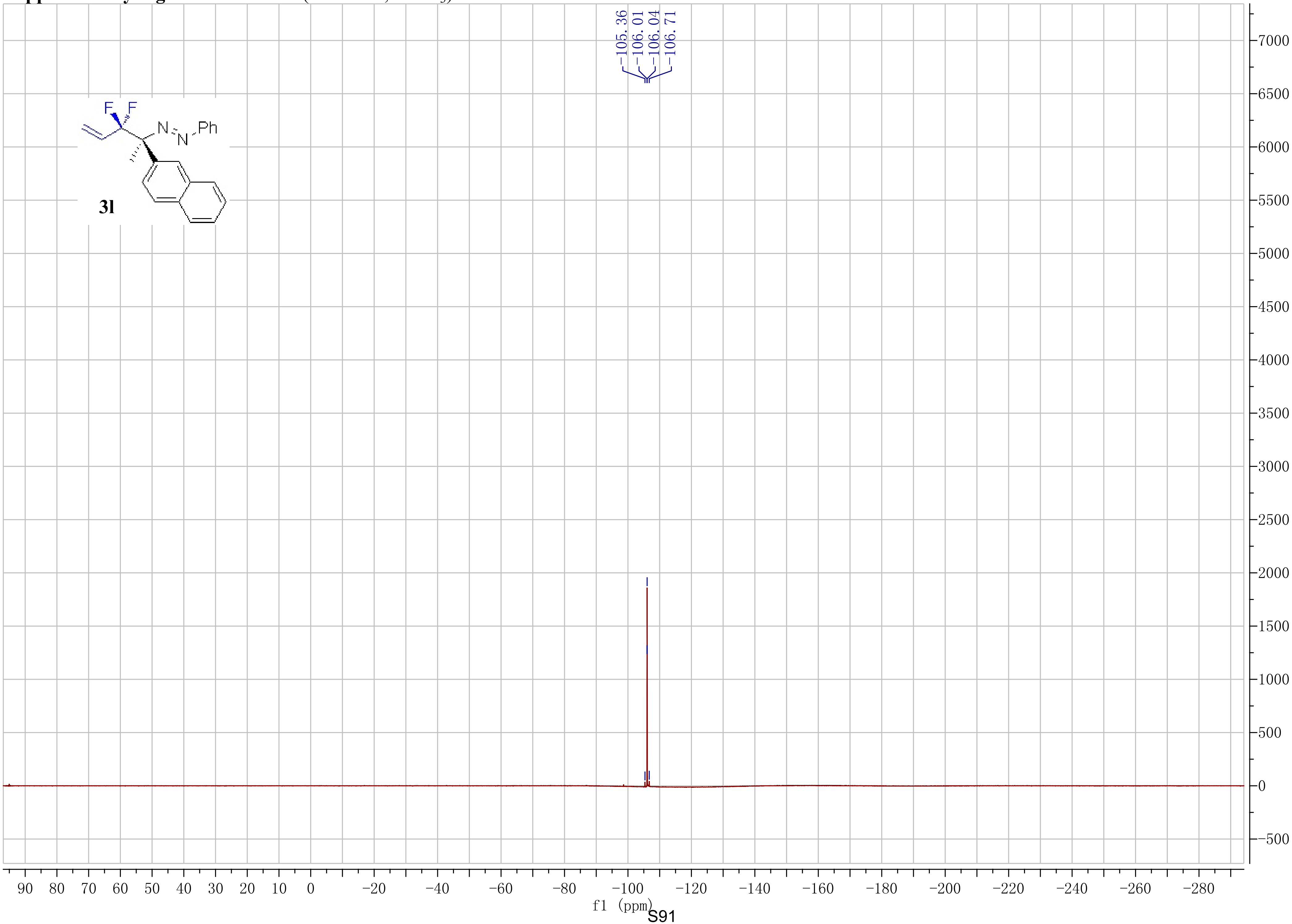

Supplementary Figure 62 HPLC spectra of racemic **3l**

Chromatogram and Results

Injection Details

Injection Name:	**HS-13-38-1+- PC3 A8W2 214 0.7**	*Run Time (min):*	**20.00**
Vial Number:	**RE3**	*Injection Volume:*	**2.00**
Injection Type:	**Unknown**	*Channel:*	**UV_VIS_1**
Calibration Level:		*Wavelength:*	**214.0**
Instrument Method:	**20160223-DAD3**	*Bandwidth:*	**4**
Processing Method:	**20160223**	*Dilution Factor:*	**1.0000**
Injection Date/Time:	**06/05/20 16:48**	*Sample Weight:*	**1.0000**

Chromatogram

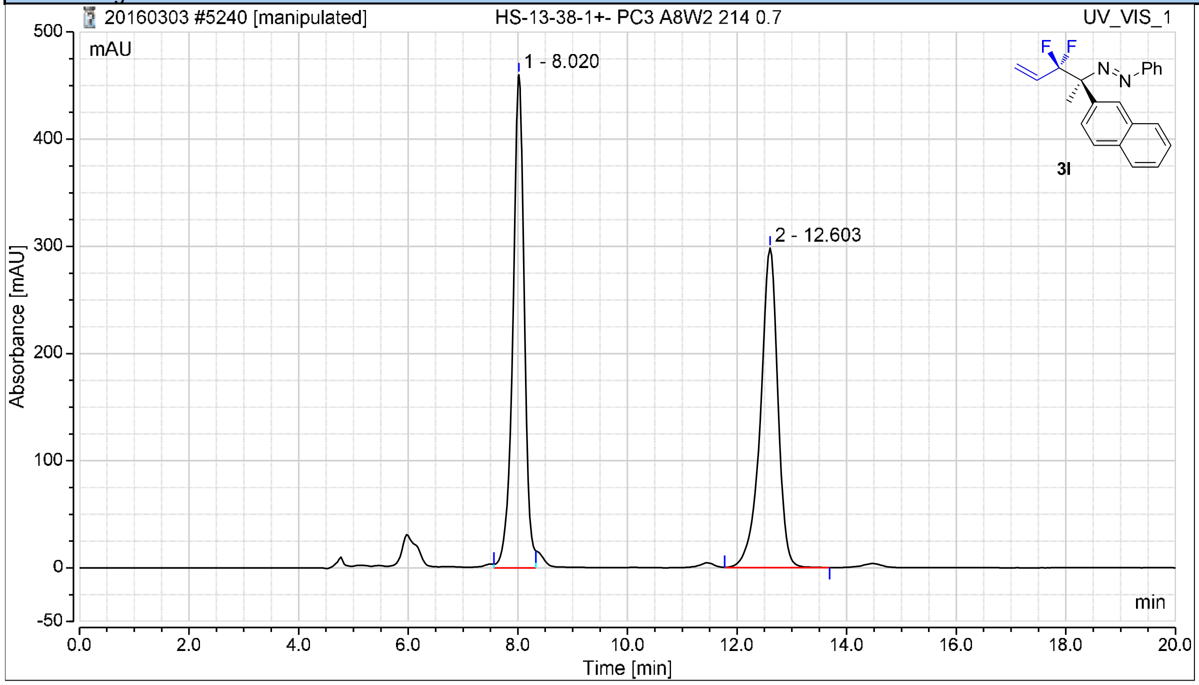

Integration Results

No.	Retention Time min	Area mAU*min	Height mAU	Relative Area %
1	8.020	111.0969	460.1900	50.281
2	12.603	109.8536	298.2120	49.719
**Total:**		**220.951**	**1401.998**	**100.000**

Supplementary Figure 63 HPLC spectra of (*S*)-**3l**

Chromatogram and Results

Injection Details

Injection Name:	**HS-13-56-9 PC3 A8W2 214 0.7**	*Run Time (min):*	**20.00**
Vial Number:	**RE4**	*Injection Volume:*	**2.00**
Injection Type:	**Unknown**	*Channel:*	**UV_VIS_1**
Calibration Level:		*Wavelength:*	**214.0**
Instrument Method:	**20160223-DAD3**	*Bandwidth:*	**4**
Processing Method:	**20160223**	*Dilution Factor:*	**1.0000**
Injection Date/Time:	**06/05/20 17:09**	*Sample Weight:*	**1.0000**

Chromatogram

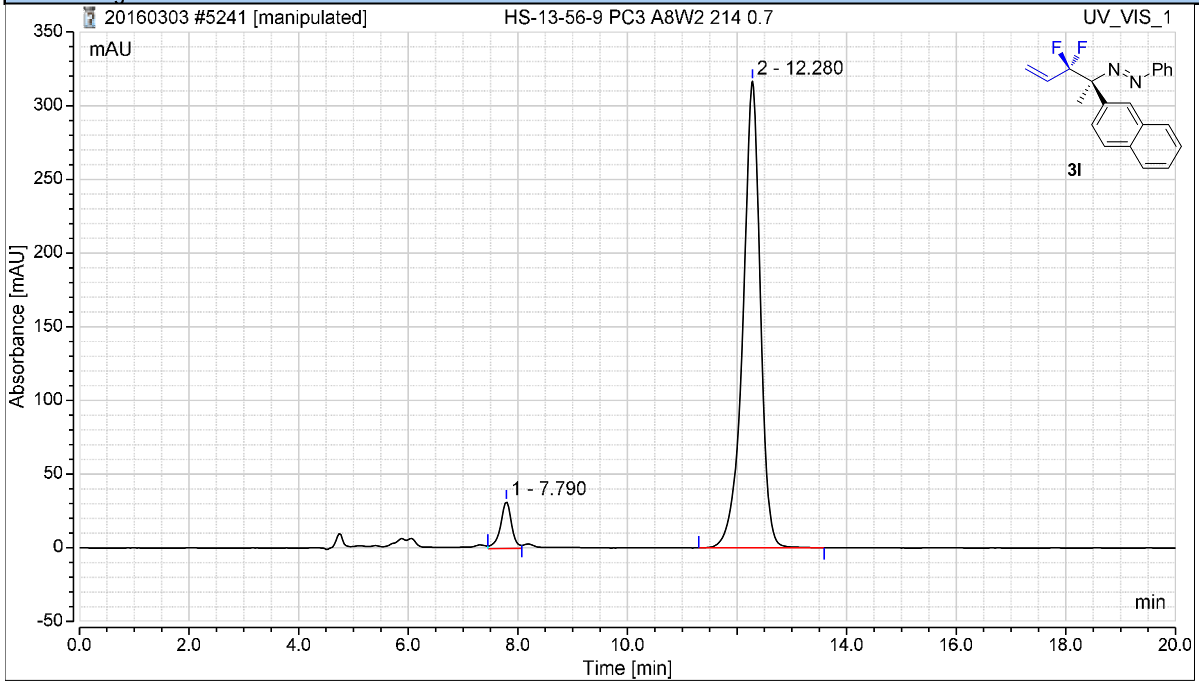

Integration Results

No.	Retention Time<br>min	Area<br>mAU*min	Height<br>mAU	Relative Area<br>%
1	7.790	7.5172	31.7281	6.093
2	12.280	115.8556	316.5406	93.907
**Total:**		**123.373**	**1401.998**	**100.000**

Supplementary Figure 64 ^{1}H NMR (400 MHz, $CDCl_3$) of **3m**

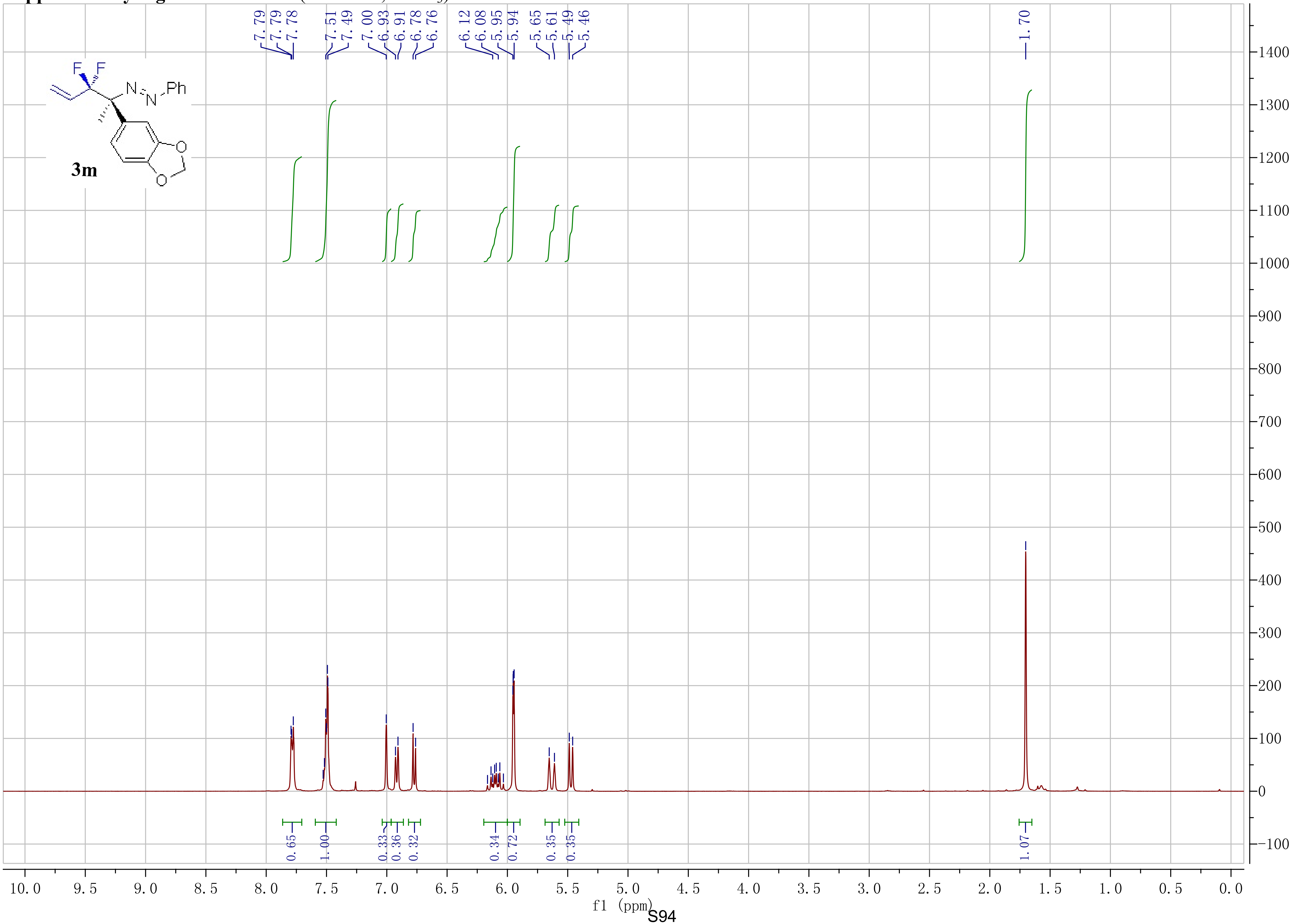

Supplementary Figure 65 ^{13}C NMR (101 MHz, $CDCl_3$) of **3m**

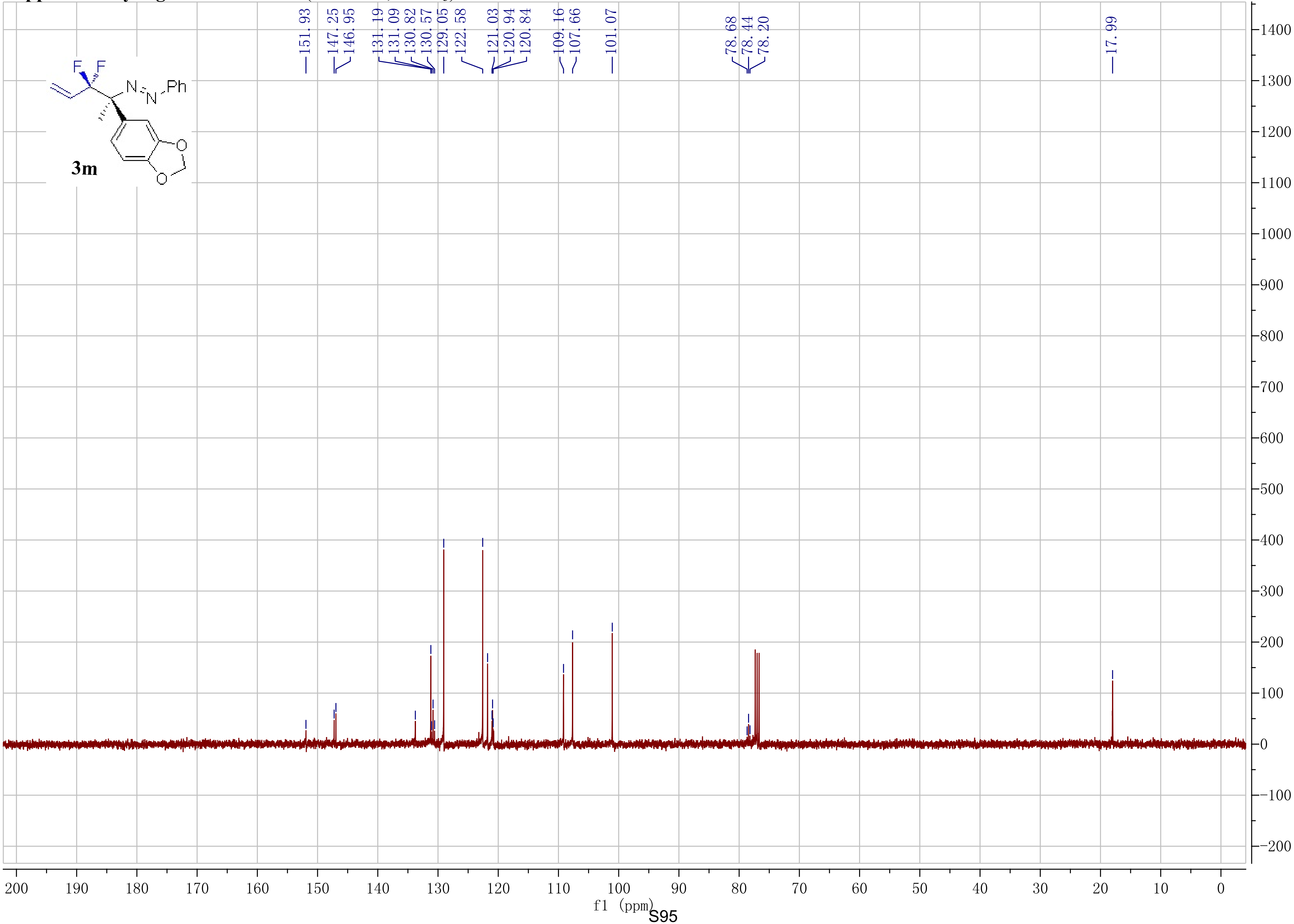

Supplementary Figure 66 ^{19}F NMR (376 MHz, $CDCl_3$) of **3m**

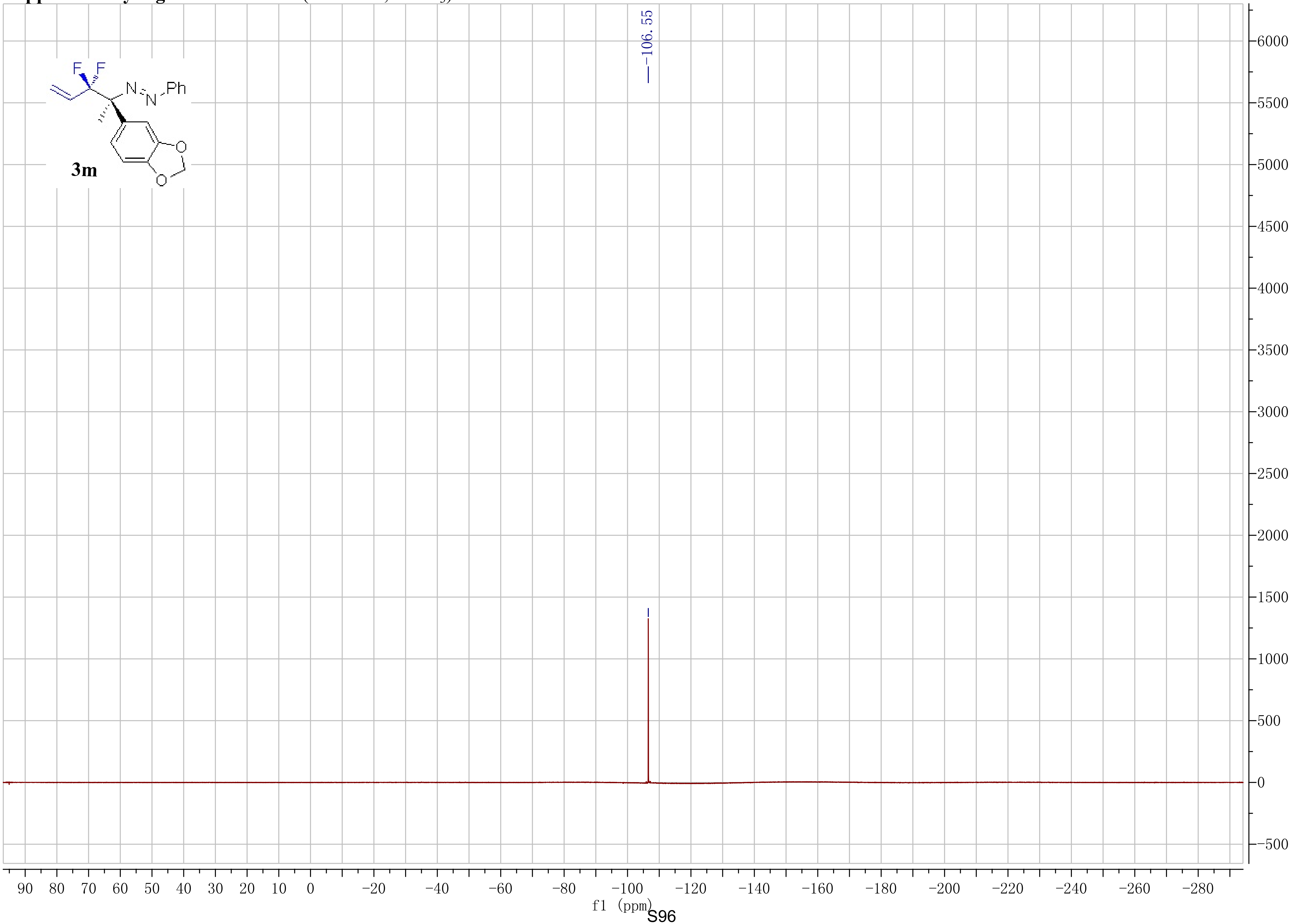

Supplementary Figure 67 HPLC spectra of racemic **3m**

8435 HS-13-38-2+- OJH 982 214 0.7

Sample Name:	**HS-13-38-2+- OJH 982 214 0.7**		**3.0**
Vial Number:	**RE4**	*Channel:*	**UV_VIS_2**
Sample Type:	**unknown**	*Wavelength:*	**214.0**
Control Program:	**test-dad3**	*Bandwidth:*	**4**
Quantif. Method:	**20170608**	*Dilution Factor:*	**1.0000**
Recording Time:	**2020-5-9 14:47**	*Sample Weight:*	**1.0000**
Run Time (min):	**36.34**	*Sample Amount:*	**1.0000**

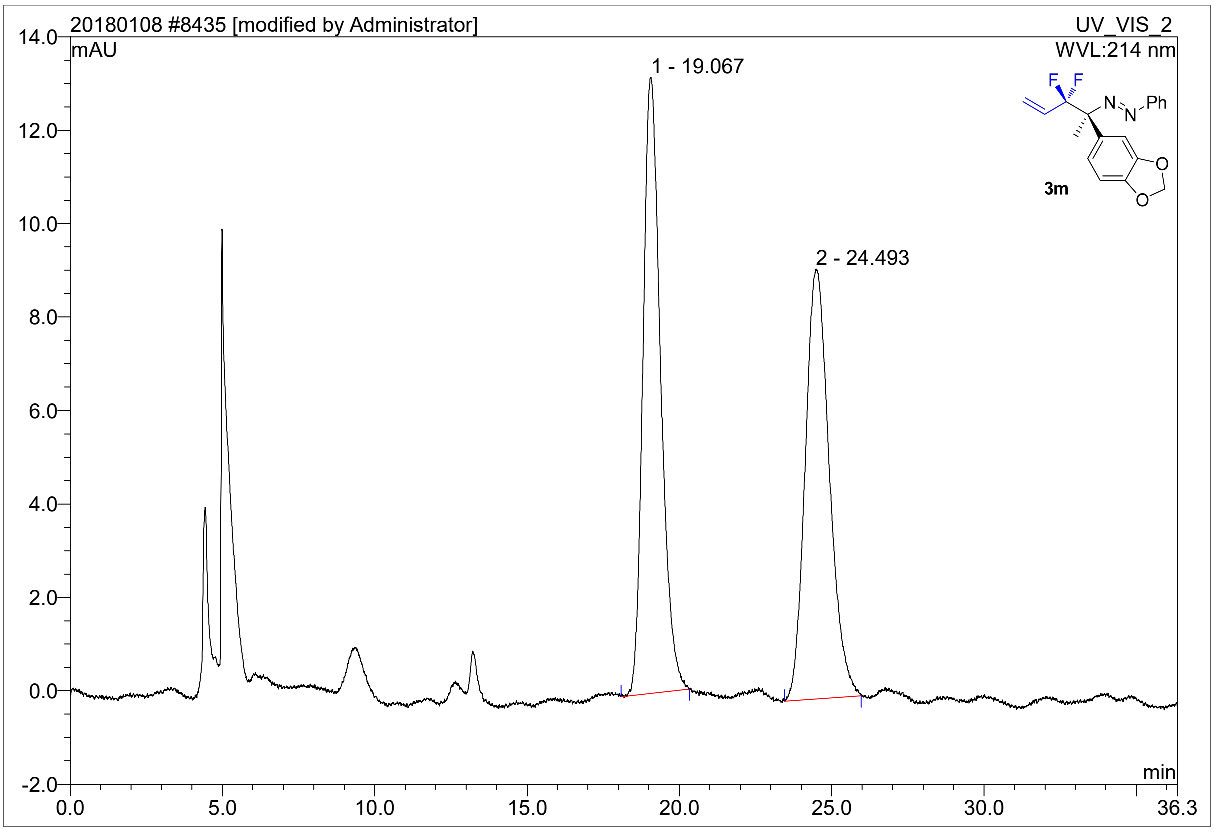

No.	Ret.Time min	Peak Name	Height mAU	Area mAU*min	Rel.Area %	Amount	Type
1	19.07	n.a.	13.188	8.869	51.21	n.a.	BMB*
2	24.49	n.a.	9.203	8.449	48.79	n.a.	BMB*
**Total:**			22.391	17.318	100.00	0.000	

Supplementary Figure 68 HPLC spectra of (*S*)-**3m**

8436 HS-13-62-4 OJH 982 214 0.7

Sample Name:	**HS-13-62-4 OJH 982 214 0.7**	*Injection Volume:*	**3.0**
Vial Number:	**RD4**	*Channel:*	**UV_VIS_2**
Sample Type:	**unknown**	*Wavelength:*	**214.0**
Control Program:	**test-dad3**	*Bandwidth:*	**4**
Quantif. Method:	**20170608**	*Dilution Factor:*	**1.0000**
Recording Time:	**2020-5-9 15:32**	*Sample Weight:*	**1.0000**
Run Time (min):	**32.54**	*Sample Amount:*	**1.0000**

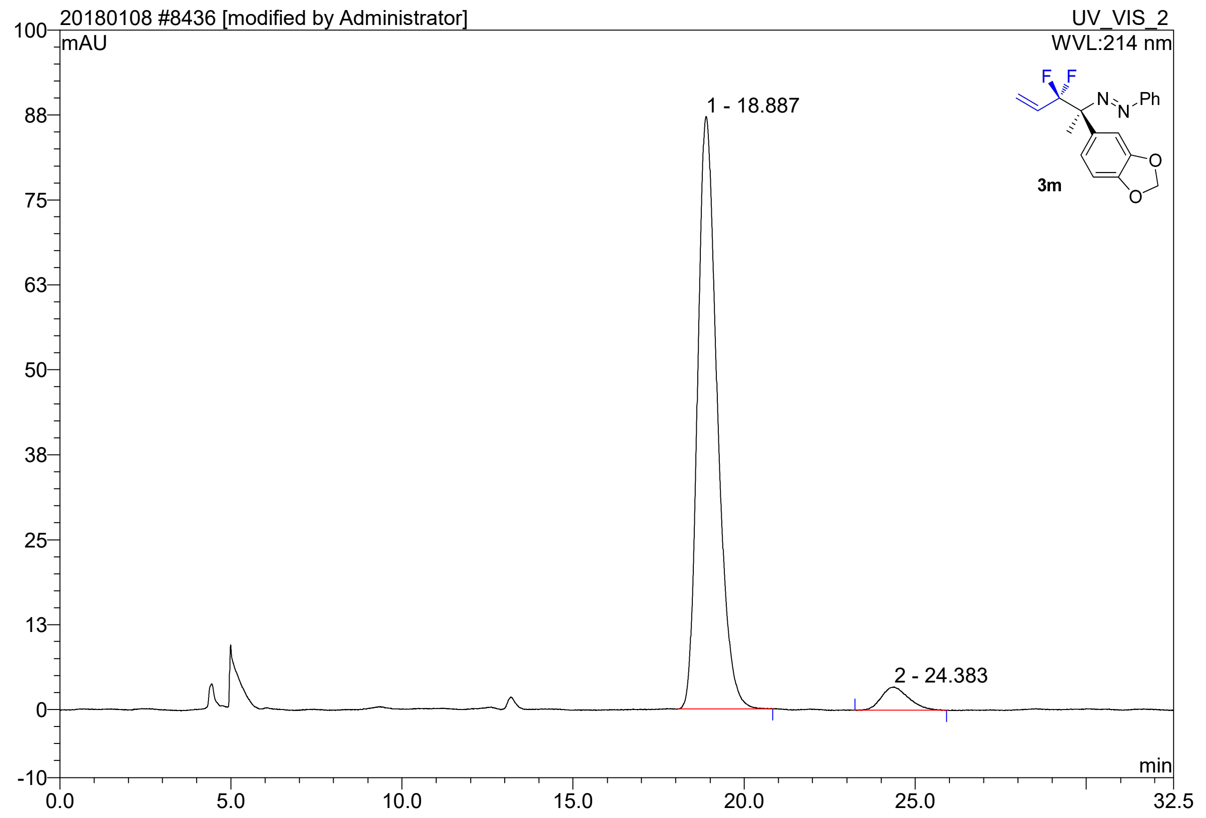

No.	Ret.Time min	Peak Name	Height mAU	Area mAU*min	Rel.Area %	Amount	Type
1	18.89	n.a.	87.174	57.917	94.85	n.a.	BMB*
2	24.38	n.a.	3.427	3.146	5.15	n.a.	BMB*
**Total:**			90.601	61.063	100.00	0.000	

Supplementary Figure 69 ^{1}H NMR (400 MHz, $CDCl_3$) of **3n**

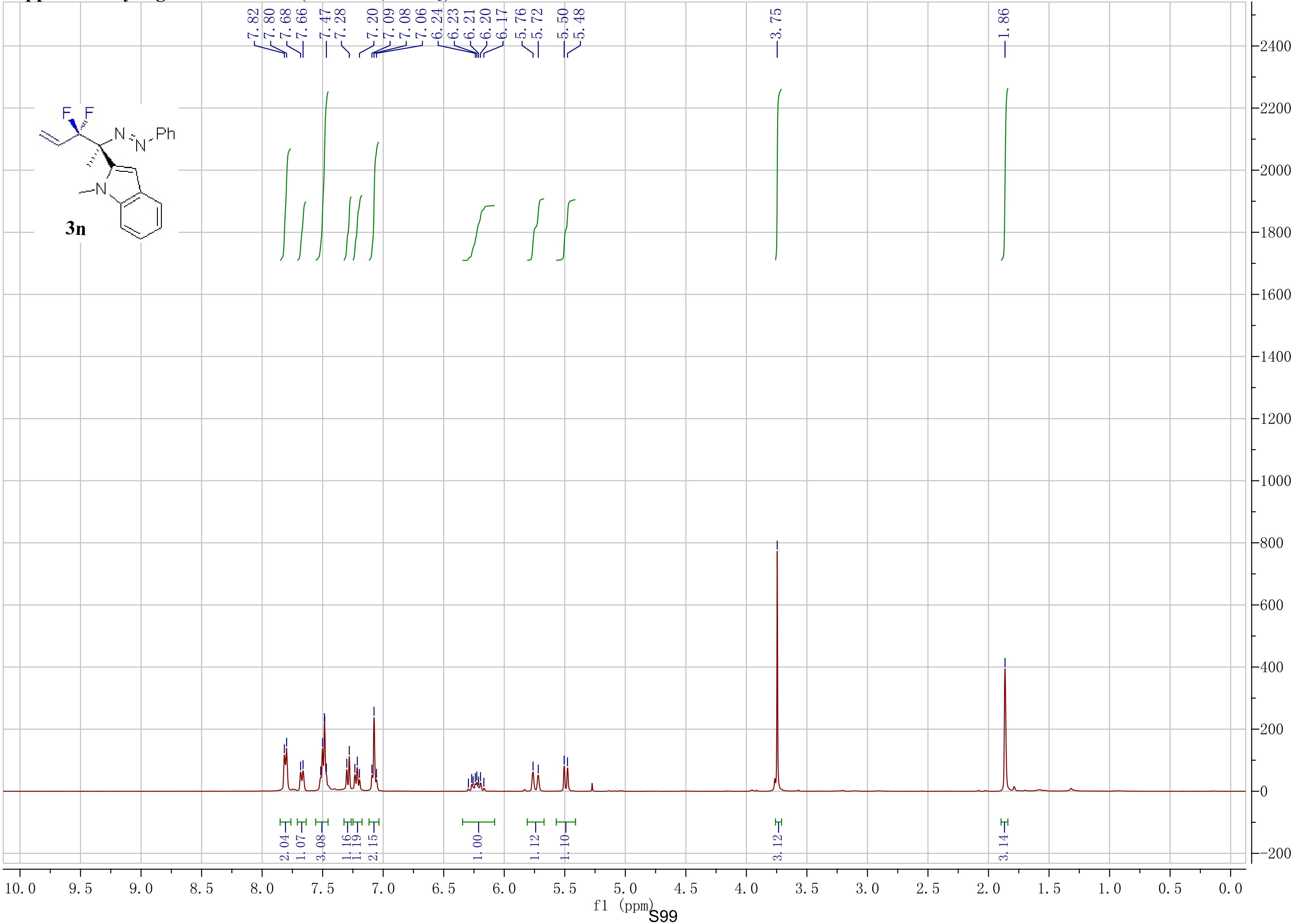

Supplementary Figure 70 ^{13}C NMR (101 MHz, $CDCl_3$) of **3n**

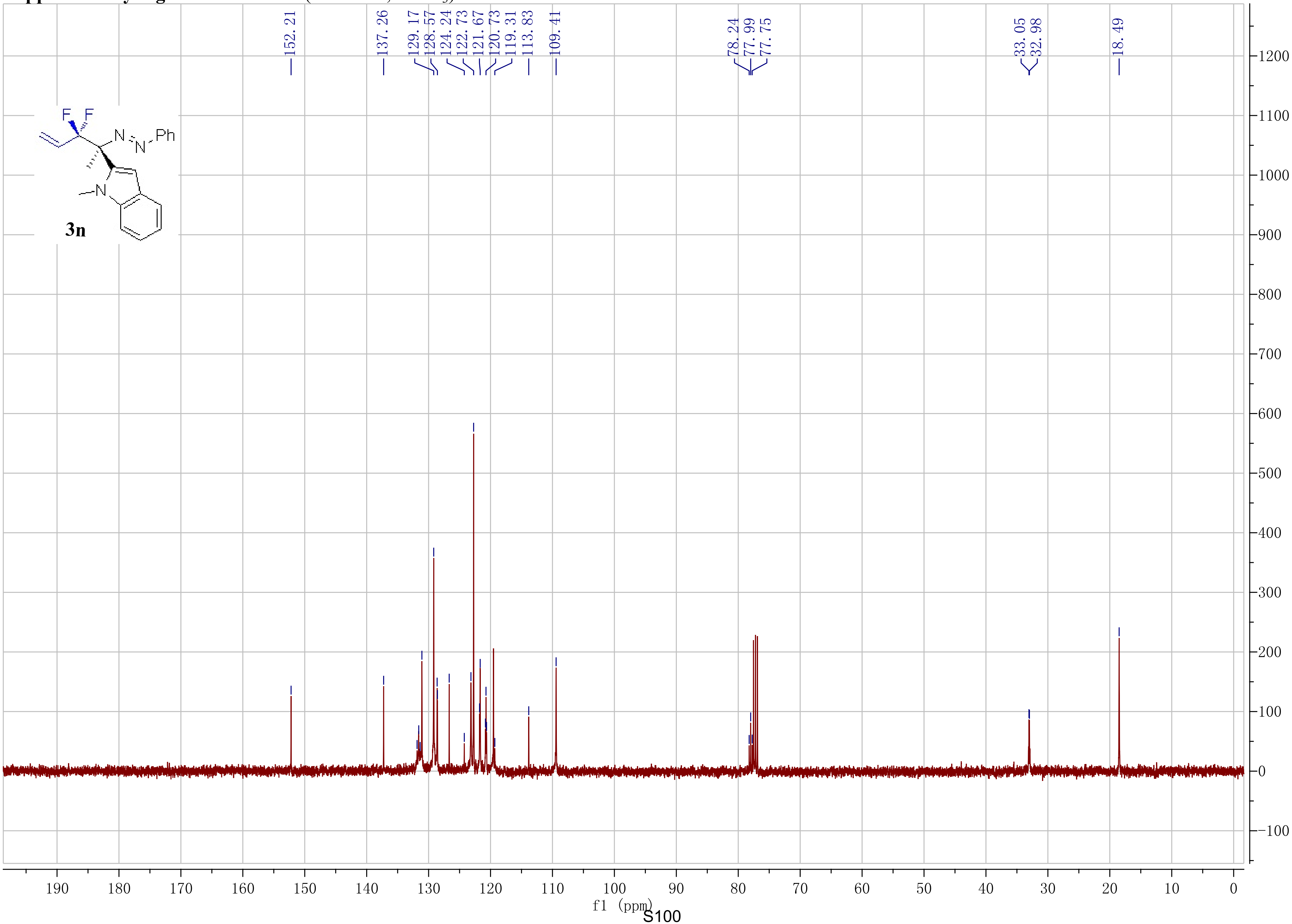

Supplementary Figure 71 ^{19}F NMR (376 MHz, $CDCl_3$) of **3n**

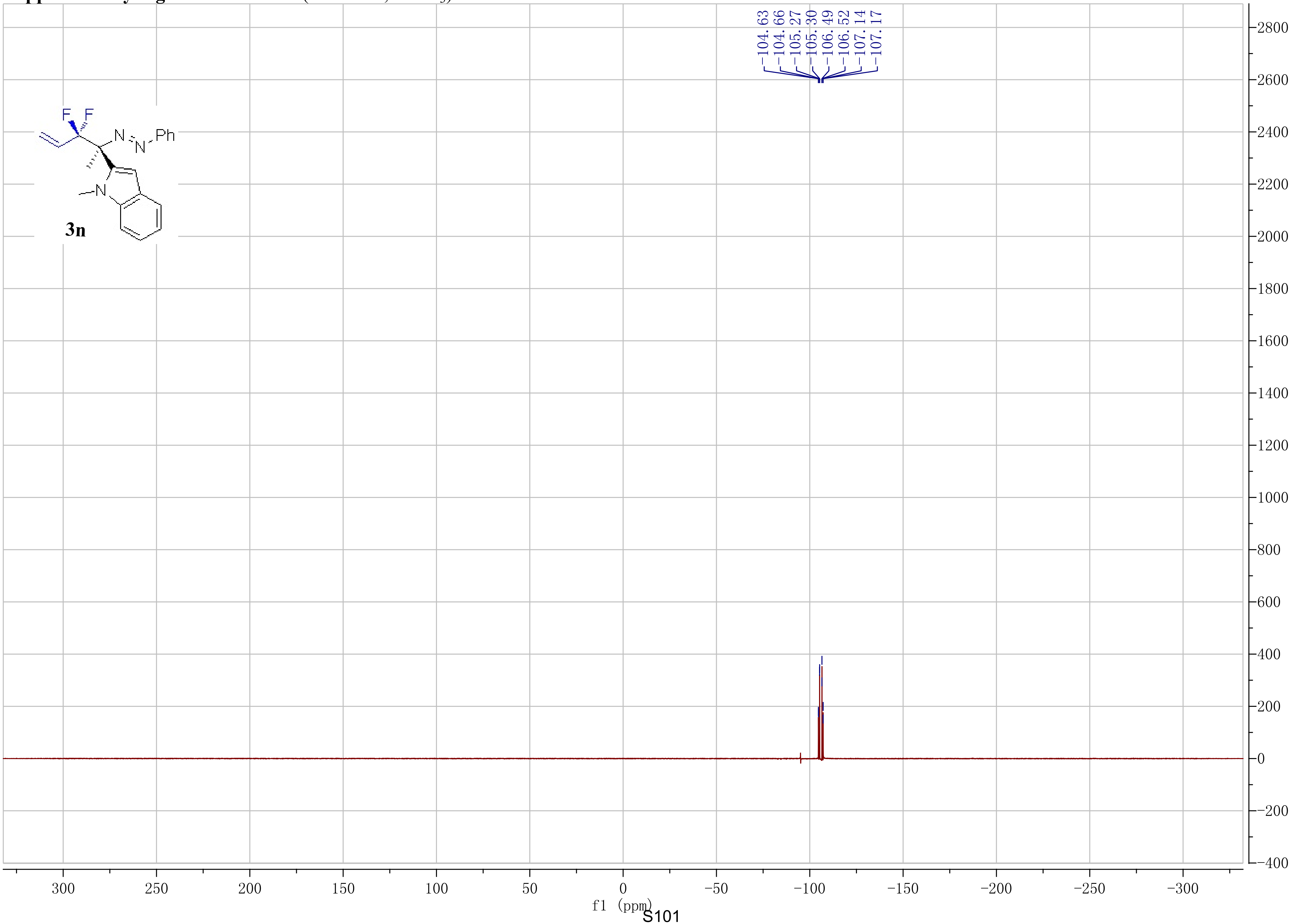

Supplementary Figure 72 HPLC spectra of racemic **3n**

Data File D:\CHEM32\1\DATA\YZR\202001000126.D
Sample Name: HS-13-46-8+- PC3 A6W4 214 0.7

===

Acq. Operator : YZR
Acq. Instrument : instr1   Location : Vial 15
Injection Date : 09/05/2020 15:44:05
Inj Volume : 3.000 µl
Acq. Method : D:\CHEM32\1\METHODS\YZR3.M
Last changed : 09/05/2020 15:14:14 by YZR
(modified after loading)
Analysis Method : D:\CHEM32\1\METHODS\YZR3.M
Last changed : 08/05/2020 08:37:34 by YZR
(modified after loading)
Additional Info : Peak(s) manually integrated

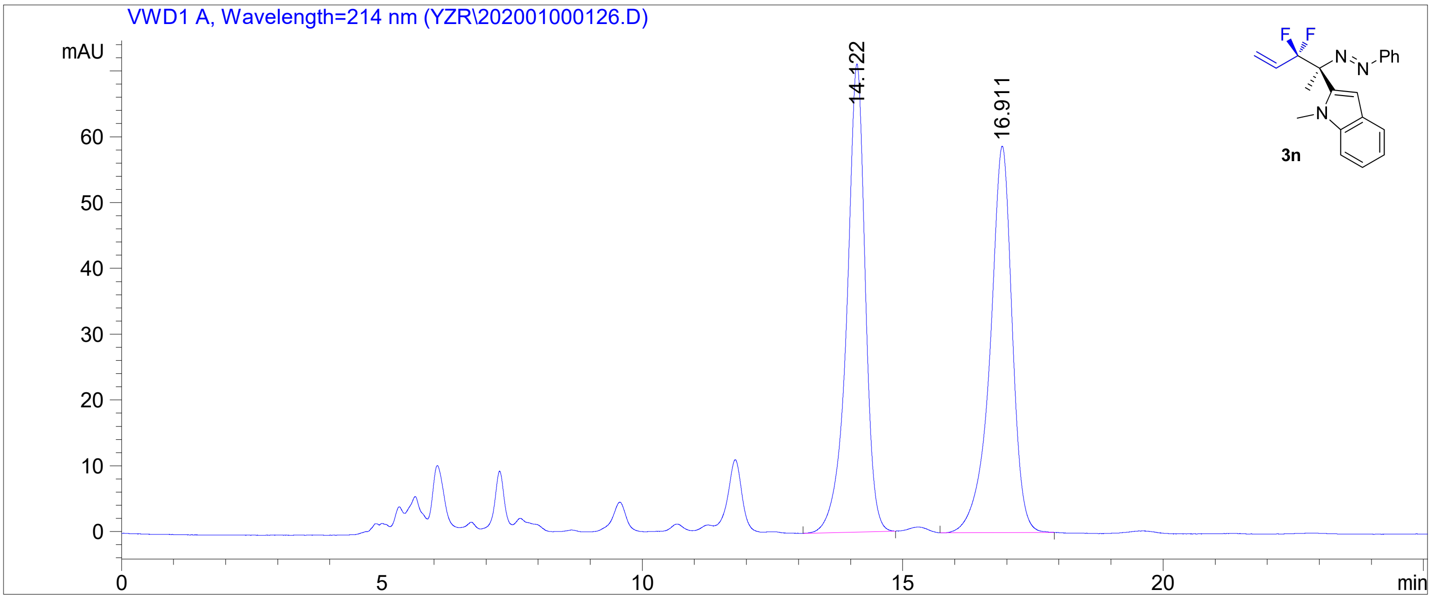

3n

===

Area Percent Report

===

Sorted By : Signal
Multiplier: : 1.0000
Dilution: : 1.0000
Use Multiplier & Dilution Factor with ISTDs

Signal 1: VWD1 A, Wavelength=214 nm

Peak #	RetTime [min]	Type	Width [min]	Area [mAU*s]	Height [mAU]	Area %
1	14.122	BB	0.3660	1750.21362	71.13828	50.1412
2	16.911	BB	0.4407	1740.35303	58.73945	49.8588

Totals : 3490.56665 129.87774

===

*** End of Report ***

Supplementary Figure 73 HPLC spectra of (*S*)-**3n**

```
Data File D:\CHEM32\1\DATA\YZR\202001000127.D
Sample Name: HS-13-62-3 PC3 A6W4 214 0.7

=====================================================================
Acq. Operator   : YZR
Acq. Instrument : instr1                          Location : Vial 16
Injection Date  : 09/05/2020 16:11:00
                                                Inj Volume : 3.000 µl
Acq. Method     : D:\CHEM32\1\METHODS\YZR3.M
Last changed    : 09/05/2020 16:09:32 by YZR
                  (modified after loading)
Analysis Method : D:\CHEM32\1\METHODS\YZR3.M
Last changed    : 08/05/2020 08:37:34 by YZR
                  (modified after loading)
Additional Info : Peak(s) manually integrated
```

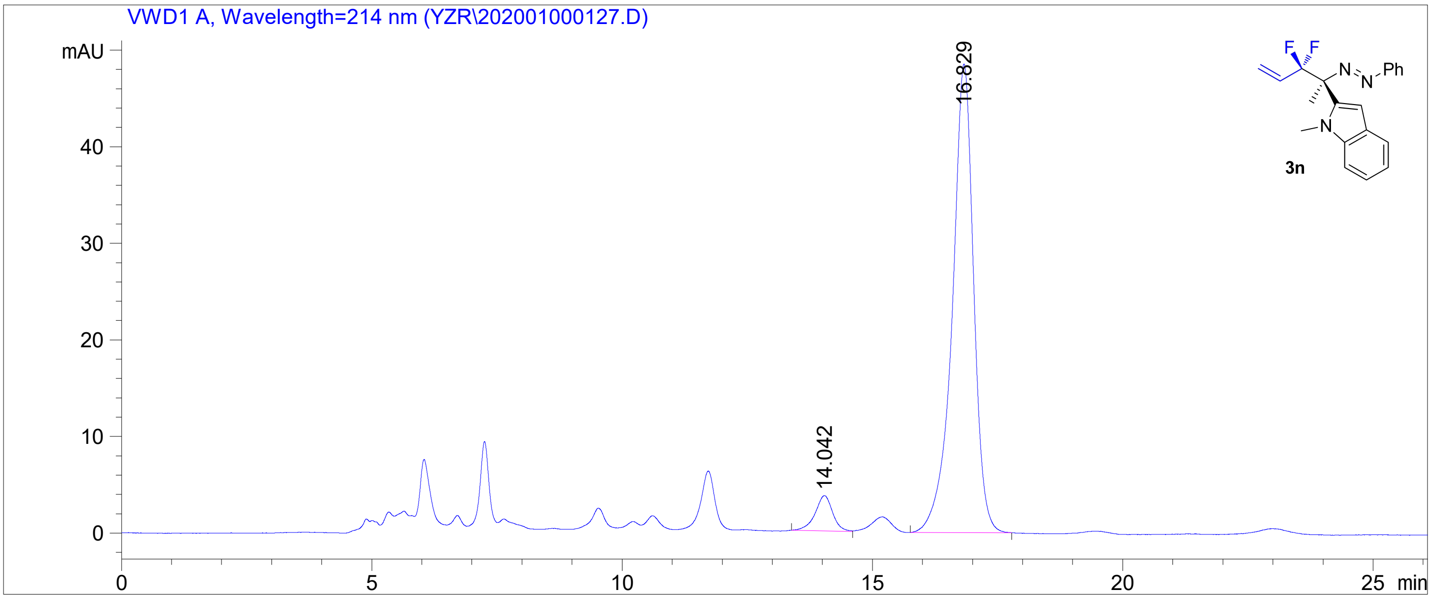


```
=====================================================================
                         Area Percent Report
=====================================================================

Sorted By             :      Signal
Multiplier:                    :      1.0000
Dilution:                      :      1.0000
Use Multiplier & Dilution Factor with ISTDs


Signal 1: VWD1 A, Wavelength=214 nm
```

Peak #	RetTime [min]	Type	Width [min]	Area [mAU*s]	Height [mAU]	Area %
1	14.042	BV	0.3383	89.06055	3.65448	5.8305
2	16.829	BB	0.4438	1438.44446	48.53432	94.1695

Totals : 1527.50500 52.18880

```
=====================================================================
                         *** End of Report ***
```

Supplementary Figure 74 ^{1}H NMR (400 MHz, $CDCl_3$) of **3o**

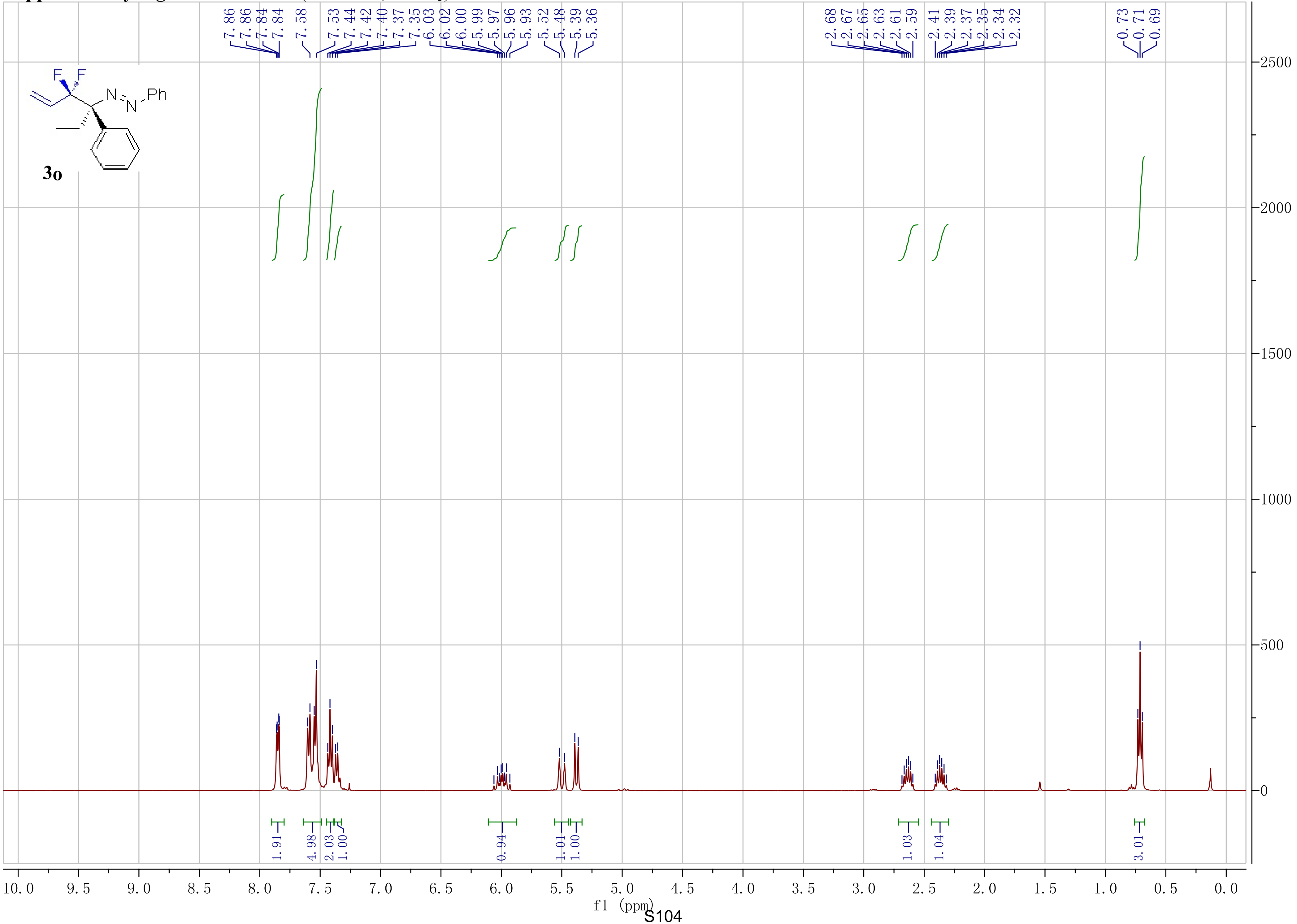

Supplementary Figure 75 ^{13}C NMR (101 MHz, $CDCl_3$) of **3o**

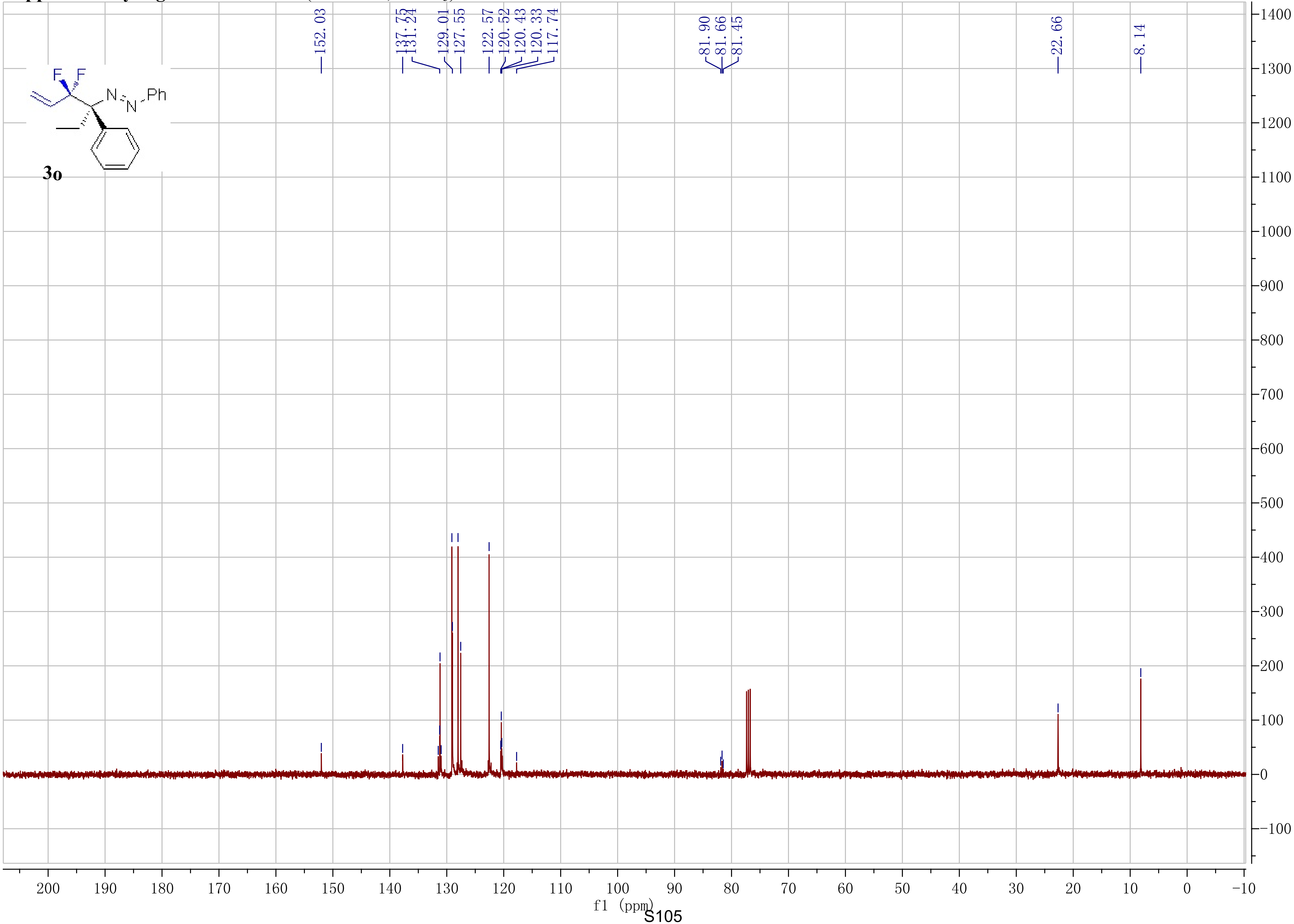

Supplementary Figure 76 ^{19}F NMR (376 MHz, $CDCl_3$) of **3o**

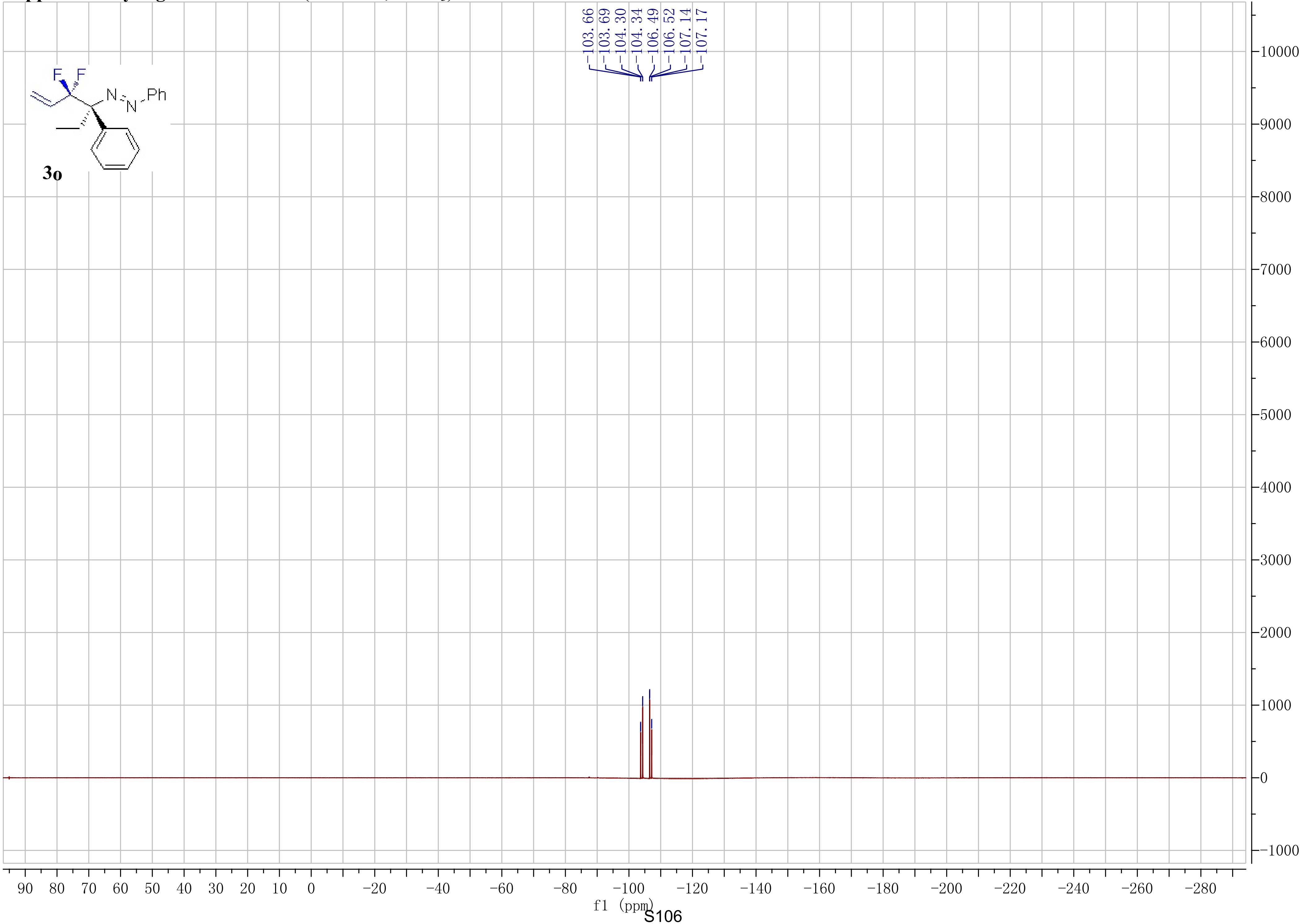

Supplementary Figure 77 HPLC spectra of racemic **3o**

8431 HS-13-38-5+- OJH 982 214 0.7

Sample Name:	**HS-13-38-5+- OJH 982 214 0.7**		**3.0**
Vial Number:	**RE6**	*Channel:*	**UV_VIS_2**
Sample Type:	**unknown**	*Wavelength:*	**214.0**
Control Program:	**test-dad3**	*Bandwidth:*	**4**
Quantif. Method:	**20170608**	*Dilution Factor:*	**1.0000**
Recording Time:	**2020-5-9 12:40**	*Sample Weight:*	**1.0000**
Run Time (min):	**20.00**	*Sample Amount:*	**1.0000**

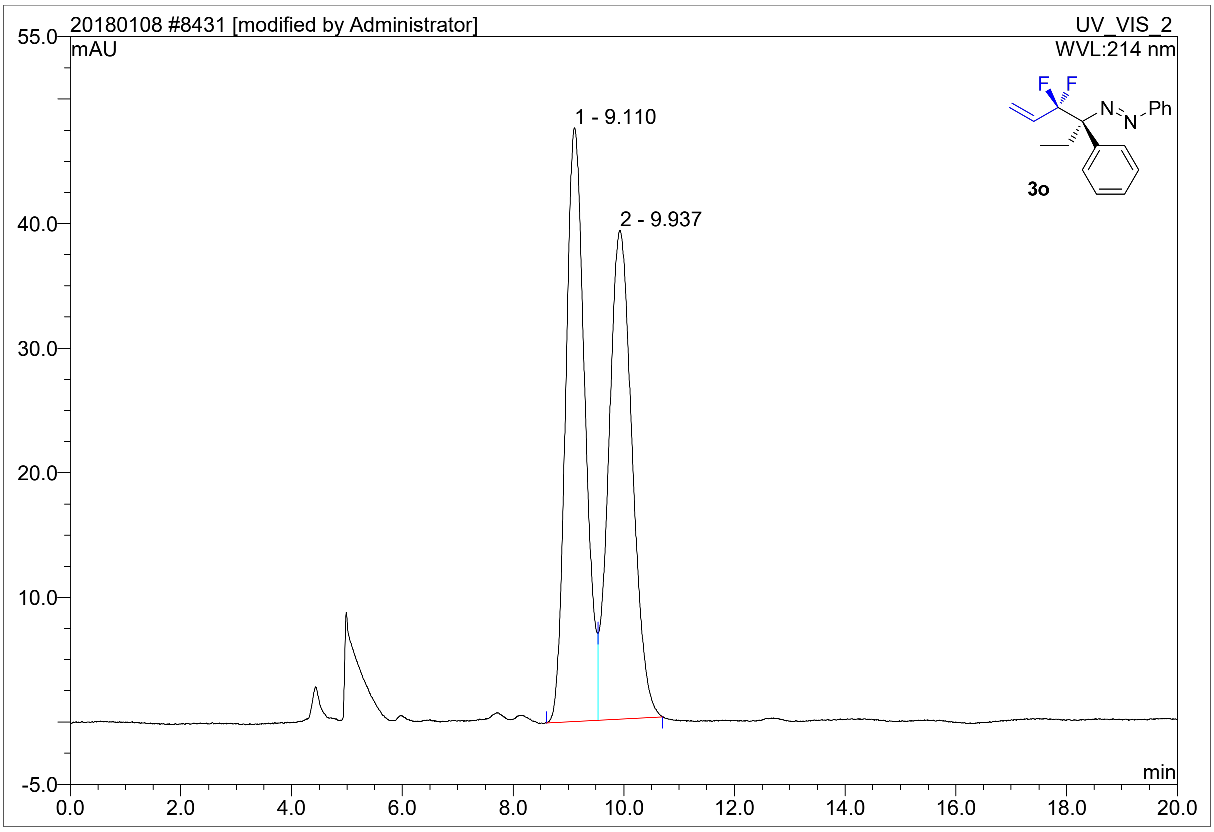

No.	Ret.Time min	Peak Name	Height mAU	Area mAU*min	Rel.Area %	Amount	Type
1	9.11	n.a.	47.659	19.169	49.82	n.a.	BM
2	9.94	n.a.	39.231	19.304	50.18	n.a.	MB
**Total:**			86.890	38.473	100.00	0.000	

default/Integration

Chromeleon (c) Dionex 1996-2006
Version 6.80 SR14 Build 4527 (238909)

Supplementary Figure 78 HPLC spectra of (*S*)-**3o**

Operator:Administrator Timebase:HPLC Sequence:20180108

8432 HS-13-56-8 OJH 982 214 0.7

Sample Name:	**HS-13-56-8 OJH 982 214 0.7**	*Injection Volume:*	**3.0**
Vial Number:	**RD6**	*Channel:*	**UV_VIS_2**
Sample Type:	**unknown**	*Wavelength:*	**214.0**
Control Program:	**test-dad3**	*Bandwidth:*	**4**
Quantif. Method:	**20170608**	*Dilution Factor:*	**1.0000**
Recording Time:	**2020-5-9 13:02**	*Sample Weight:*	**1.0000**
Run Time (min):	**20.00**	*Sample Amount:*	**1.0000**

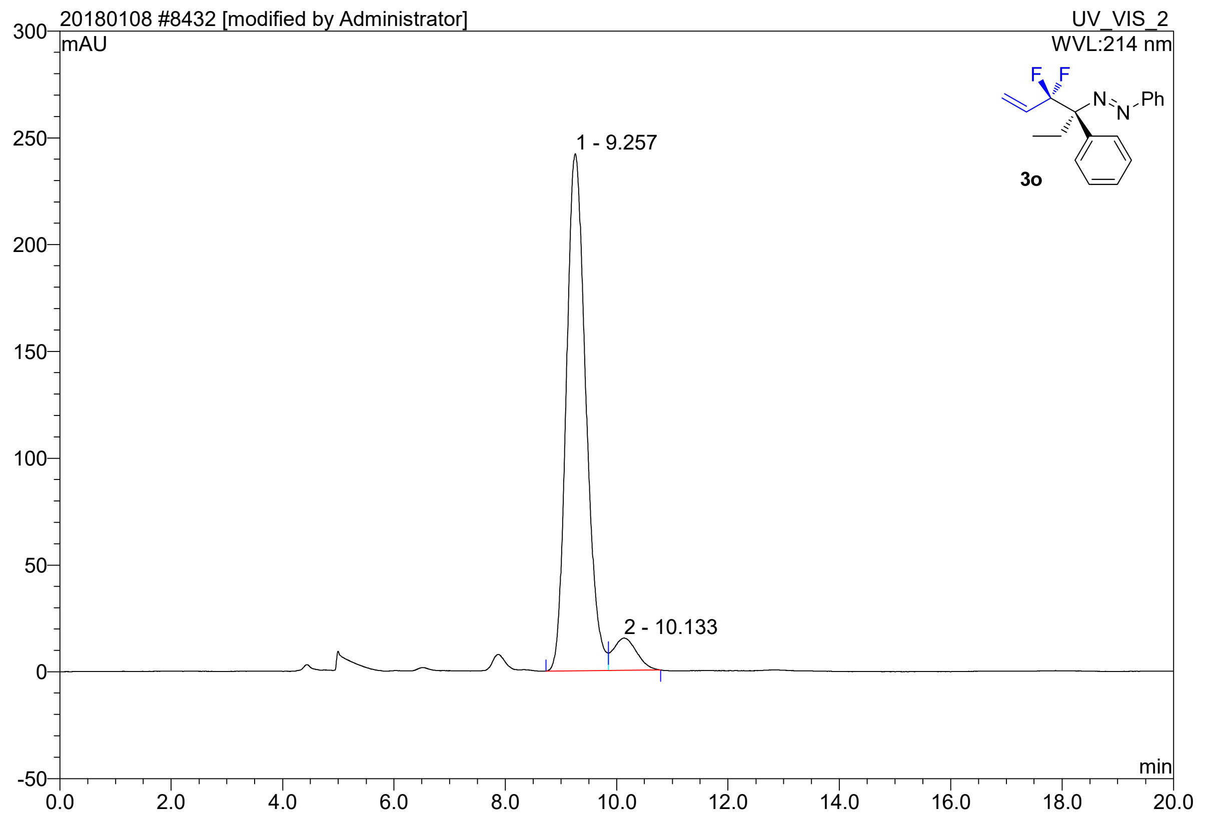

No.	Ret.Time min	Peak Name	Height mAU	Area mAU*min	Rel.Area %	Amount	Type
1	9.26	n.a.	242.198	98.630	93.06	n.a.	BM
2	10.13	n.a.	15.159	7.351	6.94	n.a.	MB
**Total:**			257.358	105.981	100.00	0.000	

default/Integration

Chromeleon (c) Dionex 1996-2006
Version 6.80 SR14 Build 4527 (238909)

Supplementary Figure 79 ^{1}H NMR (400 MHz, $CDCl_3$) of **3p**

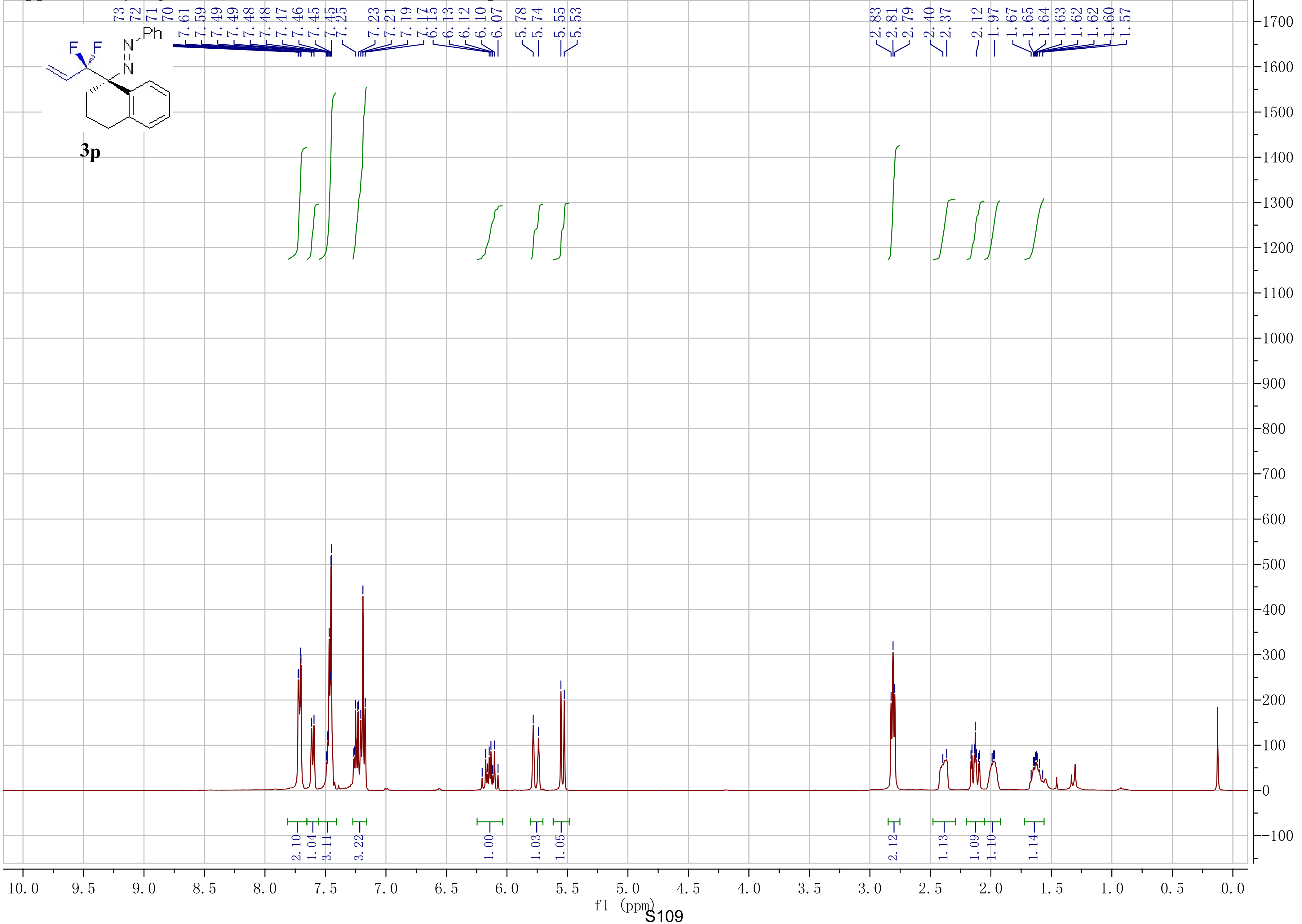

Supplementary Figure 80 ^{13}C NMR (101 MHz, $CDCl_3$) of **3p**

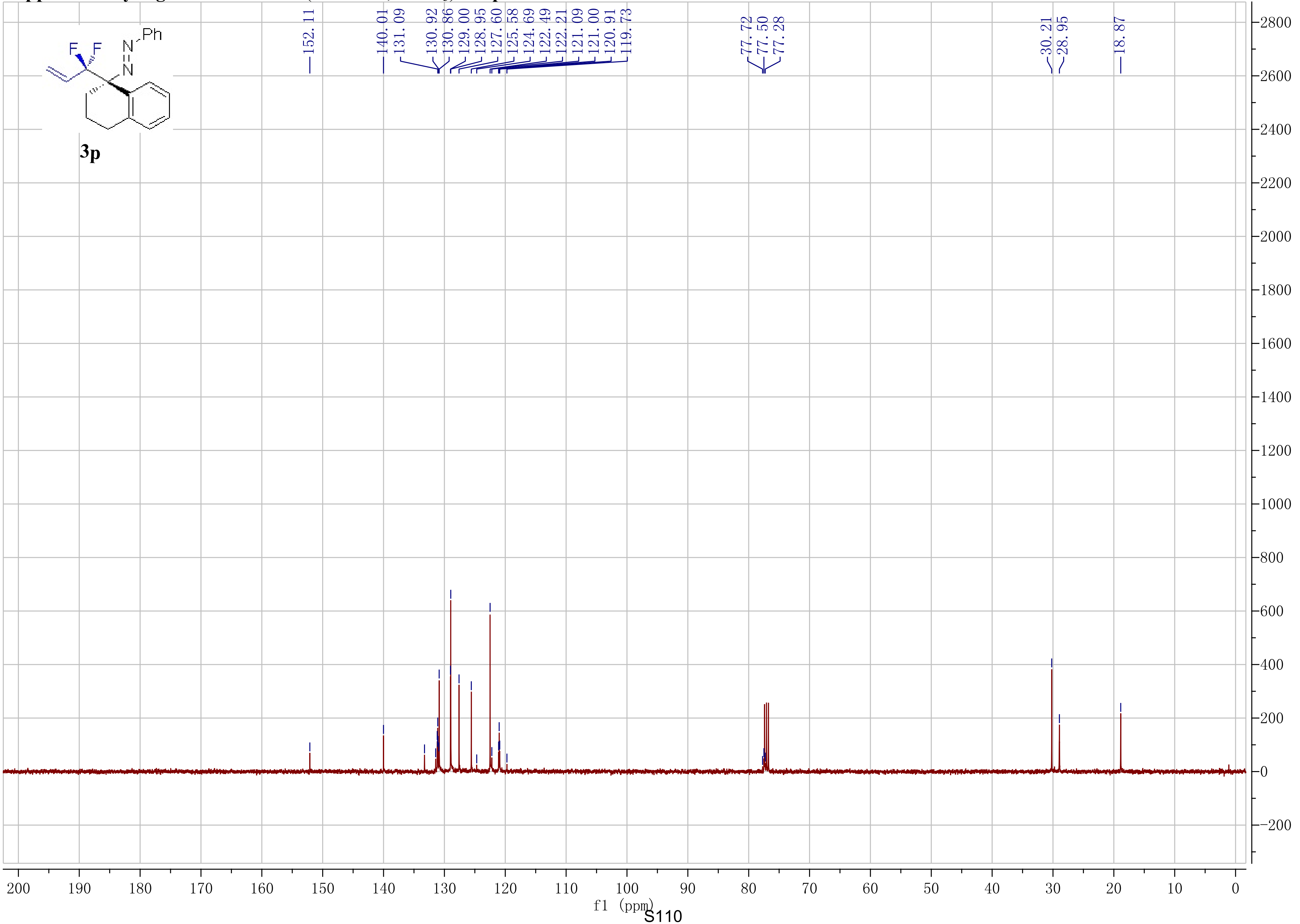

Supplementary Figure 81 ^{19}F NMR (376 MHz, $CDCl_3$) of **3p**

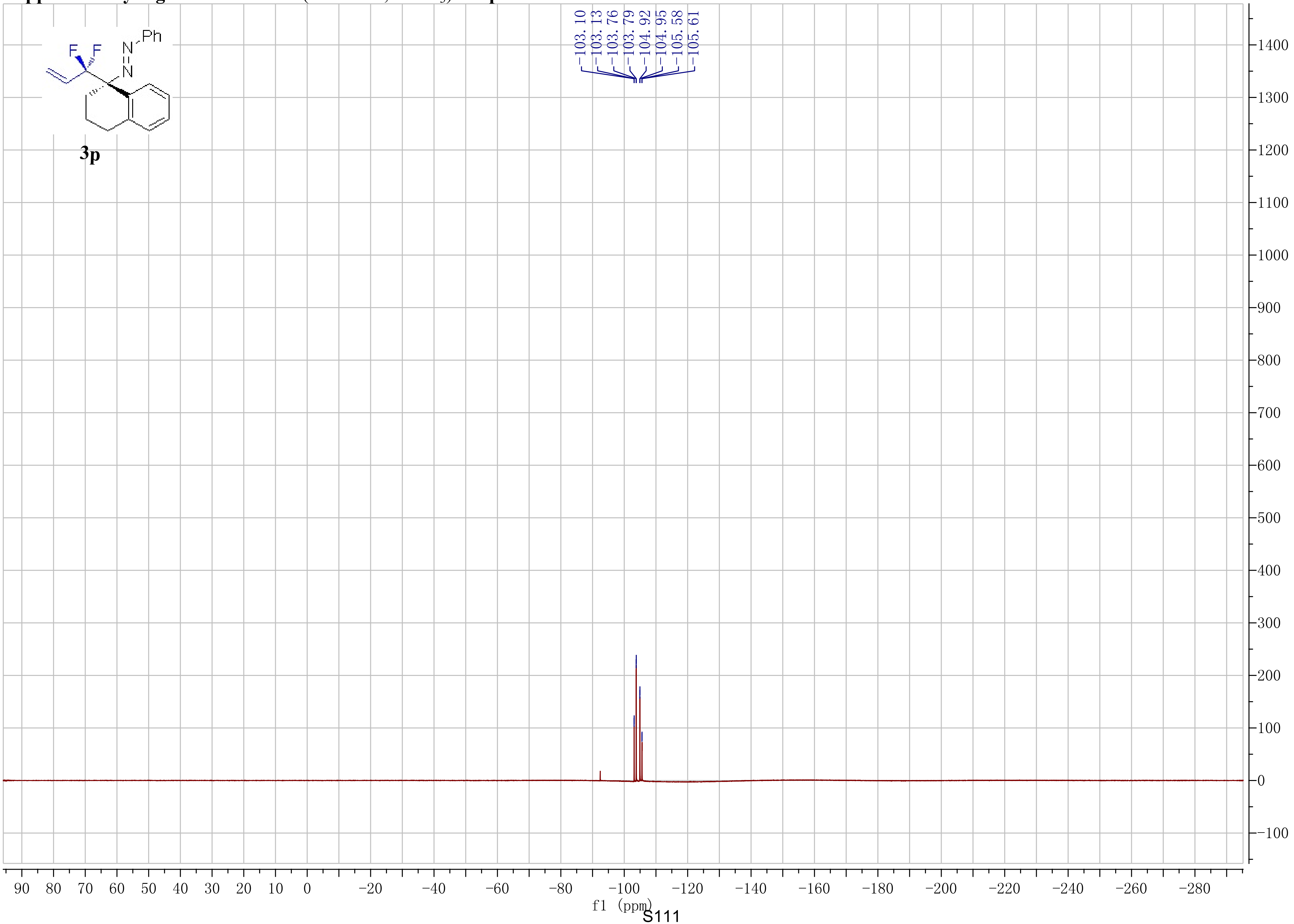

Supplementary Figure 82 HPLC spectra of racemic **3p**

Chromatogram and Results

Injection Details

Injection Name:	**HS-13-48-4+- PC3 A7W3 214 0.7**	*Run Time (min):*	**18.36**
Vial Number:	**RC2**	*Injection Volume:*	**2.00**
Injection Type:	**Unknown**	*Channel:*	**UV_VIS_1**
Calibration Level:		*Wavelength:*	**214.0**
Instrument Method:	**20160223-DAD3**	*Bandwidth:*	**4**
Processing Method:	**20160223**	*Dilution Factor:*	**1.0000**
Injection Date/Time:	**27/05/20 09:08**	*Sample Weight:*	**1.0000**

Chromatogram

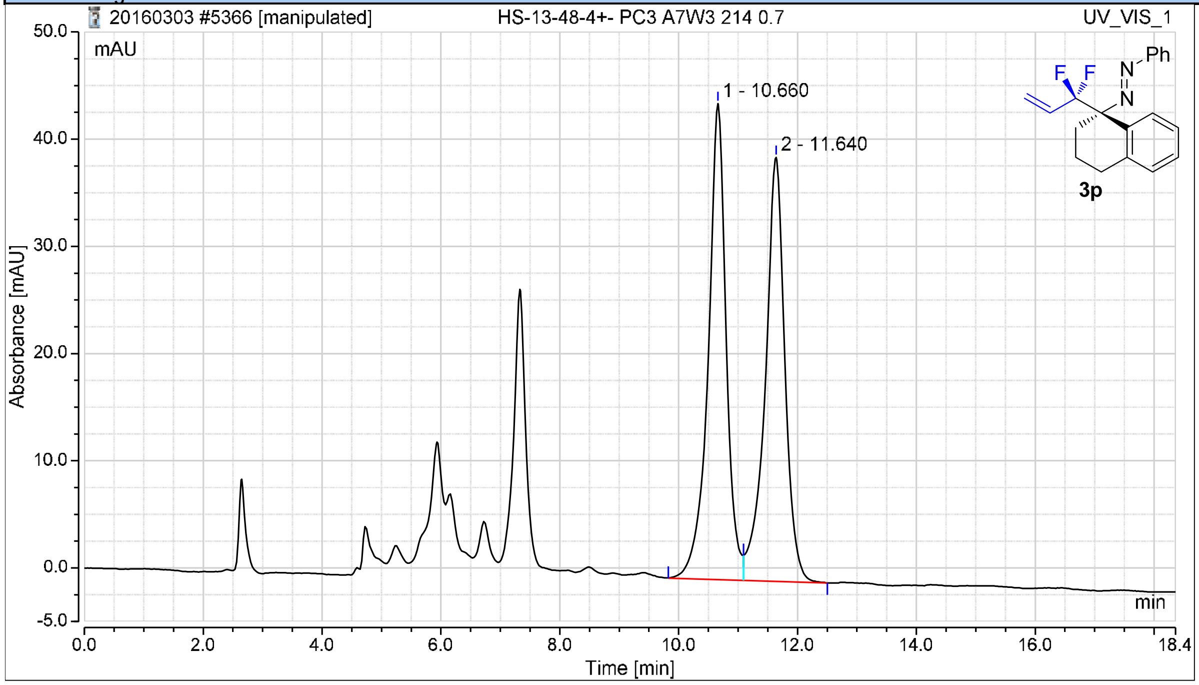

Integration Results

No.	Retention Time min	Area mAU*min	Height mAU	Relative Area %
1	10.660	15.4711	44.3956	50.481
2	11.640	15.1762	39.5596	49.519
**Total:**		**30.647**	**1401.998**	**100.000**

Supplementary Figure 83 HPLC spectra of (*S*)-**3p**

Chromatogram and Results

Injection Details

Injection Name:	**HS-13-56-11 PC3 A7W3 214 0.7**	*Run Time (min):*	**28.30**
Vial Number:	**RD2**	*Injection Volume:*	**2.00**
Injection Type:	**Unknown**	*Channel:*	**UV_VIS_1**
Calibration Level:		*Wavelength:*	**214.0**
Instrument Method:	**20160223-DAD3**	*Bandwidth:*	**4**
Processing Method:	**20160223**	*Dilution Factor:*	**1.0000**
Injection Date/Time:	**27/05/20 09:29**	*Sample Weight:*	**1.0000**

Chromatogram

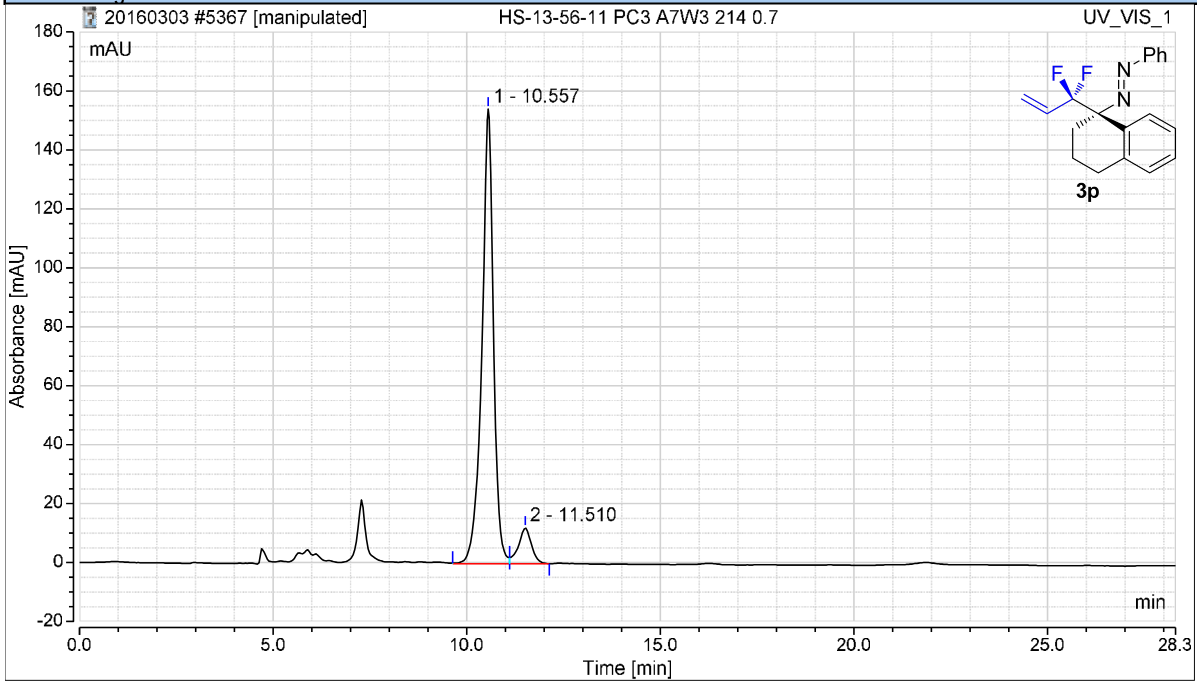

Integration Results

No.	Retention Time min	Area mAU*min	Height mAU	Relative Area %
1	10.557	53.2434	154.2687	91.781
2	11.510	4.7678	12.1497	8.219
**Total:**		**58.011**	**1401.998**	**100.000**

Supplementary Figure 84 ¹H NMR (400 MHz, $CDCl_3$) of **3q**

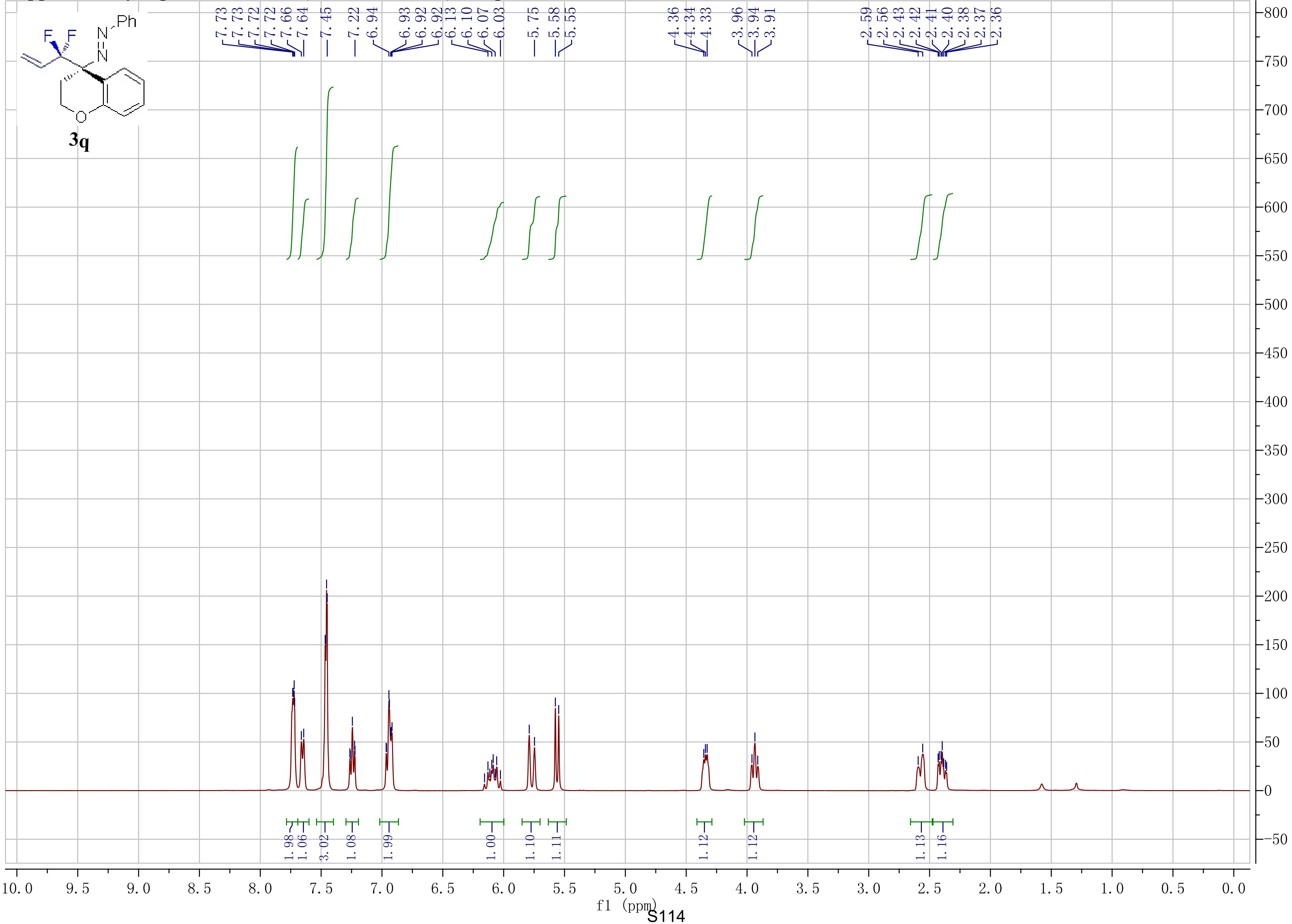

Supplementary Figure 85 ^{13}C NMR (101 MHz, $CDCl_3$) of **3q**

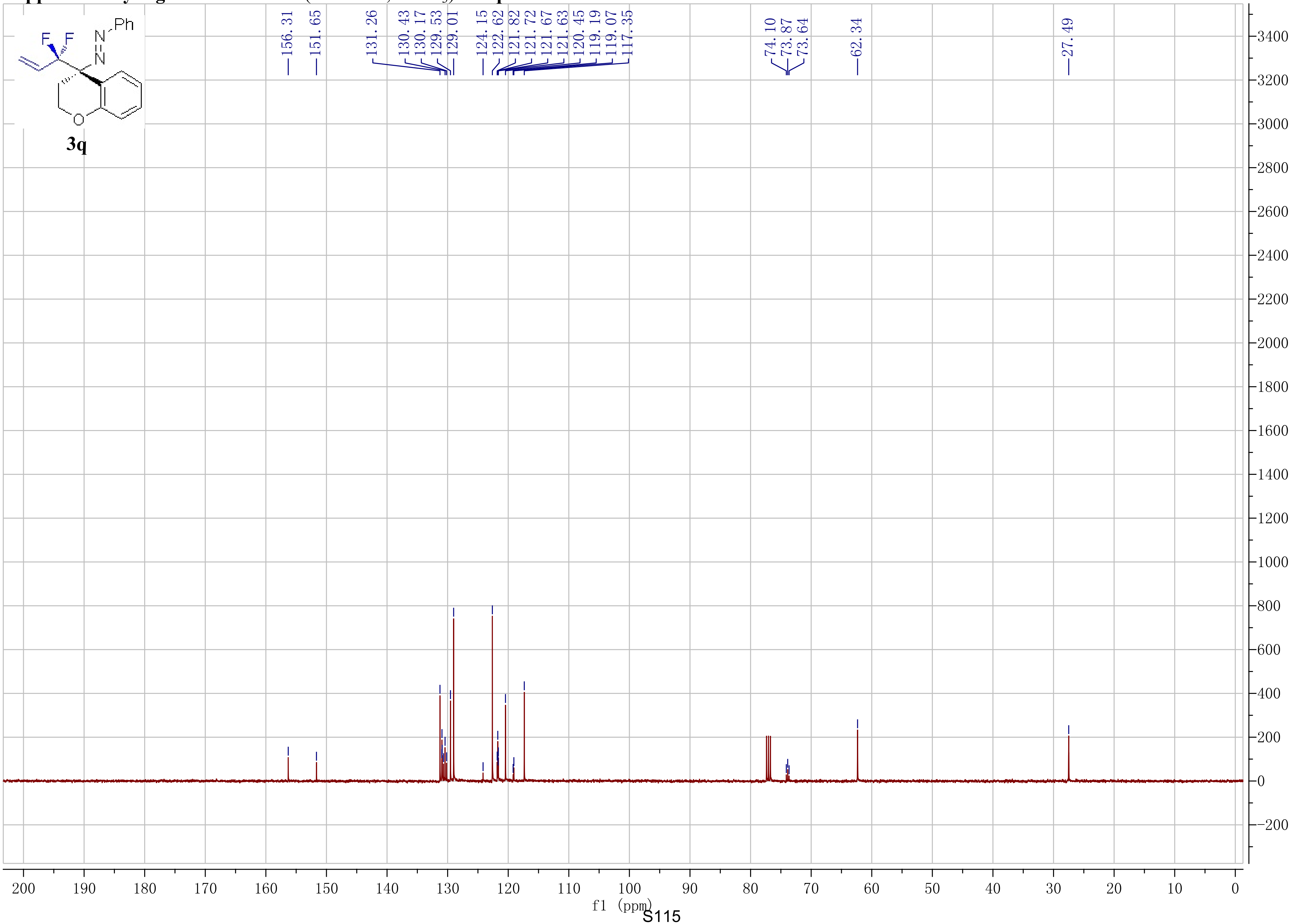

Supplementary Figure 86 ^{19}F NMR (376 MHz, $CDCl_3$) of **3q**

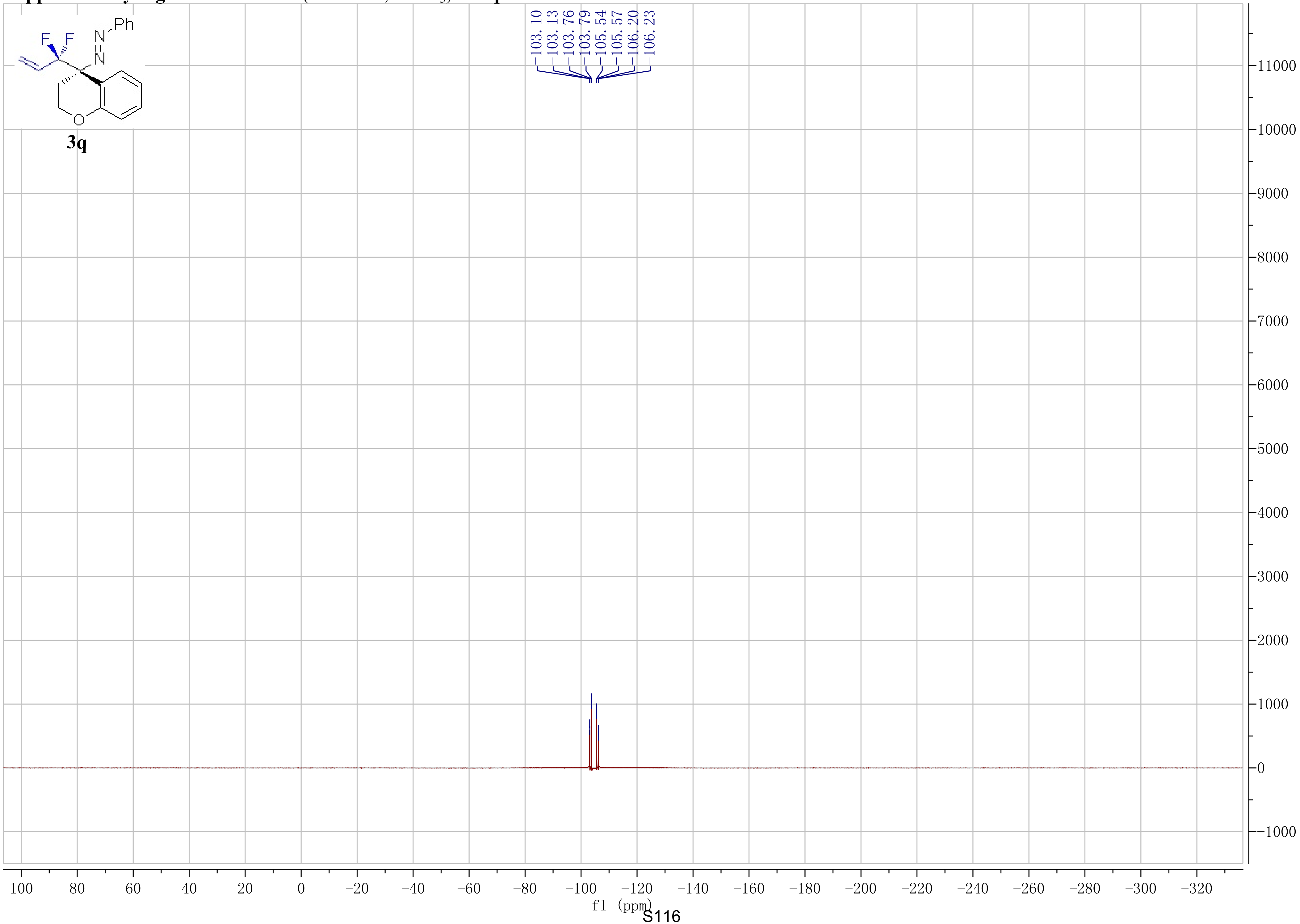

Supplementary Figure 87 HPLC spectra of racemic **3q**

HS-13-48-10+- OJH 955 214 0.7

Sample Name:	**HS-13-48-10+- OJH 955 214 0.7**	*Injection Volume:*	**2.0**
Vial Number:	**GB6**	*Channel:*	**UV_VIS_1**
Sample Type:	**unknown**	*Wavelength:*	**214**
Control Program:	**201701-4**	*Bandwidth:*	**n.a.**
Quantif. Method:	**201701**	*Dilution Factor:*	**1.0000**
Recording Time:	**2020/5/28 14:49**	*Sample Weight:*	**1.0000**
Run Time (min):	**21.40**	*Sample Amount:*	**1.0000**

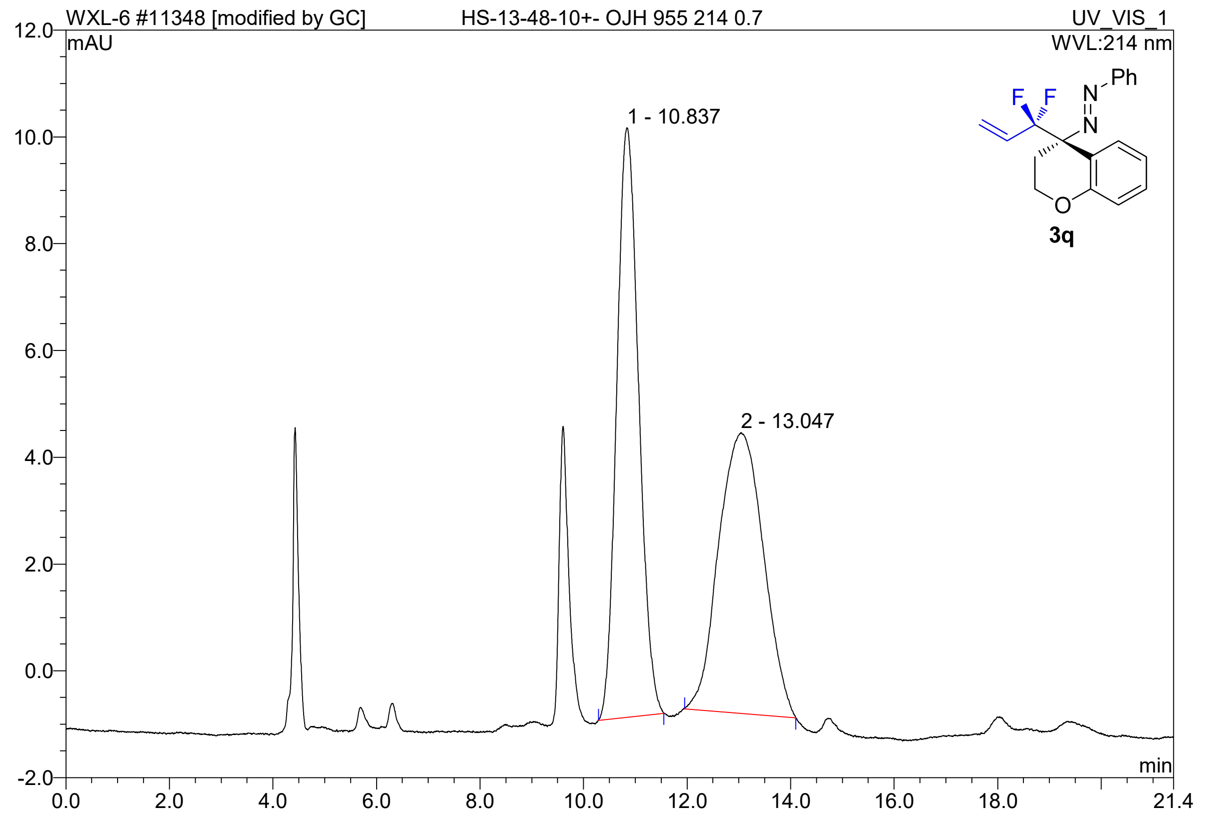

No.	Ret.Time min	Peak Name	Height mAU	Area mAU*min	Rel.Area %	Amount	Type
1	10.84	n.a.	11.042	5.423	50.73	n.a.	BMB
2	13.05	n.a.	5.265	5.267	49.27	n.a.	BMB
**Total:**			16.308	10.691	100.00	0.000	

Chromeleon (c) Dionex 1996-2006
Version 6.80 SR12 Build 3578 (207169)

Supplementary Figure 88 HPLC spectra of (*S*)-**3q**

HS-13-69-8 OJH 955 214 0.7

Sample Name:	**HS-13-69-8 OJH 955 214 0.7**	*Injection Volume:*	**2.0**
Vial Number:	**GB7**	*Channel:*	**UV_VIS_1**
Sample Type:	**unknown**	*Wavelength:*	**214**
Control Program:	**201701-4**	*Bandwidth:*	**n.a.**
Quantif. Method:	**201701**	*Dilution Factor:*	**1.0000**
Recording Time:	**2020/5/28 15:15**	*Sample Weight:*	**1.0000**
Run Time (min):	**25.00**	*Sample Amount:*	**1.0000**

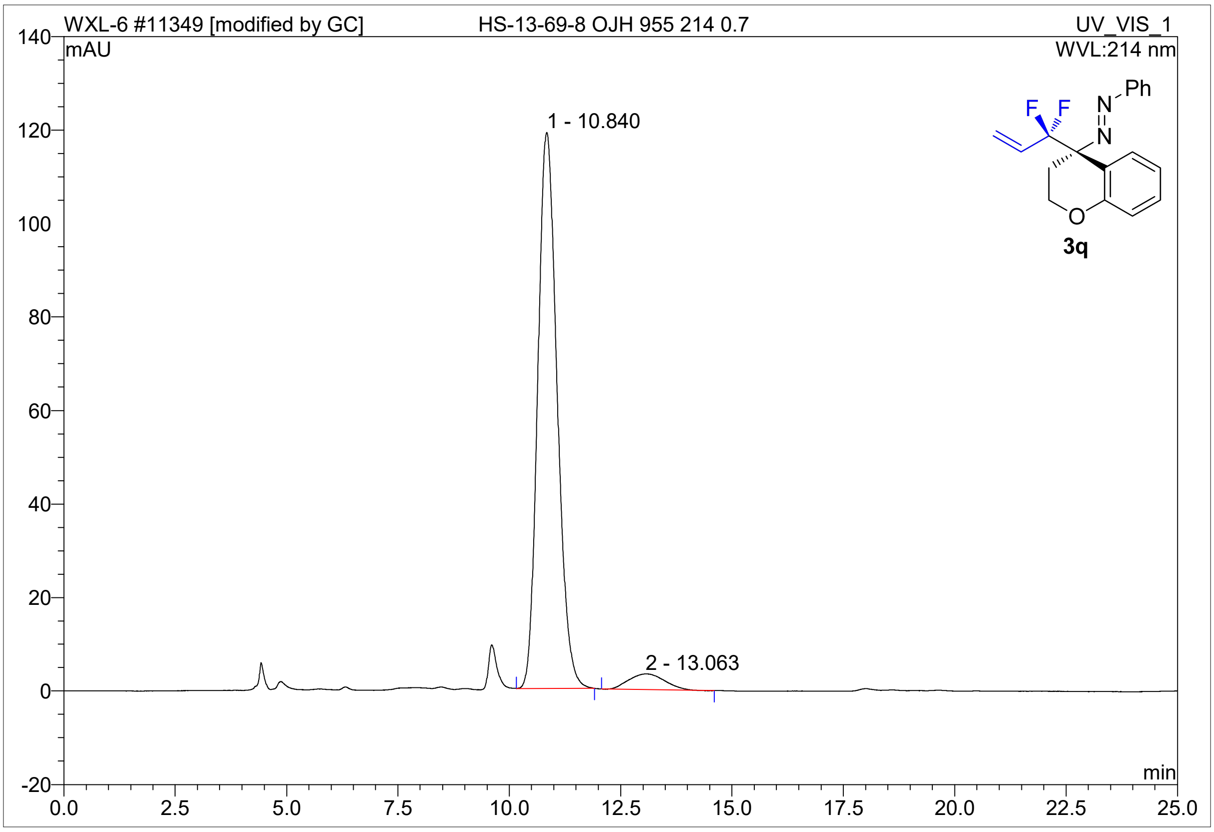

No.	Ret.Time min	Peak Name	Height mAU	Area mAU*min	Rel.Area %	Amount	Type
1	10.84	n.a.	118.975	59.860	94.99	n.a.	BMB*
2	13.06	n.a.	3.369	3.160	5.01	n.a.	BMB*
**Total:**			122.344	63.020	100.00	0.000	

Chromeleon (c) Dionex 1996-2006
Version 6.80 SR12 Build 3578 (207169)

Supplementary Figure 89 ^{1}H NMR (400 MHz, $CDCl_3$) of **3r**

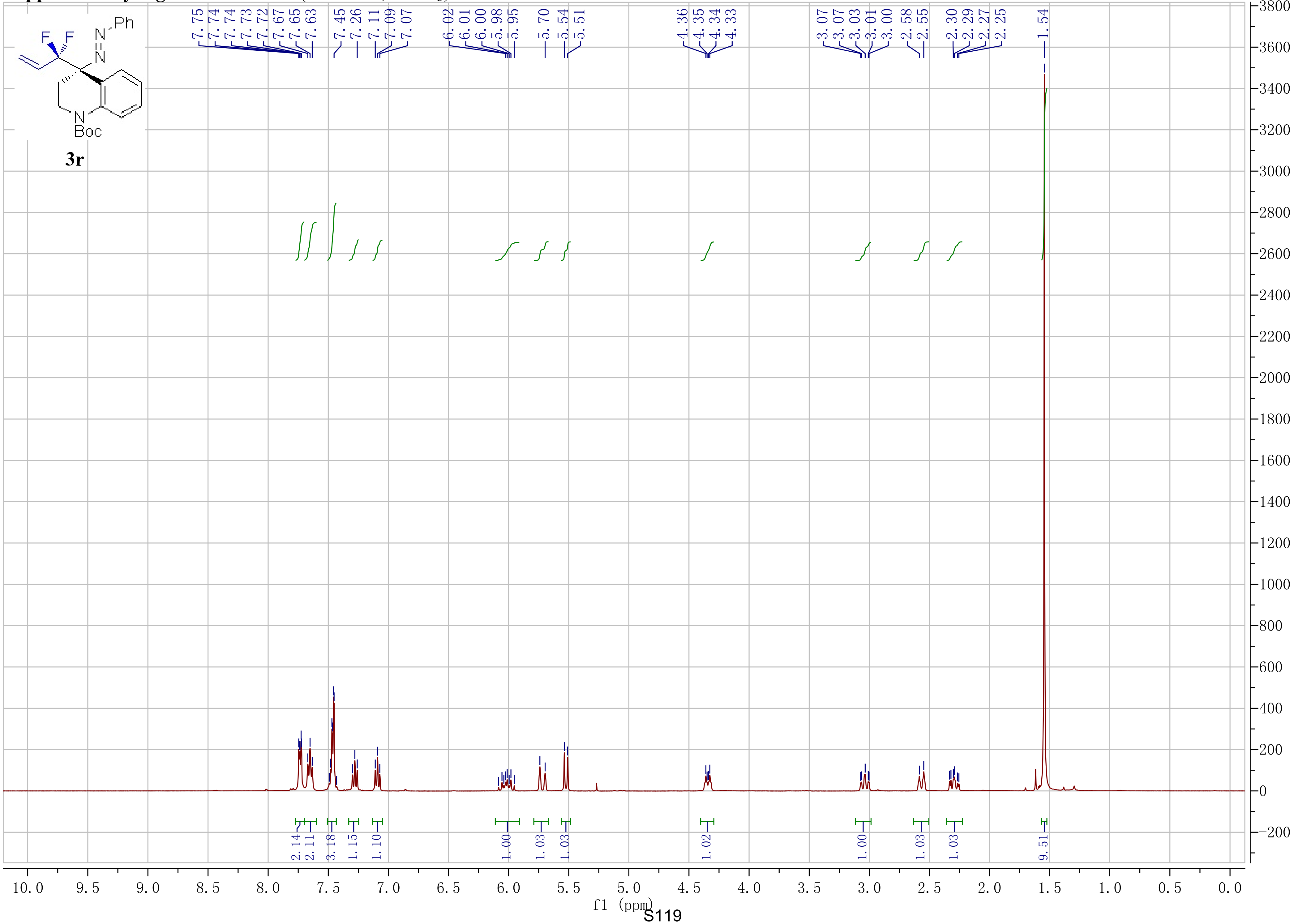

Supplementary Figure 90 ^{13}C NMR (101 MHz, $CDCl_3$) of **3r**

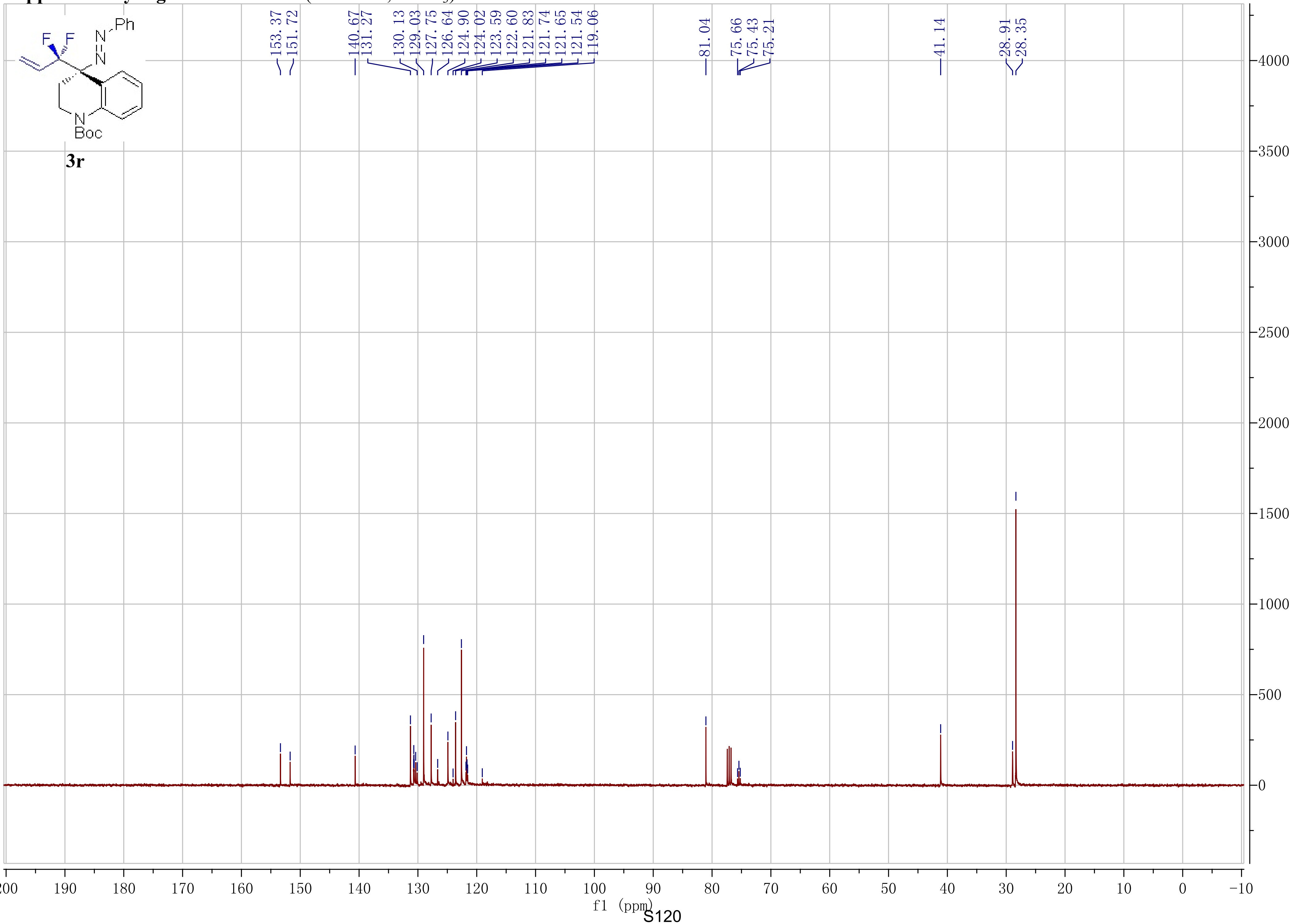

Supplementary Figure 91 ^{19}F NMR (376 MHz, $CDCl_3$) of **3r**

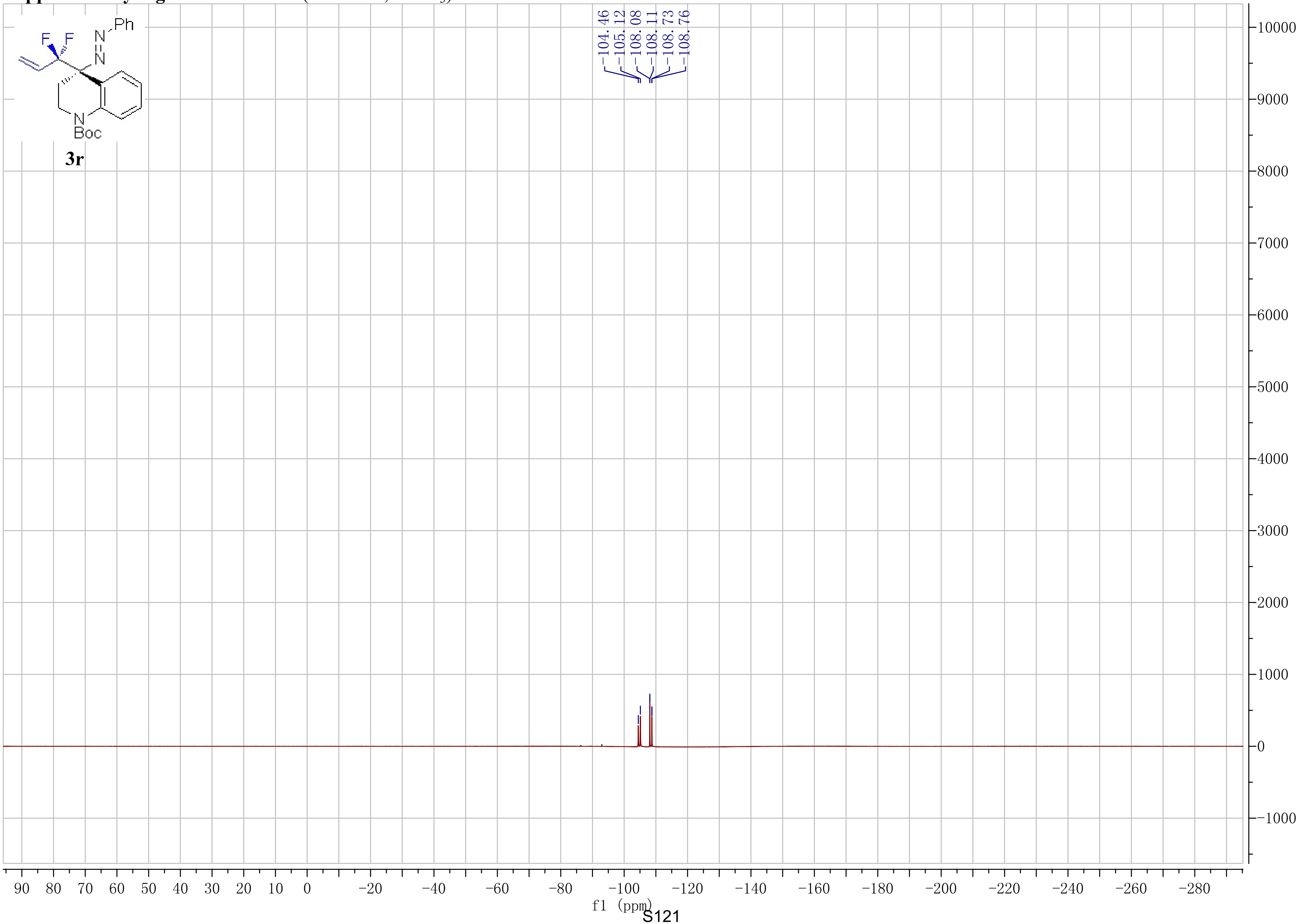

Supplementary Figure 92 HPLC spectra of racemic **3r**

Operator:GC Timebase:U3000 Sequence:WXL-6

2020/6/10 3:34 PM

HS-13-73-3+- IE3 982 214 0.7

Sample Name:	**HS-13-73-3+- IE3 982 214 0.7**		**3.0**
Vial Number:	**GA3**	*Channel:*	**UV_VIS_1**
Sample Type:	**unknown**	*Wavelength:*	**214**
Control Program:	**201701-5**	*Bandwidth:*	**n.a.**
Quantif. Method:	**201701**	*Dilution Factor:*	**1.0000**
Recording Time:	**2020/6/2 9:26**	*Sample Weight:*	**1.0000**
Run Time (min):	**11.07**	*Sample Amount:*	**1.0000**

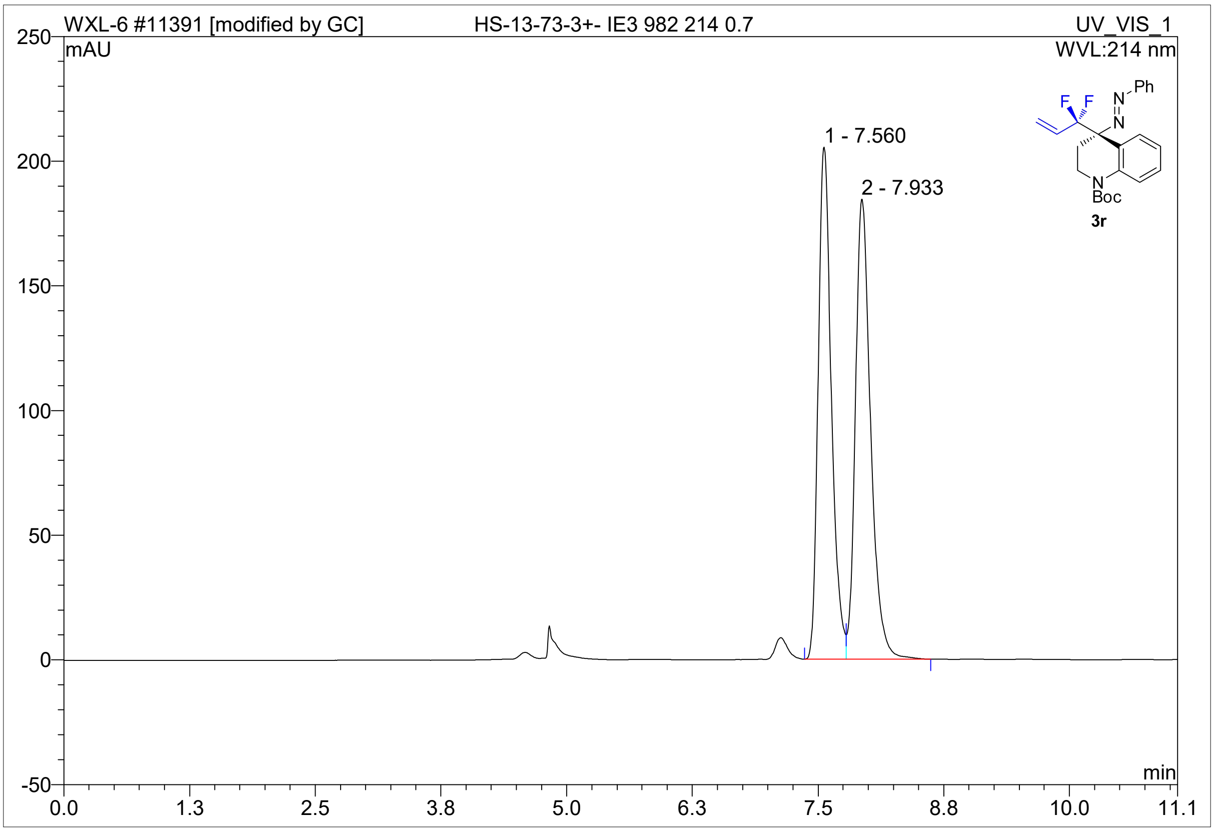

No.	Ret.Time min	Peak Name	Height mAU	Area mAU*min	Rel.Area %	Amount	Type
1	7.56	n.a.	205.512	31.634	49.42	n.a.	BM *
2	7.93	n.a.	184.592	32.379	50.58	n.a.	MB*
**Total:**			390.104	64.013	100.00	0.000	

DEFAULT/Integration

Chromeleon (c) Dionex 1996-2006
Version 6.80 SR12 Build 3578 (207169)

Supplementary Figure 93 HPLC spectra of (*S*)-**3r**

HS-13-75-2 IE3 982 214 0.7

Sample Name:	**HS-13-75-2 IE3 982 214 0.7**	*Injection Volume:*	**2.0**
Vial Number:	**GA4**	*Channel:*	**UV_VIS_1**
Sample Type:	**unknown**	*Wavelength:*	**214**
Control Program:	**201701-5**	*Bandwidth:*	**n.a.**
Quantif. Method:	**201701**	*Dilution Factor:*	**1.0000**
Recording Time:	**2020/6/2 9:39**	*Sample Weight:*	**1.0000**
Run Time (min):	**19.95**	*Sample Amount:*	**1.0000**

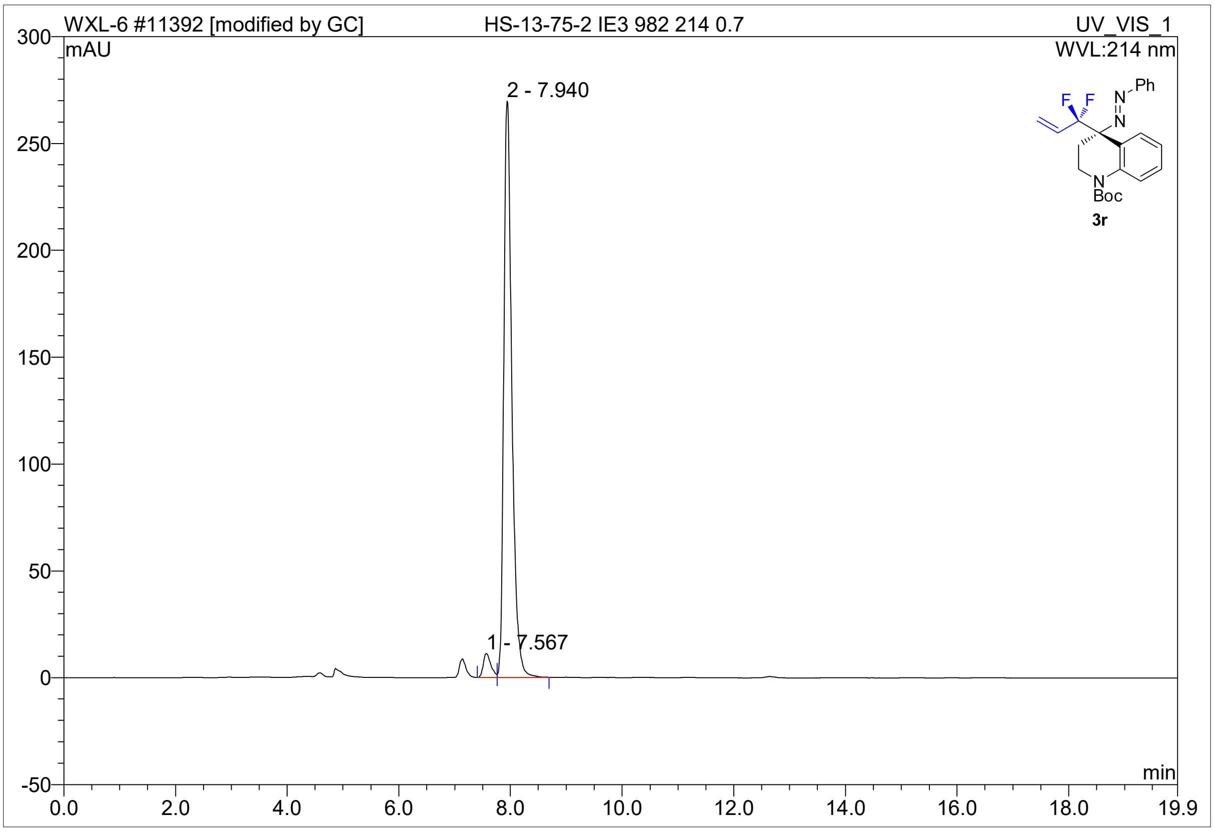

No.	Ret.Time min	Peak Name	Height mAU	Area mAU*min	Rel.Area %	Amount	Type
1	7.57	n.a.	11.176	1.768	3.77	n.a.	BM
2	7.94	n.a.	269.749	45.178	96.23	n.a.	MB
**Total:**			280.925	46.946	100.00	0.000	

Supplementary Figure 94 ^{1}H NMR (400 MHz, $CDCl_3$) of **3s**

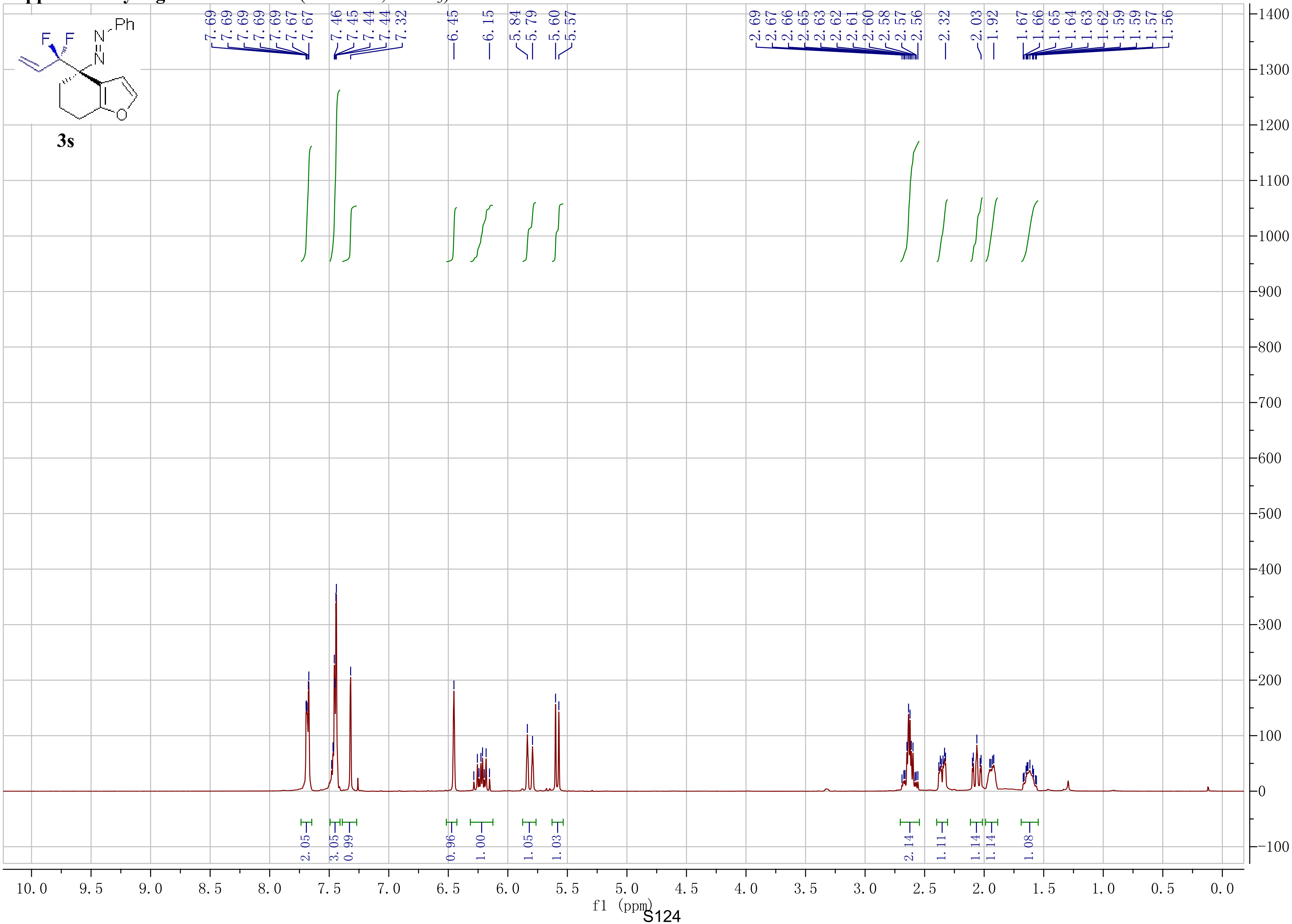

Supplementary Figure 95 ^{13}C NMR (101 MHz, $CDCl_3$) of **3s**

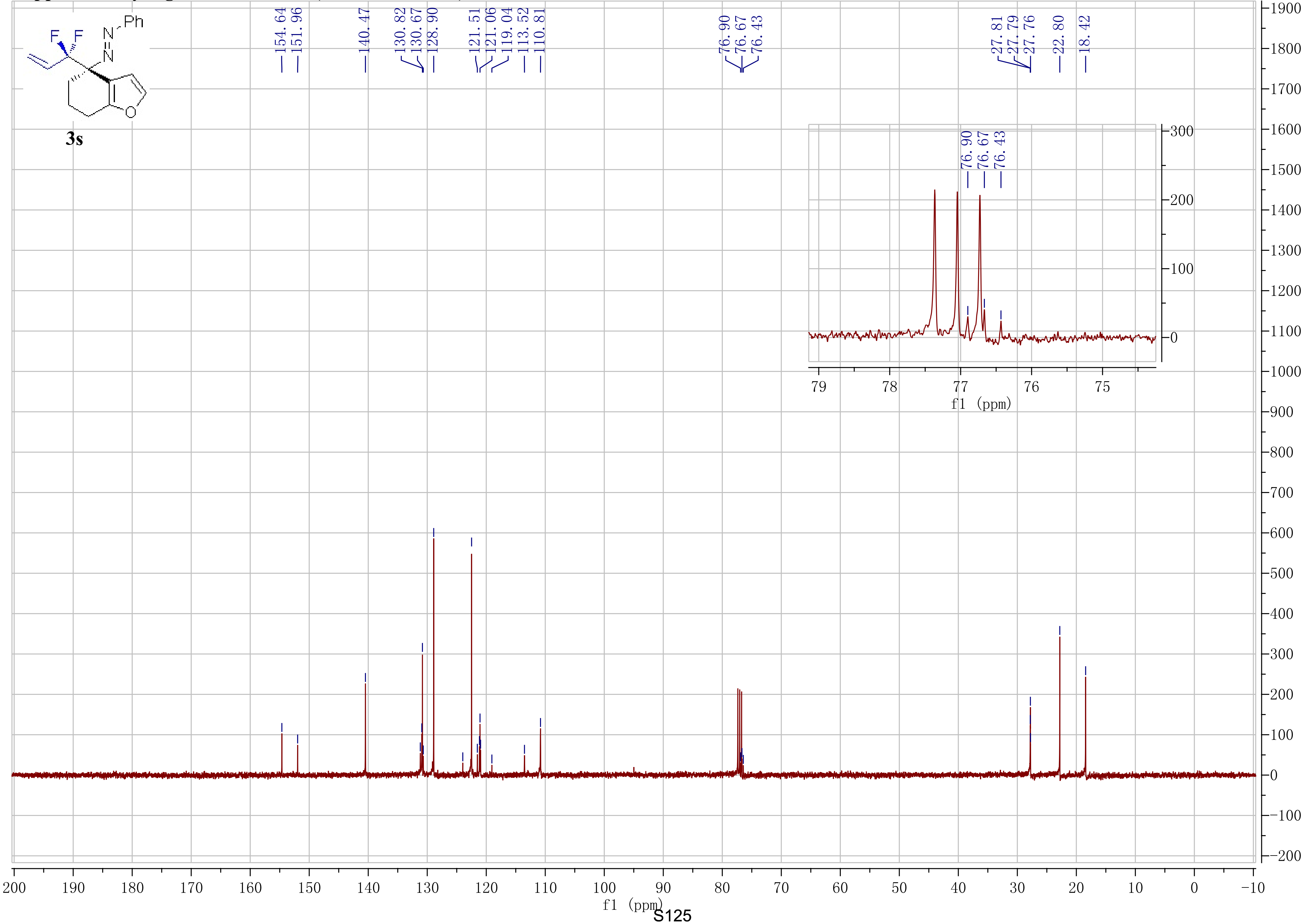

Supplementary Figure 96 ^{19}F NMR (376 MHz, $CDCl_3$) of **3s**

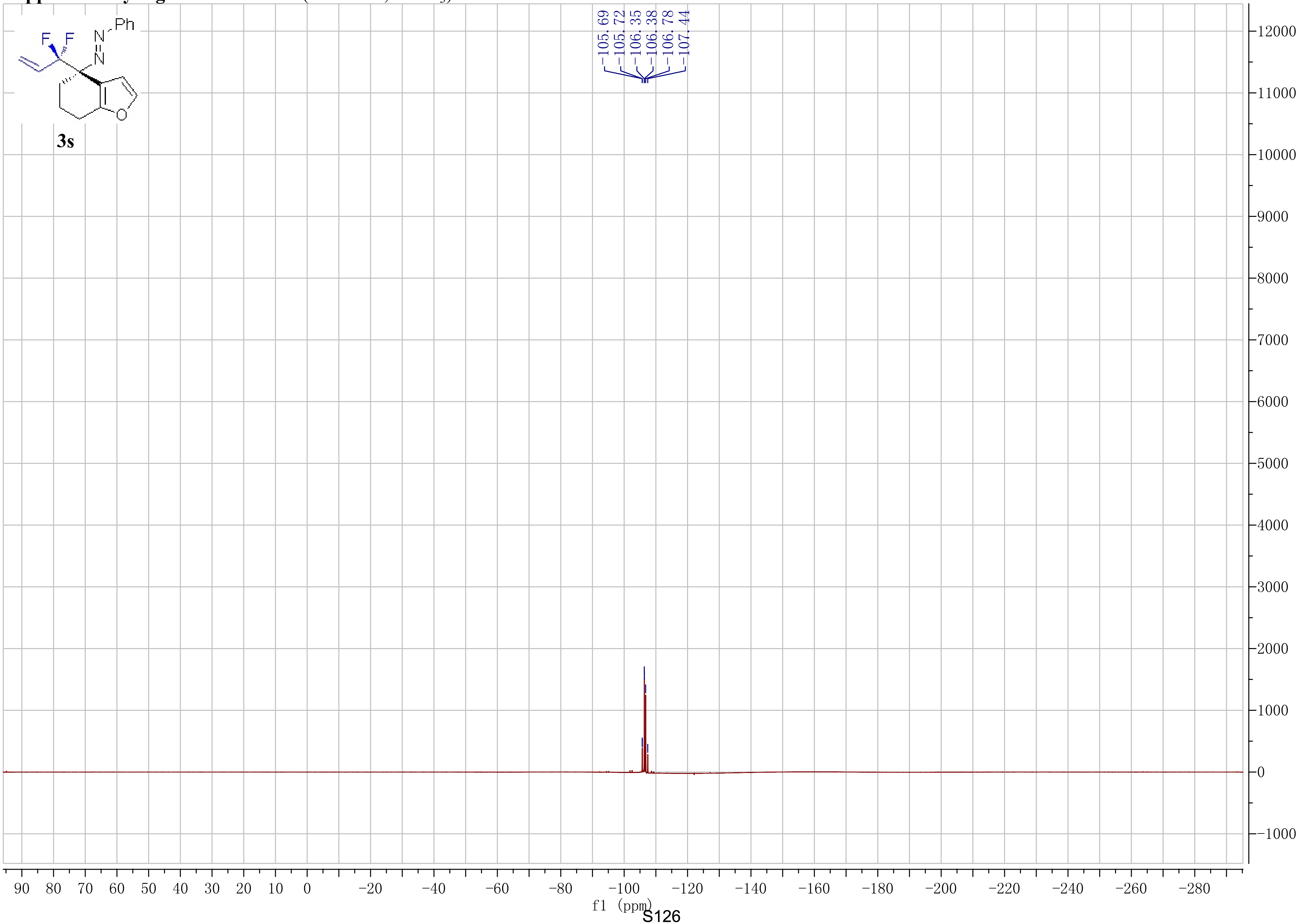

Supplementary Figure 97 HPLC spectra of racemic **3s**

HS-1373-2+- OJH 982 214 0.7

Sample Name:	**HS-1373-2+- OJH 982 214 0.7**	*Injection Volume:*	**2.0**
Vial Number:	**GB5**	*Channel:*	**UV_VIS_1**
Sample Type:	**unknown**	*Wavelength:*	**214**
Control Program:	**201701-5**	*Bandwidth:*	**n.a.**
Quantif. Method:	**201701**	*Dilution Factor:*	**1.0000**
Recording Time:	**2020/8/21 14:32**	*Sample Weight:*	**1.0000**
Run Time (min):	**19.55**	*Sample Amount:*	**1.0000**

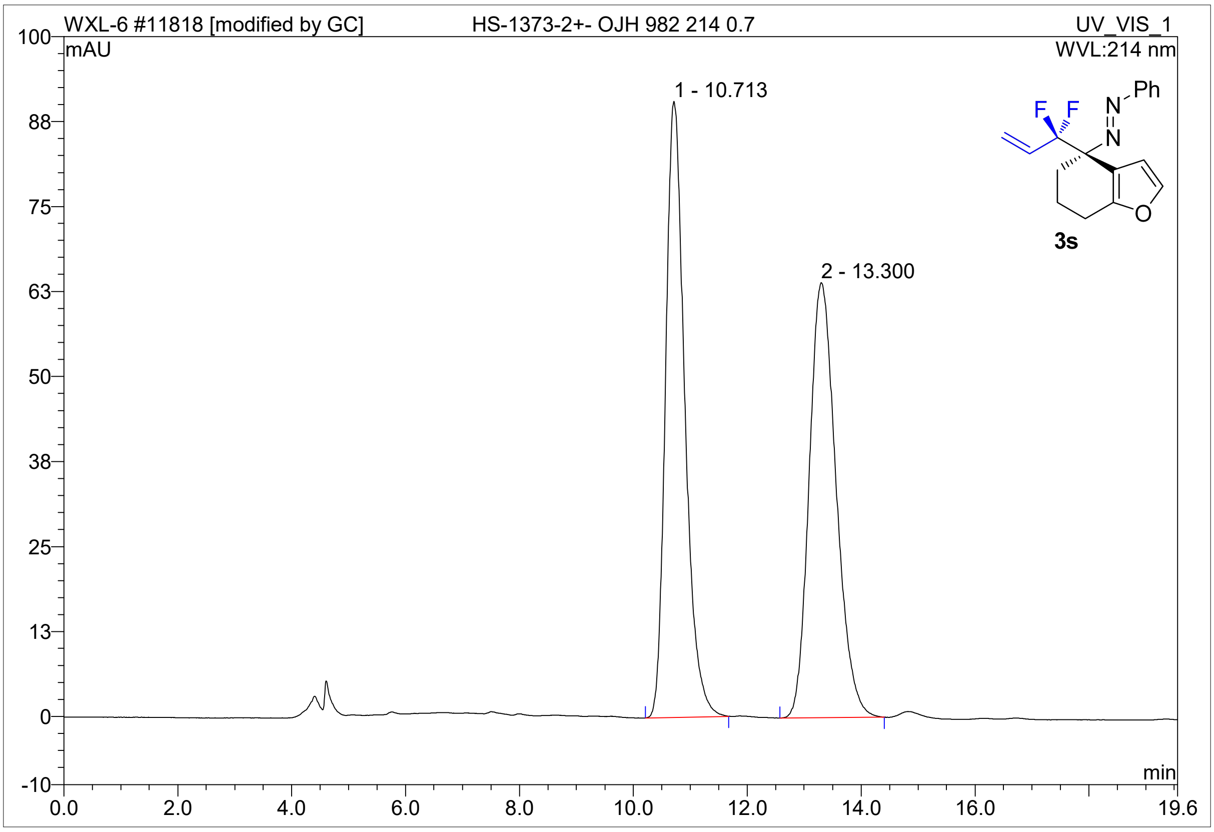

No.	Ret.Time min	Peak Name	Height mAU	Area mAU*min	Rel.Area %	Amount	Type
1	10.71	n.a.	90.595	35.442	50.25	n.a.	BMB
2	13.30	n.a.	63.965	35.085	49.75	n.a.	BMB
**Total:**			154.560	70.526	100.00	0.000	

Supplementary Figure 98 HPLC spectra of (*S*)-**3s**

HS-1375-1 OJH 982 214 0.7

Sample Name:	**HS-1375-1 OJH 982 214 0.7**	*Injection Volume:*	**2.0**
Vial Number:	**GC5**	*Channel:*	**UV_VIS_1**
Sample Type:	**unknown**	*Wavelength:*	**214**
Control Program:	**201701-5**	*Bandwidth:*	**n.a.**
Quantif. Method:	**201701**	*Dilution Factor:*	**1.0000**
Recording Time:	**2020/8/21 14:54**	*Sample Weight:*	**1.0000**
Run Time (min):	**22.64**	*Sample Amount:*	**1.0000**

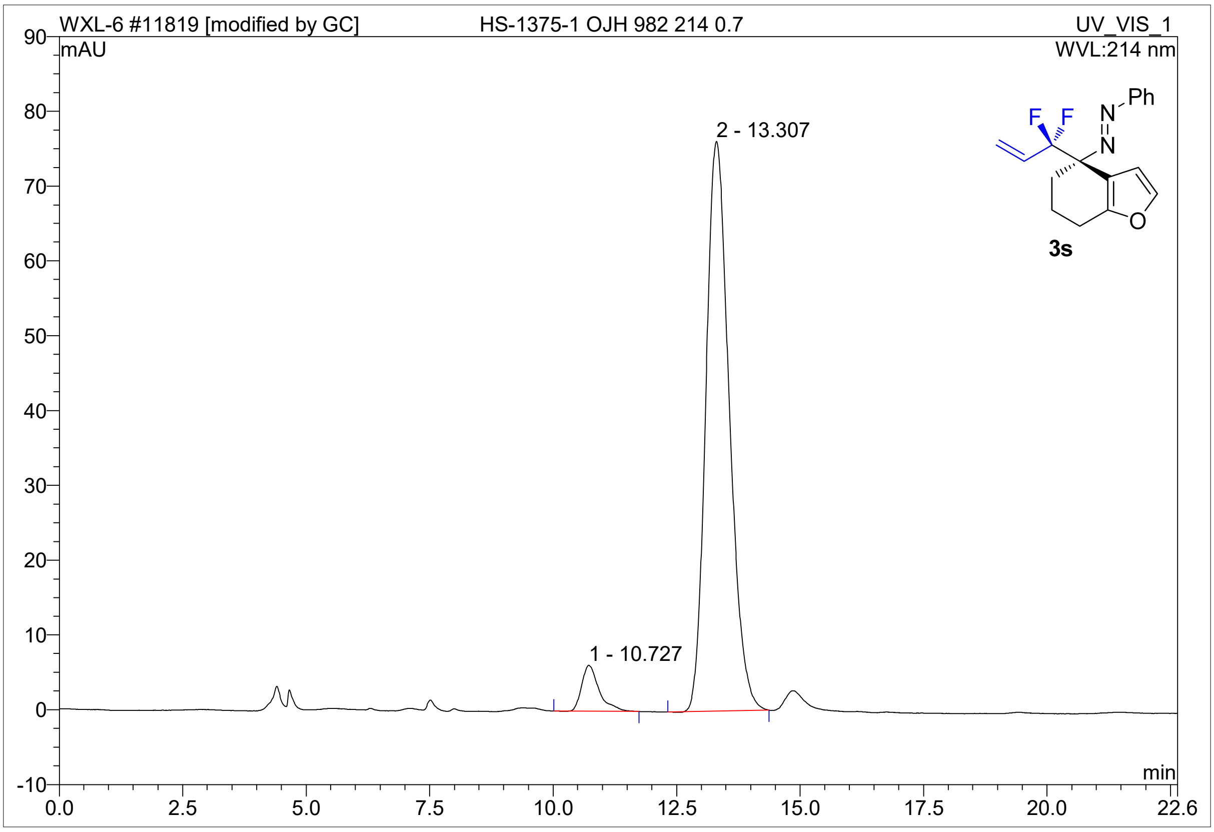

No.	Ret.Time min	Peak Name	Height mAU	Area mAU*min	Rel.Area %	Amount	Type
1	10.73	n.a.	6.135	2.498	5.64	n.a.	BMB*
2	13.31	n.a.	76.167	41.804	94.36	n.a.	BMB*
**Total:**			82.301	44.302	100.00	0.000	

Supplementary Figure 99 ^{1}H NMR (400 MHz, $CDCl_3$) of **3t**

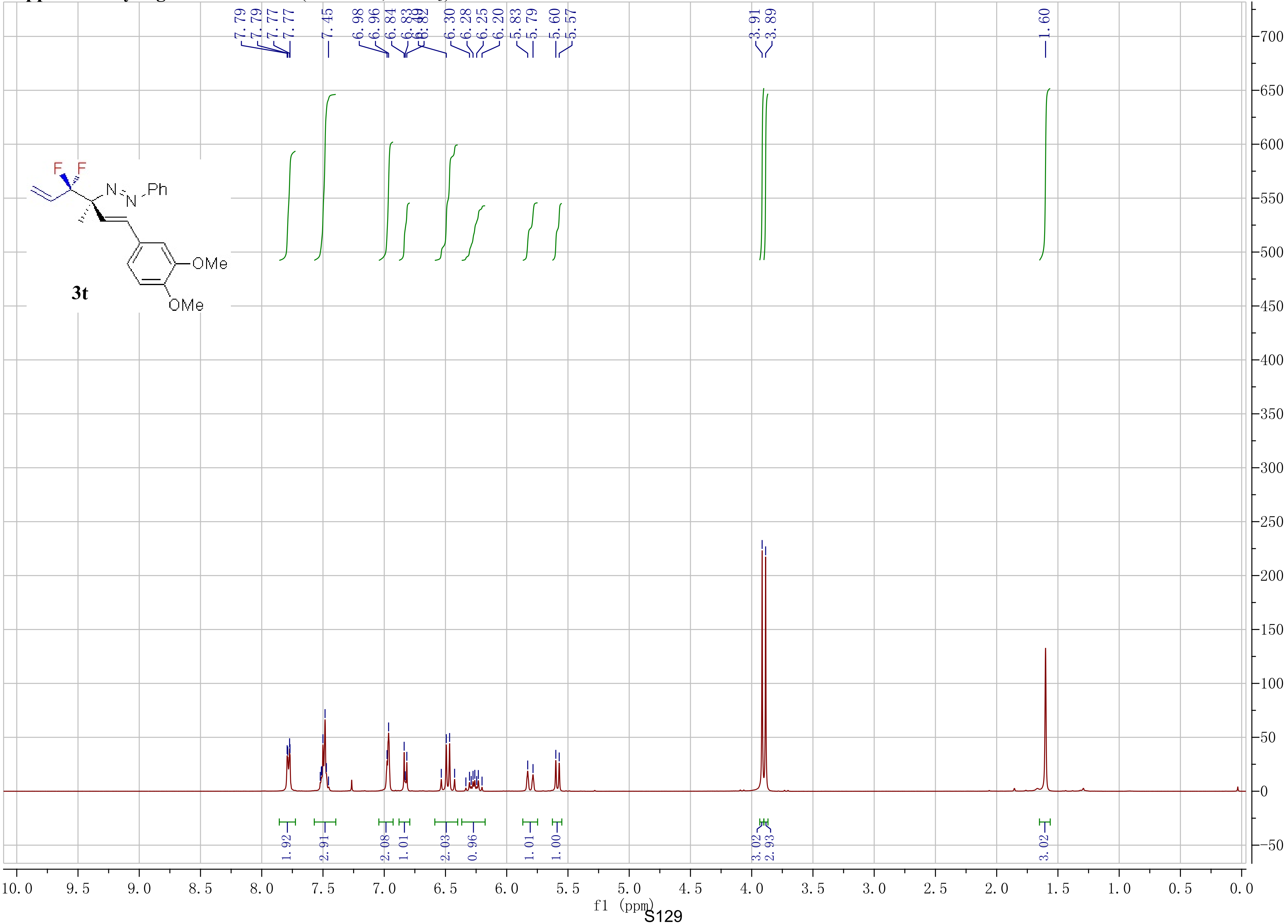

Supplementary Figure 100 ^{13}C NMR (101 MHz, $CDCl_3$) of **3t**

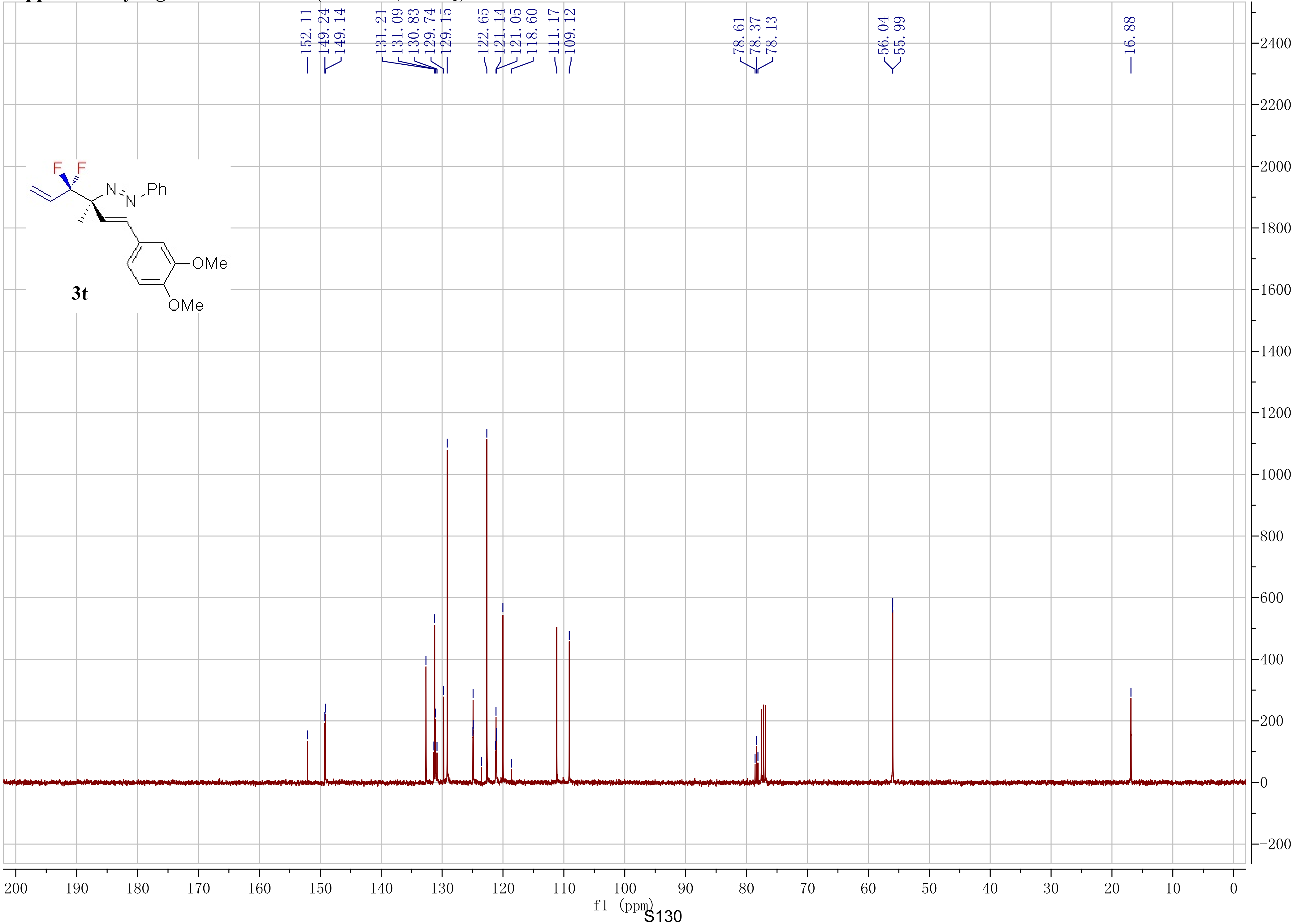

Supplementary Figure 101 ^{19}F NMR (376 MHz, $CDCl_3$) of **3t**

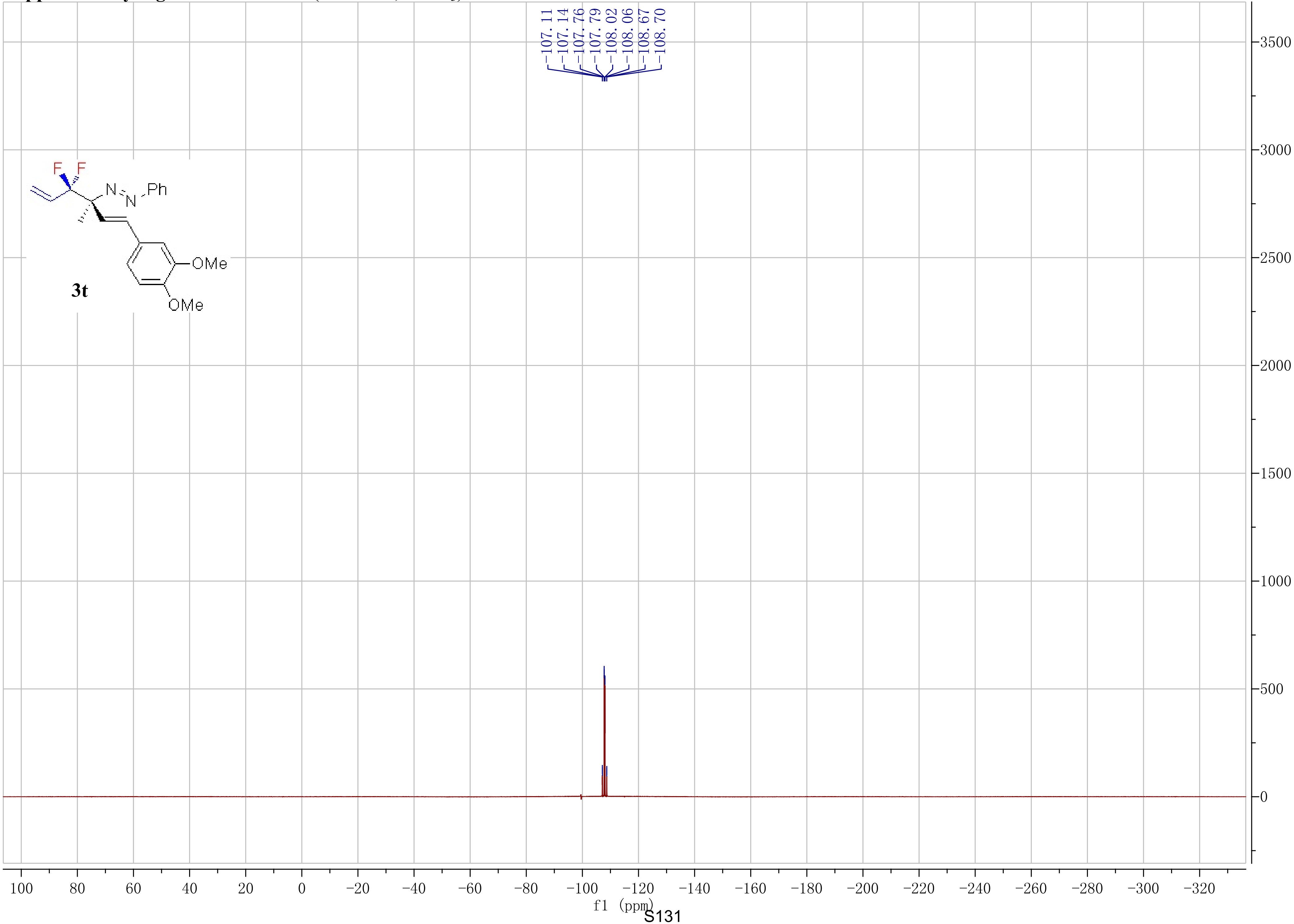

Supplementary Figure 102 HPLC spectra of racemic **3t**

9788 HS-14-6-6+- IA 955 214 0.7

Sample Name:	**HS-14-6-6+- IA 955 214 0.7**	*Injection Volume:*	**2.0**
Vial Number:	**RE5**	*Channel:*	**UV_VIS_2**
Sample Type:	**unknown**	*Wavelength:*	**214.0**
Control Program:	**test-dad4**	*Bandwidth:*	**4**
Quantif. Method:	**20170608**	*Dilution Factor:*	**1.0000**
Recording Time:	**2020-11-23 9:14**	*Sample Weight:*	**1.0000**
Run Time (min):	**29.20**	*Sample Amount:*	**1.0000**

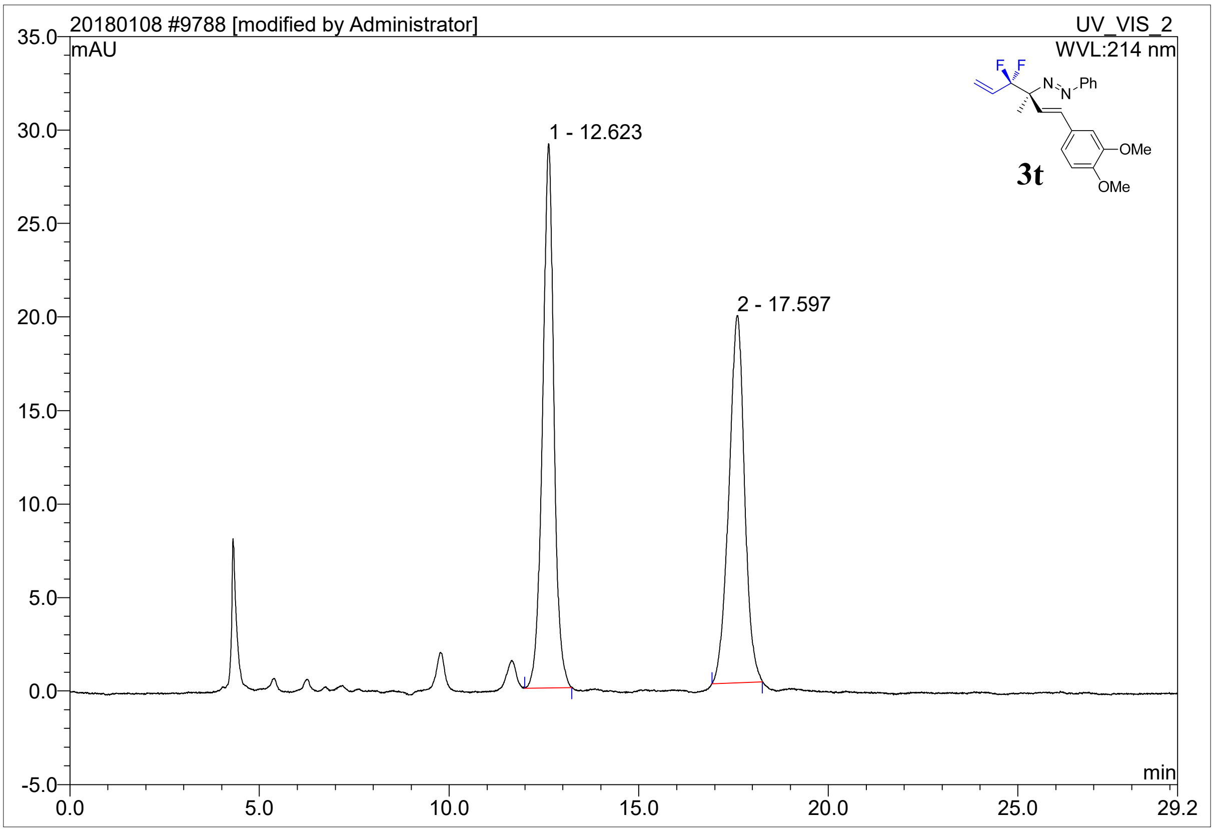

No.	Ret.Time min	Peak Name	Height mAU	Area mAU*min	Rel.Area %	Amount	Type
1	12.62	n.a.	29.120	10.079	51.64	n.a.	BMB*
2	17.60	n.a.	19.639	9.438	48.36	n.a.	BMB
**Total:**			48.760	19.517	100.00	0.000	

Chromeleon (c) Dionex 1996-2006
Version 6.80 SR14 Build 4527 (238909)

Supplementary Figure 103 HPLC spectra of (*S*)-**3t**

9789 HS-14-7-8 IA 955 214 0.7

Sample Name:	**HS-14-7-8 IA 955 214 0.7**	*Injection Volume:*	**2.0**
Vial Number:	**RE6**	*Channel:*	**UV_VIS_2**
Sample Type:	**unknown**	*Wavelength:*	**214.0**
Control Program:	**test-dad4**	*Bandwidth:*	**4**
Quantif. Method:	**20170608**	*Dilution Factor:*	**1.0000**
Recording Time:	**2020-11-23 9:45**	*Sample Weight:*	**1.0000**
Run Time (min):	**33.76**	*Sample Amount:*	**1.0000**

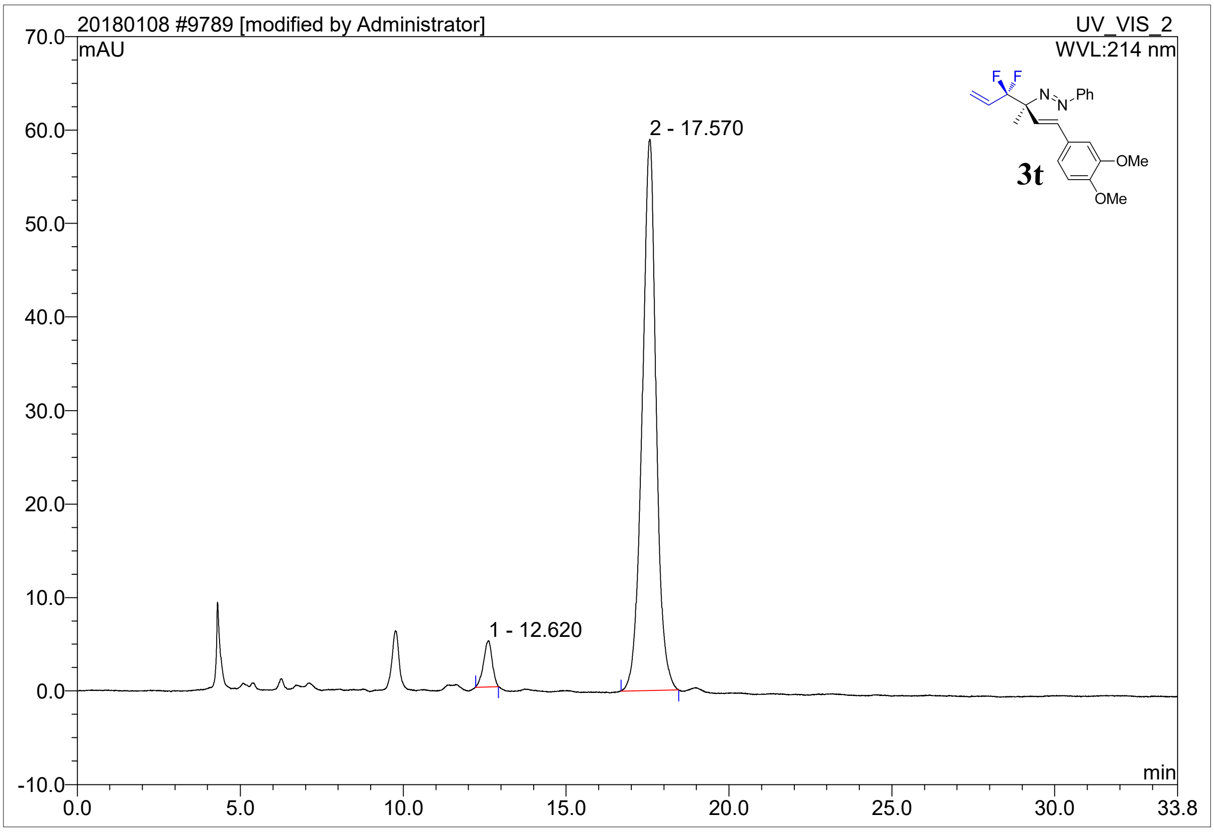

No.	Ret.Time min	Peak Name	Height mAU	Area mAU*min	Rel.Area %	Amount	Type
1	12.62	n.a.	4.937	1.517	4.92	n.a.	BMB
2	17.57	n.a.	58.985	29.334	95.08	n.a.	BMB
**Total:**			63.922	30.850	100.00	0.000	

Supplementary Figure 104 ^{1}H NMR (400 MHz, $CDCl_3$) of **3u**

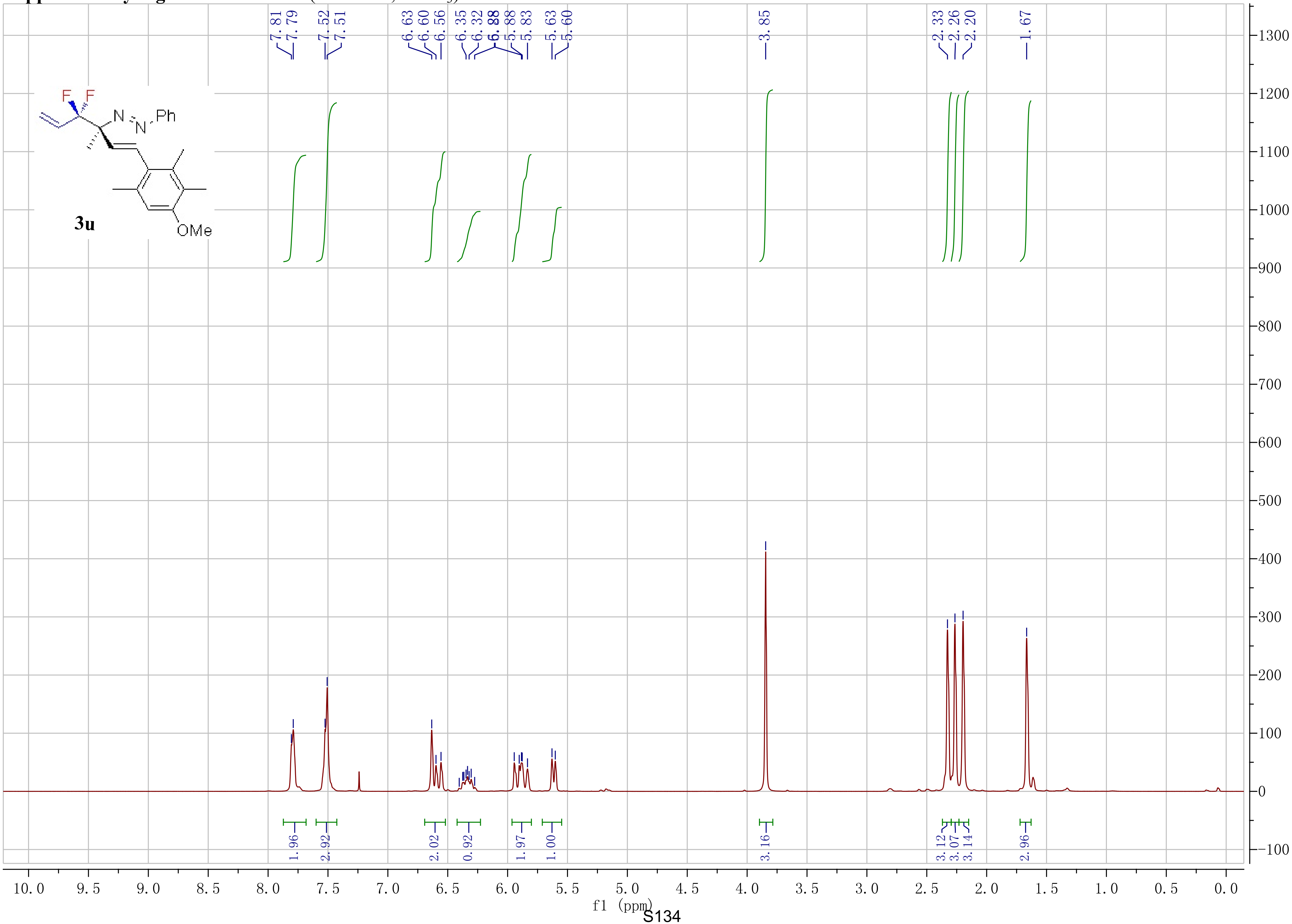

Supplementary Figure 105 ^{13}C NMR (101 MHz, $CDCl_3$) of **3u**

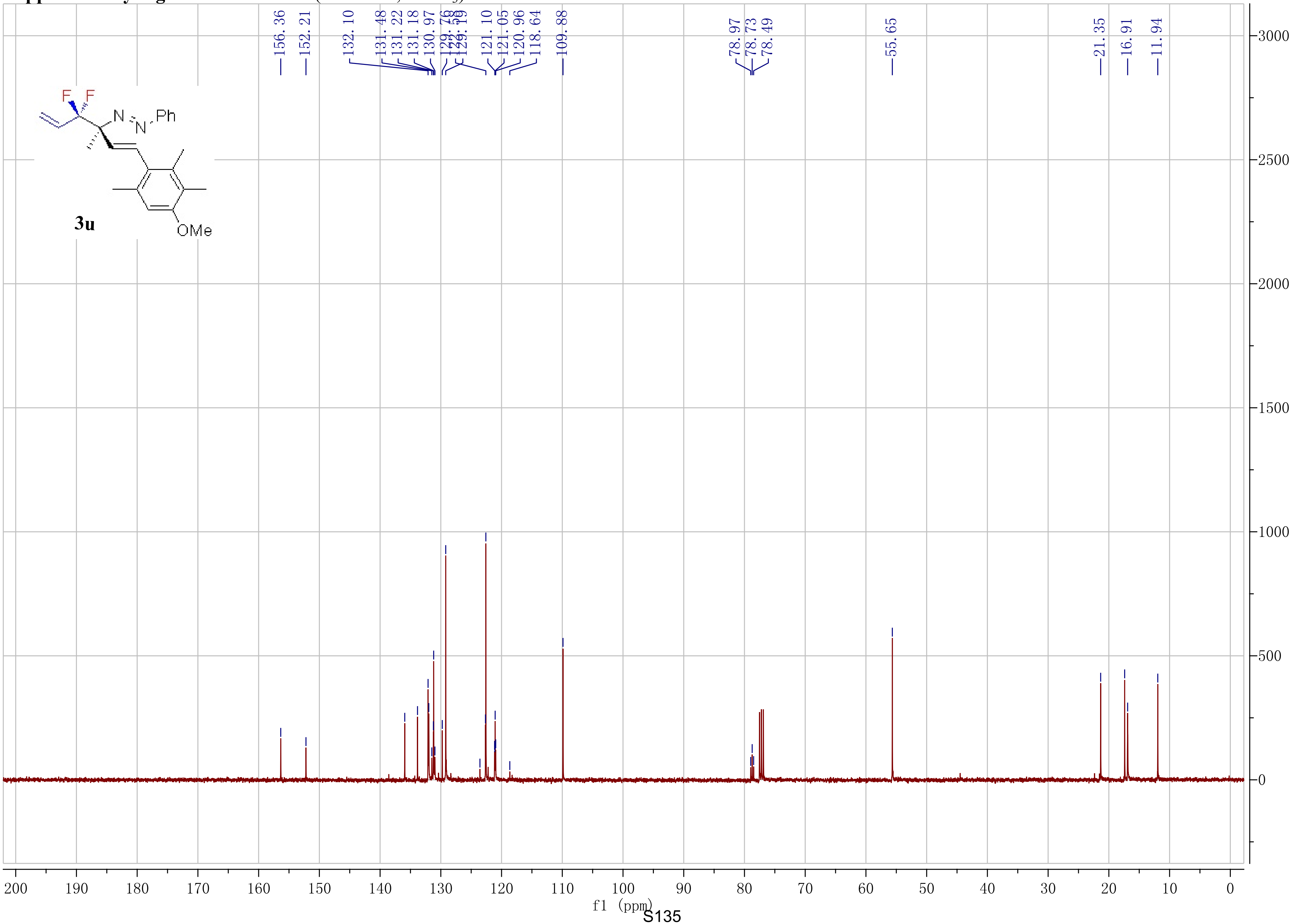

Supplementary Figure 106 ^{19}F NMR (376 MHz, $CDCl_3$) of **3u**

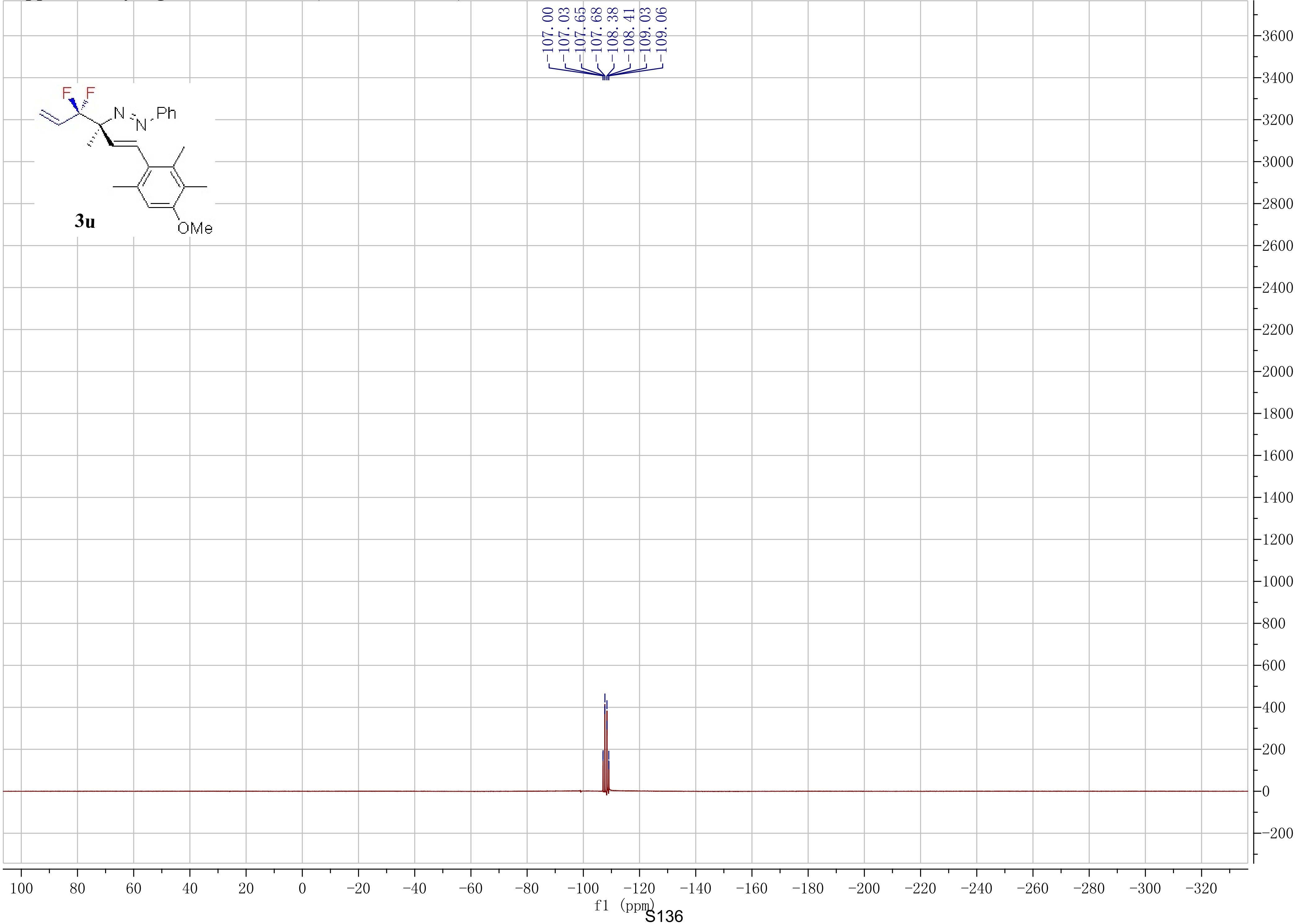

3u

Supplementary Figure 107 HPLC spectra of racemic **3u**

9743 HS-14-6-9+- OJH 982 214 0.7

Sample Name:	**HS-14-6-9+- OJH 982 214 0.7**	*Injection Volume:*	**2.0**
Vial Number:	**RE6**	*Channel:*	**UV_VIS_2**
Sample Type:	**unknown**	*Wavelength:*	**214.0**
Control Program:	**test-dad2**	*Bandwidth:*	**4**
Quantif. Method:	**20170608**	*Dilution Factor:*	**1.0000**
Recording Time:	**2020-11-17 16:22**	*Sample Weight:*	**1.0000**
Run Time (min):	**27.56**	*Sample Amount:*	**1.0000**

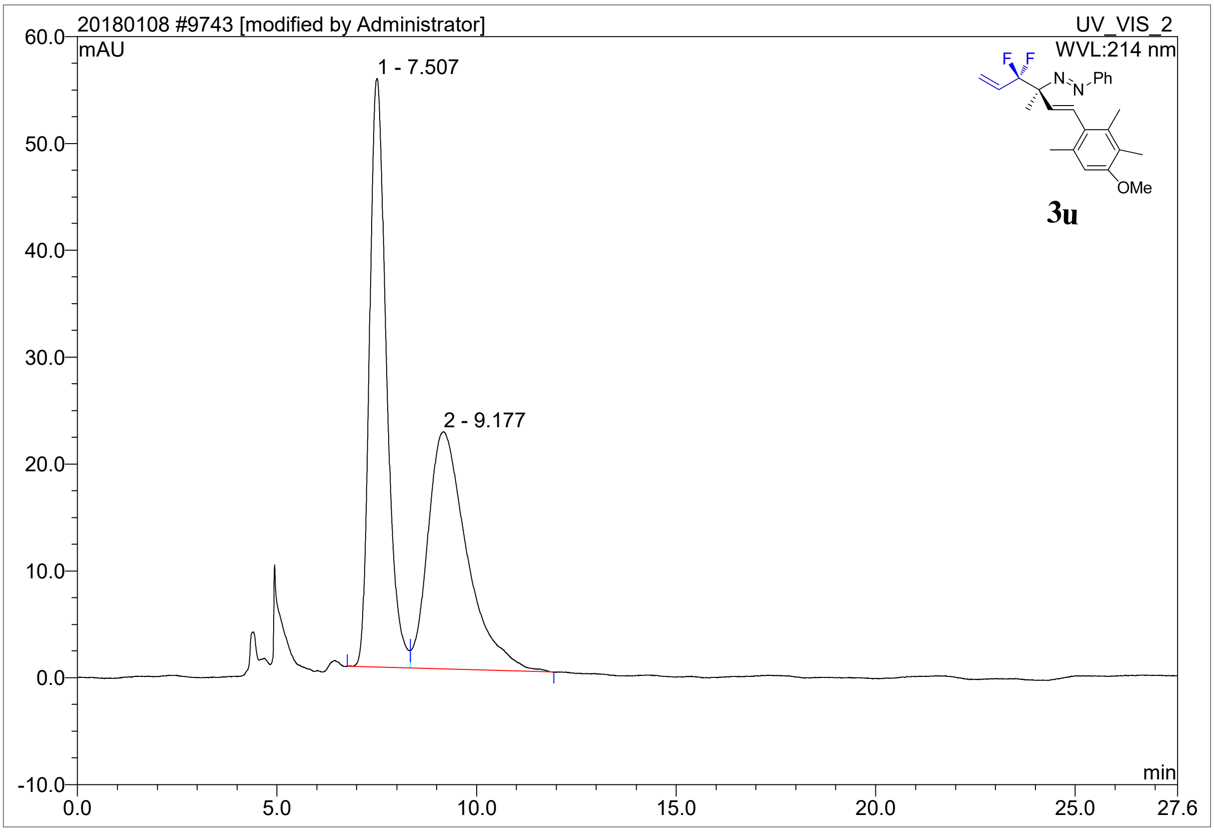

No.	Ret.Time min	Peak Name	Height mAU	Area mAU*min	Rel.Area %	Amount	Type
1	7.51	n.a.	55.091	27.160	51.93	n.a.	BM *
2	9.18	n.a.	22.198	25.136	48.07	n.a.	MB*
**Total:**			77.288	52.296	100.00	0.000	

Chromeleon (c) Dionex 1996-2006
Version 6.80 SR14 Build 4527 (238909)

Supplementary Figure 108 HPLC spectra of (*S*)-**3u**

9744 HS-14-7-11 OJH 982 214 0.7

Sample Name:	**HS-14-7-11 OJH 982 214 0.7**	*Injection Volume:*	**2.0**
Vial Number:	**RE7**	*Channel:*	**UV_VIS_2**
Sample Type:	**unknown**	*Wavelength:*	**214.0**
Control Program:	**test-dad2**	*Bandwidth:*	**4**
Quantif. Method:	**20170608**	*Dilution Factor:*	**1.0000**
Recording Time:	**2020-11-17 16:54**	*Sample Weight:*	**1.0000**
Run Time (min):	**13.08**	*Sample Amount:*	**1.0000**

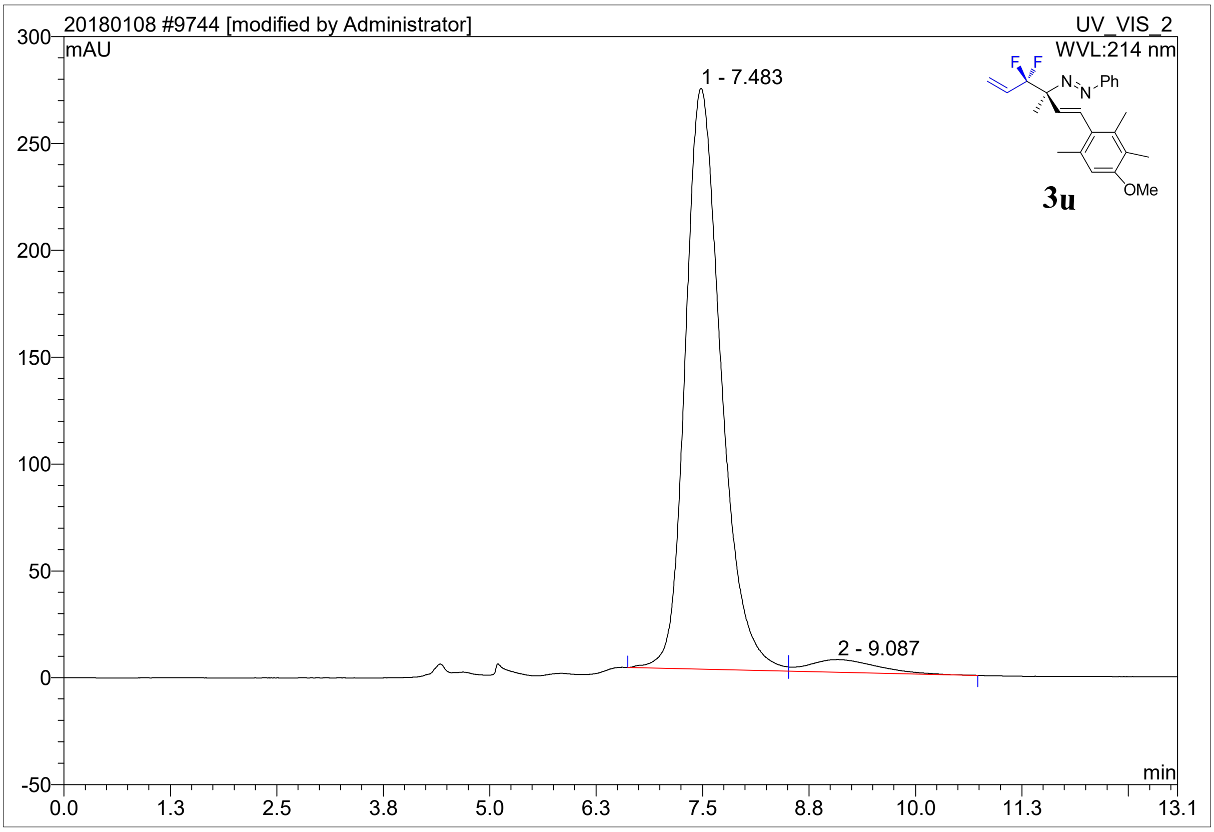

No.	Ret.Time min	Peak Name	Height mAU	Area mAU*min	Rel.Area %	Amount	Type
1	7.48	n.a.	271.750	132.823	96.09	n.a.	BM *
2	9.09	n.a.	5.917	5.406	3.91	n.a.	MB*
**Total:**			277.667	138.229	100.00	0.000	

Supplementary Figure 109 ^{1}H NMR (400 MHz, $CDCl_3$) of **3v**

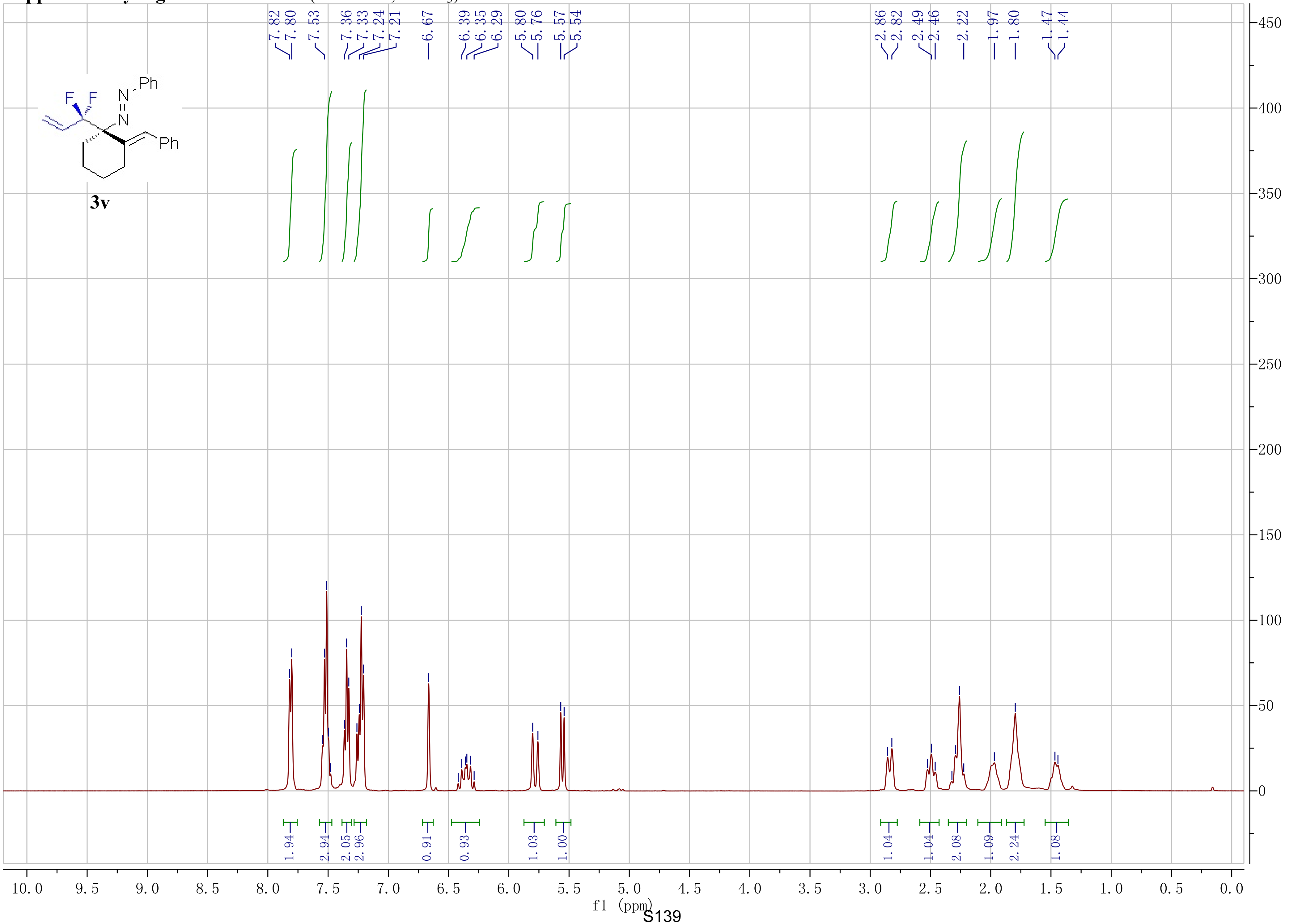

Supplementary Figure 110 ^{13}C NMR (101 MHz, $CDCl_3$) of **3v**

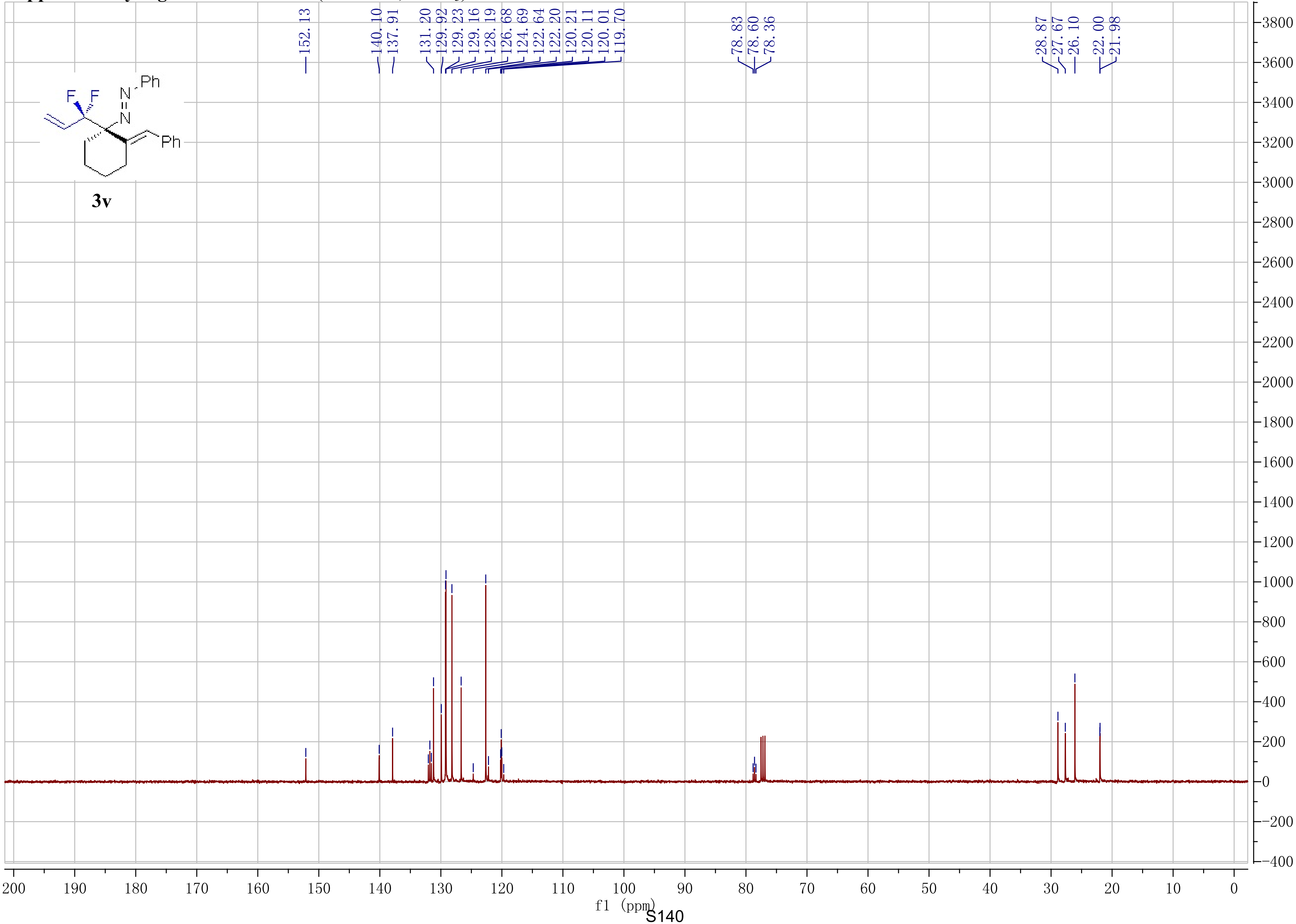

Supplementary Figure 111 ^{19}F NMR (376 MHz, $CDCl_3$) of **3v**

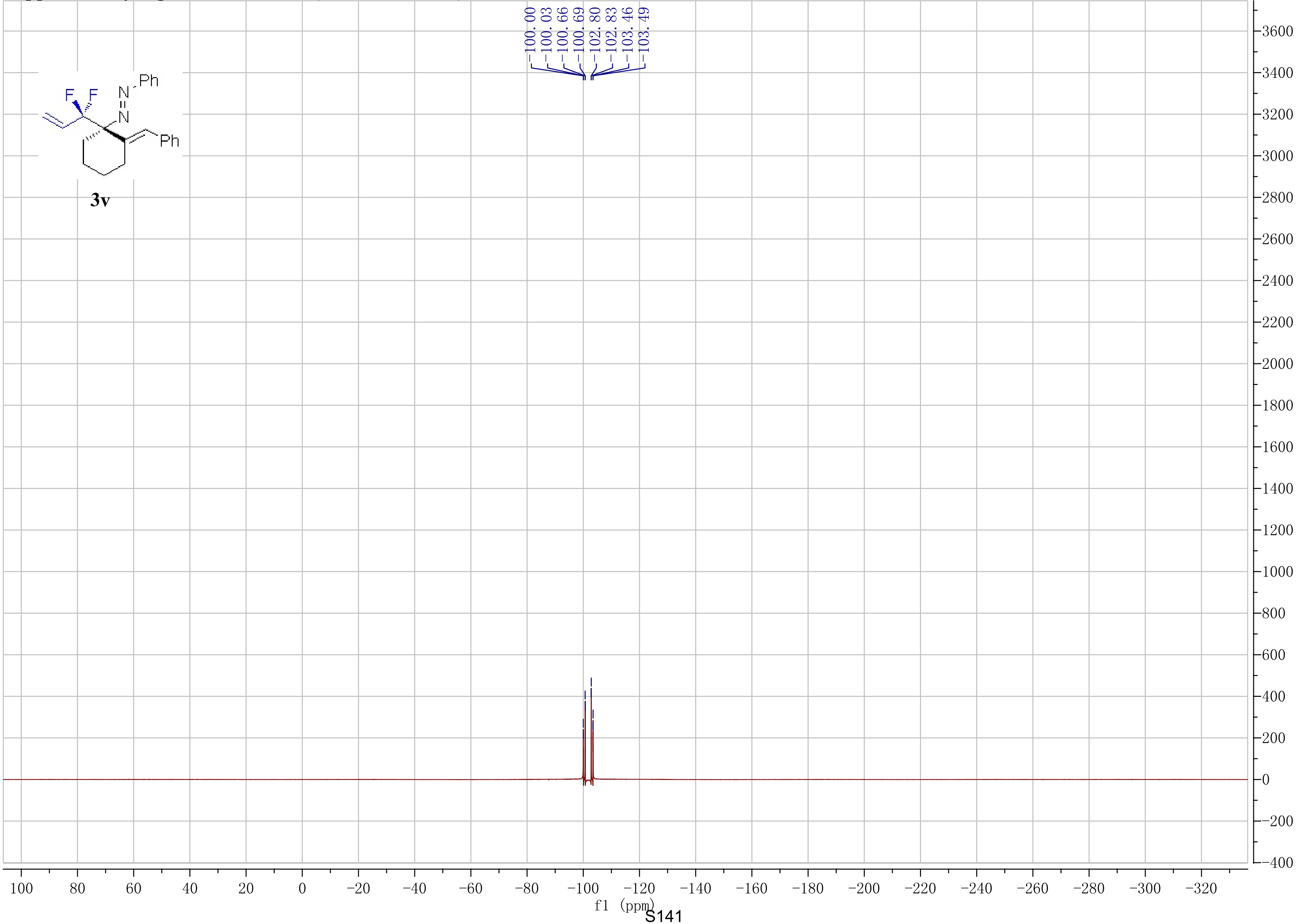

Supplementary Figure 112 HPLC spectra of racemic **3v**

Chromatogram and Results

Injection Details

Injection Name:	**HS-13-46-10+- PC3 A7W3 214 0.7**	*Run Time (min):*	**40.00**
Vial Number:	**RE4**	*Injection Volume:*	**5.00**
Injection Type:	**Unknown**	*Channel:*	**UV_VIS_1**
Calibration Level:		*Wavelength:*	**214.0**
Instrument Method:	**20160223-DAD3**	*Bandwidth:*	**4**
Processing Method:	**20160223**	*Dilution Factor:*	**1.0000**
Injection Date/Time:	**03/06/20 18:51**	*Sample Weight:*	**1.0000**

Chromatogram

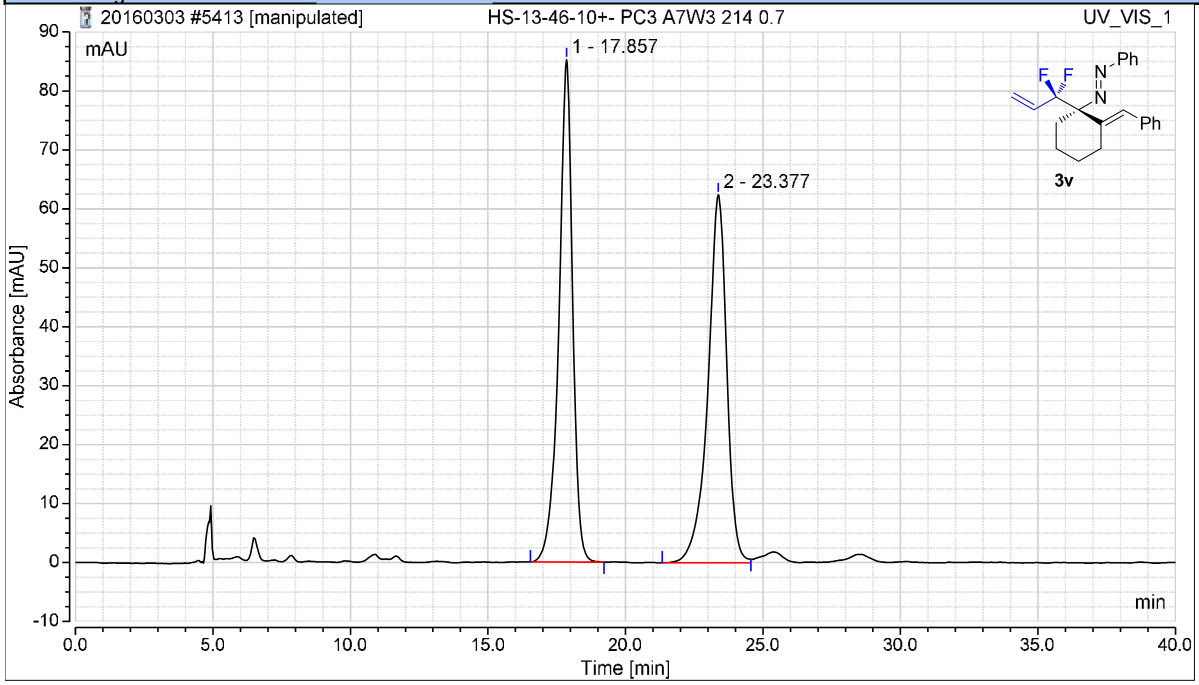

Integration Results

No.	Retention Time min	Area mAU*min	Height mAU	Relative Area %
1	17.857	49.3741	85.1999	49.924
2	23.377	49.5243	62.4397	50.076
**Total:**		**98.898**	**1401.998**	**100.000**

Supplementary Figure 113 HPLC spectra of (*S*)-**3v**

Chromatogram and Results

Injection Details

Injection Name:	**HS-13-56-7 PC3 A7W3 214 0.7**	*Run Time (min):*	**39.23**
Vial Number:	**RA2**	*Injection Volume:*	**5.00**
Injection Type:	**Unknown**	*Channel:*	**UV_VIS_1**
Calibration Level:		*Wavelength:*	**214.0**
Instrument Method:	**20160223-DAD3**	*Bandwidth:*	**4**
Processing Method:	**20160223**	*Dilution Factor:*	**1.0000**
Injection Date/Time:	**04/06/20 10:33**	*Sample Weight:*	**1.0000**

Chromatogram

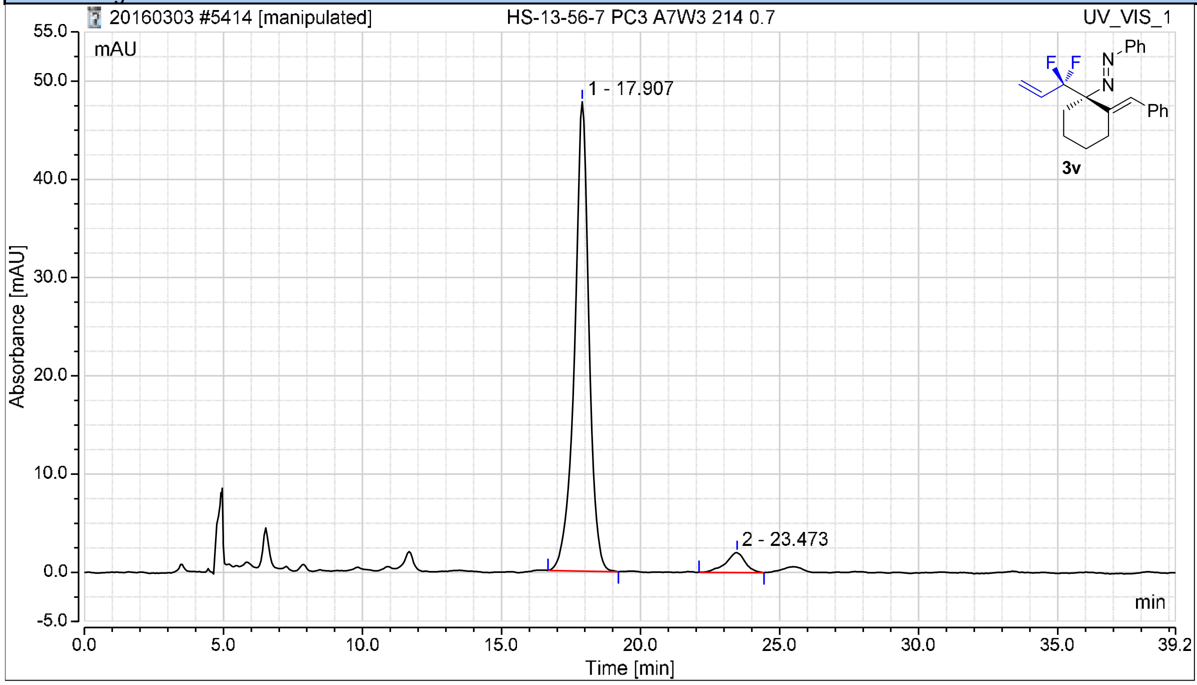

Integration Results

No.	Retention Time min	Area mAU*min	Height mAU	Relative Area %
1	17.907	28.7848	47.7695	94.577
2	23.473	1.6504	2.0303	5.423
**Total:**		**30.435**	**1401.998**	**100.000**

Supplementary Figure 114 ^1H NMR (400 MHz, $CDCl_3$) of **3w**

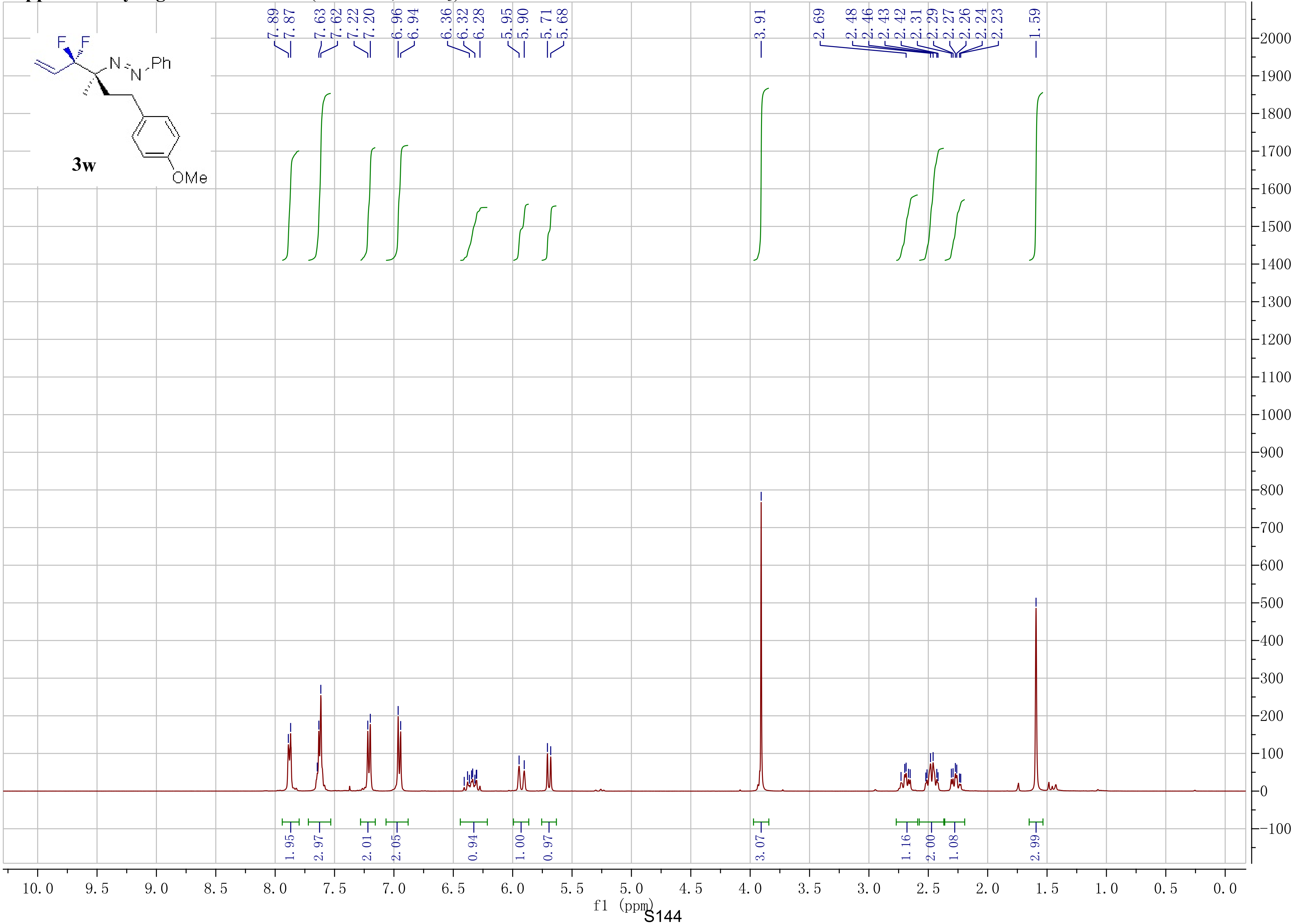

Supplementary Figure 115 ^{13}C NMR (101 MHz, $CDCl_3$) of **3w**

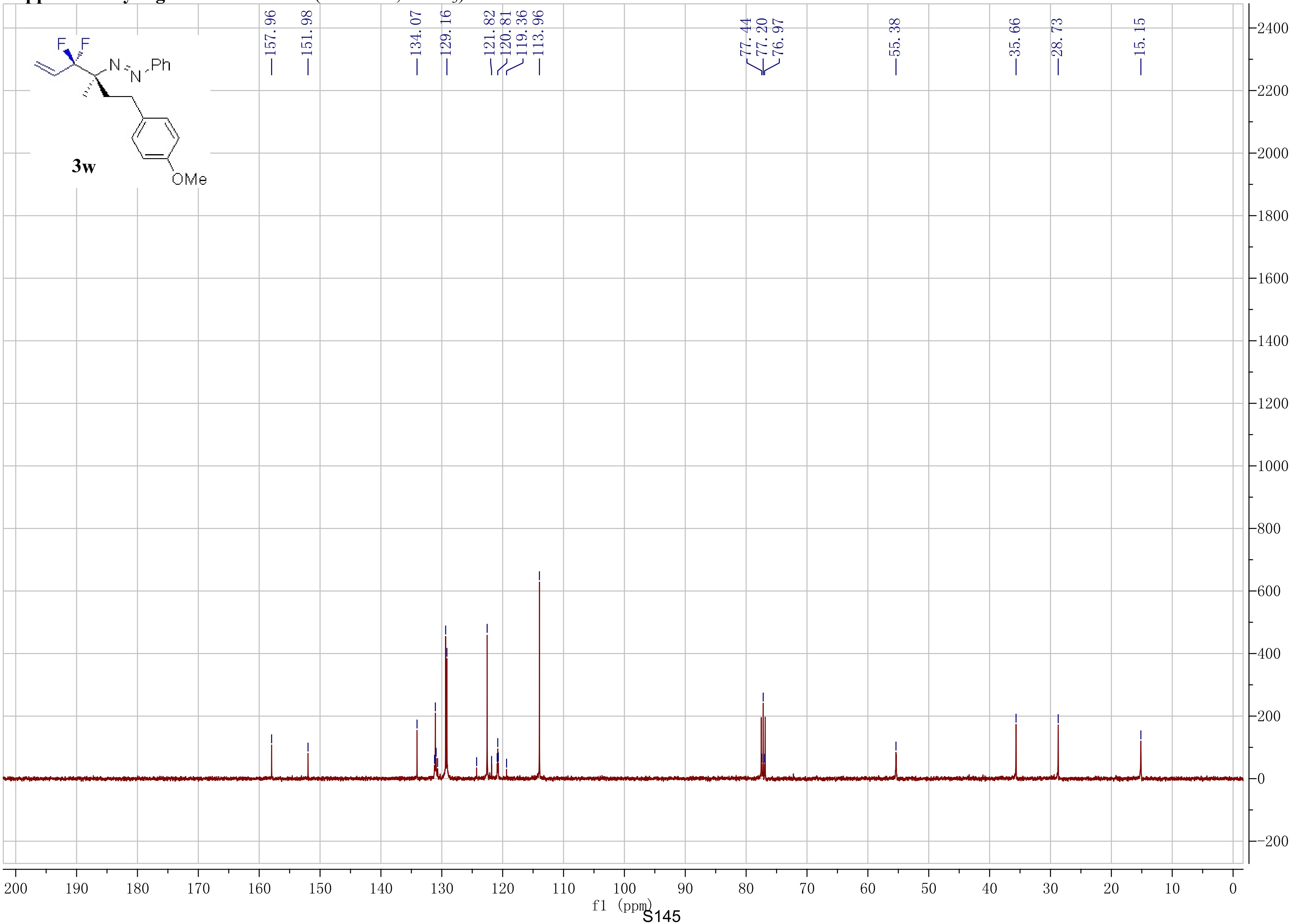

Supplementary Figure 116 ^{19}F NMR (376 MHz, $CDCl_3$) of **3w**

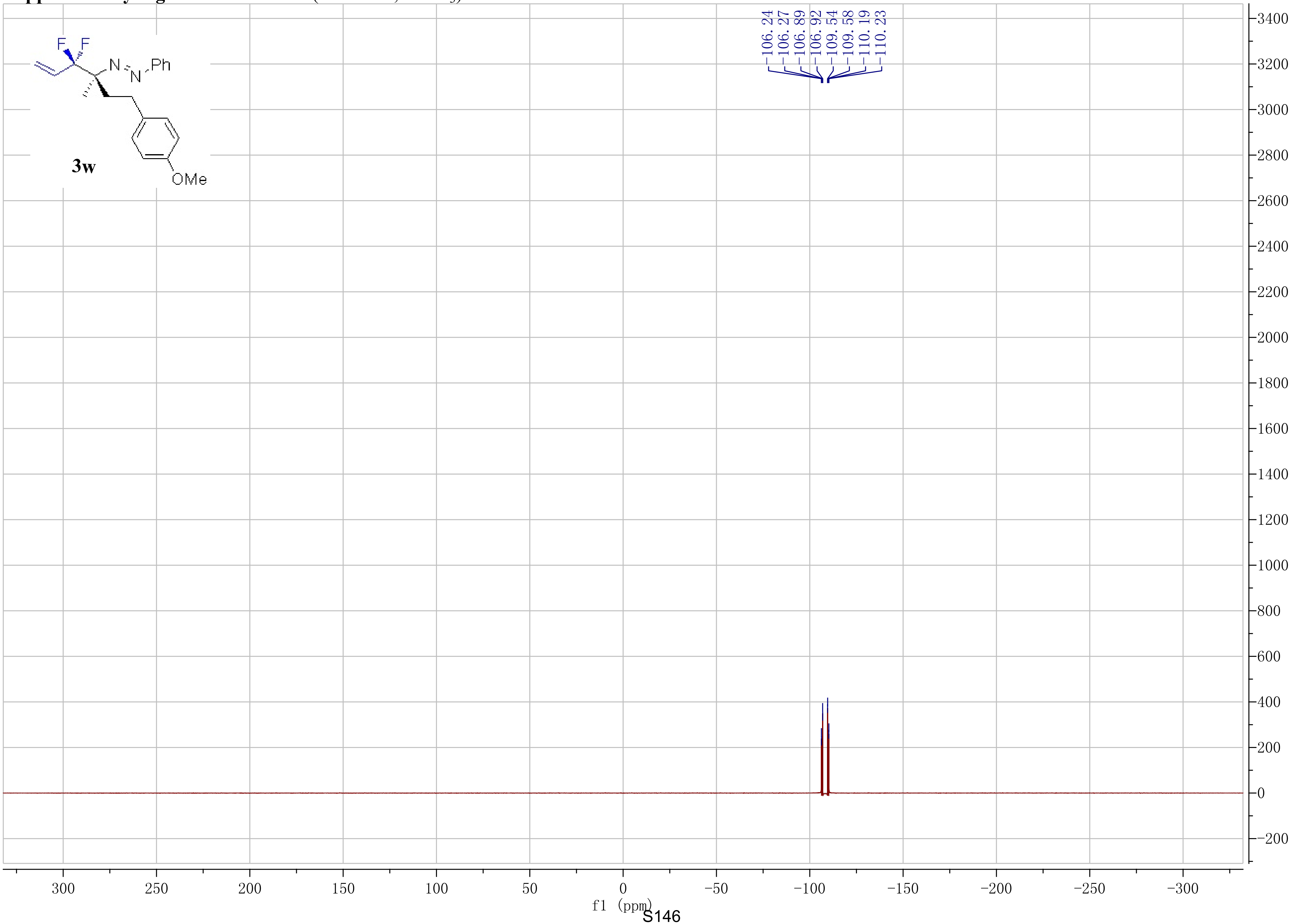

Supplementary Figure 117 HPLC spectra of racemic **3w**

Chromatogram and Results

Injection Details

Injection Name:	**HS-13-46-3+- PC3 A6W4 214 0.7**	*Run Time (min):*	**45.98**
Vial Number:	**RD1**	*Injection Volume:*	**2.00**
Injection Type:	**Unknown**	*Channel:*	**UV_VIS_1**
Calibration Level:		*Wavelength:*	**214.0**
Instrument Method:	**20160223-DAD3**	*Bandwidth:*	**4**
Processing Method:	**20160223**	*Dilution Factor:*	**1.0000**
Injection Date/Time:	**07/05/20 10:42**	*Sample Weight:*	**1.0000**

Chromatogram

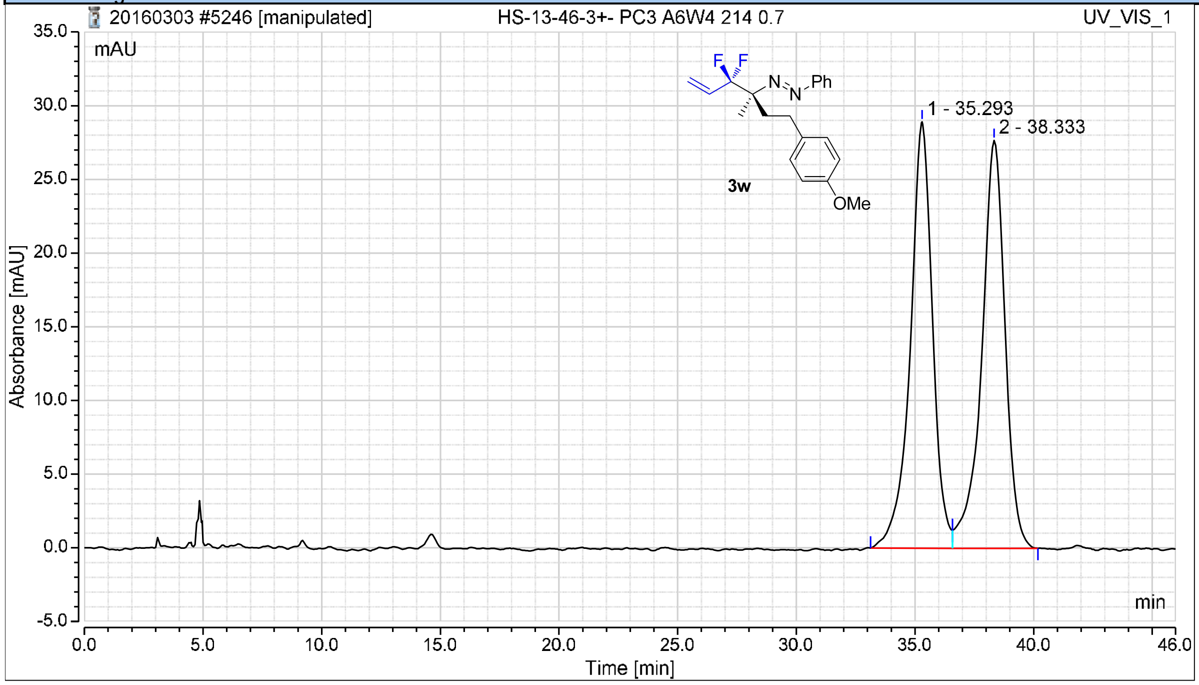

Integration Results

No.	Retention Time<br>min	Area<br>mAU*min	Height<br>mAU	Relative Area<br>%
1	35.293	31.4585	28.9353	49.949
2	38.333	31.5222	27.6795	50.051
**Total:**		**62.981**	**1401.998**	**100.000**

Supplementary Figure 118 HPLC spectra of (*S*)-**3w**

Instrument:U3000 Sequence:20160303

Chromatogram and Results

Injection Details

Injection Name:	**HS-13-56-5 PC3 A6W4 214 0.7**	*Run Time (min):*	**57.19**
Vial Number:	**RD2**	*Injection Volume:*	**2.00**
Injection Type:	**Unknown**	*Channel:*	**UV_VIS_1**
Calibration Level:		*Wavelength:*	**214.0**
Instrument Method:	**20160223-DAD3**	*Bandwidth:*	**4**
Processing Method:	**20160223**	*Dilution Factor:*	**1.0000**
Injection Date/Time:	**07/05/20 11:36**	*Sample Weight:*	**1.0000**

Chromatogram

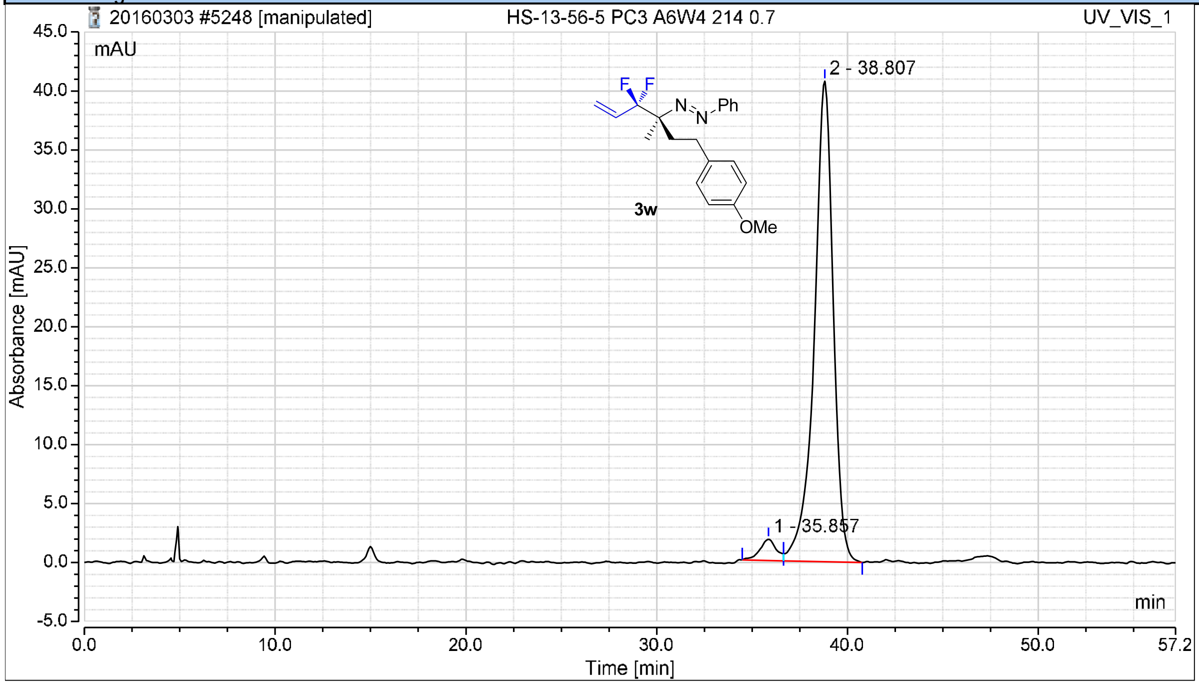

Integration Results

No.	Retention Time<br>min	Area<br>mAU*min	Height<br>mAU	Relative Area<br>%
1	35.857	1.7905	1.7859	3.678
2	38.807	46.8967	40.7445	96.322
**Total:**		**48.687**	**1401.998**	**100.000**

Supplementary Figure 119 ^{1}H NMR (400 MHz, $CDCl_3$) of **3x**

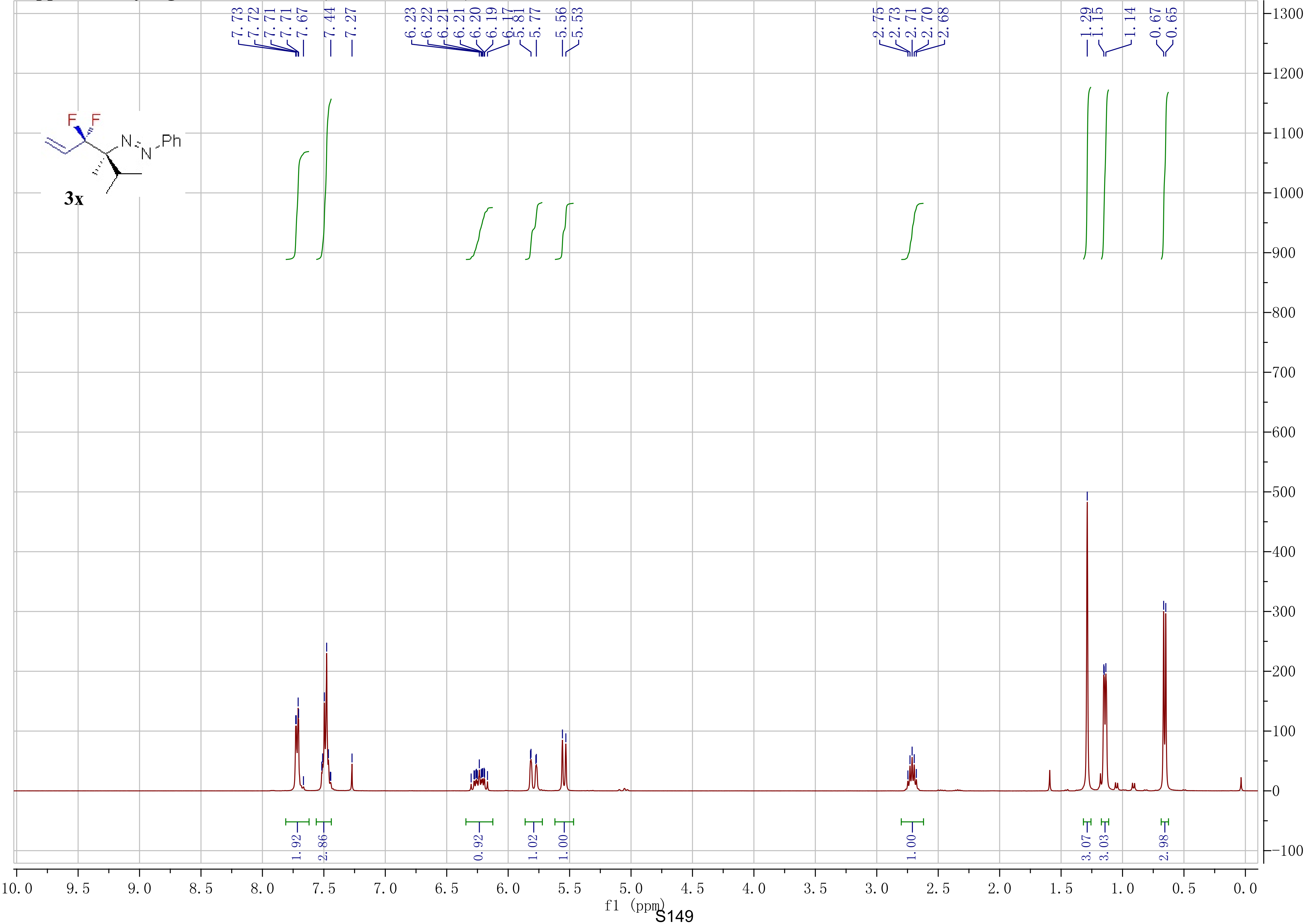

Supplementary Figure 120 ^{13}C NMR (101 MHz, $CDCl_3$) of **3x**

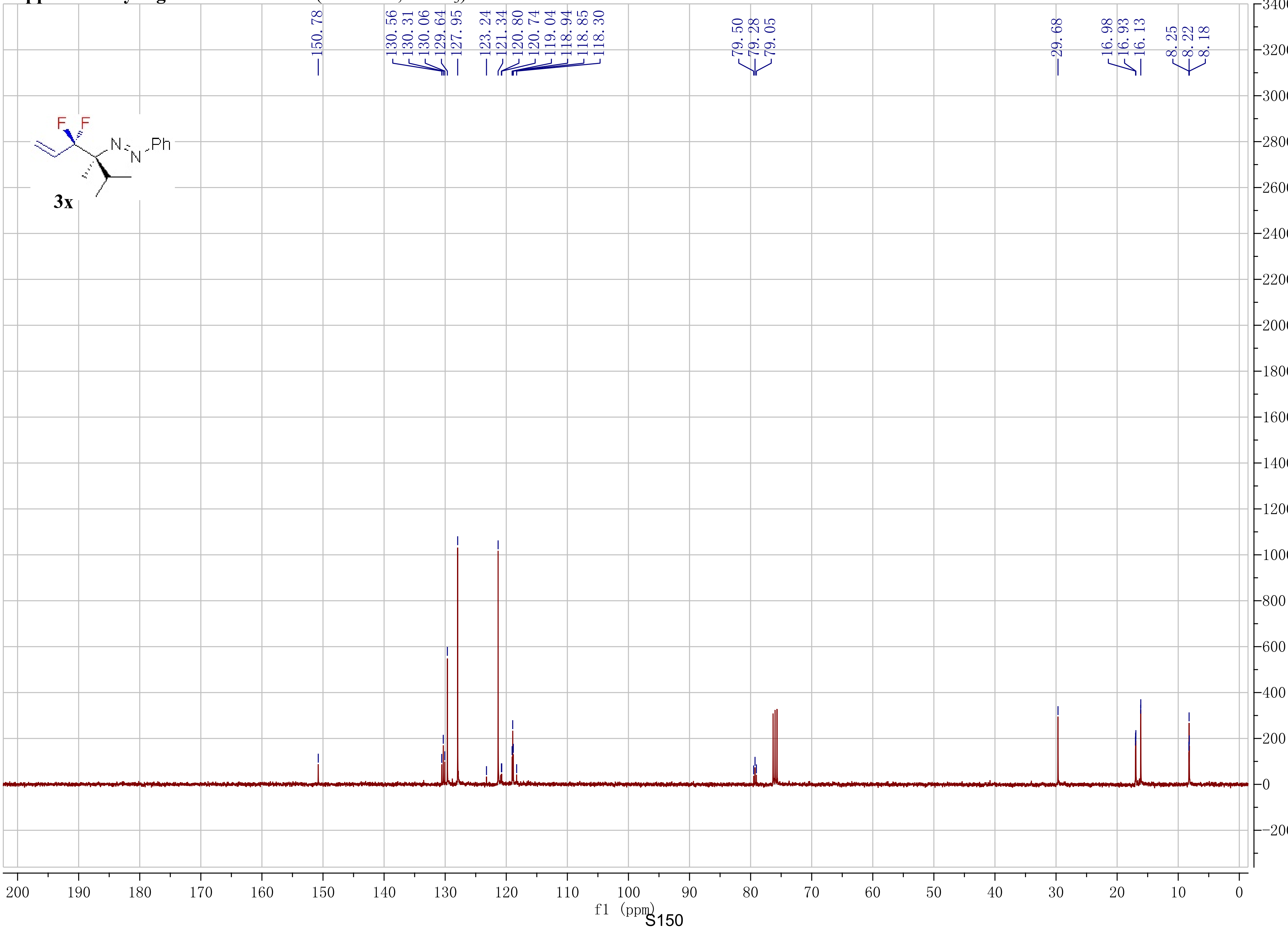

Supplementary Figure 121 ^{19}F NMR (376 MHz, $CDCl_3$) of **3x**

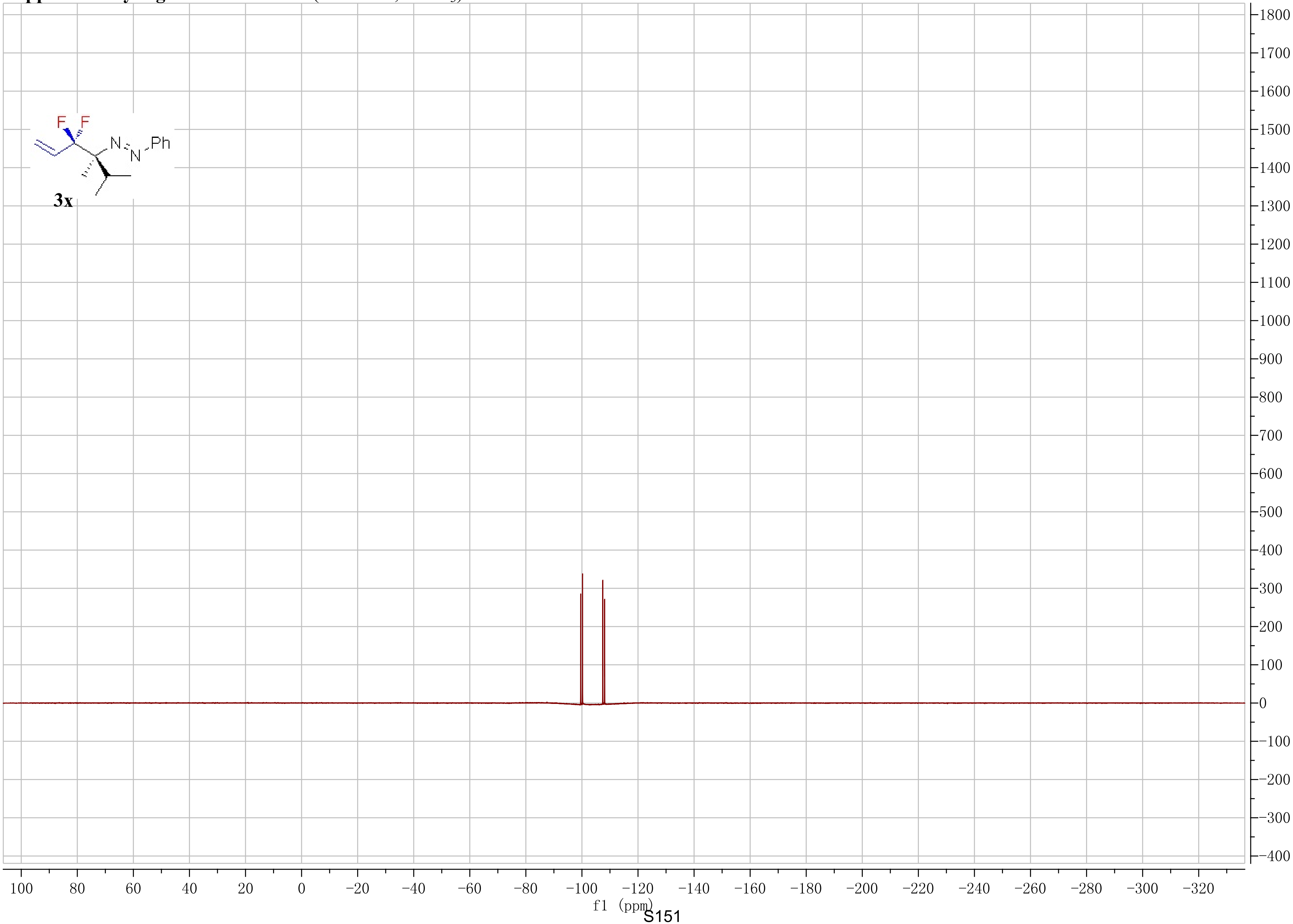

Supplementary Figure 122 HPLC spectra of racemic **3x**

9855 HS-14-36-3+- OJH 955 214 0.7

Sample Name:	**HS-14-36-3+- OJH 955 214 0.7**	*Injection Volume:*	**2.0**
Vial Number:	**RA5**	*Channel:*	**UV_VIS_2**
Sample Type:	**unknown**	*Wavelength:*	**214.0**
Control Program:	**test-dad3**	*Bandwidth:*	**4**
Quantif. Method:	**20170608**	*Dilution Factor:*	**1.0000**
Recording Time:	**2020-12-3 14:21**	*Sample Weight:*	**1.0000**
Run Time (min):	**29.94**	*Sample Amount:*	**1.0000**

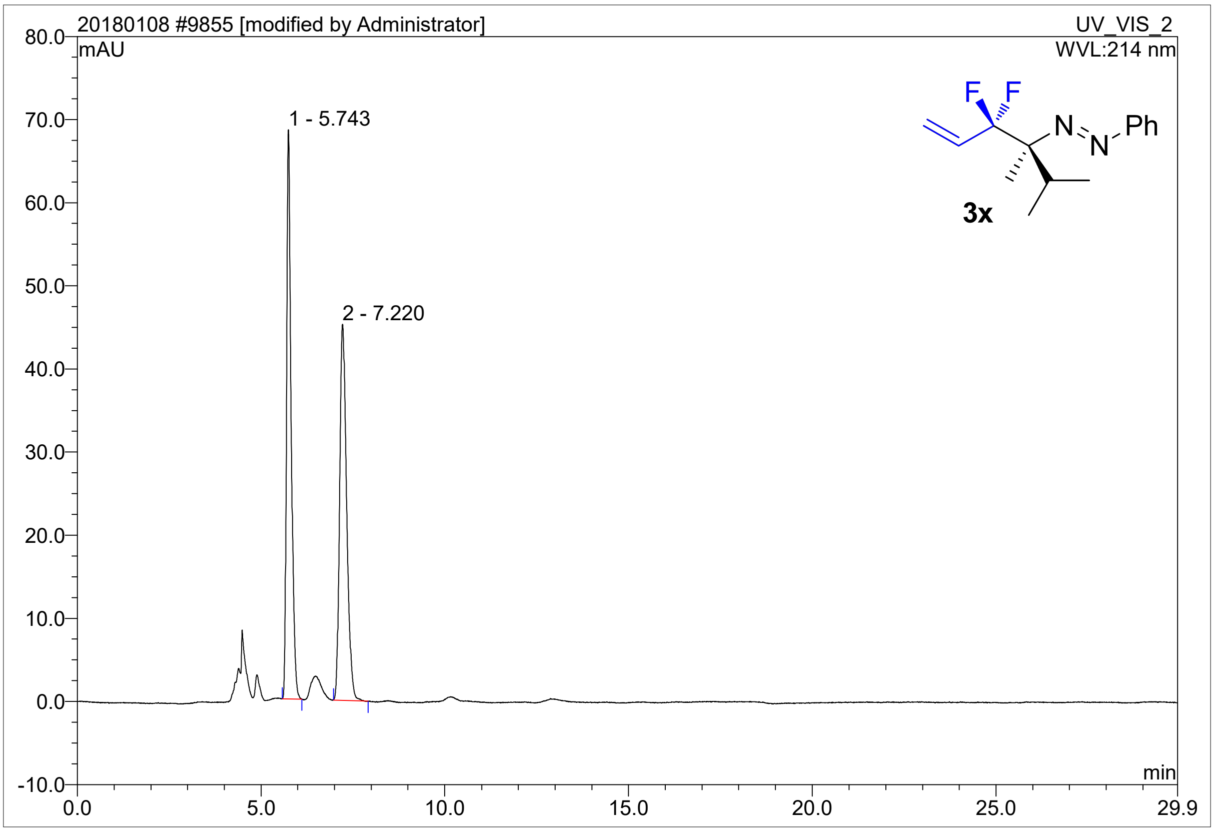

No.	Ret.Time min	Peak Name	Height mAU	Area mAU*min	Rel.Area %	Amount	Type
1	5.74	n.a.	68.488	10.041	50.12	n.a.	BMB
2	7.22	n.a.	45.255	9.995	49.88	n.a.	BMB*
**Total:**			113.743	20.037	100.00	0.000	

Supplementary Figure 123 HPLC spectra of (*S*)-**3x**

9856 HS-14-37-3 OJH 955 214 0.7

Sample Name:	**HS-14-37-3 OJH 955 214 0.7**	*Injection Volume:*	**2.0**
Vial Number:	**RB5**	*Channel:*	**UV_VIS_2**
Sample Type:	**unknown**	*Wavelength:*	**214.0**
Control Program:	**test-dad3**	*Bandwidth:*	**4**
Quantif. Method:	**20170608**	*Dilution Factor:*	**1.0000**
Recording Time:	**2020-12-3 14:08**	*Sample Weight:*	**1.0000**
Run Time (min):	**11.20**	*Sample Amount:*	**1.0000**

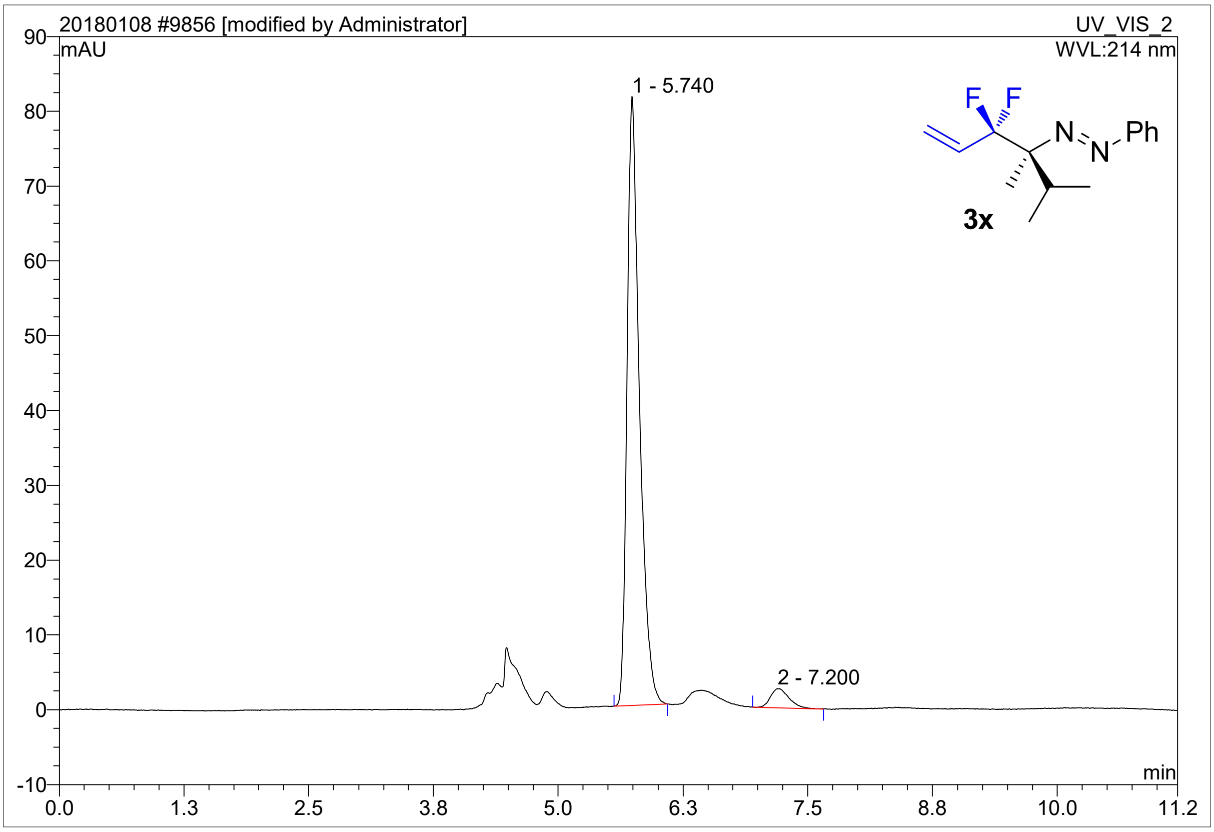

No.	Ret.Time min	Peak Name	Height mAU	Area mAU*min	Rel.Area %	Amount	Type
1	5.74	n.a.	81.426	11.904	95.62	n.a.	BMB
2	7.20	n.a.	2.554	0.546	4.38	n.a.	BMB*
**Total:**			83.980	12.450	100.00	0.000	

Supplementary Figure 124 ^{1}H NMR (400 MHz, $CDCl_3$) of **3y**

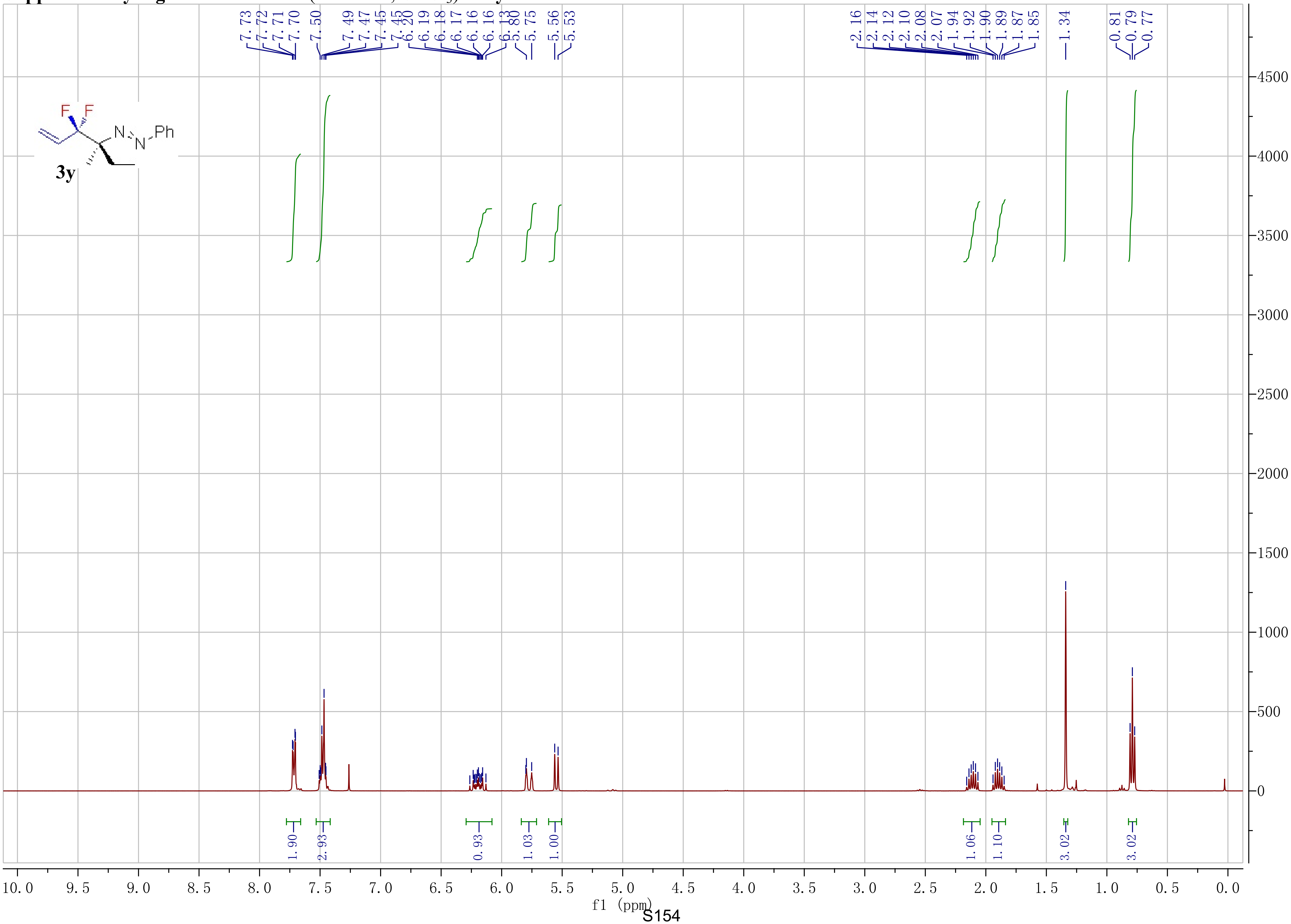

Supplementary Figure 125 ^{13}C NMR (101 MHz, $CDCl_3$) of **3y**

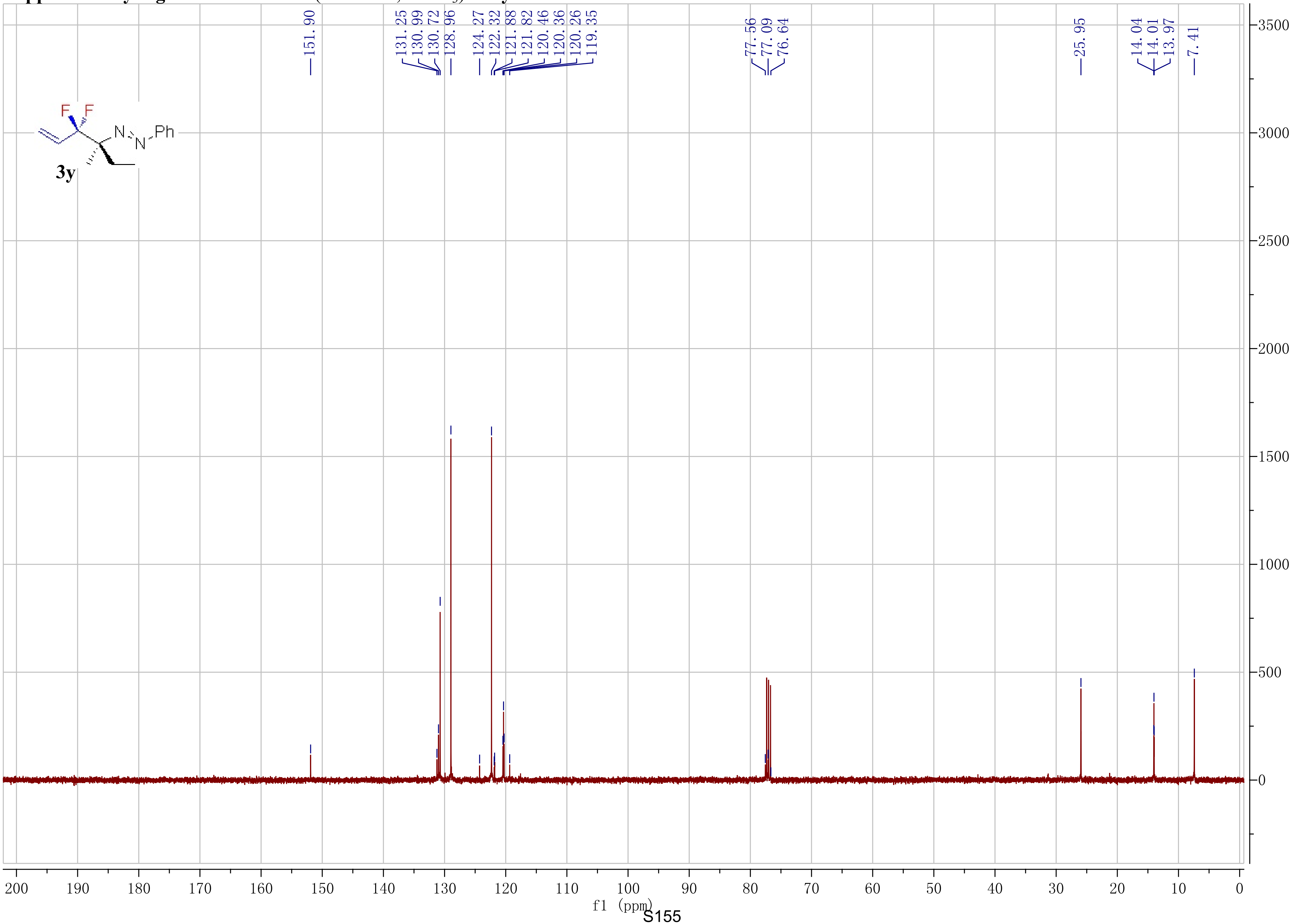

Supplementary Figure 126 ^{19}F NMR (376 MHz, $CDCl_3$) of **3y**

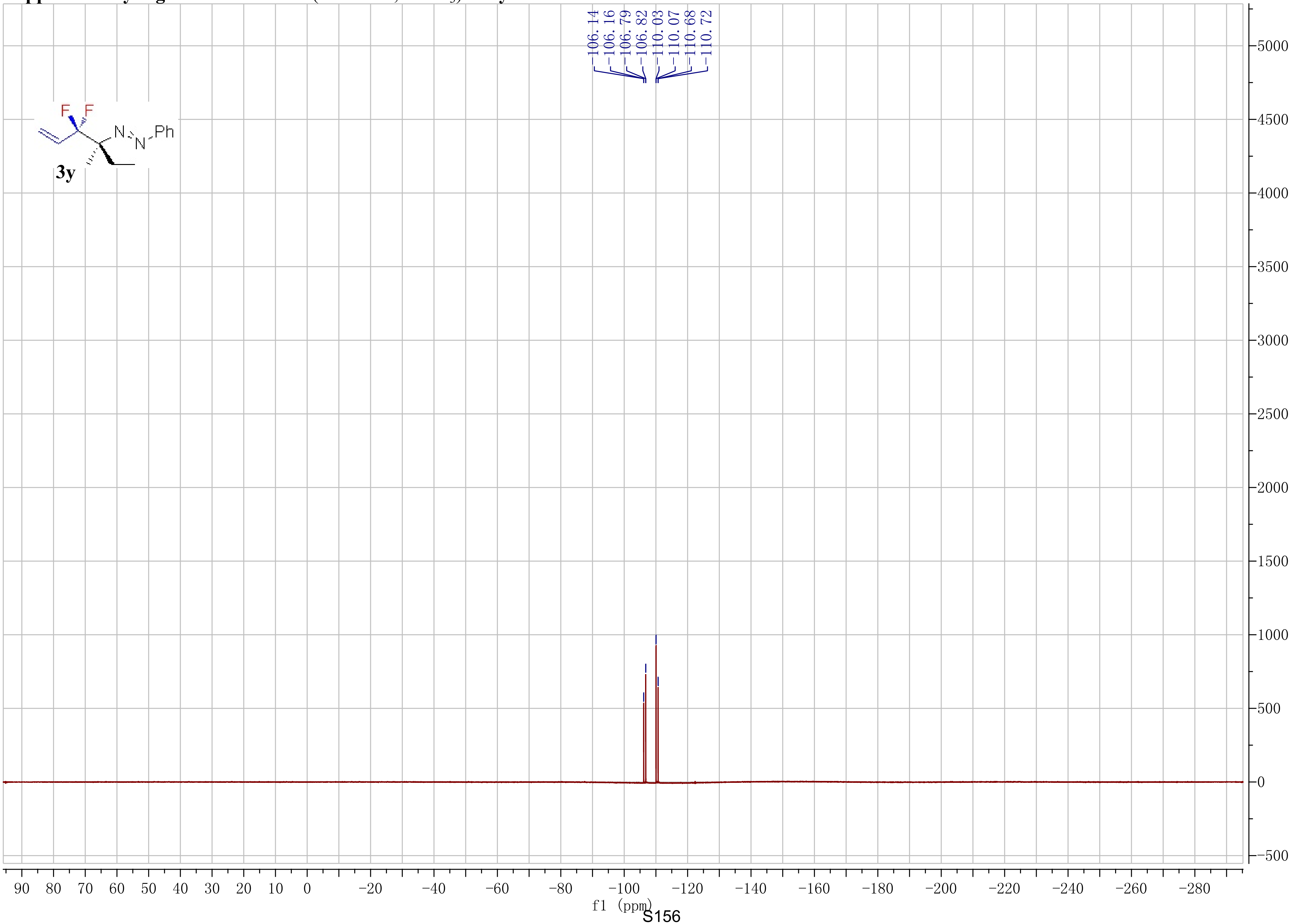

Supplementary Figure 127 HPLC spectra of racemic **3y**

HS-14-36-1+- OJH 982 214 0.7

Sample Name:	**HS-14-36-1+- OJH 982 214 0.7**	*Injection Volume:*	**2.0**
Vial Number:	**GD1**	*Channel:*	**UV_VIS_1**
Sample Type:	**unknown**	*Wavelength:*	**214**
Control Program:	**201701-5**	*Bandwidth:*	**n.a.**
Quantif. Method:	**201701**	*Dilution Factor:*	**1.0000**
Recording Time:	**2020/12/10 12:19**	*Sample Weight:*	**1.0000**
Run Time (min):	**19.03**	*Sample Amount:*	**1.0000**

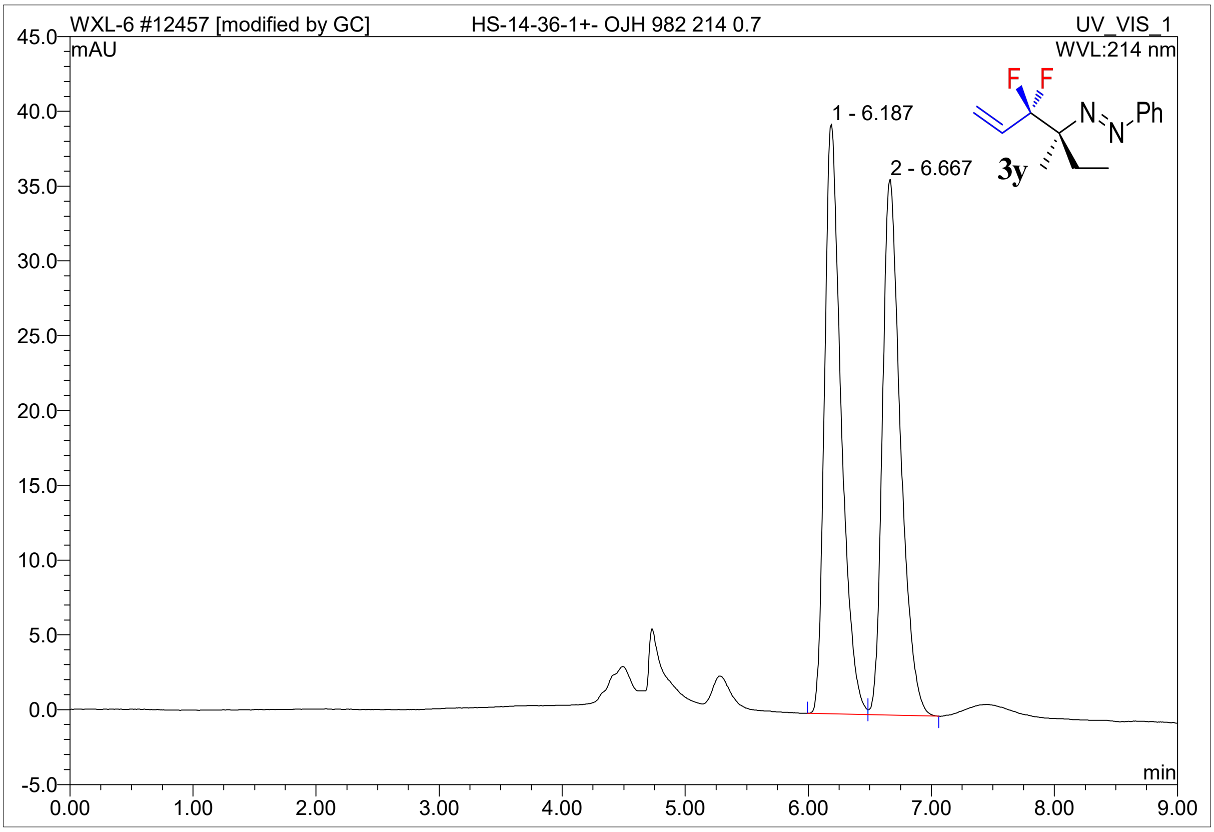

No.	Ret.Time min	Peak Name	Height mAU	Area mAU*min	Rel.Area %	Amount	Type
1	6.19	n.a.	39.428	6.238	50.31	n.a.	BM
2	6.67	n.a.	35.814	6.161	49.69	n.a.	MB
**Total:**			75.242	12.398	100.00	0.000	

Supplementary Figure 128 HPLC spectra of (*S*)-**3y**

HS-14-37-5 OJH 982 214 0.7

Sample Name:	**HS-14-37-5 OJH 982 214 0.7**	*Injection Volume:*	**2.0**
Vial Number:	**GD2**	*Channel:*	**UV_VIS_1**
Sample Type:	**unknown**	*Wavelength:*	**214**
Control Program:	**201701-5**	*Bandwidth:*	**n.a.**
Quantif. Method:	**201701**	*Dilution Factor:*	**1.0000**
Recording Time:	**2020/12/10 12:40**	*Sample Weight:*	**1.0000**
Run Time (min):	**43.87**	*Sample Amount:*	**1.0000**

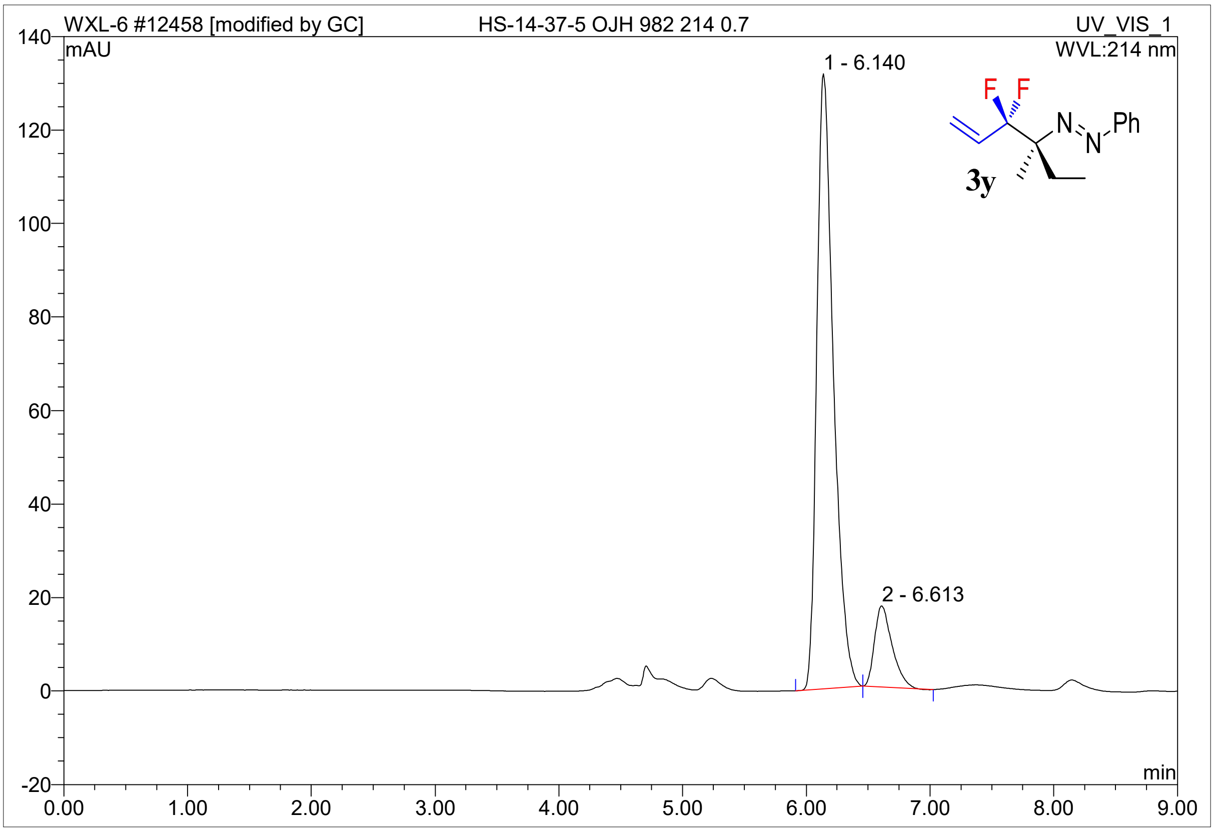

No.	Ret.Time min	Peak Name	Height mAU	Area mAU*min	Rel.Area %	Amount	Type
1	6.14	n.a.	131.567	20.516	87.73	n.a.	BMb*
2	6.61	n.a.	17.406	2.870	12.27	n.a.	bMB*
**Total:**			148.972	23.386	100.00	0.000	

Chromeleon (c) Dionex 1996-2006
Version 6.80 SR12 Build 3578 (207169)

Supplementary Figure 129 ^{1}H NMR (400 MHz, $CDCl_3$) of **4a**

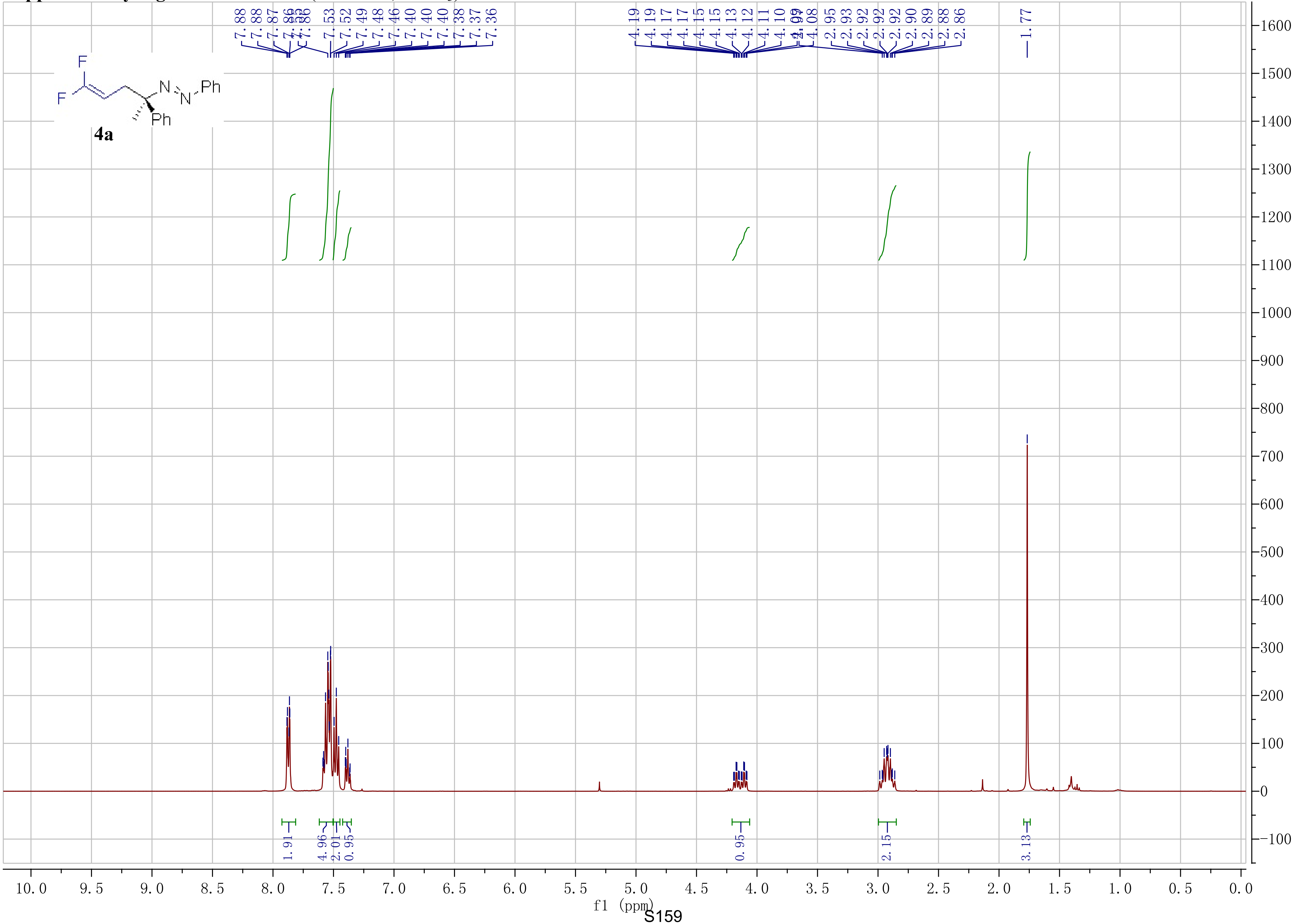

Supplementary Figure 130 ^{13}C NMR (101 MHz, $CDCl_3$) of **4a**

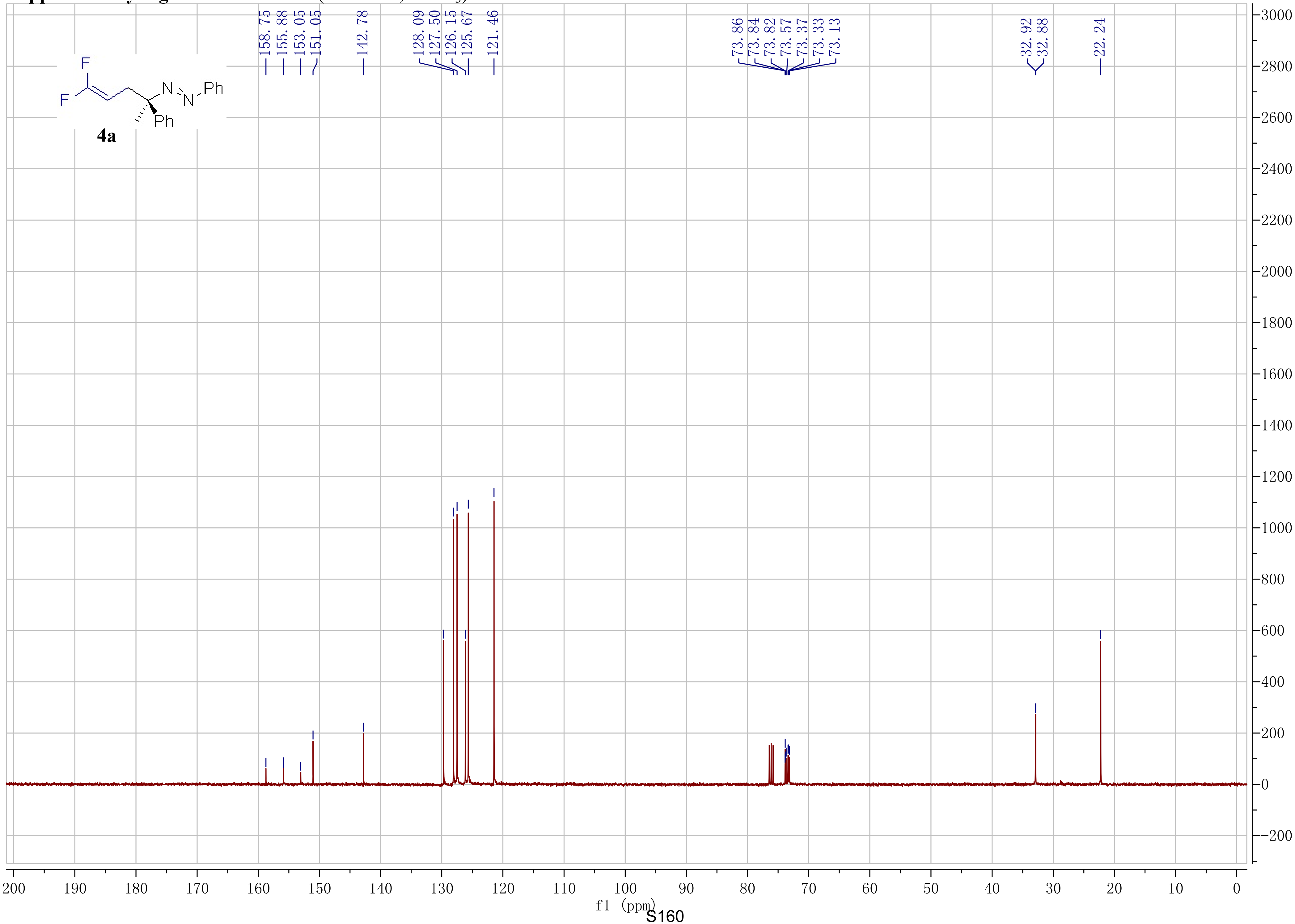

Supplementary Figure 131 ^{19}F NMR (376 MHz, $CDCl_3$) of **4a**

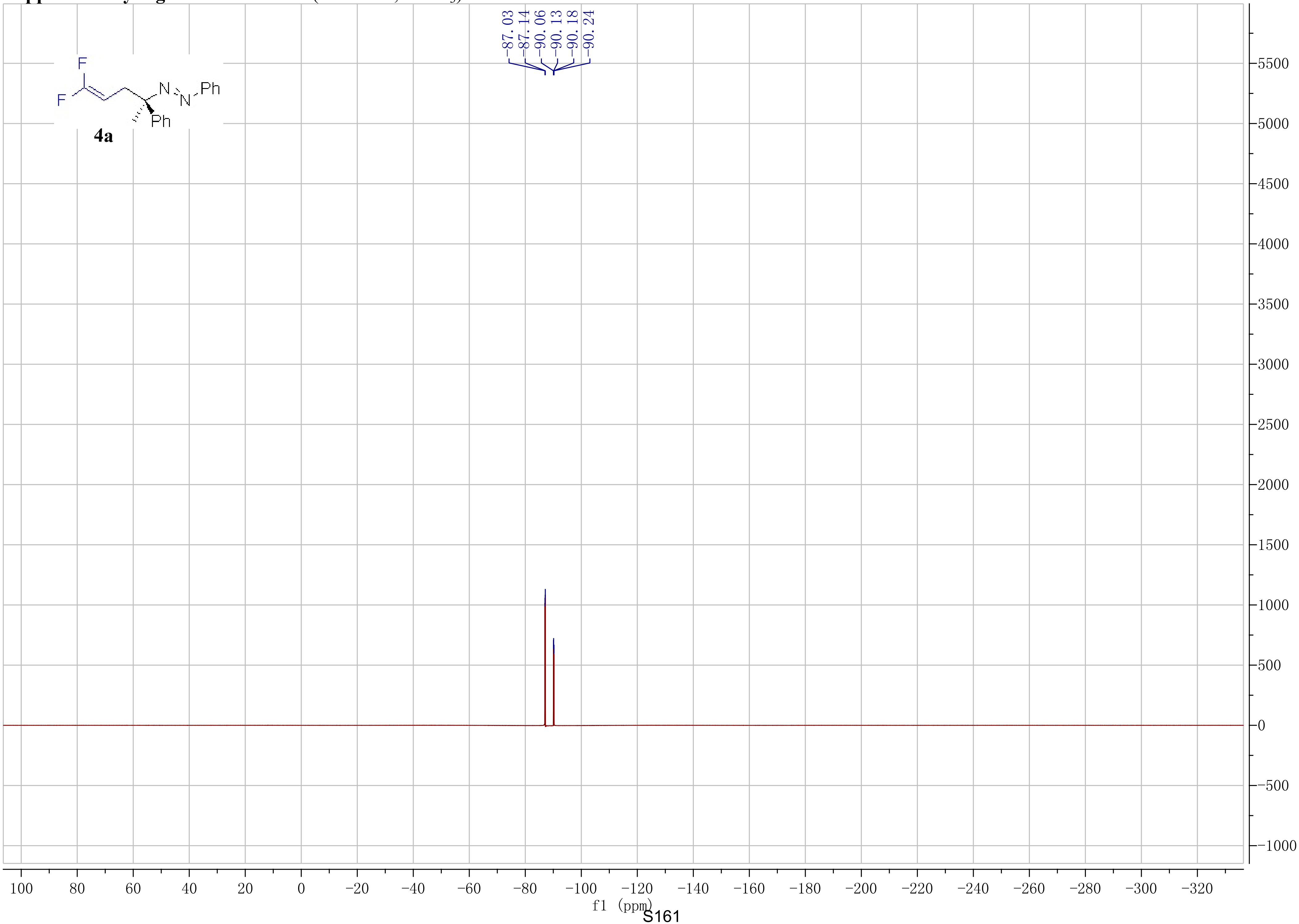

Supplementary Figure 132 ^{1}H NMR (400 MHz, $CDCl_3$) of **5a**

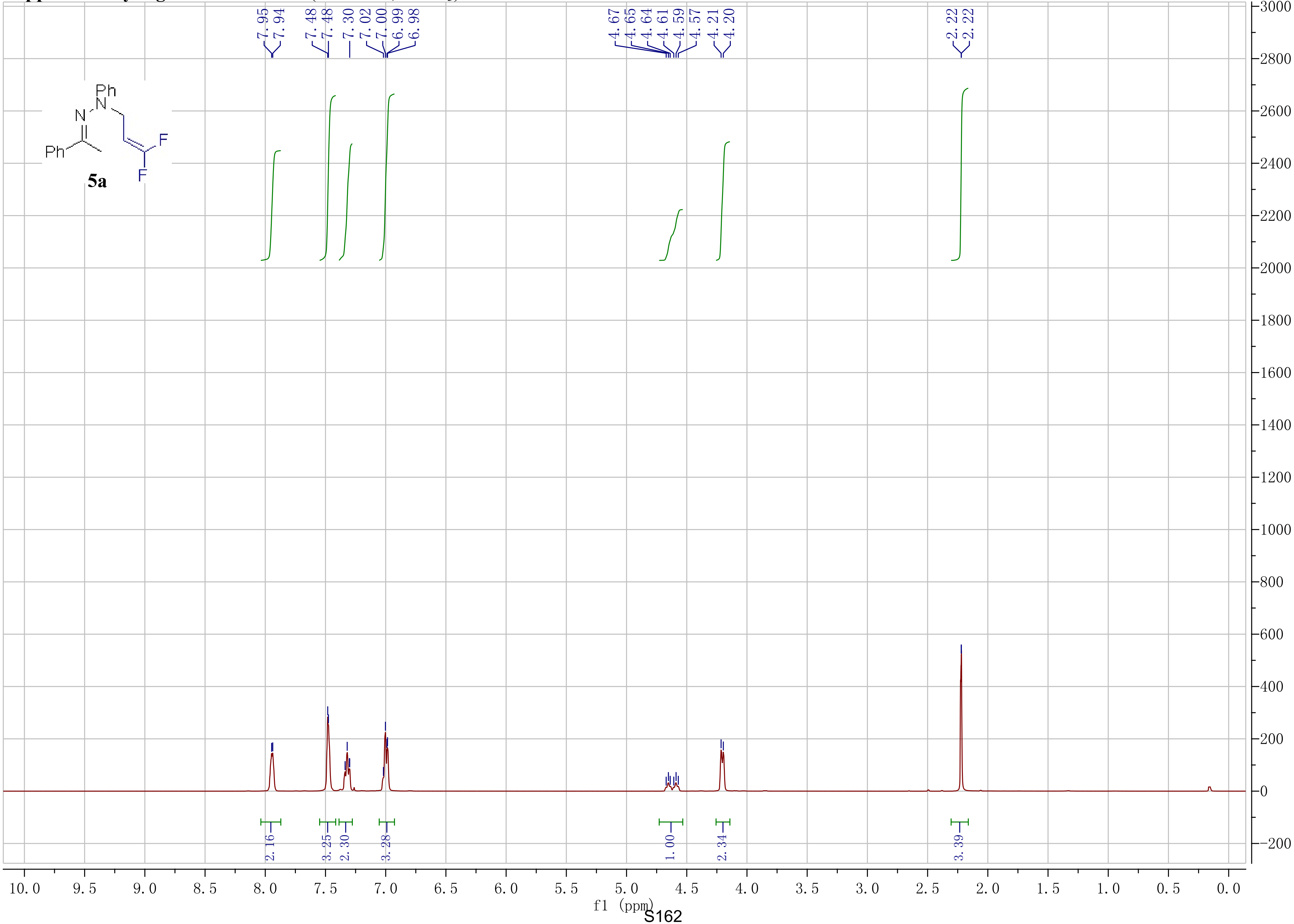

Supplementary Figure 133 ^{13}C NMR (101 MHz, $CDCl_3$) of **5a**

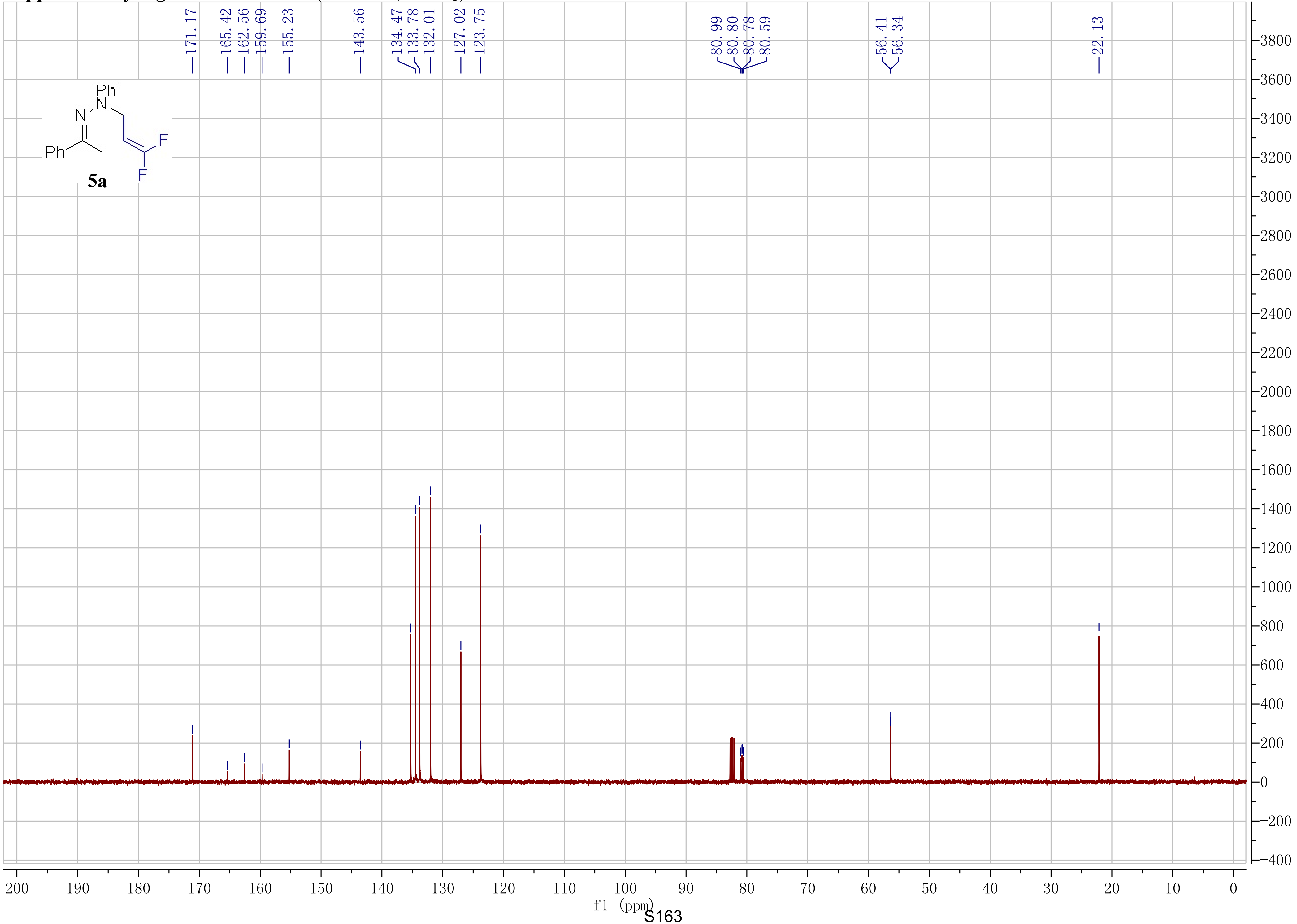

Supplementary Figure 134 ^{19}F NMR (376 MHz, $CDCl_3$) of **5a**

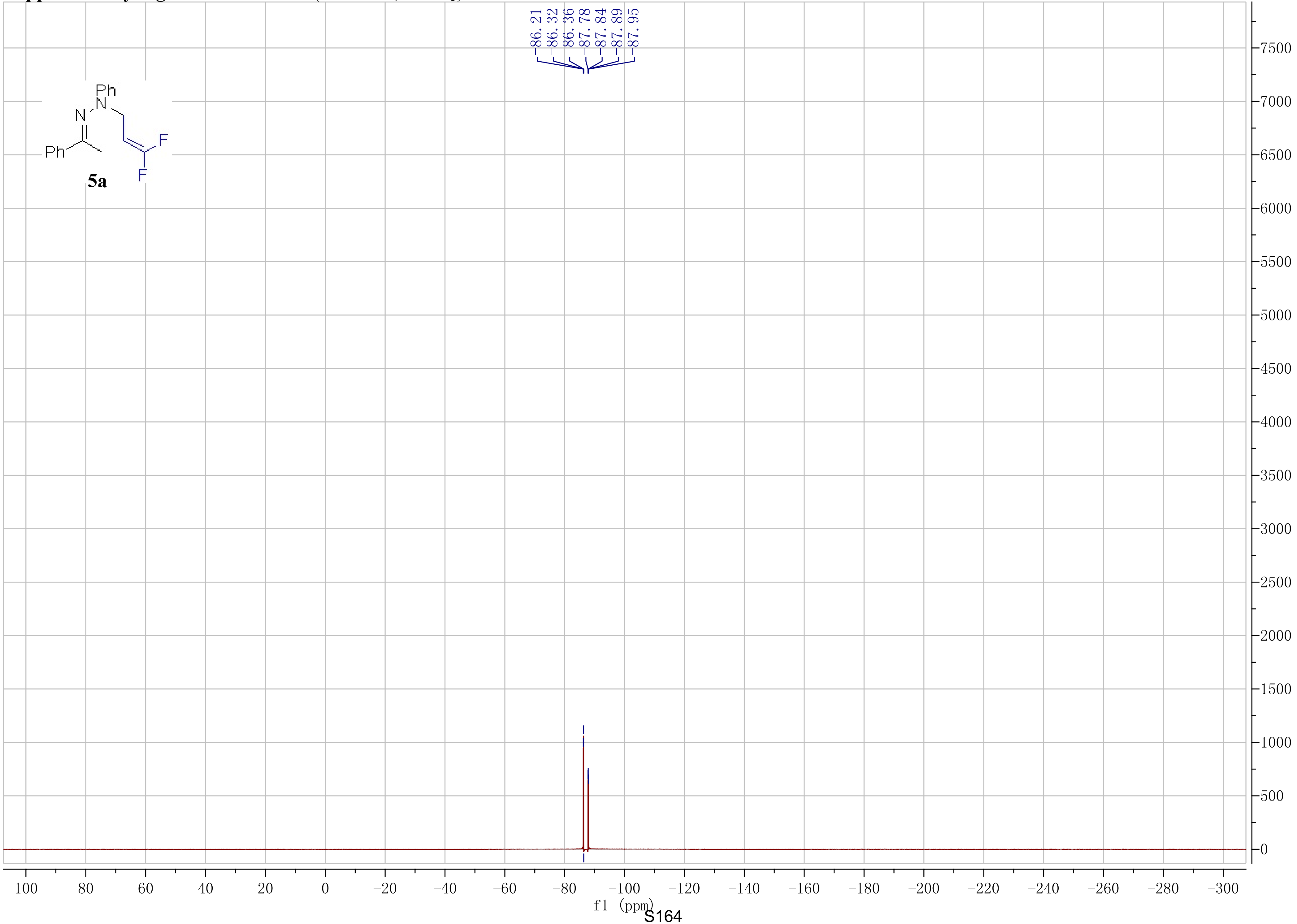

Supplementary Figure 135 ^1H NMR (400 MHz, $CDCl_3$) of **6**

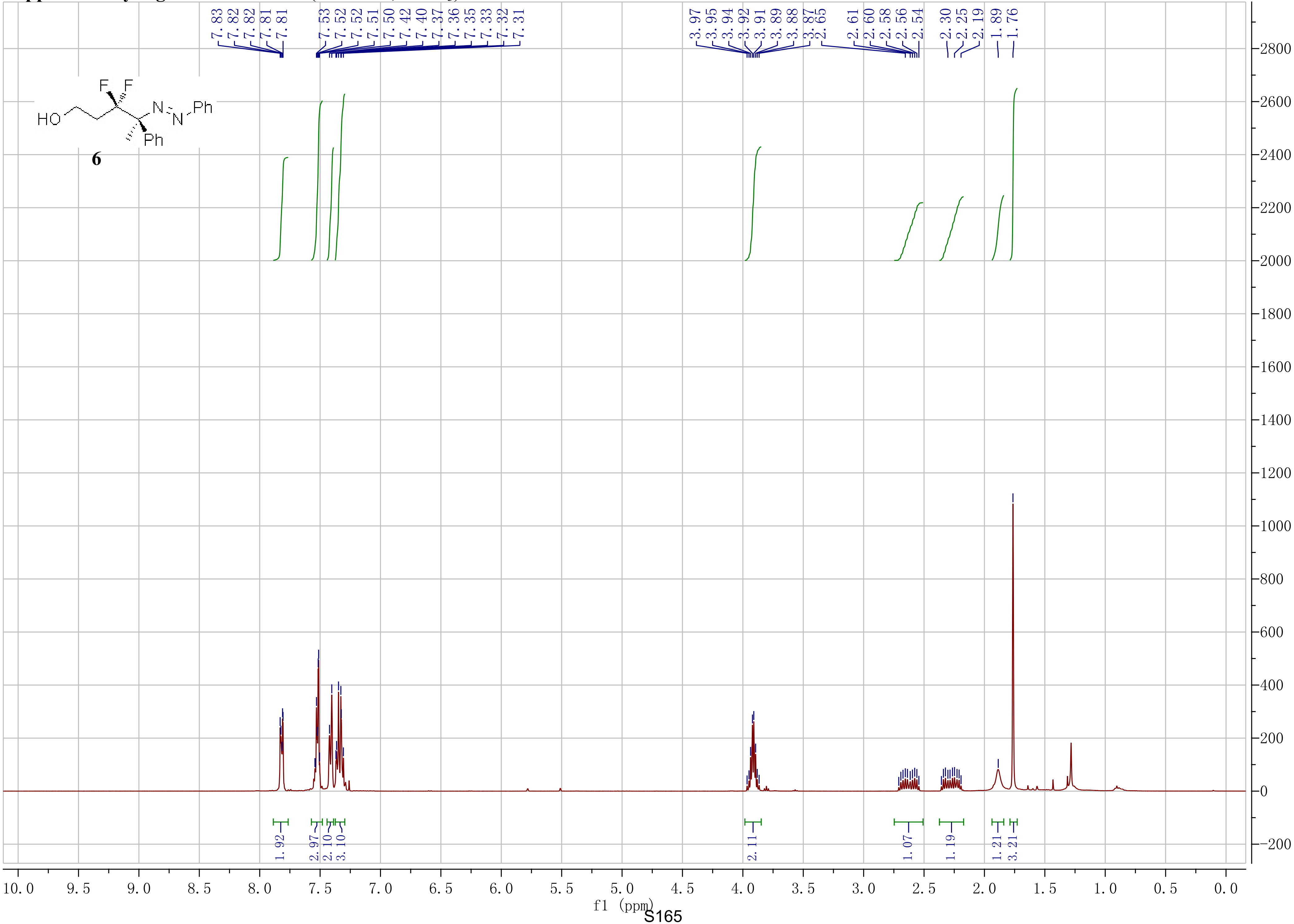

Supplementary Figure 136 ^{13}C NMR (101 MHz, $CDCl_3$) of **6**

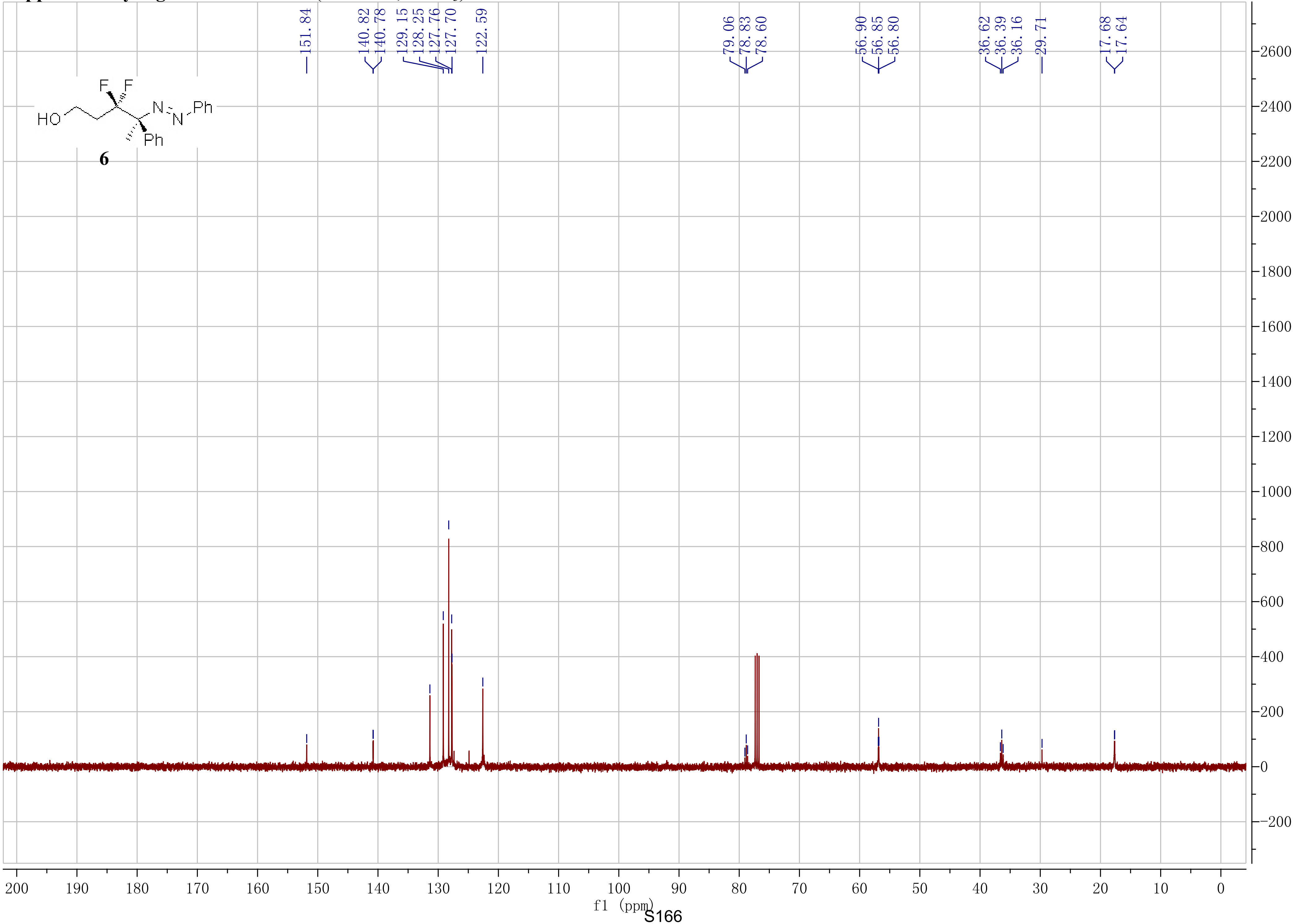

Supplementary Figure 137 ^{19}F NMR (376 MHz, $CDCl_3$) of **6**

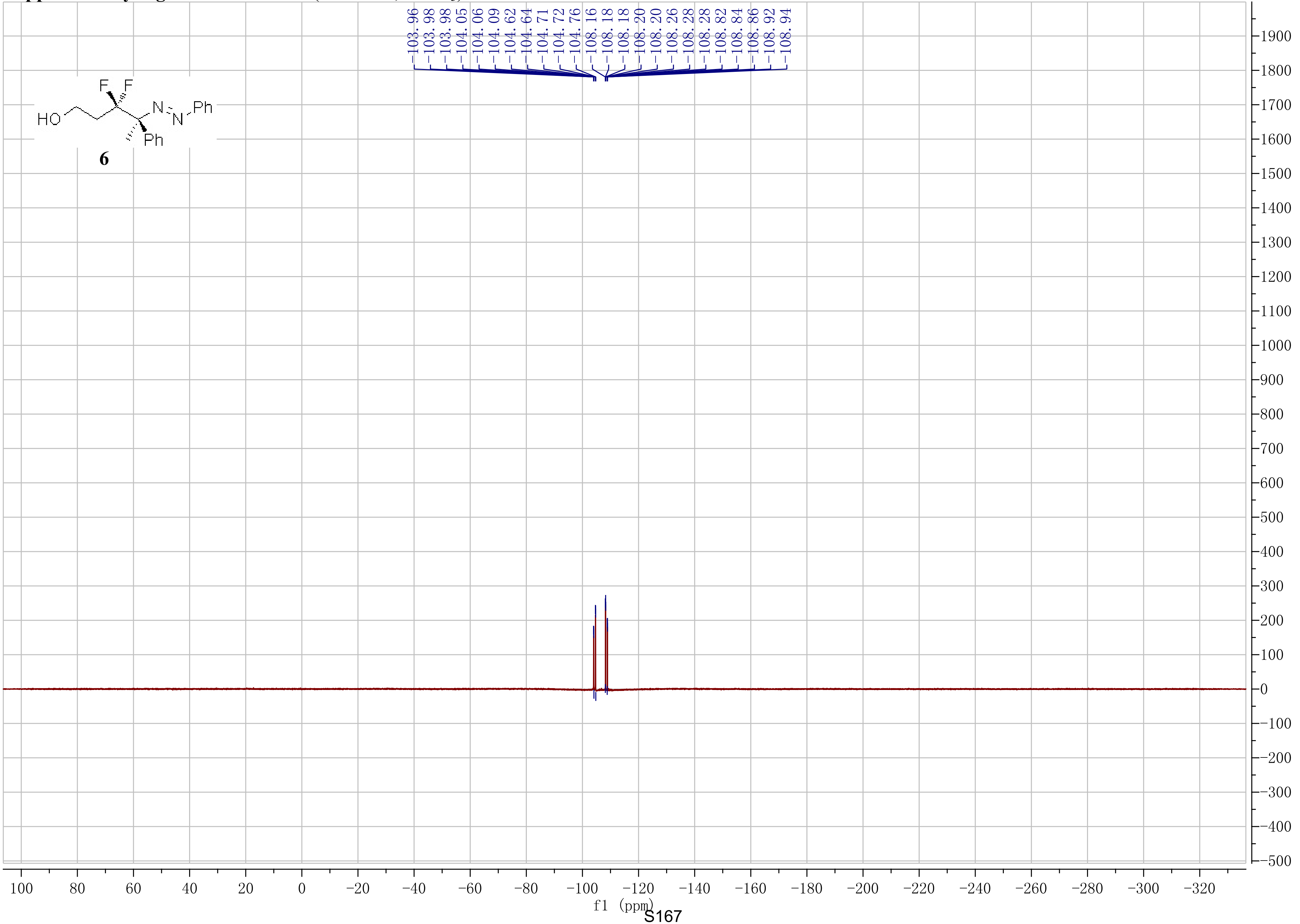

Supplementary Figure 138 HPLC spectra of racemic **6**

SAMPLE INFORMATION

Sample Name:	HS-13-54-2-2+- PC3 55 214 0.7	Acquired By:	System
Sample Type:	Unknow n	Sample Set Name:	20200806
Vial:	1:C,4	Acq. Method Set:	a7w 3
Injection #:	1	Processing Method:	1
Injection Volume:	5.00 ul	Channel Name:	PDA Ch1 214nm@4.8nm
Run Time:	105.0 Minutes	Proc. Chnl. Descr.:	PDA Ch1 214nm@4.8nm
Date Acquired:	8/31/2020 7:02:43 PM CST		
Date Processed:	9/3/2020 9:08:42 AM CST		

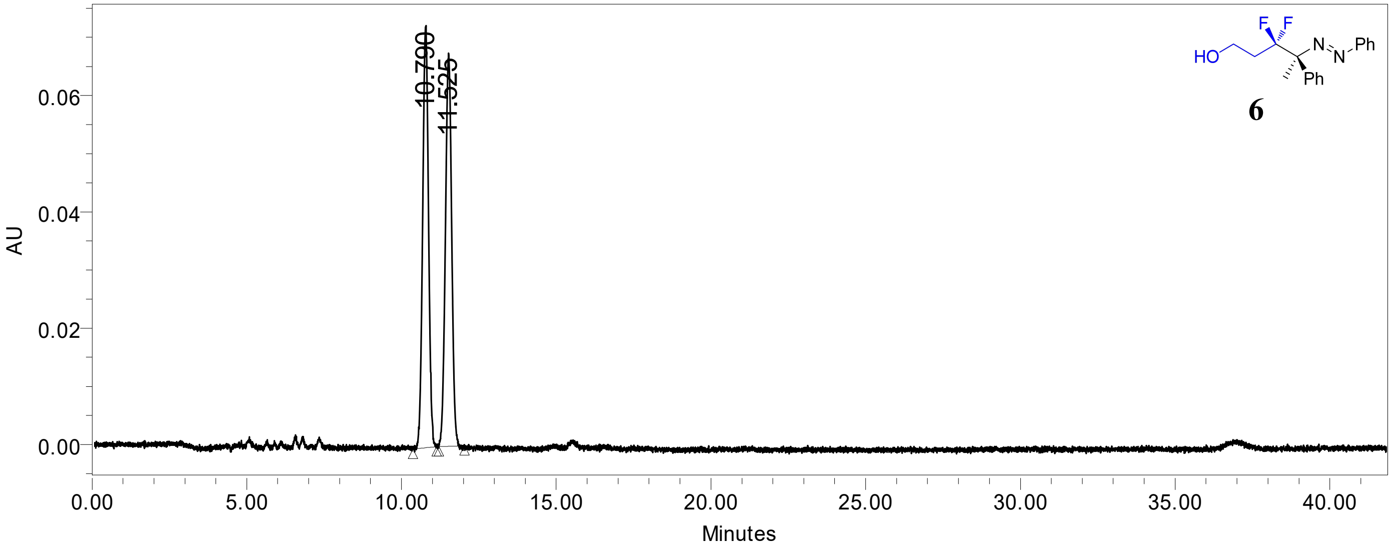

	RT	Area	% Area	Height
1	10.790	962202	50.08	72497
2	11.525	959093	49.92	67595

Supplementary Figure 139 HPLC spectra of (*S*)-**6**

S A M P L E   I N F O R M A T I O N

Sample Name:	HS-13-35-2 PC3 55 214 0.7	Acquired By:	System
Sample Type:	Unknow n	Sample Set Name:	20200806
Vial:	1:C,5	Acq. Method Set:	a7w 3
Injection #:	1	Processing Method:	1
Injection Volume:	5.00 ul	Channel Name:	PDA Ch1 214nm@4.8nm
Run Time:	15.0 Minutes	Proc. Chnl. Descr.:	PDA Ch1 214nm@4.8nm
Date Acquired:	8/31/2020 7:46:15 PM CST		
Date Processed:	9/3/2020 9:09:15 AM CST		

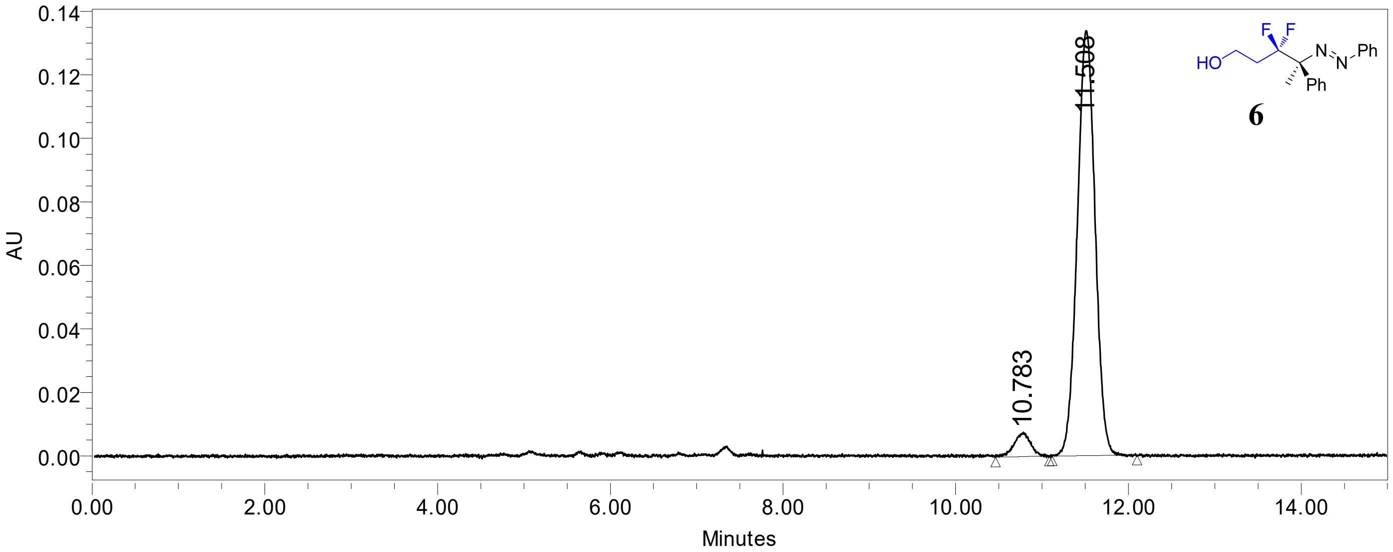

	RT	Area	% Area	Height
1	10.783	101131	5.00	7661
2	11.508	1919608	95.00	133877

Supplementary Figure 140 ^{1}H NMR (400 MHz, $CDCl_3$) of **7**

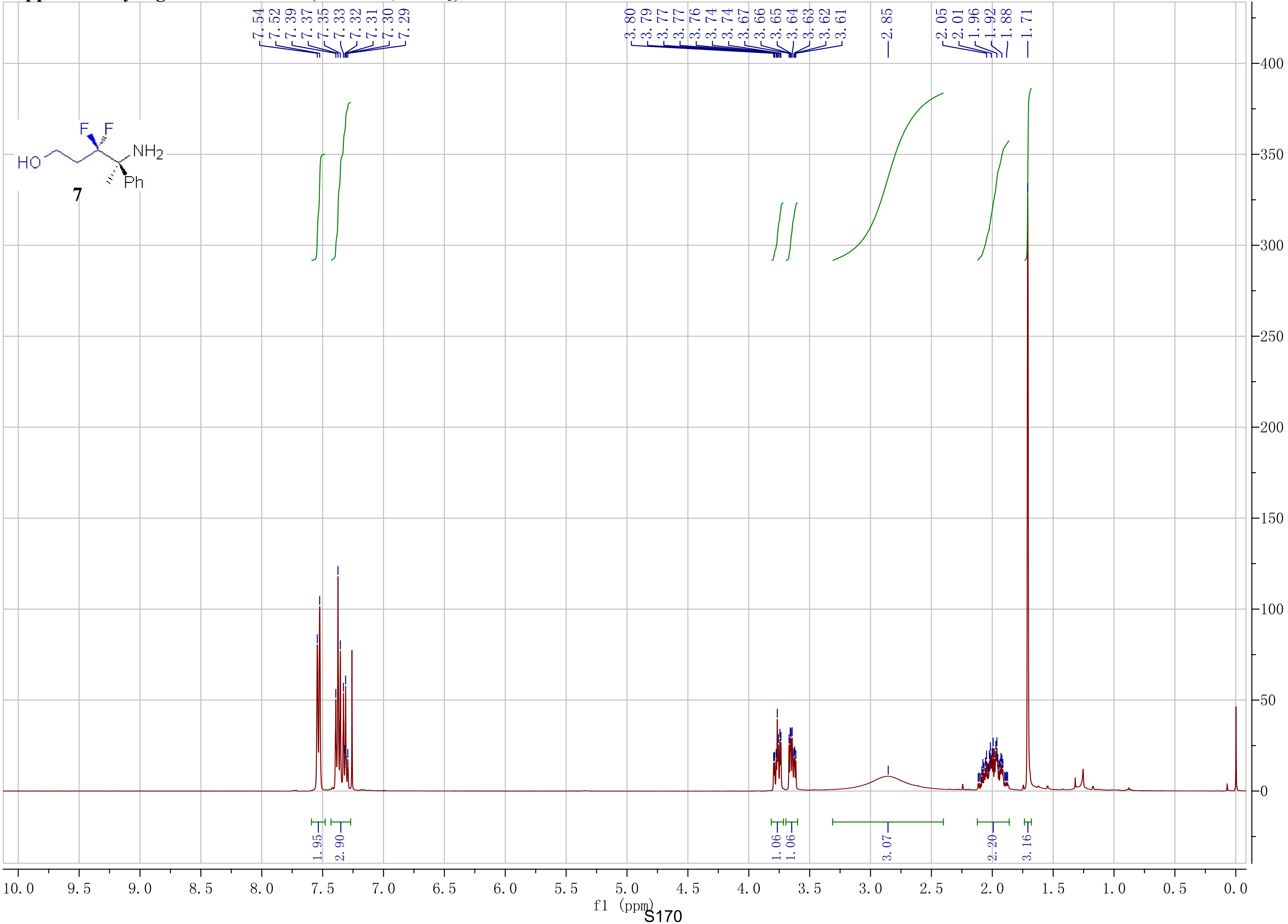

Supplementary Figure 141 ^{13}C NMR (101 MHz, $CDCl_3$) of **7**

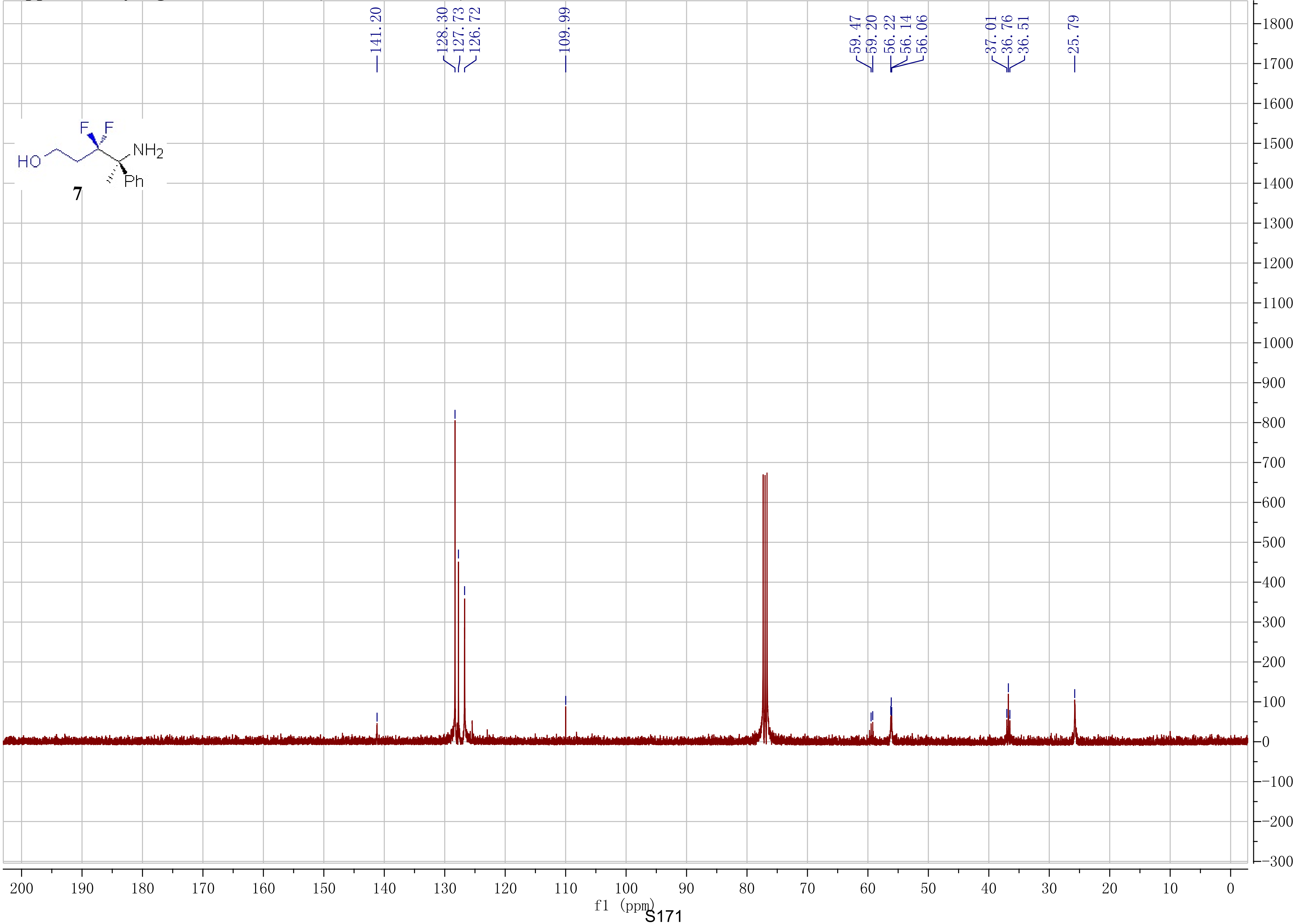

Supplementary Figure 142 ^{19}F NMR (376 MHz, $CDCl_3$) of **7**

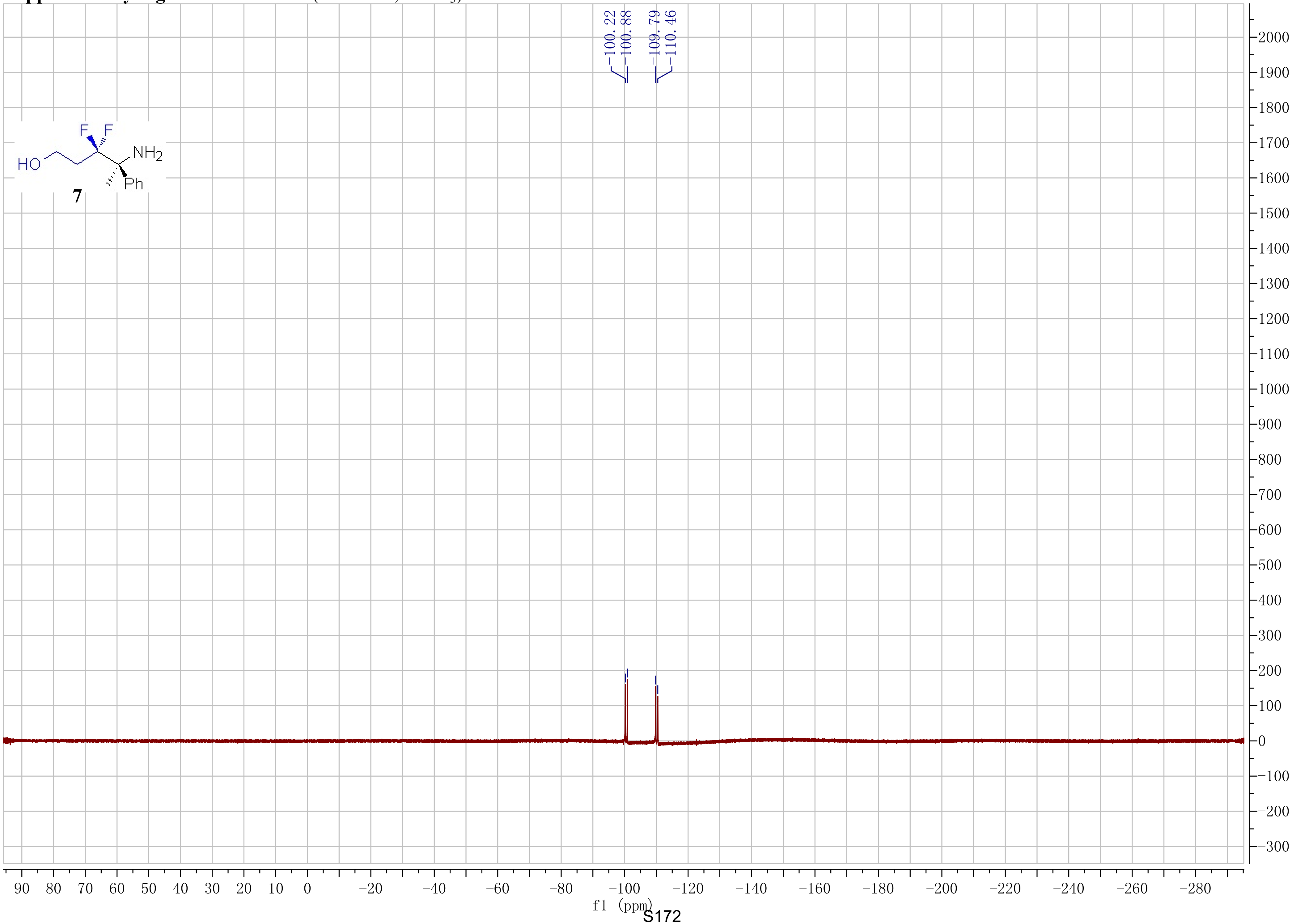

7

Supplementary Figure 143 HPLC spectra of racemic **7**

SAMPLE INFORMATION

Sample Name:	hs-13-55-1-3-RAC	Acquired By:	System
Sample Type:	Unknown	Sample Set Name	
Vial:	1	Acq. Method Set:	2695PDA
Injection #:	1	Processing Method	Default
Injection Volume:	7.00 ul	Channel Name:	2998 Ch3 210nm@1.2nm
Run Time:	35.0 Minutes	Proc. Chnl. Descr.:	2998 Ch3 210nm@1.2nm
Date Acquired:	2020/9/30 9:20:11 CST		
Date Processed:	2020/9/30 9:55:09 CST		

Auto-Scaled Chromatogram

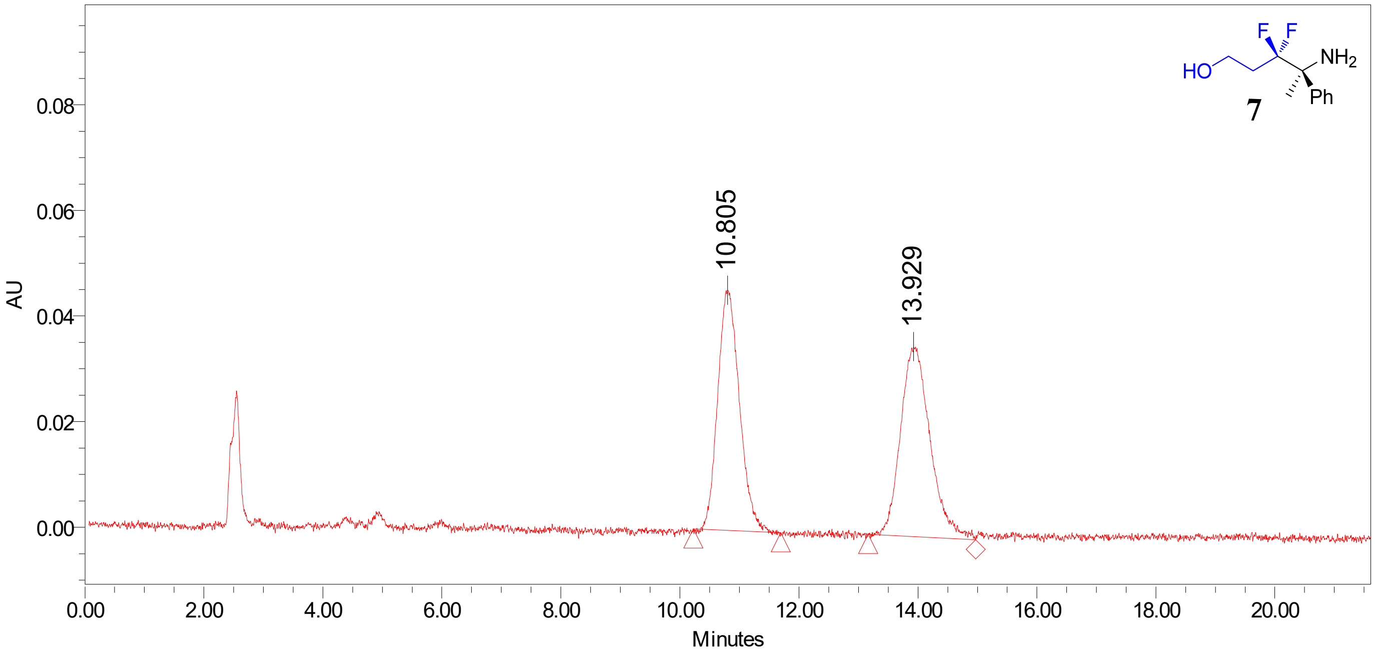

Peak Results

	hs-13-55-1-3-RAC	1	10.805	10.805	88.200	45492	1158986	48.21
	hs-13-55-1-3-RAC	1	13.929	13.929	108.100	35984	1244901	51.79

Reported by User: System
Report Method: Default Individual Report
Report Method ID: 25523
Page: 1 of 1

Project Name: installation
Date Printed:
2020/9/30
13:02:33 PRC

Supplementary Figure 144 HPLC spectra of (*S*)-**7**

SAMPLE INFORMATION

Sample Name:	hs-13-55-3-2	Acquired By:	System
Sample Type:	Unknown	Sample Set Name	
Vial:	2	Acq. Method Set:	2695PDA
Injection #:	2	Processing Methoc	Default
Injection Volume:	7.00 ul	Channel Name:	2998 Ch3 210nm@1.2nm
Run Time:	35.0 Minutes	Proc. Chnl. Descr.:	2998 Ch3 210nm@1.2nm
Date Acquired:	2020/9/30 8:59:45 CST		
Date Processed:	2020/9/30 9:55:30 CST		

Auto-Scaled Chromatogram

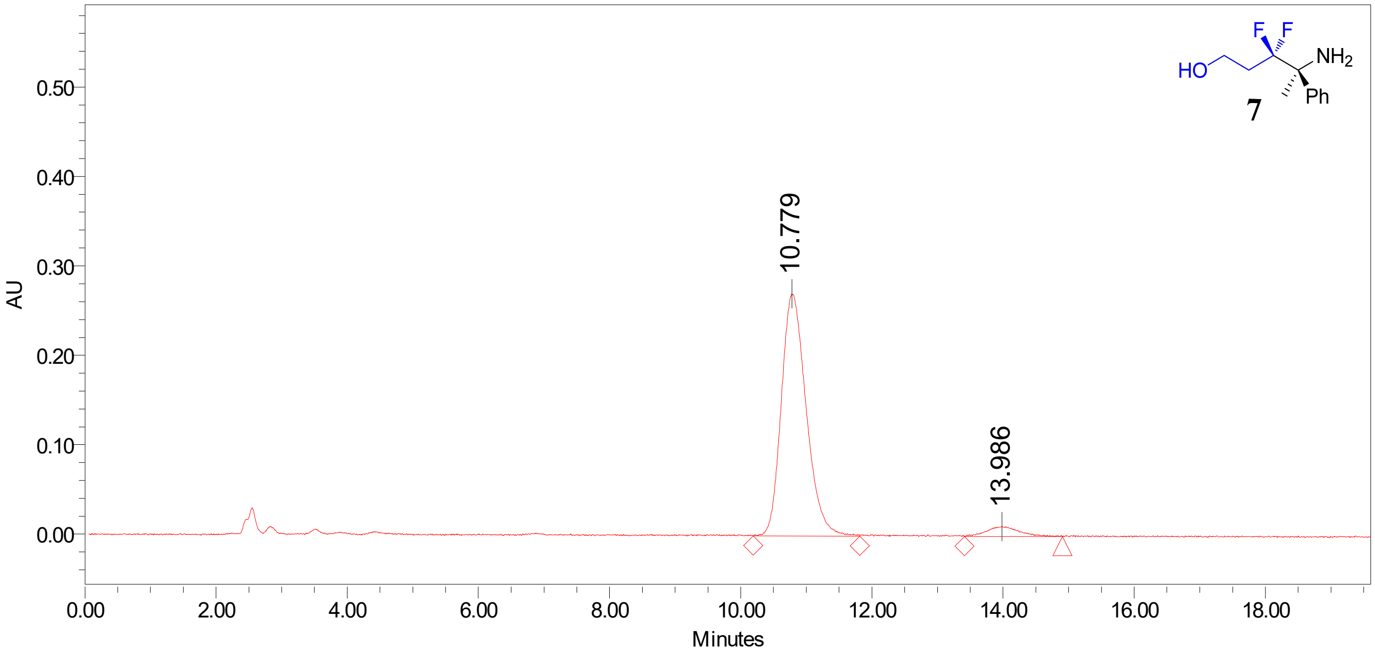

Peak Results

[redacted]

hs-13-55-3-2	2	10.779	10.779	97.600	271212	7152766	94.87
hs-13-55-3-2	2	13.986	13.986	89.400	11081	386831	5.13

Reported by User: System
Report Method: Default Individual Report
Report Method ID: 25523
Page: 1 of 1

Project Name: installation
Date Printed:
2020/9/30
13:02:52 PRC

Supplementary Figure 145 ^{1}H NMR (400 MHz, $CDCl_3$) of **8**

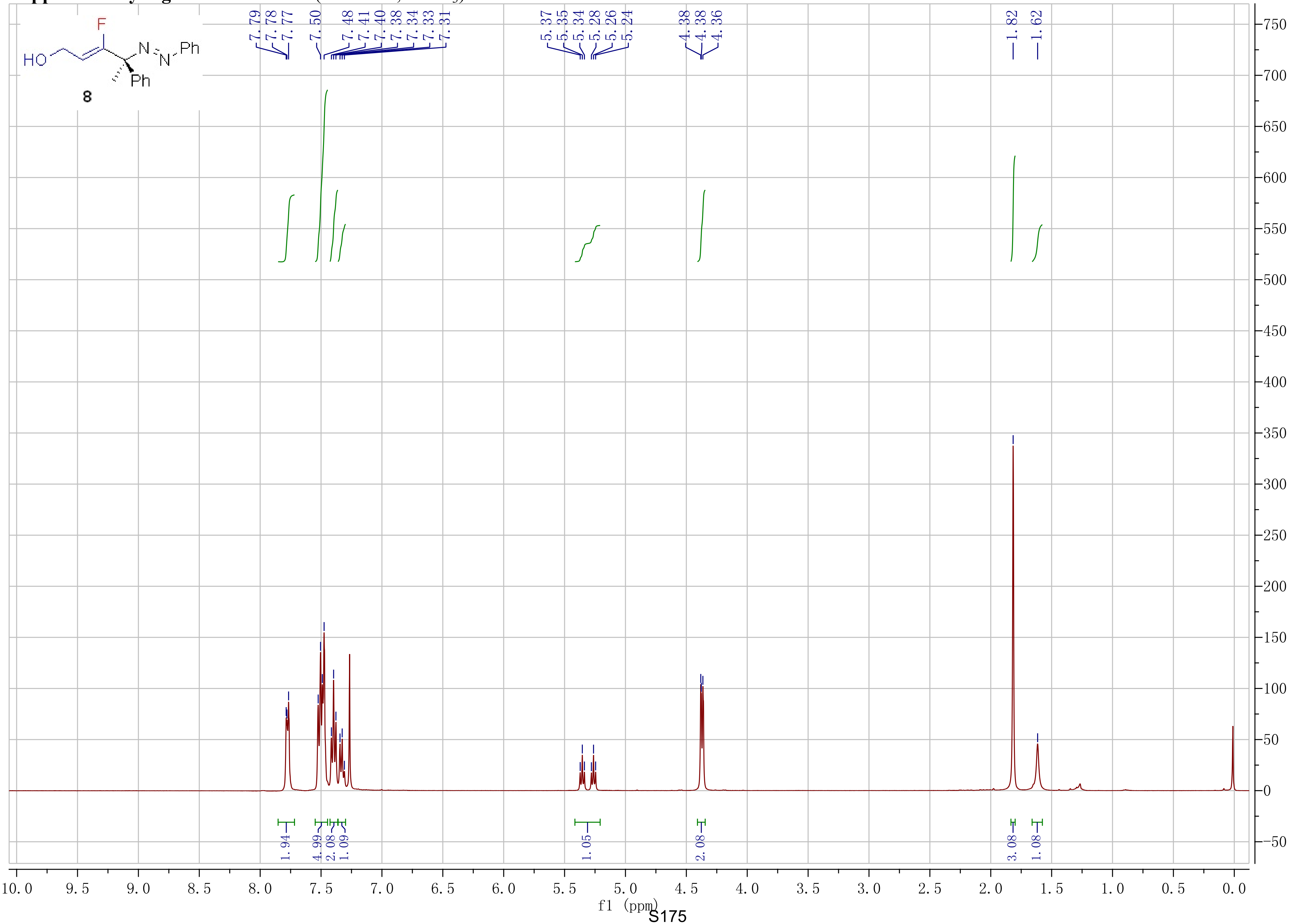

Supplementary Figure 146 ^{13}C NMR (101 MHz, $CDCl_3$) of **8**

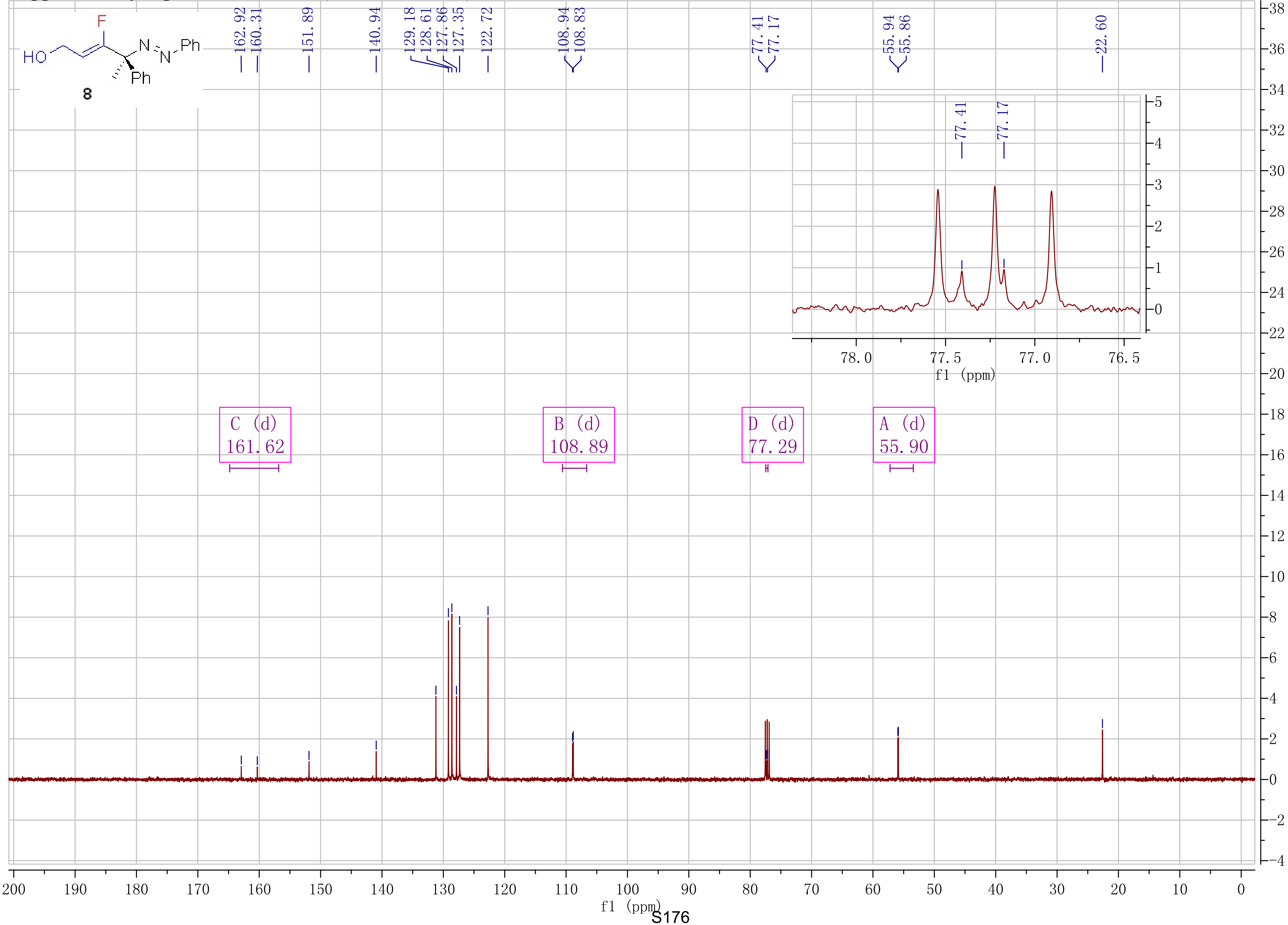

Supplementary Figure 147 ^{19}F NMR (376 MHz, $CDCl_3$) of **8**

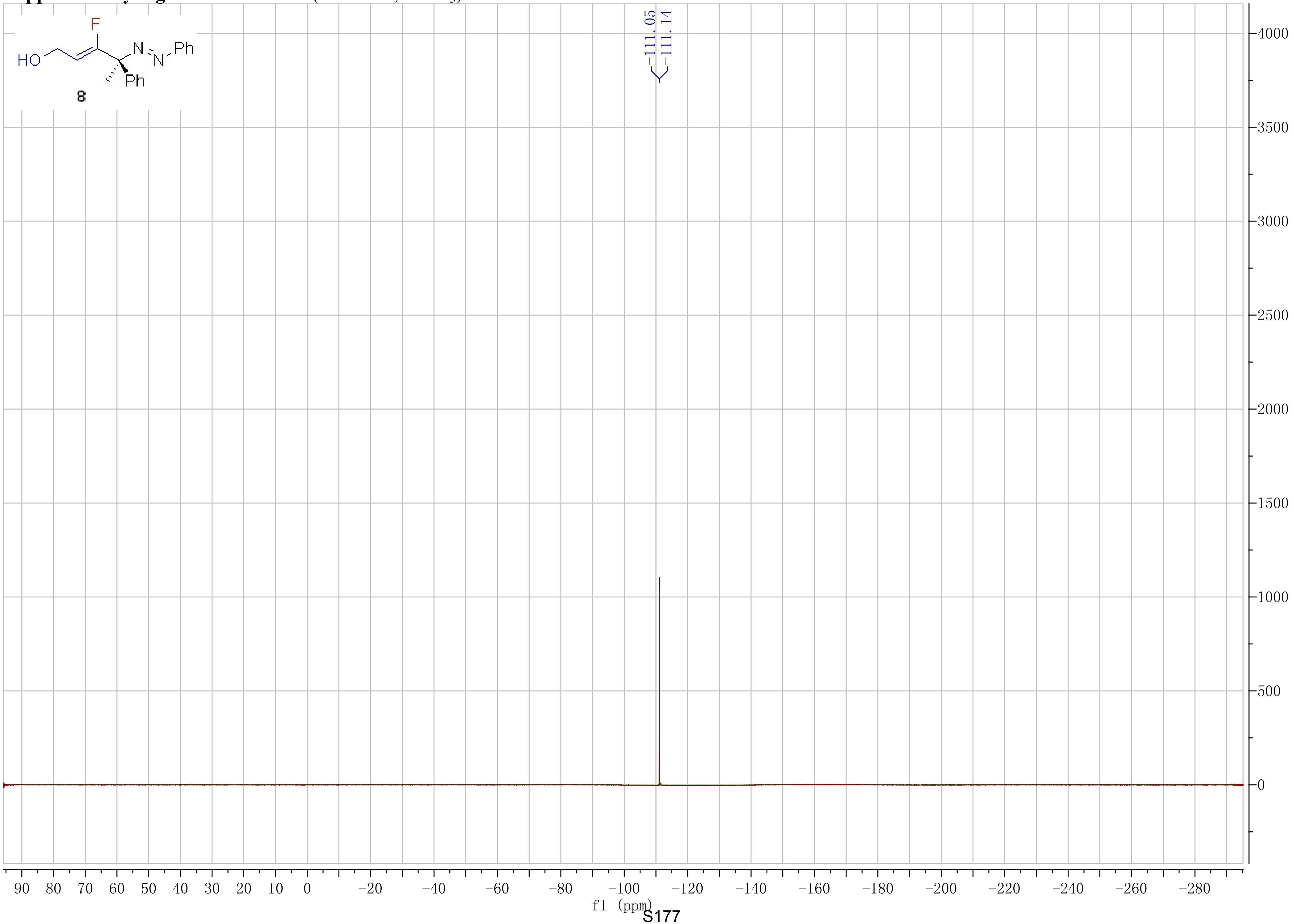

Supplementary Figure 148 HPLC spectra of racemic **8**

S A M P L E   I N F O R M A T I O N

Sample Name:	hs-13-102-2-rac-ig-9-1	Acquired By:	System
Sample Type:	Unknown	Sample Set Name	
Vial:	2:E,2	Acq. Method Set:	upc_pda_2019m
Injection #:	2	Processing Method	Default
Injection Volume:	2.00 ul	Channel Name:	PDA Ch1 214nm@4.8nm
Run Time:	30.0 Minutes	Proc. Chnl. Descr.:	PDA Ch1 214nm@4.8nm
Date Acquired:	1/13/2021 10:11:49 AM CST		
Date Processed:	1/13/2021 3:53:54 PM CST		

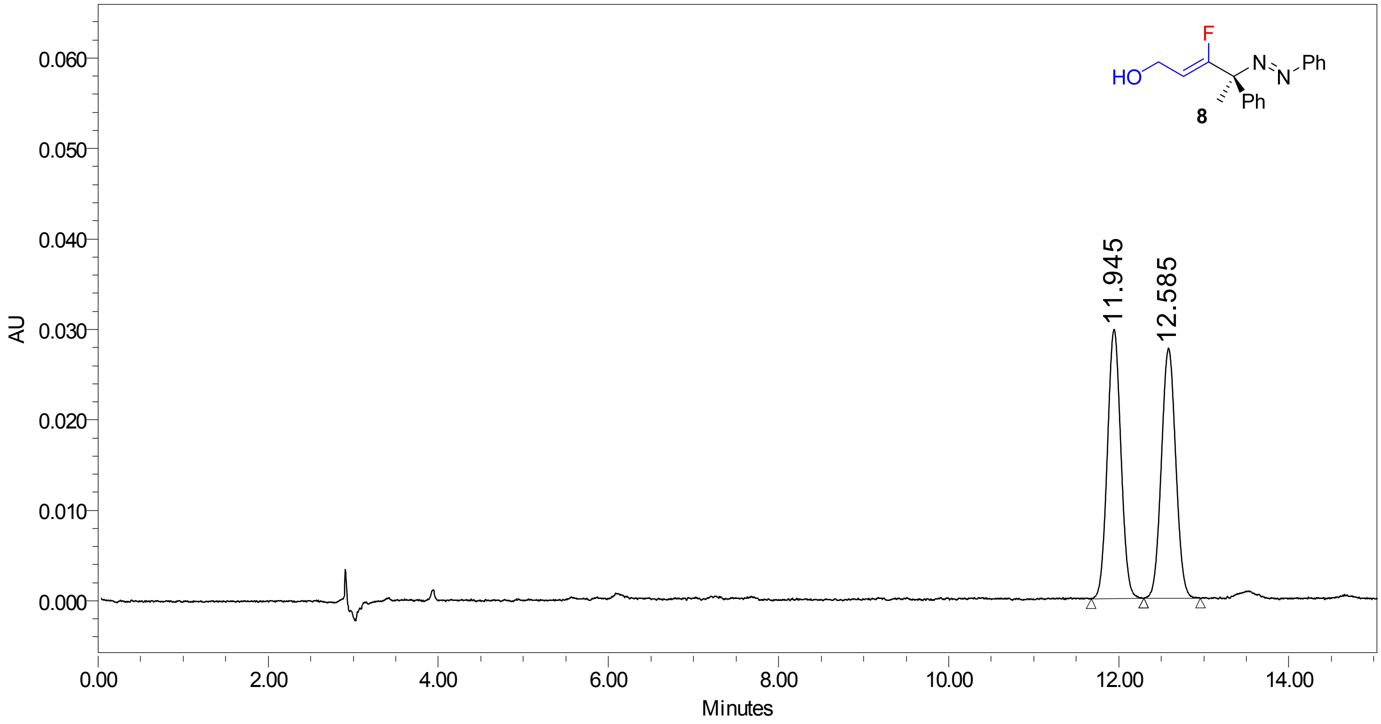

	RT	Peak Type	Height	Width (sec)	Area	% Area
1	11.945	Unknown	29750	37.000	329125	50.23
2	12.585	Unknown	27666	40.000	326124	49.77

Reported by User: System
Report Method: Default Individual Report
Report Method II 37628
Page: 1 of 1

Project Name: TEST
Date Printed:
1/13/2021
3:54:29 PM PRC

Supplementary Figure 149 HPLC spectra of (*S*)-**8**

SAMPLE INFORMATION

Sample Name:	hs-13-102-3	Acquired By:	System
Sample Type:	Unknown	Sample Set Name	
Vial:	2:E,3	Acq. Method Set:	upc_pda_2019m
Injection #:	2	Processing Method	Default
Injection Volume:	4.00 ul	Channel Name:	PDA Ch1 214nm@4.8nm
Run Time:	30.0 Minutes	Proc. Chnl. Descr.:	PDA Ch1 214nm@4.8nm
Date Acquired:	1/13/2021 11:38:20 AM CST		
Date Processed:	1/13/2021 3:54:16 PM CST		

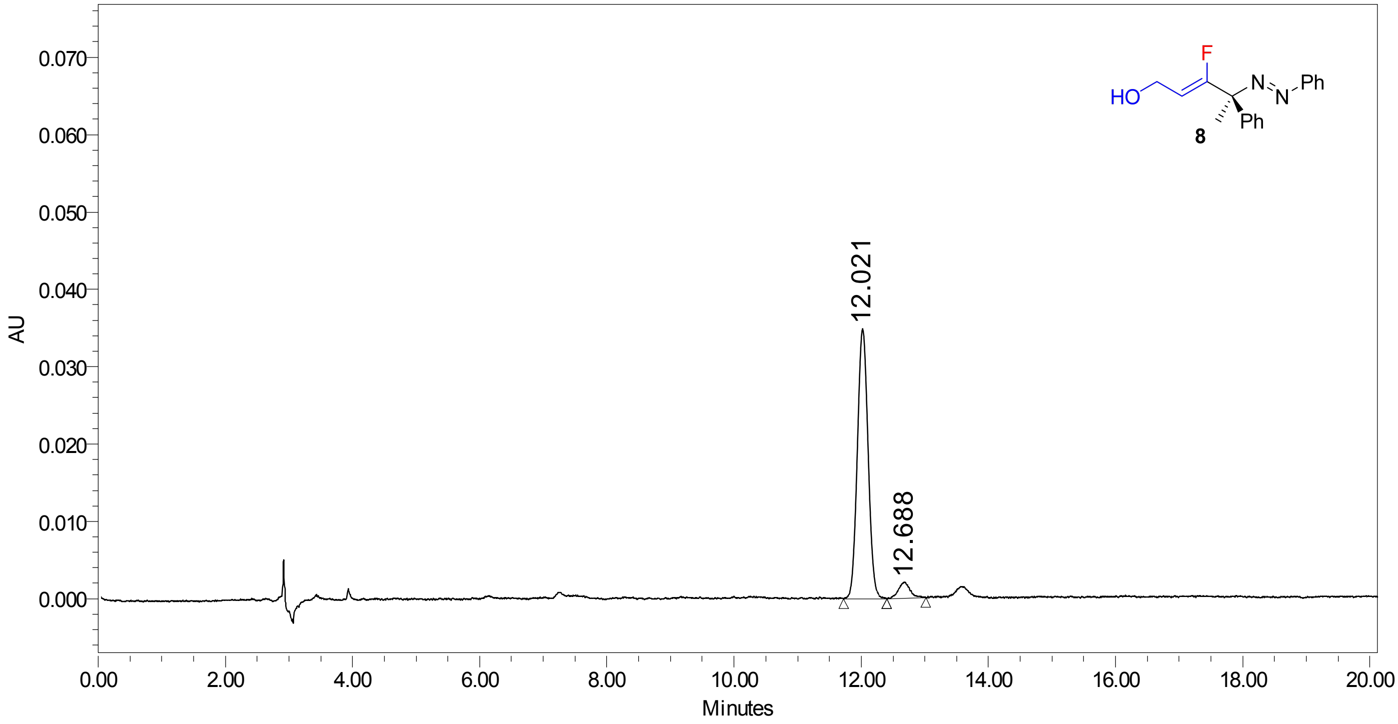

	RT	Peak Type	Height	Width (sec)	Area	% Area
1	12.021	Unknown	34929	40.700	409657	93.90
2	12.688	Unknown	2071	36.600	26591	6.10

Reported by User: System
Report Method: Default Individual Report
Report Method II 37628
Page: 1 of 1

Project Name: TEST
Date Printed:
1/13/2021
3:54:46 PM PRC

Supplementary Figure 150 ^{1}H NMR (400 MHz, $CDCl_3$) of **9**

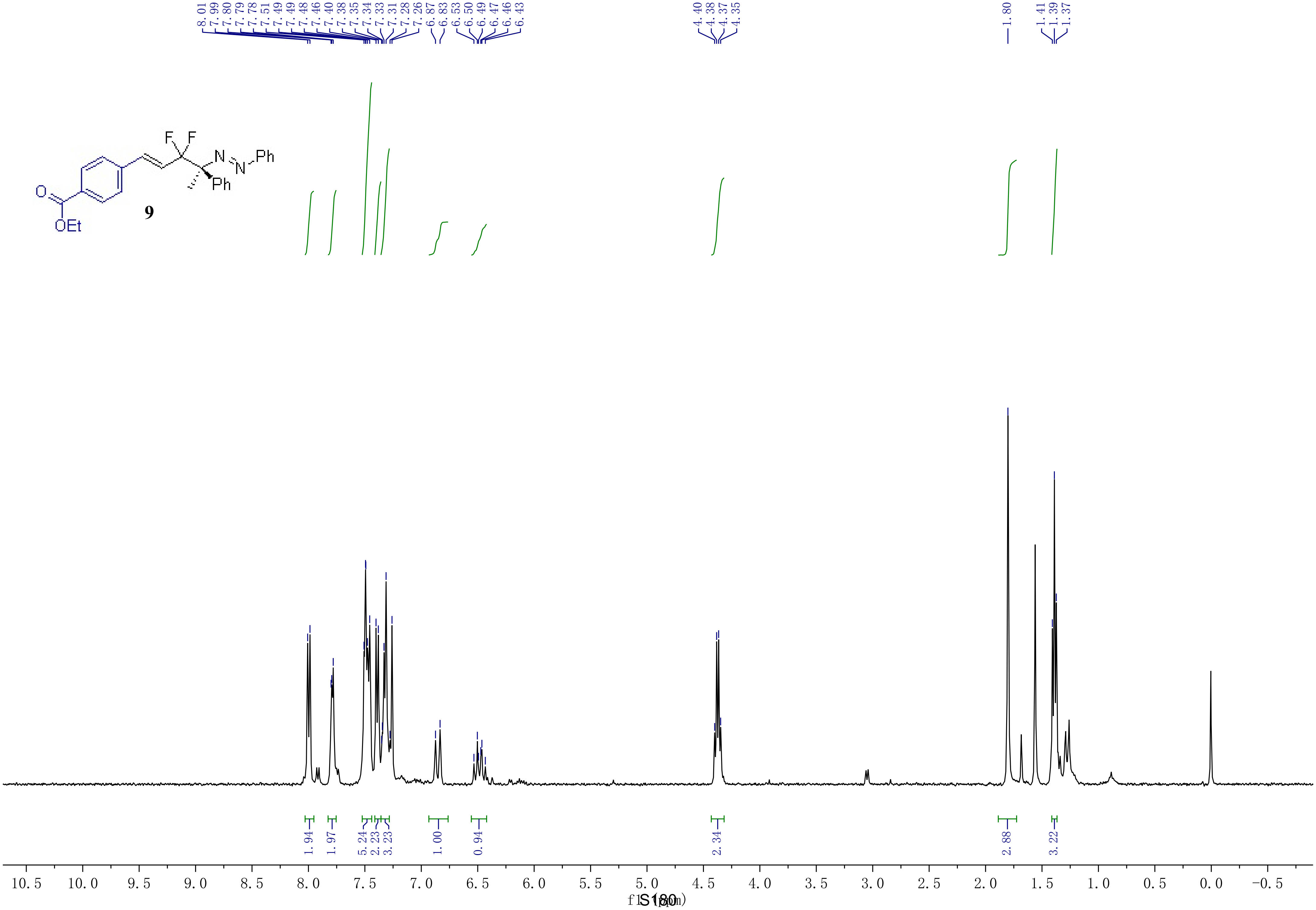

Supplementary Figure 151 ^{13}C NMR (101 MHz, $CDCl_3$) of **9**

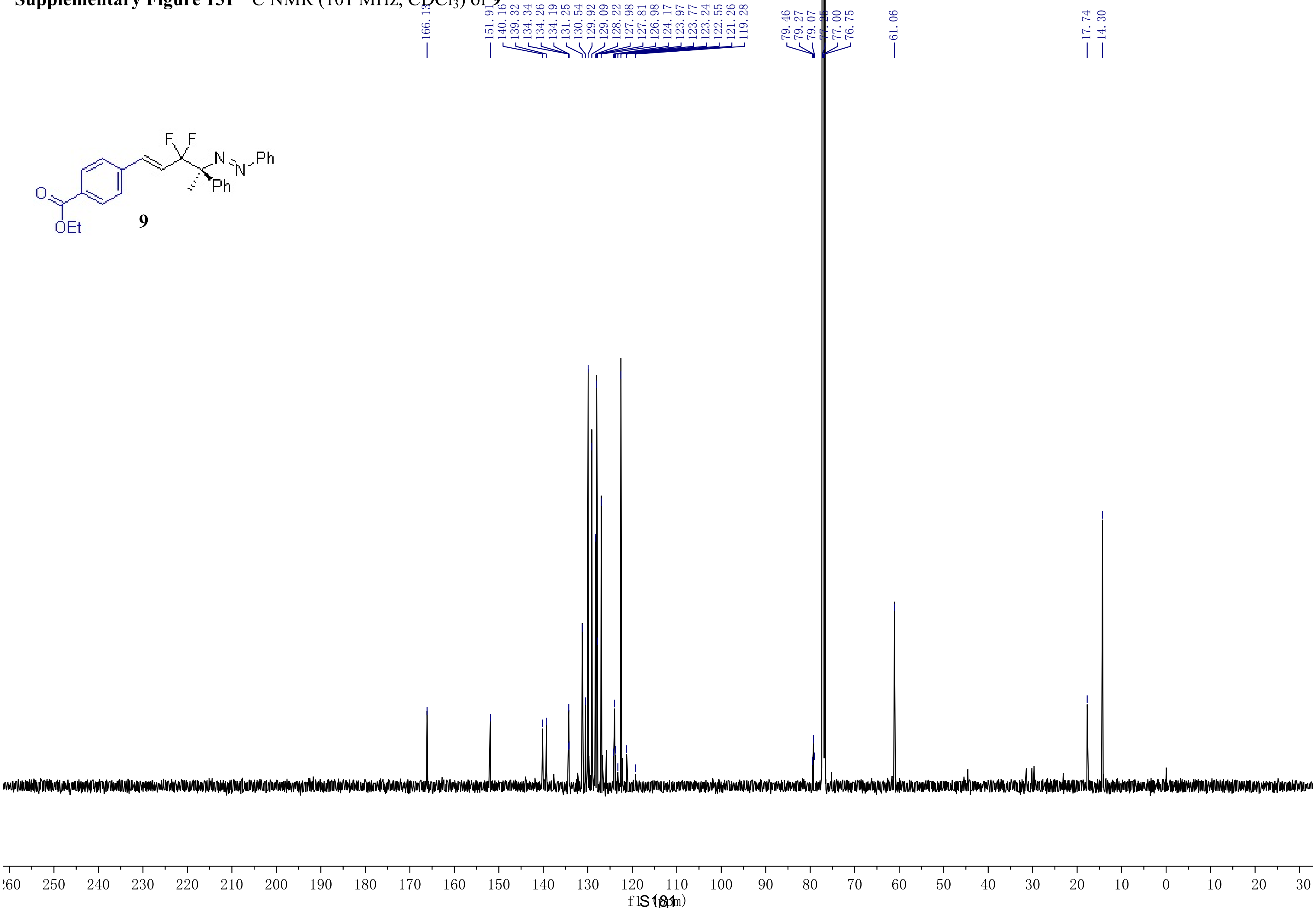

Supplementary Figure 152 ^{19}F NMR (376 MHz, $CDCl_3$) of **9**

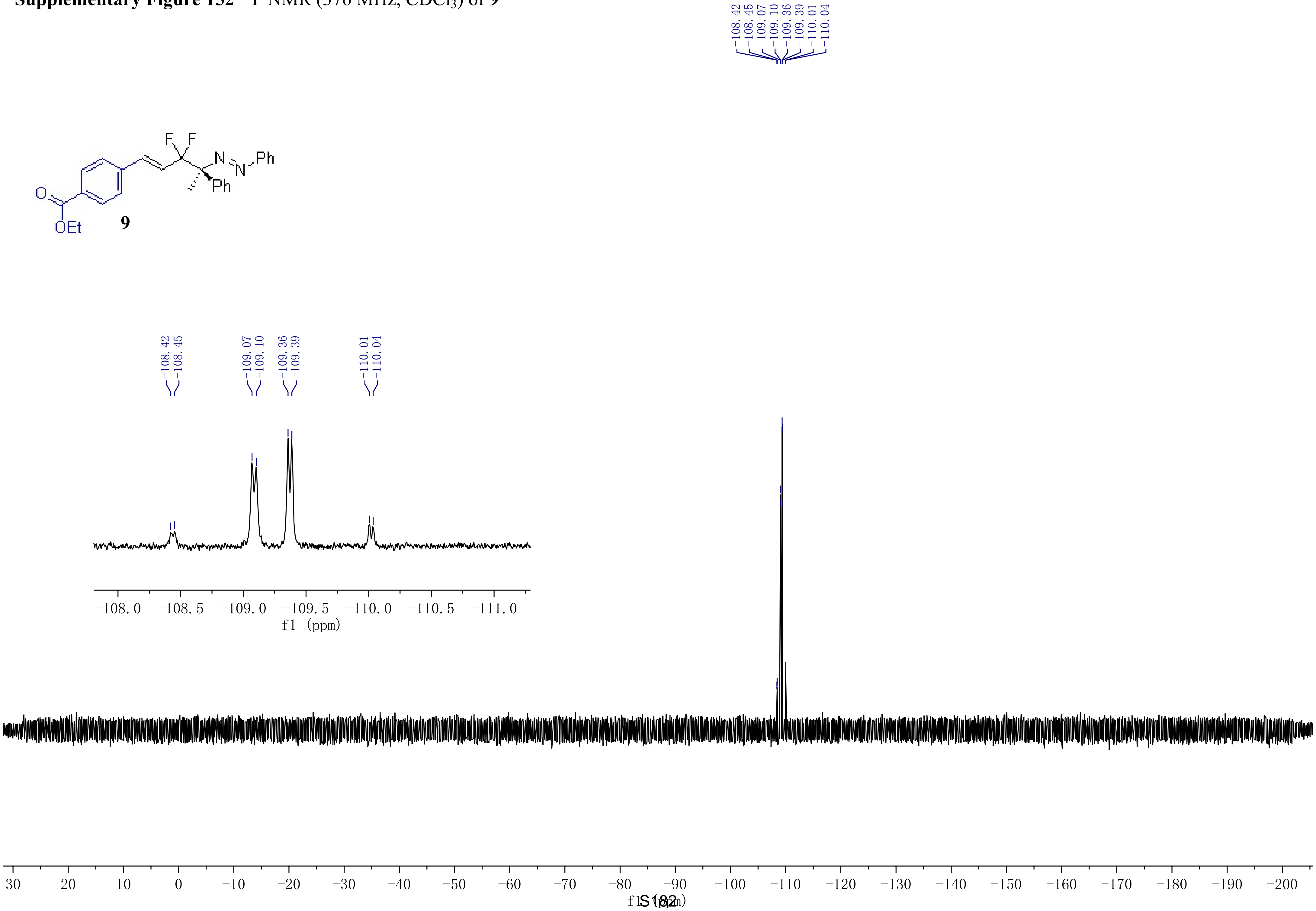

Supplementary Figure 153 HPLC spectra of racemic **9**

Operator:zhang Timebase:U-3000 Sequence:TFF-NO.1

Page 1-1
2021/6/29 10:28

509 TFF-hou-2-racmic-heck-A30B70-pc3-1

Sample Name:	**TFF-hou-2-racmic-heck-A30B70-pc3-1**	*Injection Volume:*	**10.0**
Vial Number:	**BE2**	*Channel:*	**UV_VIS_1**
Sample Type:	**standard**	*Wavelength:*	**214**
Control Program:	**AL-1**	*Bandwidth:*	**n.a.**
Quantif. Method:	**xc20130910**	*Dilution Factor:*	**1.0000**
Recording Time:	**2021/6/25 12:12**	*Sample Weight:*	**1.0000**
Run Time (min):	**30.00**	*Sample Amount:*	**1.0000**

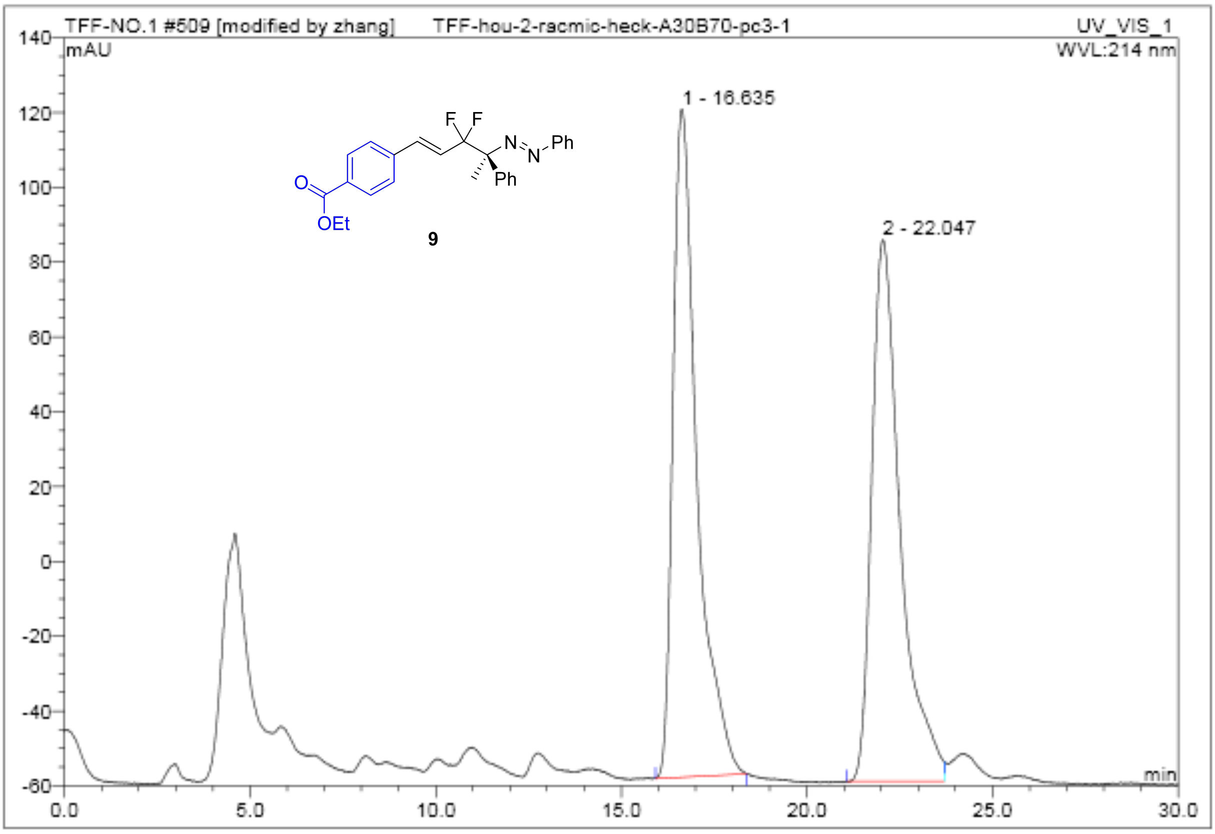

No.	Ret.Time min	Peak Name	Height mAU	Area mAU*min	Rel.Area %	Amount %	Type
1	16.64	n.a.	178.591	133.784	50.89	n.a.	BMB*
2	22.05	n.a.	144.786	129.115	49.11	n.a.	BM *
**Total:**			323.377	262.899	100.00	0.000	

DEFAULT/Integration

Chromeleon (c) Dionex 1996-2006
Version 6.80 SR10 Build 2818 (166959)

Supplementary Figure 154 HPLC spectra of (*S*)-**9**

Operator:zhang Timebase:U-3000 Sequence:TFF-NO.1

Page 1-1
2021/6/29 10:28

512 TFF-hou-2-chiral-heck-A30B70-pc3-2

Sample Name:	**TFF-hou-2-chiral-heck-A30B70-pc3-2**	*Injection Volume:*	**3.0**
Vial Number:	**BE3**	*Channel:*	**UV_VIS_1**
Sample Type:	**standard**	*Wavelength:*	**214**
Control Program:	**AL-1**	*Bandwidth:*	**n.a.**
Quantif. Method:	**xc20130910**	*Dilution Factor:*	**1.0000**
Recording Time:	**2021/6/25 15:14**	*Sample Weight:*	**1.0000**
Run Time (min):	**27.03**	*Sample Amount:*	**1.0000**

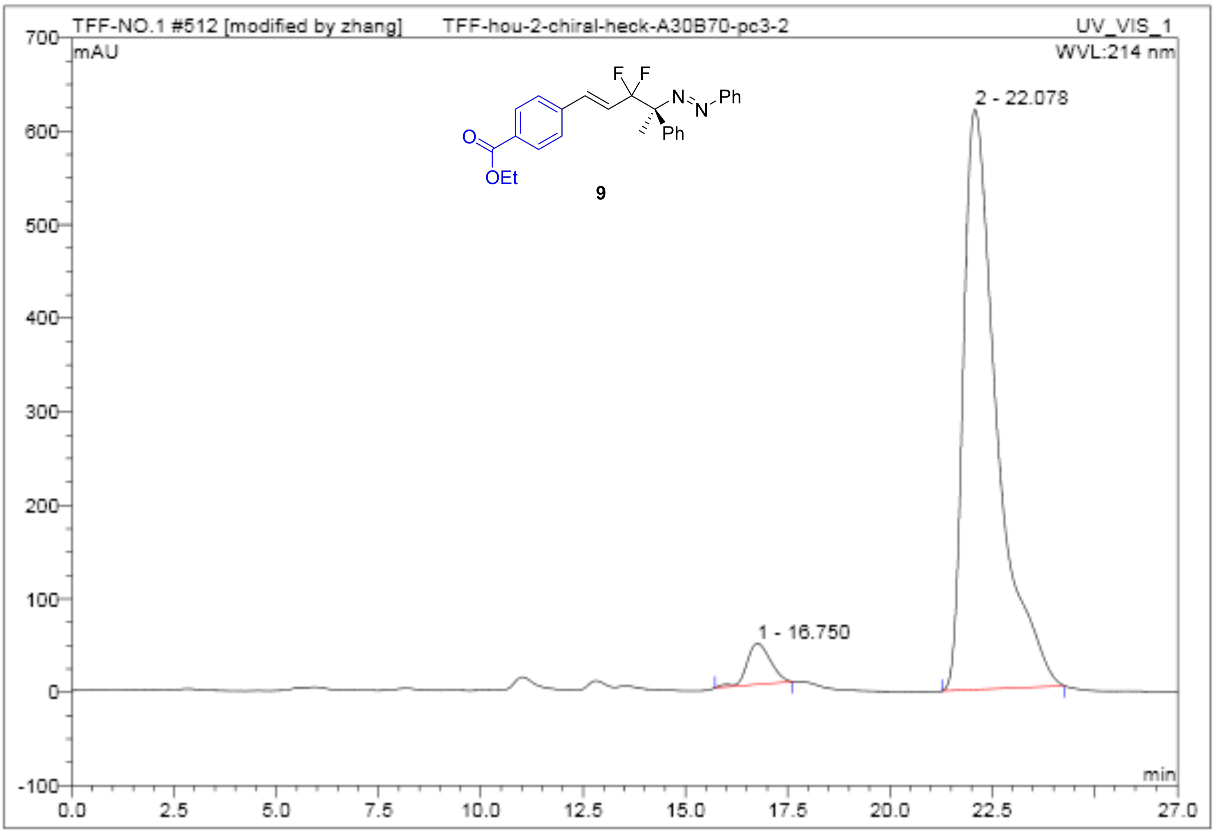

No.	Ret.Time min	Peak Name	Height mAU	Area mAU*min	Rel.Area %	Amount %	Type
1	16.75	n.a.	44.022	29.257	4.75	n.a.	BMB*
2	22.08	n.a.	620.688	586.123	95.25	n.a.	BMB
**Total:**			664.710	615.380	100.00	0.000	

DEFAULT/Integration

Chromeleon (c) Dionex 1996-2006
Version 6.80 SR10 Build 2818 (166959)

Supplementary Figure 155 ^{1}H NMR (400 MHz, $CDCl_3$) of **L3**

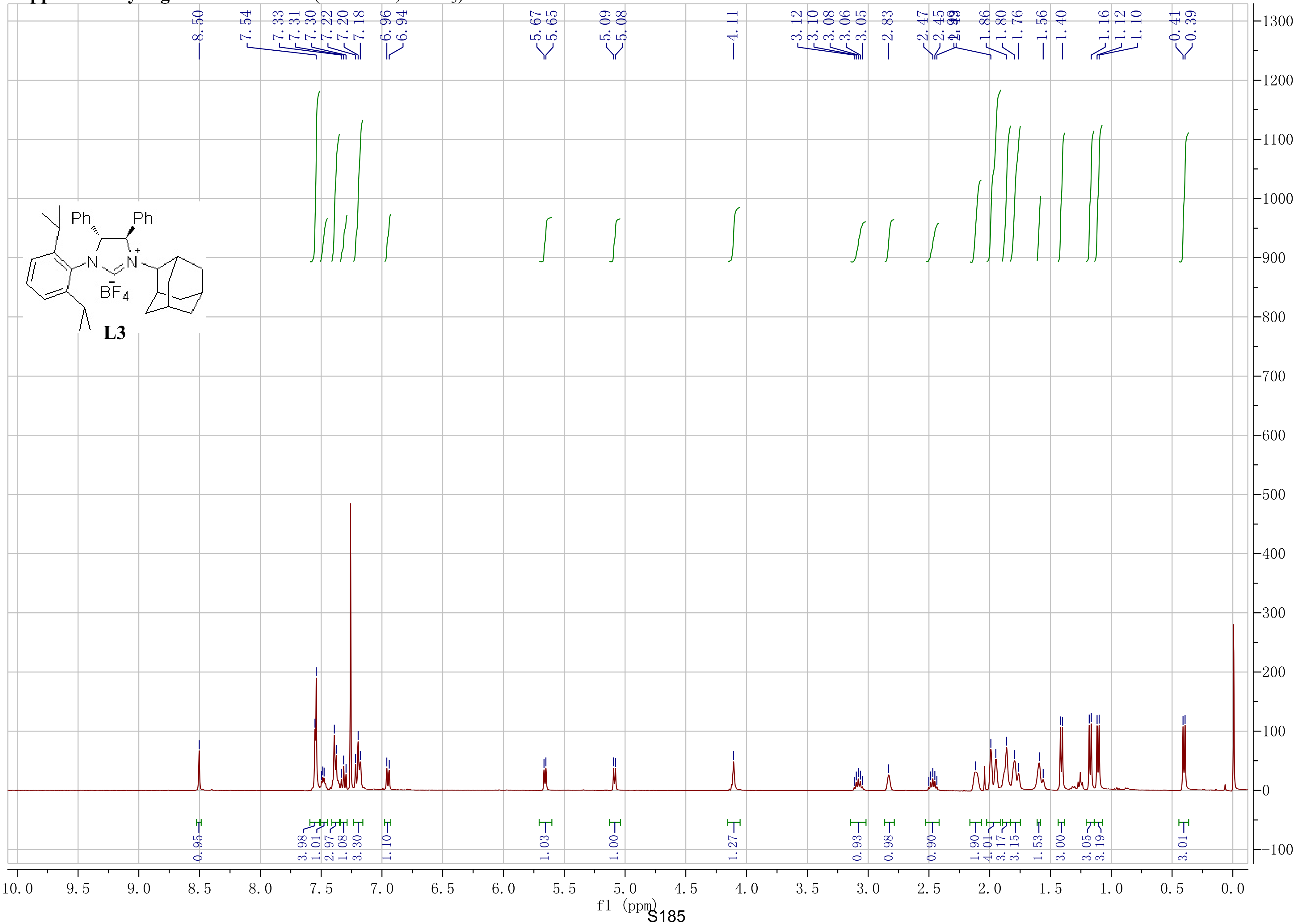

Supplementary Figure 156 ^{13}C NMR (101 MHz, $CDCl_3$) of **L3**

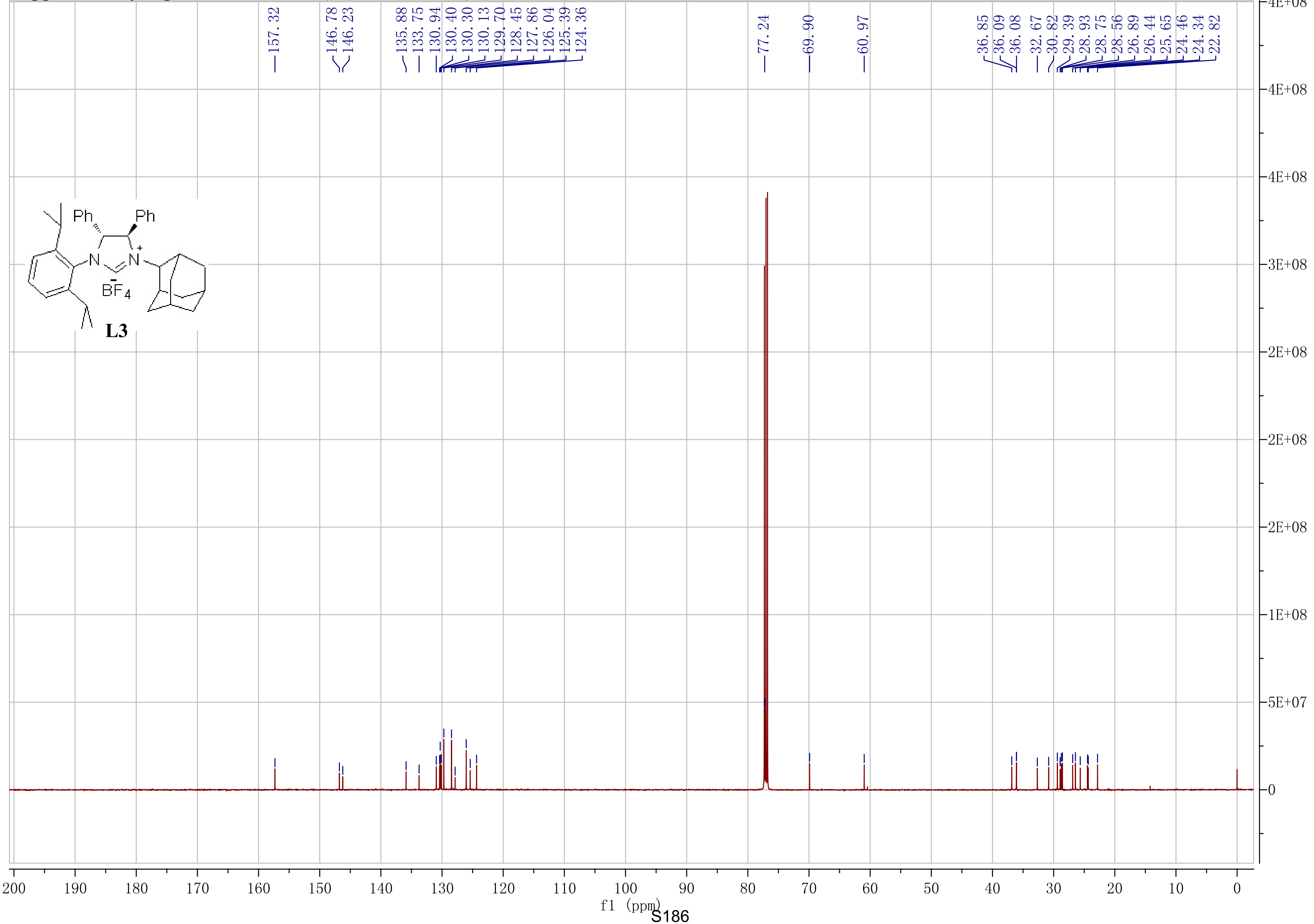

Supplementary Figure 157 ^{1}H NMR (400 MHz, $CDCl_3$) of **L4**

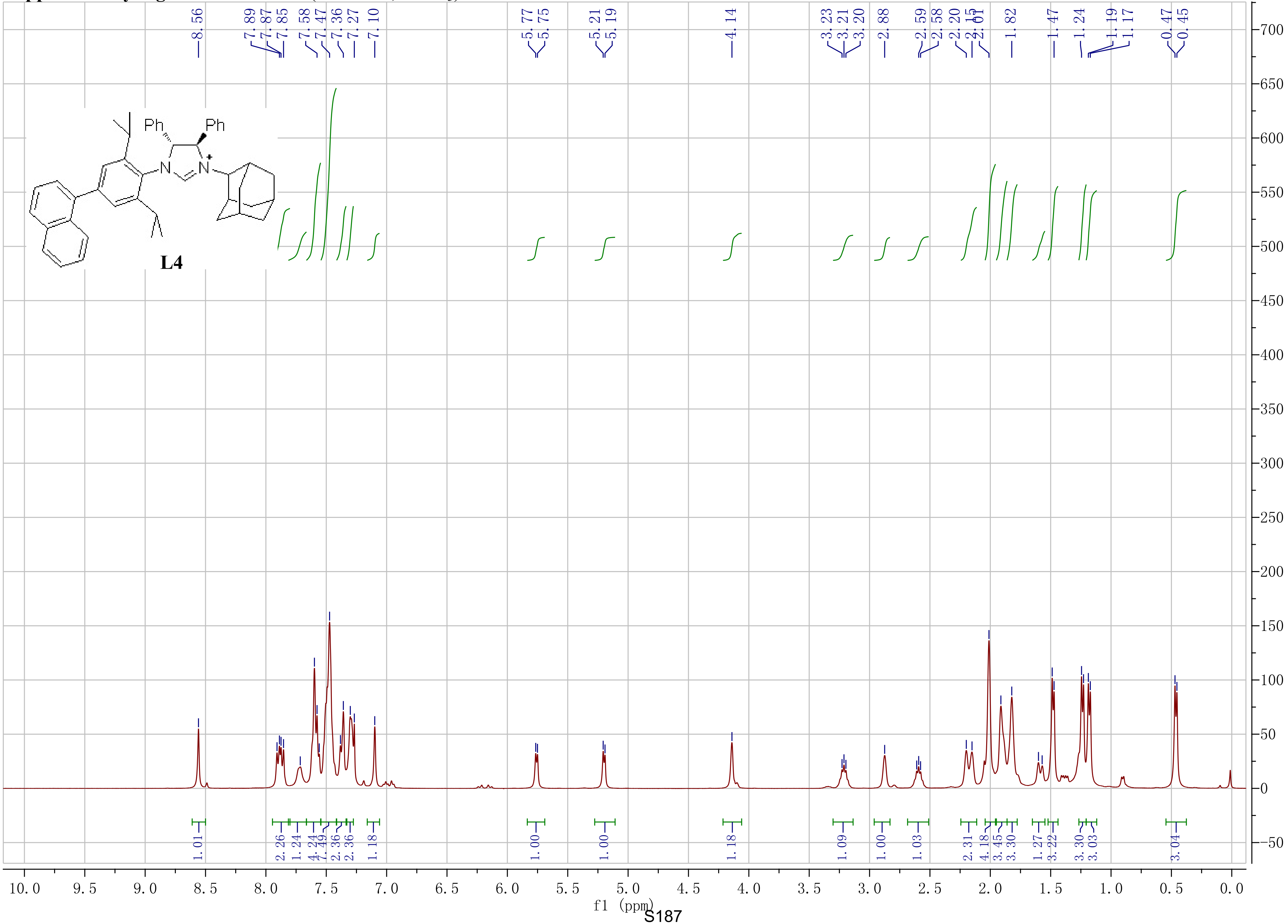

Supplementary Figure 158 ^{13}C NMR (101 MHz, $CDCl_3$) of **L4**

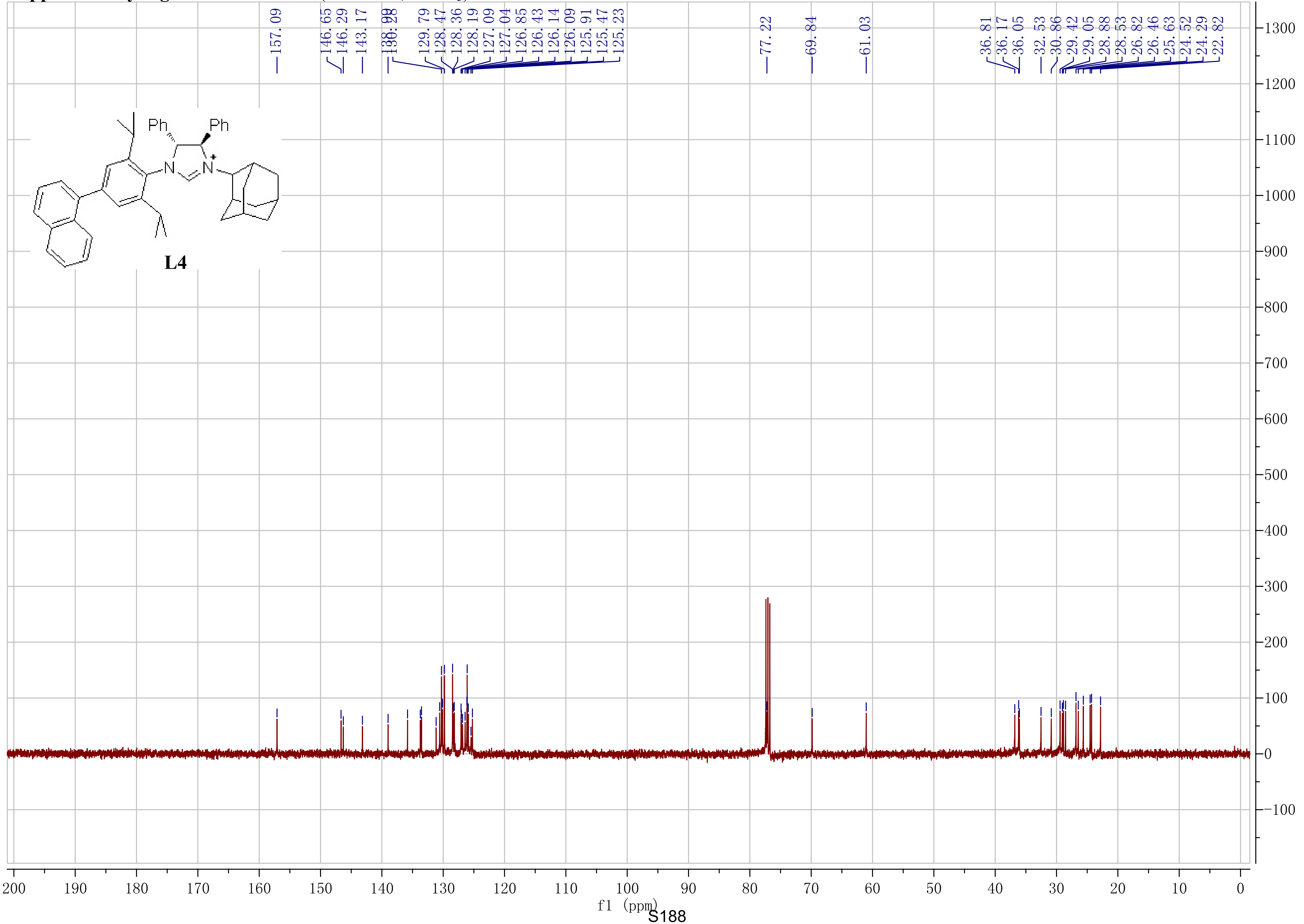

Supplementary Figure 159 ^{1}H NMR (400 MHz, $CDCl_3$) of **L5**

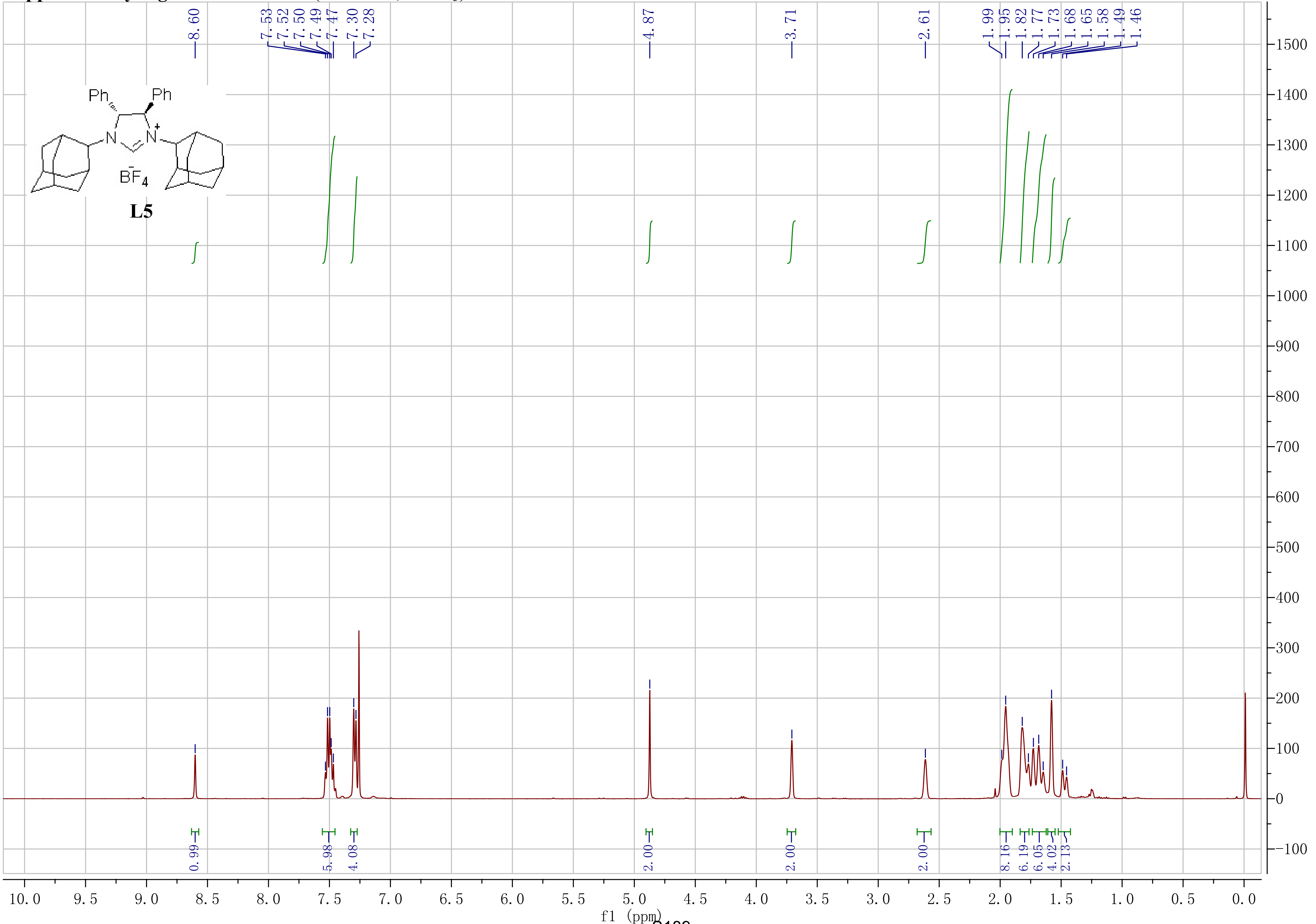

Supplementary Figure 160 ^{13}C NMR (101 MHz, $CDCl_3$) of **L5**

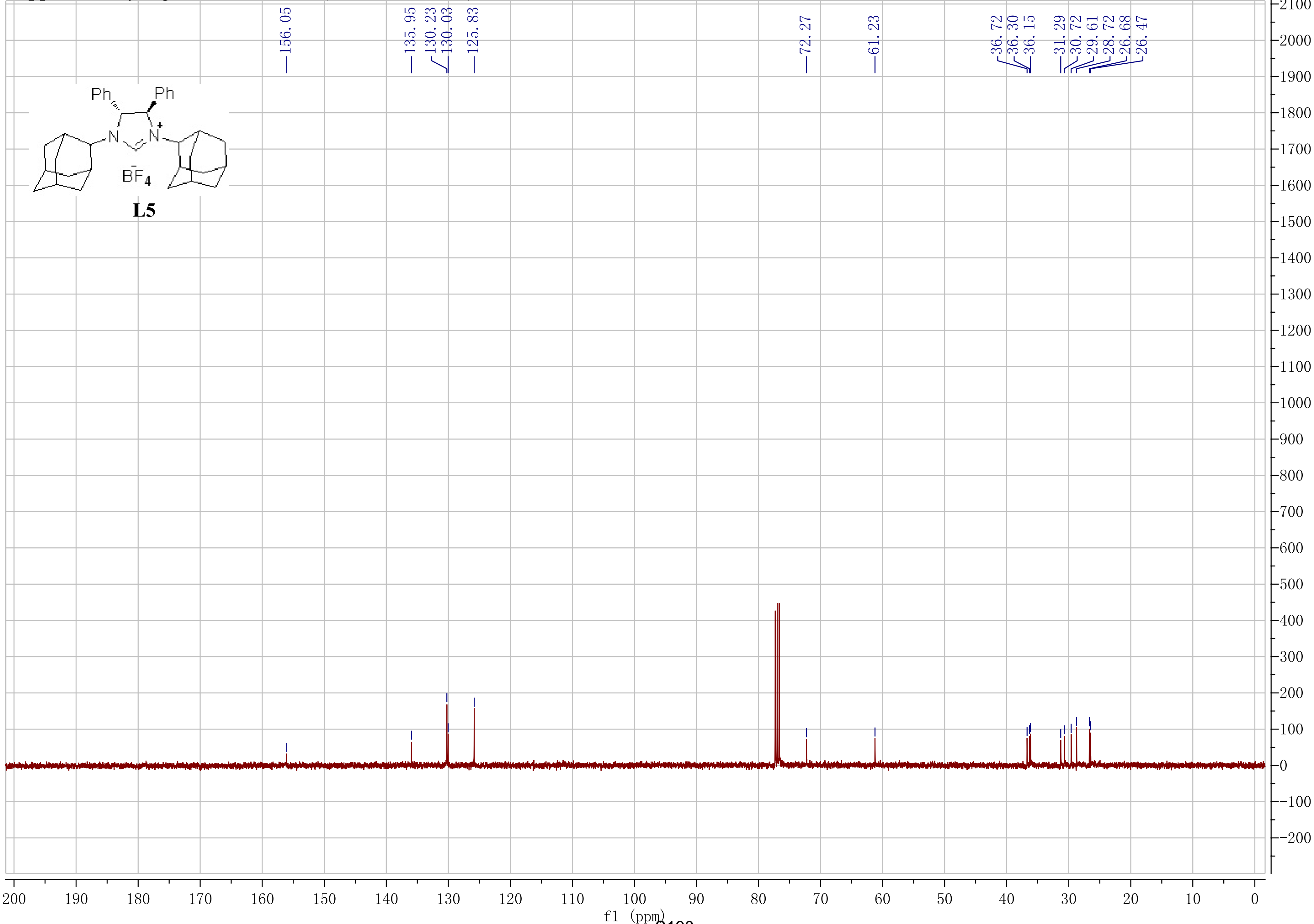

Supplementary Figure 161 ^{1}H NMR (400 MHz, $CDCl_3$) of **L6**

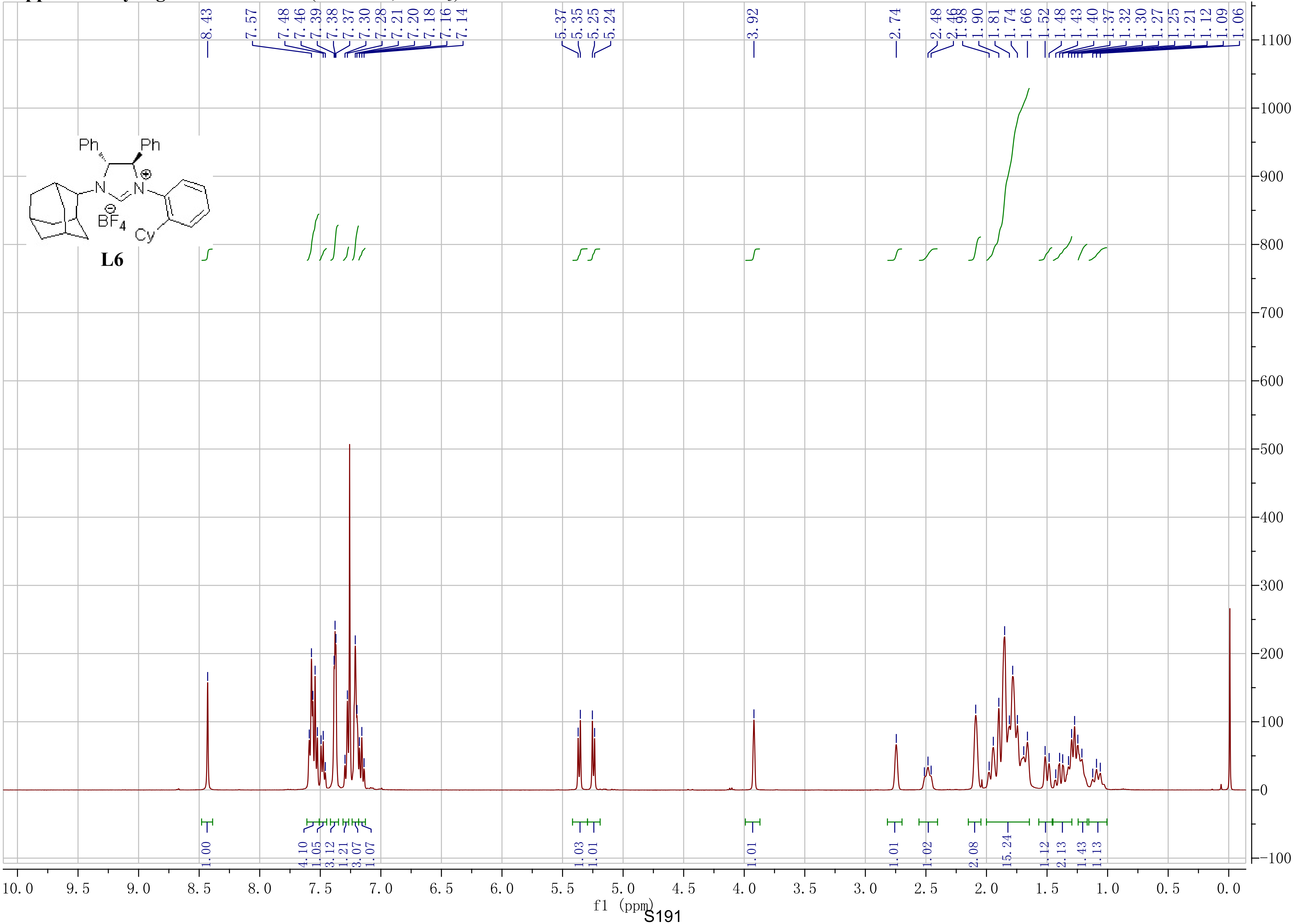

Supplementary Figure 162 ^{13}C NMR (101 MHz, $CDCl_3$) of **L6**

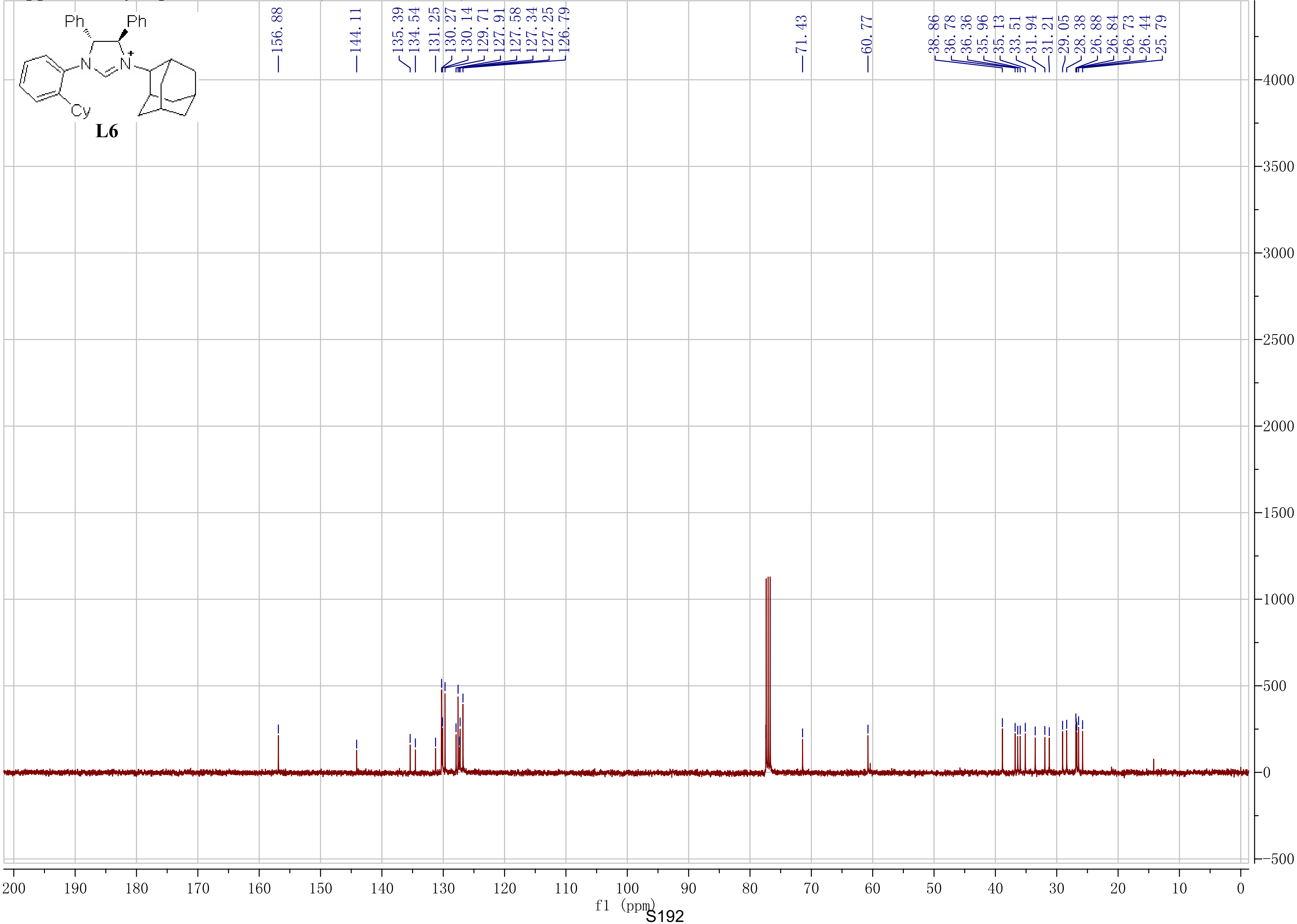

Supplementary Figure 163 ^{1}H NMR (400 MHz, $CDCl_3$) of **L7**

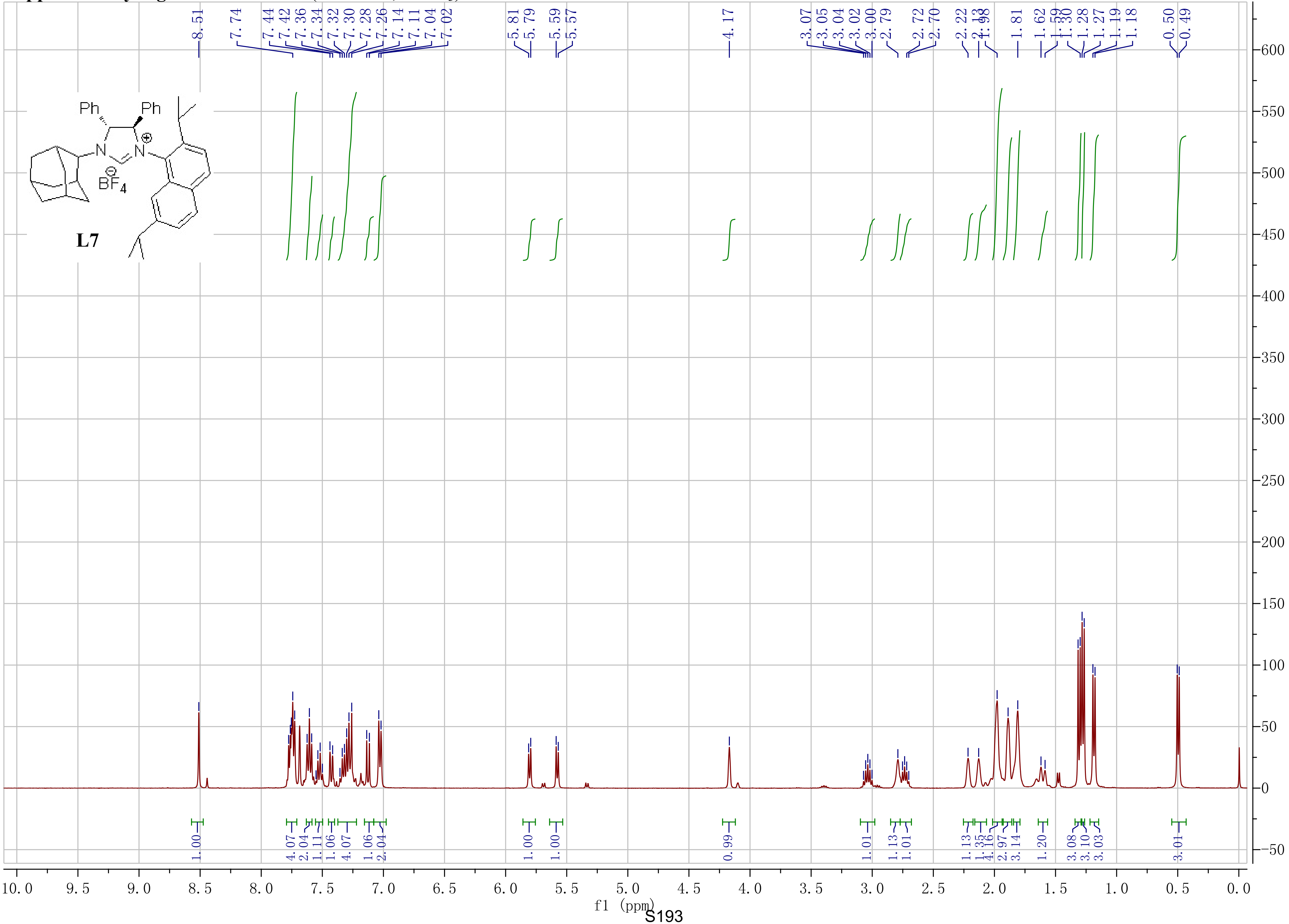

Supplementary Figure 164 ^{13}C NMR (101 MHz, $CDCl_3$) of **L7**

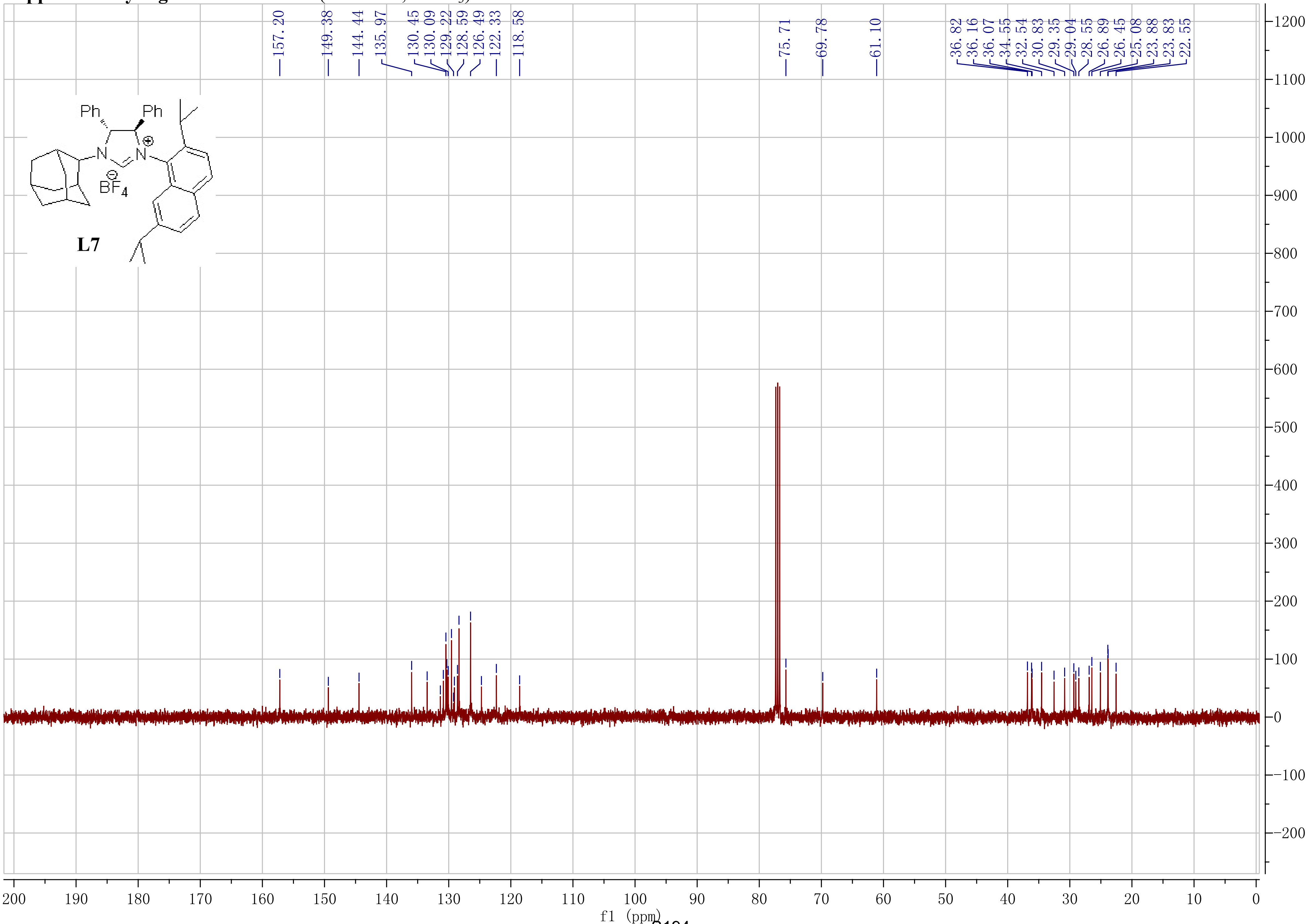

Supplementary Figure 165 ^{1}H NMR (400 MHz, $CDCl_3$) of **L8**

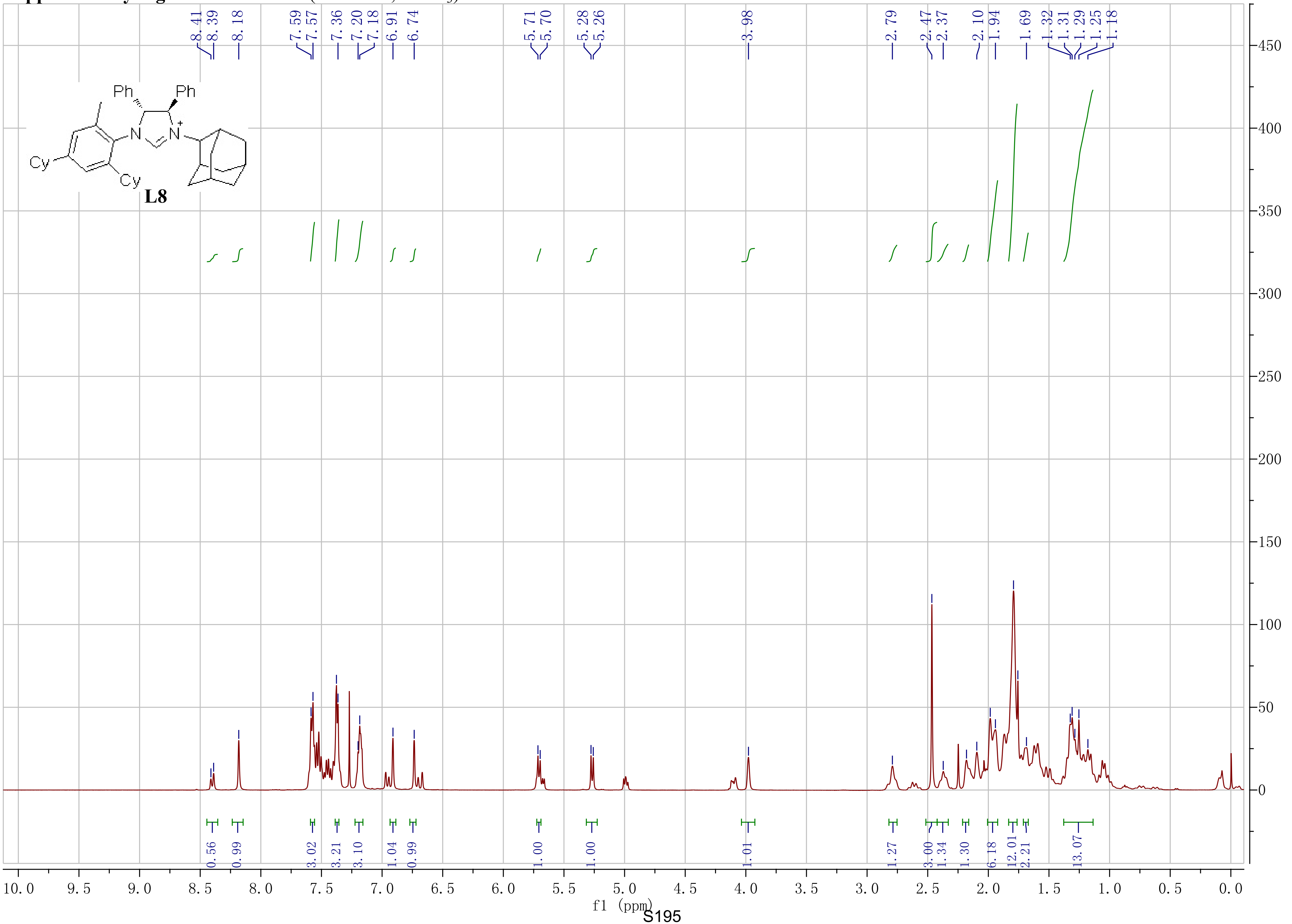

Supplementary Figure 166 ^{13}C NMR (101 MHz, $CDCl_3$) of **L8**

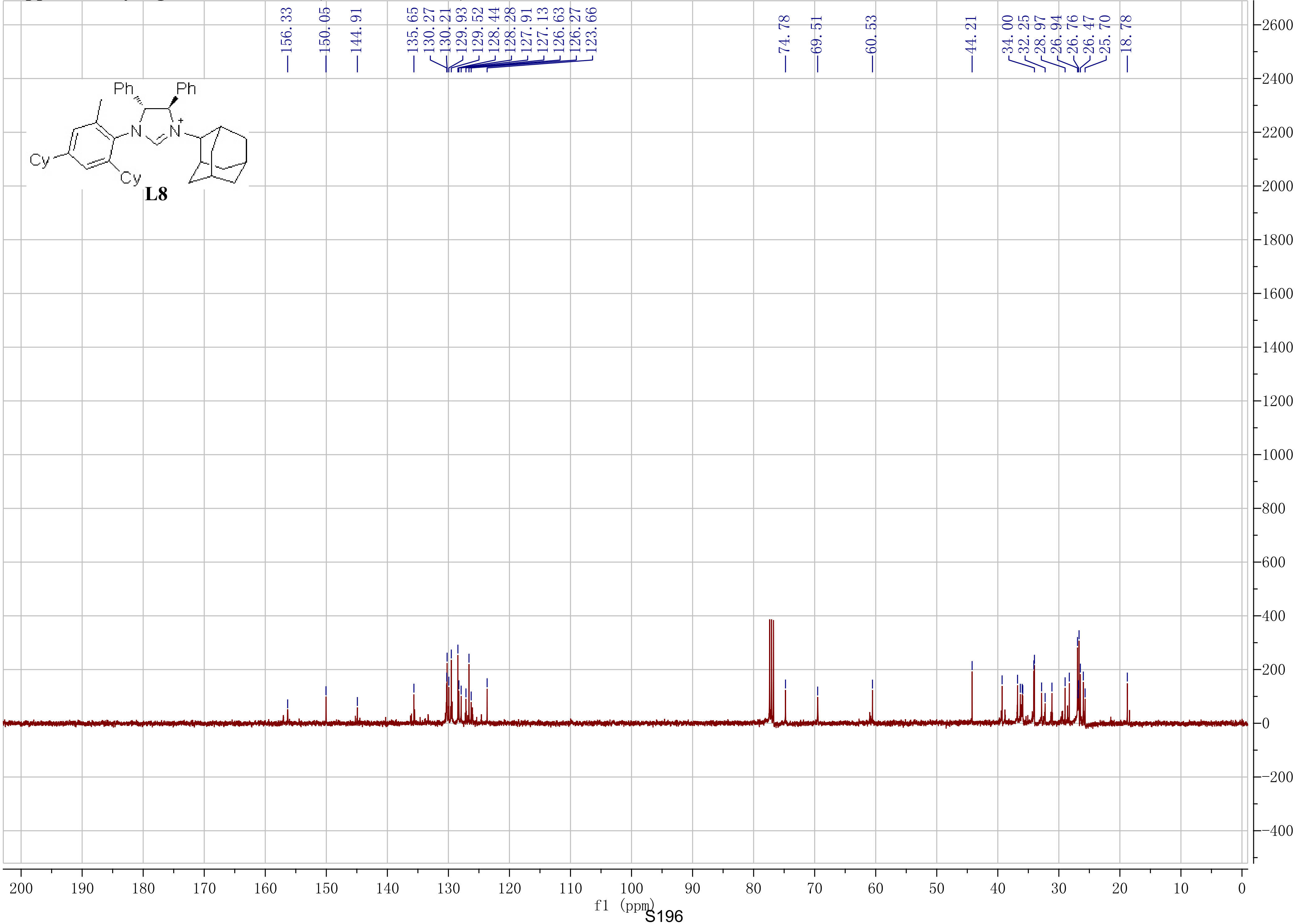

6. Supplementary References

1 Hu, J. et al. *Chem. Eur. J.* **20**, 3932-3938 (2014).

2 Yang, X.-L., Peng, X.-X., Chen, F. & Han. B. *Org. Lett.* **18**, 2070-2073 (2016.)

3 Chaulagain, M. R., Sormunen, G. J. & Montgomery. J. *J. Am. Chem. Soc*. **129**, 9568-9569 (2007).

4 Loup, J. et al. *Angew.Chem. Int.Ed.* **56**, 14197-14201 (2017).

5 W.-Y. Wang. et al. *Org. Lett.* **20**, 4773-4776 (2018).

6 Zhang, G.-P. et al. *Chem. Commun*. **55**, 6449-6452 (2019).

7 Butcher, T. W., Yang, J. L. & Hartwig. J. F. *Org. Lett.* **22**, 6805−6809 (2020) .
